# Implementation and dissemination of home- and community-based interventions for informal caregivers of people living with dementia: a systematic scoping review

**DOI:** 10.1186/s13012-023-01314-y

**Published:** 2023-11-08

**Authors:** Eden Meng Zhu, Martina Buljac-Samardžić, Kees Ahaus, Nick Sevdalis, Robbert Huijsman

**Affiliations:** 1 Erasmus School of Health Policy & Management, PO Box 1738, 3000 DR Rotterdam, The Netherlands; 2 Centre for Behavioural and Implementation Science Interventions, National University of Singapore, Singapore, Singapore

**Keywords:** Implementation science, Dementia, Informal caregiver, Community-based care

## Abstract

**Background:**

Informal caregivers of people with dementia (PwD) living at home are often the primary source of care, and, in their role, they often experience loss of quality of life. Implementation science knowledge is needed to optimize the real-world outcomes of evidence-based interventions (EBIs) for informal caregivers. This scoping review aims to systematically synthesize the literature that reports implementation strategies employed to deliver home- and community-based EBIs for informal caregivers of PwD, implementation outcomes, and the barriers and facilitators to implementation of these EBIs.

**Methods:**

Embase, MEDLINE, Web of Science, and Cochrane Library were searched from inception to March 2021; included studies focused on “implementation science,” “home- and community-based interventions,” and “informal caregivers of people with dementia.” Titles and abstracts were screened using ASReview (an innovative AI-based tool for evidence reviews), and data extraction was guided by the ERIC taxonomy, the Implementation Outcome Framework, and the Consolidated Framework for Implementation Science Research; each framework was used to examine a unique element of implementation.

**Results:**

Sixty-seven studies were included in the review. Multicomponent (26.9%) and eHealth (22.3%) interventions were most commonly reported, and 31.3% of included studies were guided by an implementation science framework. Training and education-related strategies and provision of interactive assistance were the implementation strategy clusters of the ERIC taxonomy where most implementation strategies were reported across the reviewed studies. Acceptability (82.1%), penetration (77.6%), and appropriateness (73.1%) were the most frequently reported implementation outcomes. Design quality and packaging (intervention component suitability) and cosmopolitanism (partnerships) constructs, and patient’s needs and resources and available resources (infrastructure) constructs as per the CFIR framework, reflected the most frequently reported barriers and facilitators to implementation.

**Conclusion:**

Included studies focused largely on intervention outcomes rather than implementation outcomes and lacked detailed insights on inner and outer setting determinants of implementation success or failure. Recent publications suggest implementation science in dementia research is developing but remains in nascent stages, requiring future studies to apply implementation science knowledge to obtain more contextually relevant findings and to structurally examine the mechanisms through which implementation partners can strategically leverage existing resources and regional networks to streamline local implementation. Mapping local evidence ecosystems will facilitate structured implementation planning and support implementation-focused theory building.

**Trial Registration:**

Not applicable.

**Supplementary Information:**

The online version contains supplementary material available at 10.1186/s13012-023-01314-y.

Contribution to literature

- Twenty-one of the 67 studies focused on the implementation of home- and community-based, non-pharmacological, evidence-based interventions for informal caregivers of people with dementia were guided by implementation science frameworks, which suggests a disconnect between dementia research and implementation science.
- “Train and educate stakeholders” and “provide interactive assistance” clusters contained the most frequently employed implementation strategies, which reveals discrepancies with previous feasibility and importance ratings.
- We propose the need to supplement implementation science with knowledge from integrated care research, which prioritizes multi-level, cross-sector partnerships in dementia care across all stages of implementation and leverages stakeholders’ experiential knowledge, networks, and resources.

## Background

Recent forecasts estimate 152.8 million global cases of dementia by 2050, which will increasingly strain health systems that already struggle to meet current elderly care demands [1]. Recent studies suggest that home- and community-based services (HCBS) for people with dementia (PwD), facilitated with primary support from informal caregivers, present a cost-effective and patient-preferred alternative to institutionalization [2, 3]. Informal caregivers are identified as family members, friends, and neighbors of PwD, and their roles consist of facilitating instrumental activities of daily living, care management, and care continuity [4]. In 2019, the World Health Organization reported an estimate of 133 billion hours of global unpaid informal dementia care [5]. Additionally, Rabarison and colleagues [6] estimated that the 3.2 million informal dementia caregivers, based in North America, included in their review provided unpaid care valued at US $41.5 billion, highlighting the social and economic value of informal care.

To succeed in their role, informal caregivers also require support to reduce personal experiences of stress, anxiety, burnout, and depression, commonly exacerbated by their caregiving demands [7, 8]. Cheng and Zhang [9] produced a meta-review, synthesizing over 500 individual studies on the effectiveness of non-pharmacological evidence-based interventions (EBI) that support informal caregivers of PwD, which revealed EBIs can effectively reduce caregivers’ psychological distress and strengthen dyadic communication and coping skills, improving their overall quality of life [9–12]. Types of caregiver-focused interventions include psychoeducation, eHealth, support group interventions, case management and care coordination, respite care, and exercise [9]. However, despite the multitude of EBIs that effectively support informal caregivers, the pertinent details surrounding the implementation of these interventions remain unclear.

The effectiveness of EBIs is merely one component that cannot be studied in isolation but must be considered among other contextual variables across multiple levels within the local health system and implementation setting, including clients, providers, organizations, and communities [13, 14]. EBIs must be systematically implemented within HCBS to strengthen caregiver resilience, improve quality of life, and delay institutionalization of PwD [15, 16]. This goal can be actualized by applying implementation science knowledge to steer dementia care research and practice.

### Application of implementation theories, models, and frameworks

Implementation theories, models, and frameworks, hereby referred to as frameworks, allow researchers to structurally examine the implementation and sustainment processes and the contextual determinants (i.e., barriers and facilitators) to implementation [17]. The Consolidated Framework for Implementation Science Research (CFIR) is a comprehensive determinant framework that uses a multilevel, multidimensional approach to identify “what works, where, and why”, and the breadth of constructs provides the most coverage to accurately reflect the complex nature of real-world implementation [18–20]. The CFIR has been widely applied in both empirical research [21] and in a systematic review [22] to structurally assess the barriers and facilitators to implementation.

In addition, the process of implementation can be systematically studied using the refined Expert Recommendations for Implementing Change (ERIC) taxonomy, which consists of 73 discrete implementation strategies that provide a structured set of “building blocks” used to homogenize implementation reporting and tailor a multicomponent implementation strategy [23]. Waltz and colleagues [24] grouped these strategies into nine clusters and rated each discrete strategy based on its perceived feasibility and importance. Implementation strategies act via mechanisms, which explain *how* the implementation strategy has an effect by describing the set of strategic actions that occur [25].

The Implementation Outcomes Framework (IOF) can be used to evaluate the degree of implementation success and the effectiveness of selected implementation strategies and to provide important distinction between intervention failure and implementation failure. The IOF explores the *acceptability*, *adoption*, *appropriateness*, *feasibility*, *fidelity*, *implementation cost*, *penetration*, and *sustainability* of the EBI [26]. The ERIC taxonomy and the IOF have both been applied to specify and compare implementation strategies and outcomes in empirical implementation research [27, 28] and in recent literature reviews [29–31]. The combination of the ERIC taxonomy, IOF, and CFIR allows researchers to comprehensively examine the multiple levels and stages of implementation.

### Study aims

Lourida and colleagues [32], and Bennet and colleagues [33], synthesized the implementation literature of EBIs for PwD and, indirectly, their caregivers, and each study determined an urgent need for additional synthesized literature, guided by implementation science frameworks, on the implementation of home- and community-based EBIs that support informal caregivers of PwD. This scoping review combines three implementation science frameworks to create a detailed and systematic synthesis of implementation science literature, to construct a comprehensive understanding of implementation, reflective of multifaceted, real-world complexities. This facilitates the understanding of implementation strategies employed, outcomes reported, and the contextual barriers and facilitators to implementation. Accordingly, this scoping review aims to accomplish the following objectives:

1. Guided by CFIR, map, describe, and synthesize the contextual barriers and facilitators to implementation of EBIs.
2. Guided by the ERIC taxonomy, map, describe, and synthesize the implementation strategies employed to deliver home- and community-based EBI that support informal caregivers of PwD.
3. Guided by the IOF, map, describe, and synthesize the implementation outcomes that have been used to report and measure the success (or failure) of implementation of these EBIs.

## Methods

**Fig. 1:**
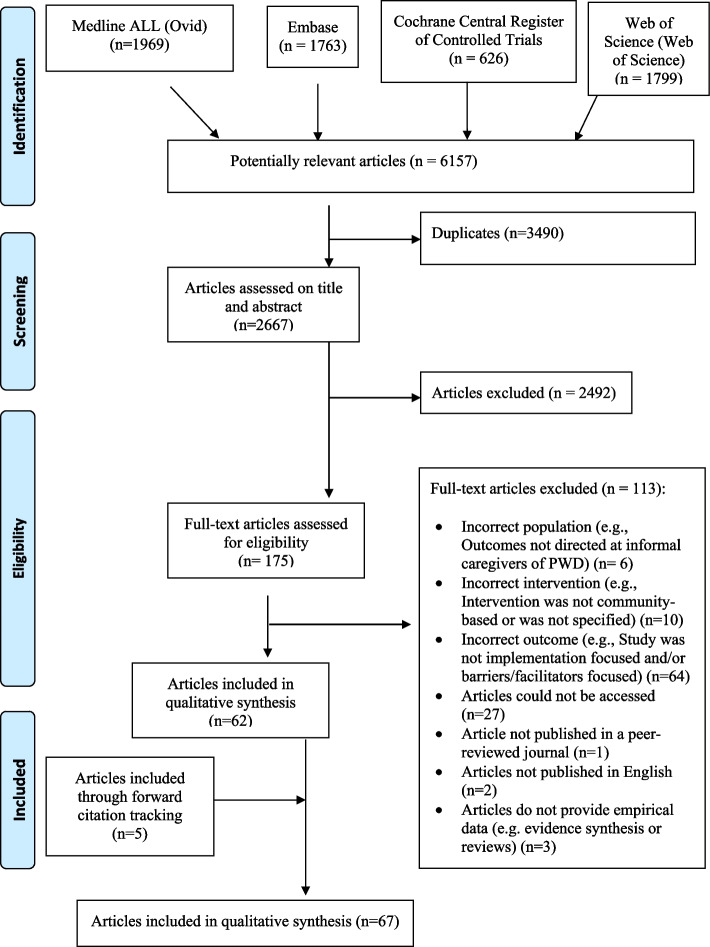
PRISMA diagram illustrates the process used to identify eligible studies

Arksey and O’Malley’s scoping review framework [34] and the Preferred Reporting Items for Systematic Reviews and Meta-Analyses Extension for Scoping Reviews (PRISMA-ScR) reporting recommendations were used to guide this review [35] (see Fig. 1 in Additional file 1. Method Overview). The scoping review protocol for this article [36], published in January 2022, provides a detailed overview of this review’s methodological steps and justifications at each stage; therefore, the methods are summarized in the sections that follow.

### Study eligibility criteria

The review included studies that focused on home- and community-based EBIs that support informal caregivers of PwD, which a) explicitly reported the implementation strategies used and implementation outcomes examined and/or b) explicitly reported the barriers and facilitators to implementation of EBIs. Studies were excluded if they examined EBIs that primarily focused on supporting the PwD or were delivered outside of the HCBS settings (e.g., institutionalized care, acute care).

### Information source and search strategy

The research team, with support from a specialized medical librarian, developed a full search strategy surrounding four key words: “dementia,” “informal caregivers,” “intervention,” and “implementation and dissemination” (see Additional file 2. Search strategy). Following, literature search was conducted across Embase, MEDLINE (Ovid), Web of Science, and Cochrane Central Register of Controlled trials (Wiley) to include all peer-reviewed studies, written in English, published from inception to 08 March 2021. Critical appraisal of included texts was performed by two reviewers (E. M. Z. and M. B.) using the Mixed-Methods Assessment Tool-version 2018 (MMAT), which is used to appraise the quality of empirical research designs and the comprehensiveness of data reporting [37].

### Study selection

In *title and abstract screening* stage, all relevant publications identified were imported into ASReview (https://asreview.nl/), an artificial-intelligence-aided tool that sequentially presented all imported publications to the reviewer from most to least relevant [38]. Previous studies indicated that ASReview’s algorithm could detect 95% of the final included publications in their study within the first 20% of publications presented, which significantly reduced time spent screening titles and abstracts while effectively maintaining result quality and integrity [39].

The first author (E. M. Z.) programmed the tool by screening 10 randomized (trial) publications and manually screened all imported titles and abstracts to completion. Following, the second author (M. B. S.) only screened the titles and abstracts of studies excluded by the first author to avoid false negatives. Given the tool’s capabilities, the second author stopped screening after 50 successively excluded studies, which was the team’s predetermined terminal point [36]. Following, the full texts of all included publications were assessed by both the first and second reviewers to exclude false positives. Any disagreements between the two authors were resolved by the third (K. A.) and fifth author (R. H.). Lastly, the reference lists of final included studies were checked to detect additional publications.

### Data extraction

*Data extraction*, *summarizing*, and *collating* process were conducted by the first and second author using a consensus approach, with regular discussion with all co-authors. A first table, guided by the domains and (sub)constructs of the CFIR, was used to extract and chart the identified barriers and facilitators. A second table was constructed based on the ERIC taxonomy and the nine clusters of implementation strategies reported in the literature. The first author identified detailed actions and mechanisms reported within each study and then “translated” and “matched” each with its corresponding discrete implementation strategies and respective clusters within the ERIC taxonomy. For example, a reported mechanism, such as “provide alternative mode of service delivery,” would “match” the discrete strategy “promote adaptability (ERIC 51)” found in “adapt and tailor to context (Cluster 3).” A third table, guided by the IOF descriptions, was also developed to systematically extract and chart the data for implementation outcomes reported. Prior to data extraction, the first author trialed the three unique data extraction tables on 10 random studies and made iterative refinements to each table after discussion with the research team.

Upon team consensus, the implementation strategies, outcomes, and barriers and facilitators to implementation from included studies were extracted by the first author (E. M. Z.). Categorization and “matching” of extracted data were reviewed for accuracy and confirmed by the second author (M. B. S.); any disagreements between reviewers at this stage were resolved by discussion until consensus was achieved. Additionally, study characteristics, including country of study origin, research design, type of intervention, target population, outcomes reported, and frameworks applied*,* were also extracted and synthesized. Further details on the methodology can be found in Fig. 2 of Additional file 1*.*

## Results

The full search yielded 2667 de-duplicated publications, 175 full-text publications were assessed for eligibility, and the reference lists of 62 publications were searched for additional relevant literature, which identified five additional publications. Sixty-seven publications were included in the final qualitative synthesis. Using the MMAT-version 2018, 56 of 67 studies were rated 100%, and 11 studies were rated 80%. The study exclusion process can be found in Fig. 1, and details of study characteristics and findings can be found in Table 1, found below, and Table 1 in Additional file 3.

**Table 1:** Overview of results from included studies

EBI title	Author(s), year/country of study origin	Implementation clusters (1–9)	ERIC taxonomy discrete strategies (1–73)	Implementation outcomes
*eHealth: electronic health interventions (eHealth) are uniquely delivered through various digital/technological mediums (e.g., computer, Internet, with or without human interaction) and can provide education, counseling, and supportive elements of other types of interventions* *Example: iSupport provides education and support for caregivers on a digital platform, equipped with an integrated caregiver network, accessible in remote areas*				
Caring for carers of people with dementia study	Banbury et al. (2019)/Australia [40]	1—Use evaluative and iterative strategies	4	Acceptability Appropriateness Penetration Sustainability
		2—Provide interactive assistance	33, 8	
		3—Use evaluative and iterative strategies	51, 63	
iSupport	Baruah et al. (2020)/India [41]	No implementation strategies identified	n/a	Acceptability Appropriateness Penetration
	Teles et al. (2020)/Portugal [42]	3—Adapt and tailor to context	51	Appropriateness Penetration
		5—Train and educate stakeholders	19, 29, 31	
	Xiao et al. (2020)/Australia [43]	3—Adapt and tailor to context	51, 63	Acceptability Appropriateness
		5—Train and educate stakeholders	29	
Partner in Balance	Boots et al. (2017)/the Netherlands [44]	1—Use evaluative and iterative strategies	5	Acceptability Appropriateness Feasibility Penetration Sustainability
		2—Provide interactive assistance	33	
		3—Adapt and tailor to context	51	
		4—Develop stakeholder interrelationships	6, 52	
		5—Train and educate stakeholders	71, 43, 16, 55, 19, 31	
		6—Support clinicians	59	
		7—Engage consumers	69	
		9—Change infrastructure	12	
InLife	Dam et al. (2019)/the Netherlands [45]	1—Use evaluative and iterative strategies	46	Acceptability Adoption Appropriateness Penetration Sustainability
		2—Provide interactive assistance	8	
		3—Adapt and tailor to context	63	
		5—Train and educate stakeholders	31	
		7—Engage consumers	69	
		9—Change infrastructure	12	
eMR-ABC	Frame et al. (2013)/USA [46]	1—Use evaluative and iterative strategies	26	Adoption Appropriateness Sustainability
		3—Adapt and tailor to context	51	
		5—Train and educate stakeholders	71	
		6—Support clinicians	32	
		9—Change infrastructure	12	
Alzheimer’s Caregiver Support Online (AlzOnline)	Glueckauf and Loomis (2003)/USA [47]	1—Use evaluative and iterative strategies	5, 46, 4, 18	Appropriateness Penetration Sustainability
		2—Provide interactive assistance	33	
		3—Adapt and tailor to context	51, 63	
		4—Develop stakeholder interrelationships	6, 38	
		5—Train and educate stakeholders	29, 43, 31	
		9—Change infrastructure	11	
iGeriCare (clinician’s perspective)	Levinson et al. (2020)/Canada [48]	1—Use evaluative and iterative strategies	4	Acceptability Adoption Appropriateness Penetration Sustainability
		4—Develop stakeholder interrelationships	38	
		5—Train and educate stakeholders	19, 31, 43, 29	
Tele.TanDem	Meichsner et al. (2018)/Germany [49]	2—Provide interactive assistance	33	Acceptability Feasibility
		3—Adapt and tailor to context	51	
		5—Train and educate stakeholders	29, 31, 55	
		9—Change infrastructure	13	
RAM (remote activity monitoring)	Mitchell et al. (2017) published in 2020/USA [50]	2—Provide interactive assistance	33, 54	Acceptability Appropriateness Penetration Sustainability
		3—Adapt and tailor to context	51	
		9—Change infrastructure	11, 12	
Cuidate Cuidador	Pagan-Ortiz et al. (2014)/USA [51]	1—Use evaluative and iterative strategies	18, 4	Acceptability Appropriateness Penetration
		2—Provide interactive assistance	8	
		3—Adapt and tailor to context	51, 63, 67	
		4—Develop stakeholder interrelationships	52	
		5—Train and educate stakeholders	29, 43, 31	
		7—Engage consumers	69	
Mastery over dementia (MoD)	Pot et al. (2015)/the Netherlands [52]	1—Use evaluative and iterative strategies	5	Acceptability Appropriateness Penetration
		2—Provide interactive assistance	33	
		5—Train and educate stakeholders	29, 43, 31, 55, 19	
		6—Support clinicians	59	
		7—Engage consumers	39	
		9—Change infrastructure	12, 13	
Partner in Sight (PsyMate)	van Knippenberg et al. (2017)/the Netherlands [53]	3—Adapt and tailor to context	51, 63	Acceptability Appropriateness Penetration
		5—Train and educate stakeholders	19, 71, 43	
		7—Engage consumers	50	
		9—Train and educate stakeholders	11, 12	
FamTechCare	Williams et al. (2020)/USA [54]	1—Use evaluative and iterative strategies	26	Acceptability Appropriateness Adoption Feasibility
		2—Provide interactive assistance	8, 33	
		5—Train and educate stakeholders	31, 43	
		6—Support clinicians	21, 59	
		9—Change infrastructure	11	
*Respite care: respite care provides caregivers with temporary relief through day care services* *Example: Adult day service (ADS) provides a safe environment for people with dementia and provides support resources for caregivers*				
Adult day care—On Lok project/Program of All-Inclusive Care for the Elderly (PACE)	Beisecker et al. (1996)/USA [55]	No implementation strategies identified	N/a	Acceptability Penetration
Caring for the caregiver	Brandao et al. (2016)/Portugal [56]	No implementation strategies identified	n/a	Acceptability Penetration
Adult day service (ADS)	Gaugler (2014)/USA [57]	6—Support clinicians	59	Acceptability Appropriateness
		7—Engage consumers	39, 50	
		9—Change infrastructure	13	
Adult day service Plus (ADS Plus)	Gitlin et al. (2019)/USA [58]	1—Use evaluative and iterative strategies	4, 5, 18, 23, 26, 56	Fidelity Implementation cost
		2—Provide interactive assistance	33	
		3—Adapt and tailor to context	63	
		4—Develop stakeholder interrelationships	35, 57	
		5—Train and educate stakeholders	71, 15, 19, 43, 29, 31	
		6—Support clinicians	59	
		8—Utilize financial strategies	2	
Adult day care (respite programming)	Roberts and Struckmeyer (2017)/USA [59]	No implementation strategies identified	N/a	Acceptability Appropriateness Implementation cost Sustainability
*Psychoeducation: psychoeducation interventions primarily provide education for caregivers regarding the physiological stages of dementia, care planning, behavior management, and self-care (e.g., managing anxiety and depression)* *Example: START (StrAtegies for RelaTives) consists of 8-week, dementia, individual psychological intervention designed for carers of people with dementia consisting of education about dementia, strategies to identify/manage behavior challenges, and planning for future needs*				
The booklet, Information for Families and Friends of People with Severe and End-Stage Dementia	Chang et al. (2010)/Australia [60]	1—Use evaluative and iterative strategies	4	Acceptability Appropriateness Penetration
		4—Develop stakeholder interrelationships	36, 52	
		5—Train and educate stakeholders	29	
START (StrAtegies for RelaTives)	Foley et al. (2020)/UK [61]	3—Adapt and tailor to context	63, 51	Acceptability Appropriateness Feasibility
		4—Develop stakeholder interrelationships	35	
		5—Train and educate stakeholders	19, 43	
		8—Utilize financial strategies	1	
		9—Change infrastructure	13	
	Sommerlad et al. (2014)/UK [62]	5—Train and educate stakeholders	43, 31	Acceptability Appropriateness Sustainability
		9—Change infrastructure	13	
Tele-Savvy for Dementia Caregivers/The Savvy Caregiver Program	Griffiths et al. (2015)/USA [63]	1—Use evaluative and iterative strategies	4, 46	Acceptability Appropriateness Fidelity Penetration Sustainability
		2—Provide interactive assistance	33	
		5—Train and educate stakeholders	19, 31, 43, 29	
		7—Engage consumers	50	
		9—Change infrastructure	11	
	Kovaleva et al. (2019)/USA [64]	3—Adapt and tailor to context	63	Acceptability Appropriateness Penetration Sustainability
		5—Train and educate stakeholders	19, 55, 29, 43, 31	
ANSWERS	Judge et al. (2010)/USA [65]	1—Use evaluative and iterative strategies	26, 27, 5, 56	Acceptability Appropriateness Fidelity
		3—Adapt and tailor to context	51	
		4—Develop stakeholder interrelationships	57	
		5—Train and educate stakeholders	19, 31, 71	
		9—Change infrastructure	13	
REACH II	Lykens et al. (2014)/USA [66]	1—Use evaluative and iterative strategies	4, 26, 27	Acceptability Appropriateness Penetration Sustainability
		2—Provide interactive assistance	54, 8	
		3—Adapt and tailor to context	51	
		4—Develop stakeholder interrelationships	52, 47, 6	
		5—Train and educate stakeholders	73, 71, 43, 31	
		6—Support clinicians	21, 59	
		9—Change infrastructure	12	
REACH into Indian country	Martindale-Adam et al. (2017)/USA [67]	1—Use evaluative and iterative strategies	61, 56, 26	Acceptability Appropriateness Adoption Implementation cost Penetration Sustainability
		2—Provide interactive assistance	33	
		3—Adapt and tailor to context	51, 63	
		4—Develop stakeholder interrelationships	72, 6, 40, 35	
		5—Train and educate stakeholders	71, 29, 31, 15	
		6—Support clinicians	30	
		7—Engage consumers	37, 69	
		8—Utilize financial strategies	34, 2, 70, 42	
		9—Change infrastructure	44, 13, 22, 62	
Star-C	McCurry et al. (2017)/USA [68]	1—Use evaluative and iterative strategies	5, 56, 14	Acceptability Appropriateness Adoption Feasibility Fidelity Penetration Sustainability
		2—Provide interactive assistance	33	
		3—Adapt and tailor to context	63	
		4—Develop stakeholder interrelationships	35, 7, 40, 52	
		5—Train and educate stakeholders	19, 29, 31, 43, 71	
		8—Utilize financial strategies	34	
		7—Engage consumers	69	
Medway Carers Course	Milne et al. (2014)/UK [69]	2—Provide interactive assistance	33	Acceptability Appropriateness Sustainability
		4—Develop stakeholder interrelationships	6	
		5—Train and educate stakeholders	29, 19, 31, 43	
		6—Support clinicians	59, 21	
CARES Dementia Basics Program	Pleasant et al. (2016)/USA [70]	4—Develop stakeholder interrelationships	52	Acceptability Appropriateness Penetration
		5—Train and educate stakeholders	43, 31	
		7—Engage consumers	50	
		9—Change infrastructure	13, 22	
Taking Care of YOU: Self-Care for Family Caregivers Toolkit	Smith and Graves (2020)/USA [71]	1—Use evaluative and iterative strategies	4	Acceptability Appropriateness Penetration
		2—Provide interactive assistance	33	
		4—Develop stakeholder interrelationships	64	
		5—Train and educate stakeholders	19, 29	
*Exercise: exercise interventions primarily consist of physical activities aimed to enhance the participants physical capacity* *Example: TACIT trial provided Tai Chi exercises to participants under the supervision of a professional trainer who provides safe guidance*				
Tai Chi for people with dementia (TACIT trial)	Barrado-Martin et al. (2019)/UK [72]	1—Use evaluative and iterative strategies	4, 56	Acceptability Appropriateness Penetration
		2—Provide interactive assistance	33	
		3—Adapt and tailor to context	63, 51	
		4—Develop stakeholder interrelationships	57	
		5—Train and educate stakeholders	31, 16, 29, 19	
		9—Change infrastructure	12	
	Barrado-Martin et al. (2020)/UK [73]	2—Provide interactive assistance	33	Acceptability Appropriateness Penetration
		4—Develop stakeholder interrelationships	57	
		5—Train and educate stakeholders	55, 31, 43	
		9—Change infrastructure	11, 12	
Reducing Disability in Alzheimer Disease (RDAD) program	Prick et al. (2014)/the Netherlands [74]	1—Use evaluative and iterative strategies	56	Acceptability Appropriateness Feasibility Penetration Sustainability
		3—Adapt and tailor to context	63, 51	
		4—Develop stakeholder interrelationships	52	
		5—Train and educate stakeholders	19, 31	
		6—Support clinicians	30	
		7—Engage consumers	69	
		8—Utilize financial strategies	49	
		9—Change infrastructure	13	
*Care coordination and case management: care coordination and case management interventions provide caregivers with care consultants who support with case management, care planning, referrals to resources, and continuity of care for people with dementia* *Example: Partners in Dementia Care is a care-coordination program integrating healthcare (Veteran Affairs Medical Centers) and community services (Alzheimer’s Association chapters) and supporting veterans with dementia and their caregivers*				
Cleveland Alzheimer’s managed care demonstration	Bass et al. (2003)/USA [75]	1—Use evaluative and iterative strategies	27, 4	Acceptability Appropriateness Feasibility Penetration
		2—Provide interactive assistance	33	
		3—Adapt and tailor to context	63	
		4—Develop stakeholder interrelationships	52	
		5—Train and educate stakeholders	55, 71, 19, 15, 43	
		6—Support clinicians	59, 21, 30	
		7—Engage consumers	39	
		8—Utilize financial strategies	49, 66, 34	
Partners in Dementia Care	Bass et al. (2014)/USA [76]	2—Provide interactive assistance	8	Acceptability Implementation cost Penetration Sustainability
		4—Develop stakeholder interrelationships	52, 6, 36, 72, 24	
		5—Train and educate stakeholders	73, 19, 71	
		6—Support clinicians	59, 30, 21	
		7—Engage consumers	50, 41	
		8—Utilize financial strategies	66	
		9—Change infrastructure	22, 12, 13	
Aged Care Assessment Teams	Bruce and Patterson (2000)/Australia [77]	No implementation strategies identified	n/a	Acceptability Appropriateness Penetration Sustainability
Community Outreach Education Program (COEP)	Connell and Kole (1999)/USA [78]	1—Use evaluative and iterative strategies	4, 56	Acceptability Penetration Sustainability
		4—Develop stakeholder interrelationships	47, 52, 17, 24, 64, 6, 38, 40, 48	
		5—Train and educate stakeholders	29, 15	
		6—Support clinicians	30, 59	
		7—Engage consumers	37, 69	
		8—Utilize financial strategies	1, 34	
		9—Change infrastructure	13	
Healthcare professional support	Laparidou et al. (2018)/UK [79]	2—Provide interactive assistance	33	Acceptability Penetration
		4—Develop stakeholder interrelationships	24, 52, 36, 64	
		6—Support clinicians	59, 21	
SUSTAIN program	Mavandadi et al. (2017)/USA [80]	1—Use evaluative and iterative strategies	4	Acceptability Appropriateness Penetration Sustainability
		2—Provide interactive assistance	33	
		3—Adapt and tailor to context	51, 63	
		4—Develop stakeholder interrelationships	52	
		5—Train and educate stakeholders	29, 43, 31, 55	
		8—Utilize financial strategies	34	
		9—Change infrastructure	13	
*Occupational therapy: occupational therapy interventions consist of training for activities of daily living and reminiscence, life story work, or cognitive stimulation therapy, for the cognitive, emotional, occupational, and functional aspects of dementia* *Example: “VALID-Occupational Therapy” consists of 10 tailored sessions with an occupational therapist, providing personalized goal setting, based upon assessment findings,and then supported practice and strategy use to achieve goals*				
Community Occupational Therapy in Dementia (COTiD) program	Burgess et al. (2020)/UK [81]	1—Use evaluative and iterative strategies	4	Acceptability Appropriateness Penetration
		3—Adapt and tailor to context	51	
		5—Train and educate stakeholders	19, 43	
		7—Engage consumers	50	
		9—Change infrastructure	13	
VALID-Occupational Therapy	Field et al. (2019)/UK [82]	1—Use evaluative and iterative strategies	4, 18	Acceptability Appropriateness Penetration
		3—Adapt and tailor to context	63, 51	
		4—Develop stakeholder interrelationships	52	
		5—Train and educate stakeholders	19	
		6—Support clinicians	21	
Environmental skill-building program (ESP)	Gitlin et al. (2010)/USA [83]	1—Use evaluative and iterative strategies	4, 18, 56	Acceptability Adoption Appropriateness Feasibility Fidelity Implementation cost Penetration Sustainability
		3—Adapt and tailor to context	63	
		4—Develop stakeholder interrelationships	17, 6, 25	
		5—Train and educate stakeholders	20, 73, 43, 71	
		8—Utilize financial strategies	49, 70	
*Multicomponent interventions: multicomponent interventions possess various types of interventions bundled into one program* *Example: New York University Caregiver Intervention (NYU-CI) consists of counseling meetings, caregiver consultancy, *ad hoc* calls, e-mail/telephone communication, information/referral, support groups*				
REACH	Burgio et al. (2001)/USA [84]	1—Use evaluative and iterative strategies	27, 5	Feasibility Sustainability
		2—Provide interactive assistance	53	
		3—Adapt and tailor to context	63, 51, 68	
		4—Develop stakeholder interrelationships	57, 52	
		5—Train and educate stakeholders	29, 31, 71, 43	
		8—Utilize financial strategies	34, 1	
		9—Change infrastructure	12, 22	
REACH OUT (offering useful treatments)—adaptation of REACH II for use in Area Agencies on Aging	Burgio et al. (2009)/USA [85]	1—Use evaluative and iterative strategies	27	Acceptability Adoption Appropriateness Feasibility Fidelity Penetration Sustainability
		2—Provide interactive assistance	33, 8	
		3—Adapt and tailor to context	63, 51	
		4—Develop stakeholder interrelationships	47, 24, 6, 40, 64, 25	
		5—Train and educate stakeholders	31, 16, 71, 43, 55	
		9—Change infrastructure	12, 11	
REACH-TX (a community-based translation of REACH II)	Cho et al. (2019)/USA [86]	1—Use evaluative and iterative strategies	4, 56	Acceptability Feasibility Penetration Sustainability
		4—Develop stakeholder interrelationships	47, 52	
		5—Train and educate stakeholders	71, 55, 15, 43, 31, 29	
iMCSP	Droes et al. (2019)/the Netherlands [87]	1—Use evaluative and iterative strategies	4, 18	Acceptability Appropriateness Implementation cost Penetration Sustainability
		4—Develop stakeholder interrelationships	24, 6, 35, 7	
		5—Train and educate stakeholders	19, 71	
		7—Engage consumers	69	
		8—Utilize financial strategies	1	
Care of Persons with Dementia in their Environment (COPE) integrated in Connecticut Home Care Program for Elders (CHCPE)	Fortinsky et al. (2016)/USA [88]	1—Use evaluative and iterative strategies	4, 18, 5, 26	Fidelity Penetration Sustainability
		2—Provide interactive assistance	33, 54, 53	
		3—Adapt and tailor to context	63	
		4—Develop stakeholder interrelationships	52, 6	
		5—Train and educate stakeholders	43, 29, 31, 16	
		6—Support clinicians	21, 32, 30, 59	
		9—Change infrastructure	11, 12	
NYU Caregiver-Adult Child Intervention	Gaugler et al. (2018)/USA [89]	2—Provide interactive assistance	33	Acceptability Appropriateness Feasibility
		5—Train and educate stakeholders	43, 19	
		7—Engage consumers	50	
Unforgettable (interactive museum program)	Hendriks et al. (2018)/the Netherlands [90]	1—Use evaluative and iterative strategies	61, 4	Acceptability Fidelity Sustainability
		3—Adapt and tailor to context	63, 51	
		4—Develop stakeholder interrelationships	57, 6, 24, 72, 36	
		5—Train and educate stakeholders	43, 71	
		6—Support clinicians	59, 30	
		7—Engage consumers	41	
RDAD	Menne et al. (2014)/USA [91]	1—Use evaluative and iterative strategies	5, 56	Appropriateness Feasibility Penetration Sustainability
		3—Adapt and tailor to context	51, 63	
		4—Develop stakeholder interrelationships	57, 64, 52	
		5—Train and educate stakeholders	31, 19, 71, 43, 29	
Savvy Caregiver + REACH II	Meyer et al. (2018)/USA [92]	2—Provide interactive assistance	33	Acceptability Adoption Appropriateness Penetration Sustainability
		4—Develop stakeholder interrelationships	38	
		5—Train and educate stakeholders	19	
		7—Engage consumers	39, 41	
Multicomponent non-pharmacological interventions (NPIs)	Milders et al. (2019)/UK [93]	1—Use evaluative and iterative strategies	5, 56	Acceptability Appropriateness Fidelity Implementation Cost Penetration Sustainability
		3—Adapt and tailor to context	51, 63	
		4—Develop stakeholder interrelationships	57, 64, 52	
		5—Train and educate stakeholders	31, 19, 71, 43, 29	
REACH VA	Nichols et al. (2011)/USA [94]	2—Provide interactive assistance	33	Acceptability Appropriateness Penetration Sustainability
		4—Develop stakeholder interrelationships	6	
		5—Train and educate stakeholders	71, 43, 31	
		6—Support clinicians	59	
		8—Utilize financial strategies	34	
	Nichols et al. (2016)/USA [95]	1—Use evaluative and iterative strategies	4, 56, 61, 14	Acceptability Adoption Feasibility Fidelity Penetration Sustainability
		2—Provide interactive assistance	8	
		4—Develop stakeholder interrelationships	47, 17, 35	
		5—Train and educate stakeholders	29, 19, 43, 31, 71	
		6—Support clinicians	59	
		7—Engage consumers	69, 37	
		8—Utilize financial strategies	34, 49	
		9—Change infrastructure	22, 44	
New York University Caregiver Intervention (NYUCI)—Minnesota Family Memory Care	Mittelman and Bartel (2014)/USA [96]	4—Develop stakeholder interrelationships	52, 35, 48	Acceptability Appropriateness Penetration Sustainability
		5—Train and educate stakeholders	19, 71	
		6—Support clinicians	59	
		7—Engage consumers	69	
		8—Utilize financial strategies	1, 34, 49	
		9—Change infrastructure	12	
SHARE Program	Orsulic-Jeras et al. (2019)/USA [97]	1—Use evaluative and iterative strategies	4	Acceptability Appropriateness Feasibility Fidelity Penetration
		2—Provide interactive assistance	33	
		5—Train and educate stakeholders	19, 59, 71, 55, 31, 43	
		6—Support clinicians	59	
New York University Caregiver Intervention (NYUCI)—Minnesota Family Memory Care	Paone (2014)/USA [98]	1—Use evaluative and iterative strategies	27	Acceptability Adoption Fidelity Implementation Cost Penetration Sustainability
		2—Provide interactive assistance	33	
		4—Develop stakeholder interrelationships	65	
		5—Train and educate stakeholders	55	
		6—Support clinicians	59	
		7—Engage consumers	69	
		8—Utilize financial strategies	1, 34	
		9—Change infrastructure	11, 22	
Maine Savvy Caregiver	Samia et al. (2014)/USA [99]	1—Use evaluative and iterative strategies	61	Acceptability Adoption Appropriateness Fidelity Penetration Sustainability
		4—Develop stakeholder interrelationships	64, 24, 52, 35, 36, 6	
		5—Train and educate stakeholders	29, 71, 73	
		7—Engage consumers	69	
		8—Utilize financial strategies	1	
		9—Change infrastructure	22	
REACH II — implemented in Scott & White Family Caregiver Program (a nonprofit collaborative healthcare system)	Stevens et al. (2012)/USA [100]	1—Use evaluative and iterative strategies	23, 56, 4	Acceptability Adoption Fidelity Implementation cost Penetration Sustainability
		2—Provide interactive assistance	33	
		3—Adapt and tailor to context	51, 63	
		4—Develop stakeholder interrelationships	52, 47, 24, 35, 6, 48, 64	
		5—Train and educate stakeholders	43, 19, 71, 29	
		6—Support clinicians	30, 32, 59	
		7—Engage consumers	50, 39	
		8—Utilize financial strategies	1	
		9—Change infrastructure	13	
Israeli NYUCI	Werner et al. (2020)/Israel [101]	4—Develop stakeholder interrelationships	6, 36, 57, 24	Appropriateness Adoption Penetration Sustainability
		5—Train and educate stakeholders	71, 29	
		8—Utilize financial strategies	34, 1	
		9—Change infrastructure	22	
*Support interventions: support interventions provide psychological, social, and emotional support to caregivers, facilitated in a safe environment by professionals* *Example: Meeting Center Support Program (MCSP) included educational meetings, support groups, social activities, and individual consultations*				
Meeting Center Support Program (MCSP/MEETINGDEM)	van Haeften-van Dijk et al. (2015)/the Netherlands [102]	1—Use evaluative and iterative strategies	4, 5, 18, 56	Adoption Feasibility Penetration Sustainability
		3—Adapt and tailor to context	51	
		4—Develop stakeholder interrelationships	35, 36, 64, 65, 6, 52, 35, 38, 47	
		5—Train and educate stakeholders	73, 19, 20, 71	
		6—Support clinicians	59	
	van Mierlo et al. (2017)/the Netherlands [103]	No implementation strategies identified	n/a	Adoption Penetration Sustainability
	Mazurek et al. (2019)/Poland [104]	1—Use evaluative and iterative strategies	61	Acceptability Appropriateness Feasibility Penetration
		2—Provide interactive assistance	33	
		3—Adapt and tailor to context	63, 51	
		4—Develop stakeholder interrelationships	35, 57, 38, 47, 17, 52	
		5—Train and educate stakeholders	43, 19, 55, 71	
		7—Engage consumers	37, 39	
		8—Utilize financial strategies	34	
		9—Change infrastructure	13	
	Meiland et al. (2005)/the Netherlands [105]	1—Use evaluative and iterative strategies	23	Adoption Penetration Sustainability
		3—Adapt and tailor to context	63	
		4—Develop stakeholder interrelationships	35, 6, 52, 24, 64, 47	
		5—Train and educate stakeholders	19, 55, 43	
		7—Engage consumers	39	
		9—Change infrastructure	13	
DemenTalent	van Rijn et al. (2019)/the Netherlands [106]	1—Use evaluative and iterative strategies	5, 27, 4	Adoption Feasibility Penetration Sustainability
		4—Develop stakeholder interrelationships	35, 57, 6, 52	
		5—Train and educate stakeholders	71	
		7—Engage consumers	39	

### Study characteristics

The 67 included studies were published between 1996 and 2021; more than half were published between 2016 and 2021 (*40/67*; *59.7%*). These studies reported 58 unique interventions, which were classified into one of eight types of interventions for informal caregivers of PwD based on the most prominent intervention components. This stratification was performed to examine the implementation characteristics of EBIs with clear commonalities to enhance the review’s usability. Multicomponent interventions (e.g., the combined use of case management, support groups, and eHealth tools) (18/67; 26.9%) [84–101] were most common, followed by eHealth (15/67; 22.3%) [40–54], psychoeducation (12/67; 17.9%) [60–71], care coordination and case management (6/67; 8.9%) [75–80], support interventions (5/67; 7.4%) [102–106], respite care (5/67; 7.4%) [55–59] exercise (3/67; 4.4%) [72–74], and occupational therapy (3/67; 4.4%) [81–83]. Studies originated mostly from the USA (*36/67*; *53.7%*), followed by The Netherlands (*11/67*; *16.4%*), the UK (*9/67*; *13.4%*), Australia (*4/67; 5.9%*), Portugal (2/67; 2.9%), and India, Israel, Poland, Germany, Canada (*each n* = *1*). The most common study designs were pre-posttest studies (38/67; 56.7%), followed by descriptive qualitative studies (20/67; 29.9%) and parallel convergent mixed-methods design (9/67; 13.4%).

### Use of implementation theories, models, and frameworks

Twenty-one articles were explicitly guided by an implementation framework (21/67; 31.34%). Ten unique frameworks were used, including adaptive implementation model [90, 102, 103, 105, 106], multimethod assessment process (MAP)/reflective adaptive process (RAP) [46], reach, efficacy, adoption, implementation, and maintenance (RE-AIM) [83, 98–100], Medical Research Council Framework [44, 45, 89], Fixsen and Blasé Implementation Process Model [67, 95], Consolidated Framework for Implementation Research [48], Leontjevas process evaluation model [45, 53], process evaluation model by Reelick and colleagues [74], Lichstein’s treatment implementation model [84], and normalization process theory [88].

Several constructs were frequently included within these frameworks. Intervention characteristics, including quality and validity of evidence, were prevalent considerations made prior to implementation [44, 45, 48, 53, 83, 88–90, 98, 100, 102, 103]. All ten frameworks included constructs relating to implementation setting factors, including both internal (e.g., resources) and external (e.g., government policy) to the implementing organization, and the implementation process, including planning, program adoption, implementation execution, and sustainment. Iterative and reflexive monitoring and (re-)evaluating implementation strategies and outcomes were also components of all included frameworks (see Table 2 in Additional file 3 for details).

**Table 2:** Barriers and facilitators to implementation of EBIs for caregivers of people with dementia, mapped onto the Consolidated Framework for Implementation Research constructs

CFIR domains	Barriers to implementation	Facilitators to implementation
eHealth [40–54] *Implemented in and/or delivered by community-based aged care organization (e.g., dementia day care centers), university medical centers (i.e., research teams), and/or ambulatory mental healthcare institutions*		
I. Intervention characteristics	• Appropriateness: Technical issues with intervention components; poor connectivity, unintuitive user experience/interface (e.g., illegible font, no functional real-time chat box with access to facilitator feedback, unnecessary and confusing tools and functions); existing video communication tools insufficient for health education sector • Acceptability: Difficulty level of language used unsuitable for end users, privacy and ethical concerns, intervention rigidity limited tailoring, unsuitable length of intervention (session and program) duration	• Real-time information/alert/notifications and direct instant access to human facilitator (coach) to provide tailored, individualized support; engaging topical forums allows users to share/exchange questions; accessible resources/library • Multimodal delivery of information (e.g., centralized Internet-based platform with information, paired with print-out copies, written in simple language, presented in clear font), video and audio (verbal) guidance/instructions facilitated use • Caregivers (with sufficient digital literacy) appreciate virtual on-demand access and timing flexibility
II. Outer setting	• Participants faced time constraints (due to caregiving obligations), users’ lack of awareness of the program availability and preparedness to participate (e.g., poor technological literacy; inflexible schedule), improper timing of intervention (e.g., too early/too late in PwD care trajectory) • Lack of integration with existing dementia/aged care services (lack of integration support from local government agencies) • Traditional healthcare settings (e.g., hospitals) unable to adopt intervention and can only be implemented as a community-based resource (e.g., FamTechCare) • Poor physical infrastructure in geographical region (internet connectivity), widespread sociocultural resistance to adopt Internet-based interventions	• Logistics: Active dissemination through network events and leveraging network partners’ channels (e.g., locally trusted intermediaries and clinicians’ existing caseload, social networks/social media) • Personal factors: Applying a consumer-directed care model for implementation, high digital literacy rates within target demographic and trust toward implementing agencies (e.g., health-professional-led integrated network model)
III. Inner setting	• Systems unprepared to deliver care to caregivers, caregiver support is administratively filed under PwD care (complicated when PwD is unregistered) • Internal financial cutbacks limit intervention adoption and internal capacity (e.g., staff members lack time needed to review/approve the intervention and learn/train)	• Streamlined administrative processes (e.g., caregiver registered independent from PwD, insurance compensation, integrated online support) • Human resources: Well-prepared/educated staff, engaged leadership • Sustainable financing mechanisms from foundation/government grants • Directly engaging intervention initiators/vendors in implementation (staff training) process
IV. Characteristics of individuals	• Unfit digital literacy in caregivers and staff members • Primary implementation agent (e.g., physicians) does not identify with or recommend the intervention	• Primary implementation agent identifies with program and developers (intervention source) (e.g., internally developed interventions are more familiar and more likely to be recommended)
V. Process	• User recruitment challenges (end-user restrictions, limited reach due to insufficient international search engines, caregivers were not registered with PwD) • Resistance from network members (need for multimodal engagement strategy targeting organizations, clinicians, trainees, and caregiver) • Cost of promotion and sustainment, high user attrition rates • Lack of systematic planning with end users and audit/feedback mechanisms across implementation trajectory	• User recruitment facilitated through partnering with network agency (leveraging partners’ channels), hiring external marketing agencies, creating public awareness/outreach campaigns, and promoting speaking engagements (conferences/seminars/expos) • Using social media marketing strategies to disseminate and strategically target reach and evaluate implementation outcome indicators via site analytics (website traffic, visitor retention) • Iterative changes made to intervention components based on user feedback
Respite care [55–59] *Implemented in and/or delivered by day care centers operated by nursing homes and/or community centers, may be located physically in an existing clinic or repurposing alternative infrastructures (i.e., church)*		
I. Intervention characteristics	• Cost of intervention (attendance fees and unsustainable financing mechanisms) • Intervention programming unsuitable for users (e.g., nutrition plan, lack of dementia-specific accommodation)	• Respite care had a positive atmosphere compared to nursing homes (philosophy surrounding staff training, schedule flexibility and care routine), one-on-one interaction and individualized support sparked position affect and engagement
II. Outer setting	• Lack of transportation to facility; tedious administrative process to apply for respite vouchers (i.e., recurring [re-]application paperwork) • Poor service advertisement: need for “business-like approach in marketing” to attract users • Insufficient financing mechanisms (e.g., respite care vouchers lack comprehensive coverage and are limited by budgets) and high out-of-pocket expenditure for caregivers	• Information sources include home health workers, Alzheimer’s helpline, support groups and legal aid services; health professionals recommend respite care service to patients • Local community integration and participation in activities/events to build awareness and trust • Allocated respite vouchers may subsidize service payments
III. Inner setting	• Staff shortage as a barrier to use • Infrastructure: safety concerns, inadequate space, improper atmosphere and environment (furniture)	• Staff knowledge, qualifications, empathy, and sensitivity were facilitators
IV. Characteristics of individuals	• Perceived misalignment between staff and organizational mission	• Staff individual competency and ability to balance meeting PwD wishes and delivering intervention components (maintaining fidelity), staff assumed multifaceted roles (PwD server and caregiver, liaison with family members)
V. Process	• Poor service advertising and lack of client engagement	• Engagement facilitated by widespread promotion and “business-like” approaches to dissemination
Care coordination and case management [75–80] *Implemented in and/or delivered by primary care practitioners, Veteran Affairs Medical Center (healthcare organization) (USA) and partnering Alzheimer’s Association chapter (community service organization)*		
I. Intervention characteristics	• Underutilized components include health information/education, care planning and coordination, emotional support • Fragmented care continuity and access support reduce intervention use (e.g., caregivers were left to contact community support agencies independently) • Inconsistent quality and accuracy of prescribed information	• Care consultants co-created health plans with dyads, provided tools to enhance dyad competence and self-efficacy, delivered accurate information about local community services, and reduced care fragmentation by connecting dyads to other complementary service agencies • Flexible, tailored (multimodal), manualized care coordination/support improved access and user engagement, scheduling telephone calls ensures caregiver availability and access for rural caregivers
II. Outer setting	• Health system (pathways and information) fragmentation: lack of timely referral pathway and mechanism between GP (i.e., gatekeepers) and intervention agency, GP lack information/awareness • Lack of local hospital system involvement: initiators were viewed as “outsiders” and “competitors” instead of collaborators, GP were not involved as implementation partners	• Inter-agency partnerships between initiators and intermediaries are main facilitators to implementation, embedding interventions into existing services via networks improve sustainment (e.g., PDC), external agencies (licensure and training institute) disseminate innovation • Overarching national agenda (e.g., Older Americans Act) encouraging interventions that streamline service continuity by linking healthcare services to community services, interventions that facilitate GP referral to community services initiated and sustained by the government (e.g., ACAT Australia) • Local intermediary (e.g., Alzheimer’s Association) chapters services regional caregivers and provide cross-system support to GP, academic institutions, and other stakeholders
III. Inner setting	• Resistance for change from local hospital systems (due to physicians’ time restrictions) and lack of financial investment in adopting intervention	• Implementing agency staff training was facilitated through formal education sessions (service-delivery protocol, care coordination information system explanation), additional funding for staff education provided by government grant to support community outreach programs established in partnership with external research centers and academic institutions
IV. Characteristics of individuals	• PwD personal diagnosis avoidance leads to lower diagnostic rates	None identified
V. Process	• Unanticipated challenges (e.g., nursing strike, natural disaster (snow, storm)) and geographic/logistic complications (e.g., transportation limitations) impeded implementation process	• Care consultation was facilitated by using standardized protocols for service delivery, including structured initial assessment, identifying problems/challenges, and developing tailored strategies (care plan) • Recruitment sample was drawn from hospital medical records that indicated dementia diagnosis or memory loss, consulting local community leaders (e.g., clergy members) facilitated fostering networks appropriate implementation planning • Embedding and sustainment were facilitated with support from care coordinators that worked with both the intervention site and partnering agencies/intermediaries
Psychoeducation [60–71] *Implemented in and/or delivered by Veteran Affairs Medical Centers (USA), research/clinical centers, social workers, and community-based outpatient clinics, with integrative support from regional government agencies (e.g., Administration on Community Living (USA))*		
I. Intervention characteristics	• Unsuitable intervention delivery (e.g., long duration of session and length of program; abrupt end of intervention and losing access to resources were an issue) • Courses were not (time) flexible for caregivers, least useful course content surrounded “drug treatments” and “spirituality”	• Useful interventions (e.g., relaxation CDs, educational booklets/videos/courses, group courses) had easy-to-read, multimodal delivery that allowed users access flexibility (time, location) • Intervention adaptation/translation funded by foundation grants
II. Outer setting	• Limited community resources create financing difficulties • Barriers to reach include lack of outreach to community healthcare providers and paid advertisements (resulting in limited awareness of services); intermediaries were not fully and actively involved	• Timing of intervention: caregivers prefer the intervention delivered at the time of dementia diagnosis (or soon after) to be well-prepared/informed • Contracting external agencies: intervention initiators contract local agencies (e.g., Veteran Affairs) and intermediaries (e.g., Alzheimer’s Association) to implement program as part of regular services to scale-up service provision, existing staff members are trained, use existing administrative infrastructure (billing/workload codes) to reimburse services • National/local government agenda mandate public organizations to implement intervention in existing services as regular care, embedded through academic-public partnerships
III. Inner setting	• Staff hesitant to adopt intervention as regular care (increased workload, change in role/function)	• Resources that facilitated implementation include partnering agencies, 24/7 telephone helplines, case managers, resource centers (e.g., Aging & Disabilities Resource Center) • Staff training facilitated by live webinars, consultation calls, and intervention certification programs • Existing staff members were retrained to deliver new intervention as regular care as their skills are suitable to adopt the “highly compatible” and “readily integrated” intervention
IV. Characteristics of individuals	• Staff competencies in identifying caregivers of PwD who are unregistered	• Staff members are comfortable in their role and are able to iteratively modify the delivery of services to accommodate implementation in the community setting
V. Process	• Engagement hindered by ineffective dissemination mechanisms	• Involve local religious/social influencers (“implementation leaders/champions”) to establish validation and credibility and accelerate local buy-in • Intervention sustainment facilitated by iterative adaptations to interventions according to demand (e.g., funding shortage required reconfiguration of STAR-C) and user feedback (e.g., language used, training modality) while maintaining intervention fidelity
Support interventions [102–106] *Implemented in and/or delivered by independently established memory clinics (NL), nursing homes, community centers, and daycare centers*		
I. Intervention characteristics	• Saturated “market” (“surplus”) reduced demand for new interventions serving similar functions (with no clear advantage) and minimized their value • Name of intervention: titles with “dementia” may contain negative association and poor reception	• Support intervention provided at a conveniently accessible location by a small permanent (multidisciplinary) team of professionals, flexible nature of intervention is advantageous compared to institutionalization (which has less capacity, longer wait-lists, increasing care fragmentation) • Clear participant inclusion criteria reduced unexpected challenges by establishing uniform groups
II. Outer setting	• Poor existing health system (e.g., referral pathway, post-diagnostic support, health financing mechanisms) and resource limitations, ineffective reimbursement schemes determined by user attendance (staffing challenges) • Poor relationship between initiator and regional network stakeholders/referrers (GP/welfare organizations), misalignment between partnering organizations • Lack of clarity about structural financing of interventions and sustainability due to national agenda volatility resulting from changes in political parties, fragmented funding (and need for reapplication) impedes implementation process	• Interventions facilitated in proximity to local community centers (church, welfare center) with recognition and support/collaboration (referrals, sustainment) from regional networks, local champions and influencers were more successful • Obtaining financial support from sponsors or care administrations to fund programs, access to multiple sources of financing (reimbursements) and government-initiated incentive schemes (“waiting-list subsidy scheme,” “tailor-made care funds,” and the “informal care subsidy scheme”) or national legislations (or municipality funding) that establish structural funding to claim finances from • Collaboration protocols and formal contracts were facilitators to referrals, placements, execution, and partnership continuation/sustainment
III. Inner setting	• Organizations resist adoption if externally developed interventions are perceived as competition or deemed unsuitable for services provided • Difficulties financing projects implemented in welfare organizations in the interim given inflexible budgets and inflexible organizational structure • Low implementation capacity in adopting organizations: human resource (rigid staff, lack of knowledge about dementia-specific needs, lack of leadership motivation and pioneering spirit, role uncertainty within management team and staff turnover), and financing limitations (no financing for people without formal diagnosis, insufficient finances to compensate contracted staff hours)	• Internal team maintains intensive contact with external partners to facilitate execution • Repurposing existing financing mechanisms within organizations accelerates implementation • Staff training and refresher courses facilitated by an external consultant supported implementation • Motivated leaders who sought out cooperative partnerships and were readily responsive to bottlenecks
IV. Characteristics of individuals	• End-user skepticism causes resistance • Organizations resist existing interventions to “reinvent the wheel”	• Enthusiasm from end users toward the organization and initiators facilitated implementation • Staff competencies (open attitude, motivated) facilitated implementation
V. Process	• Lack of suitable location (high costs, unsuitable atmosphere, inconvenience) and insufficient pre-implementation environmental assessment risked unanticipated challenges • Need for stronger evidence base before network agencies will adopt interventions • Network support (collaboration) gradually decreased over time (e.g., support received during initiation phase decreased in execution phase), and user recruitment became difficult due to lack of publicity • Service capacity was insufficient, and wait-lists became long • Interventions lack promotion (via pharmacies, GP) and public awareness about the components	• Using a stepwise implementation procedure facilitates implementation planning (preparation, execution, continuation), planning by assessing local/regional (demographic) need for intervention and establishing formal agreements with pre-established networks and partners, following (adaptive) project plans • Establishing the intervention in an existing facility with similar practices is more efficient and establishes confidence in users • Enthusiastic initiators/champions within organizations, program coordinator should be professionally up-to-date and possess management experience, hiring external consultant/agencies to facilitate staff training • Engagement and recruitment of users facilitated by partnering agencies/intermediaries (e.g., Alzheimer’s Café), and personalized materials (flyers/newsletters) and media
Exercise [72–74] *Implemented in and/or delivered by professional trainers in sizeable and safe venues, accessible by car and public transportation, or practiced at home with support from video recordings*		
I. Intervention characteristics	• Caregivers unable to independently access intervention (difficult location and scheduling) • Unsuitable information delivery (content was unclear and difficult to understand; content did not fit in the recommended timeframe) • Action plan, coping plan, and clock were not useful components	• Multimodal delivery of clear information (e.g., photos and videos of exercise) facilitated use, support of human facilitator/instructor who provided positive reinforcement/feedback and iteratively adapted intervention components to meet users’ circumstances (e.g., difficulty level of exercise) • Home visits and home-exercise logs supported independent performance
II. Outer setting	• Caregivers did not fill their action plan and found the intervention “too intensive”; limited free time and physical capacity hindered intervention use	None identified
III. Inner setting	None identified	None identified
IV. Characteristics of individuals	• Users lacking sense of self-efficacy in performing exercises at home without guidance (e.g., video instruction material) and perceived skepticism toward intervention efficacy	• Routinization of exercise facilitated sustainment
V. Process	• Recruitment of users through advertisements and personal letters to caregivers were ineffective; barriers included participation burden and lack of caregiver time	• Recruitment was facilitated using an information leaflet (information regarding balance, fall prevention, Tai Chi, and implication of involvement for dyad), key facts sheet, and participant information sheet
Occupational therapy [81–83] *Implemented in and/or delivered by NHS memory services (UK), community mental health services, or in the caregiver’s home (by trainers)*		
I. Intervention characteristics	• Intervention components are not cost-effective within the implementing agency (e.g., agency revenue and therapist salaries are based on patient contact; need to balance training needs and patient contact)	• Individualized face-to-face OT sessions, setting realistic goals with a clear (practical) roadmap, and follow-up check-ins facilitated use • Introducing OT gradually at early stages following dementia diagnosis is optimal and supporting dyads in the adjustment period
II. Outer setting	• Outcomes are influenced by personal factors (e.g., dyad readiness for intervention); introducing OT immediately after diagnosis may confuse caregivers • Lack of health financing and system infrastructure (e.g., patients were mainly referred to OT for other comorbid chronic conditions contributing to functional decline and home safety issues; referral for OT for dementia diagnosis should not be declined)	• Dyads preferred interventions provided by government agencies (local health system) • Using existing health financing infrastructure (e.g., Medicare Part A & B) and adapting intervention to fit reimbursement (“billing”) criteria while maintaining fidelity facilitates implementation
III. Inner setting	• Lack of available resources (e.g., equipment) hindered intervention outcomes • Lack of appropriate, cost-efficient fidelity monitoring mechanisms that fit agency culture	• Organizational readiness facilitates implementation through changing the role and function of existing staff/administrators (transformative agency leadership, training early adopters) • Internal structures should be available (e.g. supervisory structure, training support/referral, client tracking, billing infrastructure)
IV. Characteristics of individuals	• Negative dyad relationship dynamic/quality influences OT outcomes • Dyad’s lack of perceived need for OT and lack of availability to participate	• Caregiver self-efficacy and perceived competency improved with support from OT; positive attitude toward intervention facilitated willingness to try
V. Process	• Engagement hindered due to lack of perceived need for OT • Translation of intervention components was labor intensive	• Engaging long-term staff members, familiar with organizational structure/policy/clients, as early adopters; reflect on training of trainers • Perform in-depth assessment of practice site characteristics and reimbursement requirements; understand mutable and immutable components of intervention and training (for fidelity)
Multicomponent intervention [84–101] *Implemented in and/or delivered by nonprofit community-based organizations focused on services for older adults and caregivers, hospital-sponsored service program for seniors, clinics and nursing facility/assisted living-based program, and Alzheimer’s Association chapter*		
I. Intervention characteristics	• Assessment and monitoring components (e.g., videotaping client behavior) may be intrusive to daily residential use • Complexity of intervention (length of program duration, time consuming) and unsuitable components (reading material difficulty level, font size/color, visuals) deter users • Lack of implementation manual increases training difficulties due to intervention complexity	• Treatment and implementation manuals, time-flexible structured training (with human facilitator), certification, ongoing monitoring and feedback (progress notes, caregiver notebook), cultural/language inclusivity, accounting for polypharmacy in PwD, psychological support (support groups), and caregiver-focused co-created material, delivered through accessible (multi-modal) mechanisms, facilitated implementation • Removing/refining intervention components but maintaining program fidelity and efficacy (e.g., shortening length of sessions or duration of program iteratively)
II. Outer setting	• Caregivers face time constraints, leading to underutilization of interventions, components within intervention unsuitable for users’ conditions • Scaling up is hindered by larger agencies’ complexity (e.g., Veteran Affairs), including size of organization, number of facilities, large catchment areas • Lack of diverse partnerships beyond aging network	• Collaborative agencies within regional network support implementation: leverage strengths of each separate agency, each have a role in implementation (e.g., training, staffing, analyzing outcomes), communication between initiators and community agency facilitated sustainment with local funding agency • Training and scaling-up were facilitated by external private company and academic institutions, embedding intervention as part of regular services within intermediary agencies supported scale-up, partnering with faith-based institutions supported engagement and recruitment • Legal reform in aging policy facilitates caregiver-oriented interventions, extending financial coverage and benefits to caregivers of PwD, interventions with government (state/municipality) recognition and support had more successful continuation and scale-up
III. Inner setting	• Staff were unprepared (lack of dementia care training/competencies, unclear intervention budget plan, staff turnover, large caseloads, difficulties convincing staff of intervention value) • Poor fit between existing services/organizational capacity/culture (including allocating finances/infrastructure/human resources) and intervention components, workload credit and billing codes influenced implementation	• Staff/trainers received clear manual and instructions, refresher sessions were also provided, staff enthusiasm toward intervention facilitates continuation, sufficient time contracted to train staff and deliver intervention components • Administration must buy into the intervention, modifying interventions to fit organization infrastructure/resources and routine facilitates sustainability, good cultural fit between intervention and implementing agency
IV. Characteristics of individuals	• Overburdened caregivers could not participate in interventions • Lack of trust in government-related institutions deterred participation • Trainer turnover hindered program maintenance, existing need for timely certification process	• Staff/counselor competency and personalized approach to care facilitated use • Sense of community ownership and culturally adapted intervention facilitated uptake and continuation
V. Process	• Monitoring fidelity using video cameras is intrusive in naturalistic settings • Sustainment and continuation difficult due to staff turnover and user dropout • RCT results may not reflective of real-world demands and outcomes • Lack of dissemination and user recruitment, localized recruitment limited use and awareness	• Translating intervention to fit wider demographic; co-designing components with advisory committee and users to improve suitability • Exploring and engaging local partners facilitated implementation, dissemination, and scale-up, partnering with cross-sectoral agencies and leveraging individual strengths enhance outcomes, continuous promotion across implementation trajectory

### Barriers and facilitators to implementation (CFIR)

The barriers and facilitators to implementation were mapped based on the domains (and constructs) of the CFIR, including *intervention characteristics*, *outer setting and inner setting of the implementing organization (e.g., nursing home)*, *characteristics of individuals*, and *process of implementation*, which allowed for systematic examination of the contextual variables.

#### Barriers to implementation

*Intervention characteristics* domain presented barriers to implementation, including lack of *relative advantage* (4/67; 6%), poor *adaptability* (12/67; 17.9%), and unsuitable *design quality and packaging* (25/67; 37.3%). New interventions are hindered by high market saturation and are less likely to penetrate organizations due to the presence of similar “usual care” programs [75, 98, 100, 105]. The EBI user’s poor digital literacy hindered use, as did the interventions’ complicated user interface designs, fragmented information, complex language, and unsuitable components that fit poorly with users’ capabilities [40, 47, 53, 54, 75, 98, 100, 105].

The *outer setting* domain presented barriers to implementation, including *patient needs and resources* (*24/67*; *35.8%*), such as implementing agencies’ lack of awareness surrounding influential cultural nuances that deter caregivers from seeking external support (e.g., filial piety) [92, 105], and caregivers’ personal circumstances, including insufficient personal finances, time constraints, poor digital literacy, and adequate information to confidently participate [41, 55, 59, 74, 89, 92, 106]. Additionally, an intervention is less likely to be positively received if introduced to caregivers at an inappropriate stage. For instance, introducing occupational therapy to caregivers immediately following a PwD’s dementia diagnosis creates confusion; alternatively, engaging caregivers in a support program at a later stage in the care trajectory will be less effective since they need communication training and decision-making guidance beginning in early stages [61, 62].

Barriers to implementation under *external policy and incentives* (15/67; 22.4%) include lack of care coordination and continuity within less developed health systems [77, 79, 103, 106], top-down policies that established unsuitable or limiting funding mechanisms to implement and sustain community-based programs [66], and fragmented care financing that requires caregivers to (re)apply for assistance covered under different legislations [83, 94, 102, 103, 105, 106]. *Cosmopolitanism* (14/67; 20.9%) also contained barriers to implementation, including the complexities of vast networks that foster misalignments between partnering agencies and obscure respective actors’ roles and responsibilities [95, 99, 102, 105]. Consequently, poorly networked EBI initiators face distrust with implementing agencies, limited regional partnerships, and impeded service referrals and dissemination [77, 79, 102, 103, 105, 106].

*Inner setting* barriers to implementation are found within implementation agencies (e.g., community nursing homes). Barriers classified under *structural characteristics* (2/67; 3.0%) and internal *network and communications* (2/67; 3%) constructs included rigid hierarchal organization structures, inflexible operating budgets, and lack of role clarity and fragmented information transfers between staff members [102, 105, 106]. *Tension for change* (5/67; 7.5%), *compatibility* (7/67; 10.45%), and *relative priority* (2/67; 2.99%) presented barriers, including staff reluctancy toward adopting externally developed interventions and implementing agency’s lack of capacity for and commitment toward promoting new innovations [68, 95, 103, 105]. *Leadership engagement* (4/67; 6.0%), *available resources* (15/67; 22.4%), and *access to knowledge and information* (5/67; 7.5%) presented barriers, including ambiguity surrounding leadership roles [102], inadequate physical and human resources [55, 78, 100], and the absence of implementation guidance and staff training resources [55, 79, 96].

*Characteristics of individuals*, including caregivers’ and implementors’ *knowledge and beliefs about the intervention* (5/67; 7.46%), also impeded implementation if they are skeptical about the intervention’s privacy and safety [45, 50, 72, 98]. Caregivers’ and implementors’ *self-efficacy* (3/67; 4.48%) and *individual identification with organization* (2/67; 2.99%) impeded implementation if the actors lacked confidence in their roles or if they perceived a misalignment between the organization’s mission and the intervention’s intended outcome [72, 73]. Caregivers’ and implementors’ *other personal attributes* (15/67; 22.39%), such as a deficit in caregivers’ personal capacity (e.g., financial, and physical capacity, digital literacy) to participate in the intervention [73, 74, 82, 84] or staff members’ lack of social and cultural awareness [59, 92, 98], impeded implementation.

The *process of implementation* also presented barriers to implementation. *Planning* (13/67; 19.4%) was hindered by the absence of implementation manuals and fidelity monitoring mechanisms [84, 96], inconsistent and fragmented communication between partnering agencies [43, 78, 103], and poor familiarity with the implementation sites’ contextual nuances [105]. *Engaging* (13/67; 19.4%) was hindered by ineffective recruitment strategies employed exclusively at the local intervention sites and unanticipated difficulties promoting the intervention and gaining caregivers’ and implementation partners’ acceptance due to a fragmented regional network [48, 68, 74, 90, 98, 103]. *Formally appointed implementation leaders* (2/67; 3.0%), *champions* (3/67; 4.5%), and *external change agents* (2/67; 3%) presented fewer barriers to implementation, but the absence of clear leadership, high staff turnover, and fragmented information across partnering agencies created tension that disrupted all stages of implementation [98, 99, 102]. *Executing* (7/67; 10.5%) was hindered by high caregiver attrition rate [52, 96] and unexpected organizational changes and diminished capacity [78, 106]. *Reflecting and evaluating* (3/67; 4.5%) revealed discrepancies between clinical and real-world results, which caused unanticipated implementation barriers that required iterative responses from implementers [95, 98, 106].

#### Facilitators to implementation

*Intervention characteristics* that facilitated implementation include the EBI’s *relative advantage* (10/67; 14.9%), *adaptability* (19/67; 28.4%), *design quality and packaging of intervention components* (42/67; 62.7%), and *cost* (4/67; 6.0%). Advantageous interventions possessed flexible, patient-centered, and culturally adapted programming, and they promoted service continuity through a comprehensive range of integrated services. Adaptable EBIs ensured homogenous participant groups and provided multimodal delivery of intervention components [51, 53, 75, 92, 101, 103]. EBIs were more successfully adopted by end users, if moderated by a human facilitator (e.g., therapist, IT specialist, coach), and by organizations, if implementation is guided by a protocolized implementation guide [42, 43, 46, 51, 52, 61, 66, 68, 71–74, 82, 92, 93, 96, 101]. Interventions with costs covered through sustainable funding sources (e.g., private foundation or government grants) were more likely to survive [59, 67].

*Outer setting* domain contained the most reported facilitators to implementation. *Patient needs and resources* (22/67; 32.8%) included convenient service location equipped with appropriate physical infrastructure and scheduling flexibility [55, 65], sufficient user awareness and preparedness [69, 75, 82], and suitable fit between intervention and users’ levels of digital literacy and needs [40, 42, 43, 52]. *Cosmopolitanism* (29/67; 43.3%) facilitators included establishing and harnessing strong, active local collaborative networks with dedicated implementation and dissemination partners, including intersectoral organizations (i.e., intermediary organizations) with influence spanning across sectors, whose insights and contributions are valuable across all stages of implementation [47, 57, 66, 67, 75, 85–88, 91, 102, 105–107]. *External policy and incentives* (20/67; 19.9%) facilitate implementation through the successful funding and reimbursement of intervention costs, delivered through mechanisms established by existing national legislations [59, 67, 76, 90, 94, 101, 102, 106, 107].

*Inner setting* constructs, including *structural characteristics* (1/67; 1.5%), *network and communications* (3/67; 4.5%), and *culture* (3/67; 4.5%), facilitated implementation through continuous structural financing, regular staff communication and training, and staff enthusiasm about the intervention [90, 98–101, 105]. Facilitators associated with *tension for change* (2/67; 3.0%), *compatibility* (15/67; 22.4%), and *learning culture* (*1/67*; *1.5%*) included the alignment of the intervention’s intended outcome and implementing agency’s mission, the agency’s willingness and administrative capacity to routinize the intervention as part of usual care (e.g., utilizing existing billing/work codes to receive compensation, integrate EBI into clinical workflow), and the modification of existing staff members’ roles to adopt new interventions [46, 68, 69, 90, 91, 95, 98, 100, 106]. Facilitators under *leadership engagement* (7/67; 10.5%) included engaging managers that possessed a clear agenda, a creative mindset, and a proactive approach of continuous improvement [48, 67, 78, 95, 102, 106]. Facilitators under *available resources* (13/67; 19.4%) included motivated, well-trained staff members, accessible and convenient implementation location, and supplemental financial and collaborative support from regional government agencies [43, 48, 55, 59, 67, 98, 100, 105, 106]. *Access to knowledge and information* (11/67; 16.42%) was facilitated by using a cascade model of training, hiring external training agencies, and requiring protocolized licensure and certification for intervention staff to ensure fidelity and program validity [66, 67, 87, 90, 93, 94, 96, 97, 99, 101].

*Characteristics of individuals*, including caregivers’ and implementors’ *knowledge and beliefs about the intervention* (2/67; 3.0%), facilitated implementation if the intervention was developed locally or within the implementing organization [48, 92]. Caregivers’ and implementors’ *self-efficacy* (8/67; 11.9%) and *individual state of change* (2/67; 3.0%) facilitated implementation if they possess competencies required to succeed in their roles and are well-equipped with communication and coping skills [40, 45, 61, 62, 67, 81, 95, 98]. *Individual identification with organization* (3/67; 4.48%) facilitated implementation if the implementation agents identified with the intervention initiators and were enthusiastic about its success [48, 67, 90]. *Other personal attributes* (10/67; 14.9%), such as staff members’ ability to adapt and cater to caregivers’ iterative needs (e.g., bilingual and technical competencies) and caregivers’ positive attitudes toward participation, also facilitated implementation [40, 57, 66, 82, 89, 90, 92, 98, 102].

The *process of implementation* was also facilitated by unique contextual factors. *Planning* (13/67; 19.4%) was facilitated by adapting and translating interventions to fit local implementation setting and co-creating implementation and marketing plans that considered influential contextual nuances [57, 78, 83, 84, 88, 96, 99, 100, 102, 105, 106]. *Engaging* (21/67; 31.3%) facilitators included the active dissemination of intervention information, by applying marketing strategies to reach specific audiences and disseminating recruitment materials through partners’ networks [40, 47, 51, 53, 57, 66, 72, 76, 78, 87, 90, 92, 94, 99, 100, 102, 103, 105, 106] and the engagement of caregivers through referrals from general practitioners and members of local care organizations [51, 75, 80, 98, 99]. Additionally, *opinion leaders* (2/67; 3.0%), *formally appointed internal implementation leaders* (8/67; 11.9%), *champions* (7/67; 10.5%), and *external change agents* (11/67; 16.4%) facilitated implementation by engaging local influential religious leaders to support normalizing the use of new interventions [78, 92], by leveraging individual strengths from external agencies to establish a multidisciplinary advisory team [47, 87, 98, 99, 106], and by appointing a leader to guide implementation and sustainment [58, 75, 76, 78, 102, 103, 105, 106]. For example, faith-based organizations may influence public perception and approval of interventions; academic partners support recruitment and registration of new participants [92], and intermediary organizations (e.g., Alzheimer’s Association) inform regional partners and support in facilitating knowledge transfer. *Executing* (14/67; 20.9%) and *reflecting and evaluating* (8/67; 11.9%) facilitated implementation through regular monitoring and evaluation, securing partnerships through formal agreements (e.g., Memorandum of understanding), and iteratively adapting operational processes to meet real-world demands and unanticipated complications. Table 2, found below, and Tables 3 and 4 in Additional file 3, provide further details found surrounding barriers and facilitators to implementation.

**Table 3:** Implementation strategies and mechanisms reported

Type of intervention	Most frequently employed discrete strategies (cluster/strategy)	Example of mechanism reported
Multi-component [84–101]	Cluster 5/ERIC 43 (Make training dynamic)	• Caregiver notebook included educational materials, interactive modules, and worksheets that corresponded with original intervention, but computerized telephone system was also sued to deliver information [86]
	Cluster 5/ERIC 71 (Use train-the-trainer strategies)	• External agency (DAZ) built to train adopting agencies in the intervention components, to scope local partners and needs, and to select professional project leaders [87] • Trainers were instructed to apply a person-centered approach and individualized activities to the PwD and caregiver [93]
	Cluster 2/ERIC 33 (Facilitation)	• Interventionist provides individualized problem-solving skills based on problems identified using the caregiver notebook [95] • Counselor creates safe and comfortable environment to enable dyads to discuss and plan at their own pace [97]
	Cluster 4/ERIC 52 (Promote network weaving)	• Caregivers were recruited by partner agencies (flyers, public service announcements, community outreach, email, website programming) [99] • Partnership with Area Agency on Ageing to translate intervention into nonprofit integrated health system [100]
eHealth [40–54]	Cluster 3/ERIC 51 (Promote adaptability)	• Digitalizing existing forms (e.g., Healthy Aging Brain Care Monitor) to collect and centralize patient information [46] • Website was provided alongside a toll-free telephone service to enhance access to intervention [47]
	Cluster 5/ERIC 31 (Distribute educational materials)	• Intervention consisted of multimedia e-learning lessons, resources, weekly educational emails, monthly livestream events [48] • Internet platform contains information for caregivers on dementia and intervention costs/privacy/registration process [52]
	Cluster 5/ERIC 29 (Develop educational materials)	• iSupport intervention, developed by the World Health Organization, provided online self-help and caregiver skills training [42, 43] • Spanish-language content for caregivers was developed by translators [51]
Psychoeducation [60–71]	Cluster 5/ERIC 19 (Conduct ongoing training)	• START provides 8-week, manualized training for caregivers of PwD [61], and Tele-Savvy reformatted the in-person Savvy Caregiver curriculum into a [digital] 7-week program [64]
	Cluster 5/ERIC 29 (Develop educational materials)	• REACH VA materials (photographs) were locally modified to reflect diversity [67] • Medway Carers Course was developed by specialist psychologists responding to clinical need for care focused on PwD and relatives [69]
	Cluster 5/ERIC 43 (Making training dynamic)	• Training was facilitated through treatment manual, role-playing, structured practice with behavioral problem-solving plans using videos [68] • Workshop included training on the resource book, role-playing, and group discussions of various situations [66]
	Cluster 5/ERIC 31 (Distribute educational materials)	• Resource notebook was provided by counselors [66]; information was distributed verbally or written on printed handouts [69]
Care coordination and case management [75–80]	Cluster 4/ERIC 52 (Promote network weaving)	• Partnership added care consultation from Alzheimer’s Association (intermediary) to usual care offered to members of Kaiser Permanente (hospital) [75] • Establishing formal partnership between VA medical center and Alzheimer’s association chapters [76]
	Cluster 4/ERIC 24 (Develop academic partnerships)	• COEP was conducted in collaboration with the Michigan Alzheimer’s Disease Research Center at the University of Michigan in Ann Arbor [78] • Informal caregivers were recruited with support from University of Lincoln [79]
	Cluster 6/ERIC 59 (Revise professional roles)	• Staff from local Dementia and Specialist Older Adult Mental Health Services were sought to deliver intervention [79] • Care consultation delivered by Alzheimer’s Association staff members who are master’s prepared social workers [75]
	Cluster 6/ERIC 30 (Develop resource-sharing agreements)	• Care coordinators from different organizations worked as a team, supported by a shared electronic Care Coordination Information System [76]
Support interventions [102–106]	Cluster 4/ERIC 35 (Identify and prepare champions)	• Planning implementation by selecting an easily accessible location with a small and permanent team of professionals [105] • Nursing home-based PwD day care centers made transition to community day care with caregiver support according to Meeting Centres Support Program [102]
	Cluster 4/ERIC 6 (Build a coalition)	• Group consisted of manager of day care center, transition supervisor from academic university, and researcher and consultant with experience delivering intervention in real-world settings [102] • Involve network of care and welfare referrers [106]
	Cluster 4/ERIC 47 (Obtain formal commitments)	• Initiative group, project group, and all relevant collaborating organizations signed cooperation agreement [102] • Community engagement and collaboration with existing local care and welfare organizations [105]
	Cluster 4/ERIC 52 (Promote network weaving)	• Collaborating across sectors and between health and social organizations; cooperating organizations include local Alzheimer’s Associations, mental health organizations, general practitioners, home care organizations, case managers, and local caregiver support organizations [102]
Respite care [55–59]	Cluster 6/ERIC 59 (Revise professional roles)	• Staff members assumed multifaceted care rolls (e.g., serving meals, collaborating with family members, providing intensive ADL) [57] • Staff members act as research liaisons and provide feedback for program evaluation [58]
Exercise [72–74]	Cluster 2/ERIC 33 (Facilitation)	• Classes were led by fully trained Tai Chi instructors who provided home-based support and real-time feedback during classes to correct the participant’s poses and movements [72]
	Cluster 5/ERIC 31 (Distribute educational materials)	• Booklets with exercise instructions (with explanatory photos and text) were distributed [72, 73]
	Cluster 5/ERIC 19 (Conduct ongoing training)	• Exercise training for caregivers ran over 4 weeks [72] to gradually become familiar with exercise movements through individual coaching [74]
	Cluster 9/ERIC 12 (Change record systems)	• Action plans and coping plans were developed for caregivers to record their exercise progress [72, 73]
Occupational therapy [81–83]	Cluster 1/ERIC 4 (Assess for readiness)	• Meaningful activities are identified through narrative interviews [81, 82] • Structured observation of activities [82]
	Cluster 1/ERIC 18 (Conduct local needs assessment)	• Evaluate local needs through home visits and monitoring activity outcome [82, 83]
	Cluster 3/ERIC 63 (Tailor strategies)	• Adapt intervention to fit the physical and social environment, apply caregiver management approaches (including prioritizing caregiver concerns), and be considerate of PwD functionality [83] • Personal goal setting based on assessment findings [82]
	Cluster 3/ERIC 51 (Promote adaptability)	

### Implementation and dissemination strategies (ERIC taxonomy)

Of the 67 included studies, 61 studies reported details on the implementation strategies employed to support the delivery of the chosen EBI for caregivers of PwD. Sixty-eight of the 73 ERIC taxonomy’s discrete strategies, across all nine clusters, were identified (see Table 5 in Additional file 3 for details); six discrete strategies (ERIC 45, 50, 68, 3, 28, 10) were not reported by any included study. Multicomponent interventions employed the widest range of discrete strategies (58/73; 79.5%), followed by psychoeducation interventions (48/73; 65.8%), and care coordination and case management (40/73; 54.8%). The most frequently identified discrete strategies were found in the “Train and educate stakeholders” cluster. Mechanisms found within this cluster included training through multimodal delivery, including delivering education and information through an Internet platform equipped with real-time feedback from trainers via a toll-free telephone line [40, 47, 53, 73, 88, 91, 95, 98]. The “Provide interactive assistance” cluster also contained frequently employed discrete strategies; mechanisms identified included providing tailored, individualized feedback to end users [54, 66, 80], facilitating flexible scheduling for end users [57, 65, 72, 80, 98], and enhancing the connectivity and reflexivity between referrers and services [47, 66, 67, 75, 76, 87, 88]. Further implementation strategies and mechanisms are included in Table 3 found below, and more detailed mechanisms and actions can be found in Table 6 of Additional file 3.

Several discrete strategies within the same cluster were also frequently employed together. In the “Develop stakeholder interrelationship” cluster, “Build a coalition” and “Obtain formal commitments” (9/67; 13.4%) were employed together across six studies [66, 78, 85, 100, 102, 105]. In the “Train and educate stakeholders” cluster, “Develop educational materials” (27/67; 40.3%), “Make training dynamic” (34/67; 50.7%), and “Distribute educational materials” (31/67; 46.3%) were employed together in 15 studies [47, 48, 51, 52, 58, 63, 64, 68, 69, 80, 84, 86, 88, 93, 95]. In the “Adapt and tailor to context” cluster, “Tailor strategies” (26/67; 38.8%) and “Promote adaptability” (27/67; 40.3%) were employed together in 18 studies [40, 43, 47, 51, 53, 61, 67, 72, 74, 80, 82, 84, 85, 90, 91, 93, 100, 104].

Eighteen of 67 studies [58, 67, 74, 83–86, 88, 91, 95, 98–103, 105, 106] conducted initial assessments of contextual determinants and, based on these, adapted the implementation strategies to target the barriers and improve the translation of the EBI into local practice. Adaptations made to enhance feasibility due to local constraints (i.e. available financial resources, compliance with local insurance reimbursement regulations) include reducing the frequency of intervention delivery [74, 83, 85, 98] and adapting the professional profile of the EBI provider to fit the available local human resources [91, 99, 101, 102]. Other challenges included the need to adapt the language used to suit users’ capabilities [84, 101] and the location, medium, and format used to deliver the EBI [85, 100, 105]. However, none of the studies was explicit about the mechanism of each adaptation nor did they report a formal evaluation of the impact the adaptation had on the effect of the selected strategies on implementation outcomes, which may indicate a lower degree of maturity of implementation science application in this area.

### Implementation outcomes (Implementation Outcomes Framework)

The IOF presents an implementation outcome taxonomy, including *acceptability*, *adoption*, *appropriateness*, *costs*, *feasibility*, *fidelity*, *penetration*, and *sustainability* [26]. *Appropriateness* (49/67; 73.1%) was reported as the intervention’s “suitability,” “usability,” and “helpfulness” for users, and it is “fit into existing workflow” within implementation agencies [48]; evaluative indicators included respondents’ rating of perceived “helpfulness” and their “intention to use.” *Acceptability* (55/67; 82.1%) was reported as the end users’ and implementing agencies’ “satisfaction” with intervention effectiveness and components, including delivery modality, timing of intervention, duration of program, and quality of interventionist [44, 45, 49].

*Penetration* (52/67; 77.6%) was only reported in relation to the wider implementation setting; studies mainly descriptively reported *how* users were recruited, including marketing strategies, and leveraging financial resources and interpersonal relationships from cross-sector partners [47, 51, 63, 68, 70, 75, 77, 82, 86, 87, 92]. *Sustainability* (40/67; 59.7%) was described as users’ and organizations’ “demand for program continuation” and “routinization of care.” Studies mainly focused on describing the existing internal and external financing mechanisms and the role of collaborators and external agencies in training and scaling up [44, 59, 66, 76, 83, 86, 87, 100, 103].

*Implementation fidelity* (14/67; 20.9%) was characterized as the facilitators’ degree of “adherence” to the implementation protocol and was explicitly reported through fidelity enhancing, measuring, and monitoring mechanisms. Implementation fidelity enhancing strategies included protocolizing implementation [58, 63, 93, 97], training certification programs with initiators [58, 63, 68, 88, 90, 93, 97–100], and using fidelity checklists and guiding scripts [68, 95, 99]. Fidelity measuring and monitoring strategies included the use of delivery assessment forms and checklists [58, 83, 88, 99] and ongoing coaching and consultation with initiators [58, 65, 68, 88, 97–99].

*Adoption* (18/67; 26.9%) was reported as *how* administrations are motivated to “buy into” the intervention and *how* the engagement of local “influencers” promotes user uptake [92, 95, 101, 105]. *Feasibility* (18/67; 26.9%) was reported as the degree to which intervention components fit within the organization; for instance, components tested in the RCTs (e.g., fidelity monitoring mechanisms [i.e., surveillance records]) were not pragmatic, or practices could not be easily streamlined into existing workflow [54, 84]. *Implementation cost* (9/67; 13.4%) was mainly reported as *how* operational and staffing costs were covered, mainly though government-regulated financing programs (e.g., Medicare, Social Support Act, Older Americans Act) [58, 59, 67, 76, 83, 87]. Implementation outcome details can be found in Table 7 of Additional file 3.

Studies did not evaluate the relationship between implementation strategies and implementation outcomes, but several descriptive trends were identified across included studies. Facilitation (ERIC 33) was employed in 23 of 55 studies that reported on acceptability. Using train-the-trainer strategies (ERIC 71) influenced implementation fidelity in 11 of the 14 studies that reported on fidelity and 23 of 40 studies that reported on sustainability. Mass media (ERIC 69) were employed in all studies that reported on penetration (see Table 8 of Additional file 3 for details).

## Discussion

To our knowledge, this is the first review to be guided by three unique implementation science frameworks to study barriers and facilitators to implementation, implementation strategies, and implementation outcomes found in literature relating to EBIs for informal caregivers of PwD.

Applying multiple frameworks allows researchers to examine the various components across implementation processes to potentially establish links between contextual determinants, implementation strategies, and implementation outcomes [108]. Through this methodological approach, our findings illuminate the achievements and gaps in theory-informed implementation thinking in modern dementia care, and they highlight contextual factors that influence successful implementation of EBIs of importance to informal caregivers of PwD.

The MMAT rating results indicated that included studies were of high quality overall, but the appraisal criteria did not assess the quality of implementation reporting nor the rigor of evaluative implementation research designs, suggesting that more suitable appraisal tools are essential to ensure high-quality implementation research [109]. Only 21 out of 67 included studies were guided by an implementation science framework, indicating a need to reinforce the application of implementation science in dementia care research. Furthermore, this review also found that the mean importance and feasibility ratings for discrete strategies, as determined by Waltz and colleagues [24], did not reflect the frequency of implementation strategies used in the real-world implementation of EBIs in home- and community-based services (HCBS). For example, the discrete strategy “use mass media,” employed by 12 of 67 studies, and “use train-the-trainer strategies,” employed by 26 of 67 studies, were both labeled in the original study as low feasibility and low importance, revealing the potential lack of suitability and relevance of existing ratings in HCBS contexts. These results call for an extension of the ERIC taxonomy, or the development of an entirely new framework, with insights from real-world community practitioners with implementation experience, as proposed by Balis and associates [110].

Included studies were also not explicit about implementation strategy mechanisms and did not evaluate implementation strategy effectiveness, nor the degree of influence on implementation outcomes, potentially due to shortage of funding for types II and III implementation-effectiveness hybrid study design prior to 2020 [111, 112]. Only one study in this review reported the rationale for the use of an implementation-effectiveness hybrid design [88] — overall, a direct link (statistical or otherwise) between the implementation strategy selected and implementation outcomes assessed could not be established or evaluated formally in this review. Furthermore, 18 included studies seemed to have adapted their implementation strategies to target barriers and enhance the translation of EBIs to fit their context, but these studies did not directly evaluate the degree of alignment between the barriers and adapted strategies, nor did they propose evaluative methods, which may suggest low maturity of implementation science application in dementia care research.

Similar to the challenges mentioned by Lengnick-Hall and colleagues [113], implementation outcomes were also inconsistently reported, and authors were not explicit about the level of analysis (i.e., individual or organizational level). Delineation is critical to determine casual mechanisms and evaluate implementation strategy effectiveness, particularly when reporting fidelity as an outcome, as authors often referred to both end-user adherence to intervention protocol and facilitator adherence to implementation protocol. The Outcomes Addendum to the CFIR can be used to support researchers in delineating the level of measurement to improve the reporting and synthesizing of contextual determinants [114].

Relating to the barriers and facilitators to implementation, the modifiable *intervention characteristics*, primarily *design quality and packaging*, should be strategically and iteratively adapted through feedback from end users to fit the implementation context. In accordance with Lundmark and colleagues [115], this review concluded that consideration of *inner* and *outer setting* determinants is also central to ensure alignment between the intervention, the implementing agency’s mission and structural capacity, and sociocultural needs and preferences in the local community [51, 53, 75, 92, 101, 103]. In the *outer setting* domain, *cosmopolitanism* included the relationship dynamics between the implementing agency, cross-sector stakeholders, and researchers in academic institutions (e.g., community-academic partnerships [116] and public–private partnerships [83]). The findings suggest for the description of *cosmopolitanism* to distinguish between multi-level, cross-sector partnerships to focus resources and expertise more effectively, which aligns with the recommendation of Proctor and colleagues [117] to leverage the individual strengths of each partner and co-develop toolkits to facilitate evidence dissemination and EBI implementation. These complex networks facilitate multiple stages of implementation, but further implementation research supported by experiential knowledge from implementation support practitioners is required to systematically examine processes of collaboration, including each partner’s role in knowledge translation, knowledge brokering, and EBI sustainment and scale-up, to advance implementation theory [118–120].

### Recent developments

To ensure the relevance of the results, an updated search was conducted in August 2023 using the original search terms. Only ten of the 1186 results published after March 2021 fitted the inclusion criteria, and these studies primarily focused on the early-stage adaptation and implementation of three EBIs, iSupport [121–126], Reducing Disability in Alzheimer’s Disease (RDAD) program [127, 128], and STrAtegies for RelaTives (START) [129, 130], which have been previously included in the results (see Table 1). The new articles indicated progress in enhancing real-world applicability but did not yield any new barriers or facilitators (as summarized in Table 2). Implementation and adaptation processes were guided by the i-PARIHS framework [129], ecological validity framework [123], WHO iSupport Adaptation and Implementation Guidelines [121, 122, 124–126], and EBI adaptation guide by Escoffery and colleagues [128, 131]. Trends in recent publications suggest that implementation science in dementia care research is slowly progressing, mainly with implementation and adaptation guidance from the World Health Organization and through international collaboration. Overall, there has been little significant progress made in recent years, and the results from this review remain representative of current literature.

### Limitations

This review has several limitations. First, the synthesized results did not include studies published after March 2021, which may have excluded implementation details from recent publications. Next, the ERIC taxonomy has limitations since it was developed exclusively through insights from hospital-based clinicians, and implementation strategies employed at the community setting may not be clearly presented in the taxonomy, which potentially limited the reviewer’s ability to optimally extract and match reported strategies from the literature. The review proposes a call to action for the implementation science community to systematically develop a new taxonomy more appropriate for use in the community setting. Additionally, since the search strategy was also developed with guidance from existing implementation science research largely conducted outside of the community setting, more suitable terminology may have been missed, which may exclude relevant articles. Next, although the validity of ASReview tool has been studied [39], there is currently no evidence-based terminal point for article screening by the second reviewer using ASReview, potentially (although unlikely) excluding relevant records. Lastly, due to the poor utilization of suitable implementation reporting guidelines by included studies, the review results were unable to present clear connections between implementation determinants, strategies, and outcomes.

### Future directions and recommendations

The main findings from this scoping review indicate a growing demand for systematic implementation and dissemination of EBI for caregivers of PwD. Further research to develop implementation frameworks that systematically guide implementation processes and address contextual barriers involved in community-based implementation of non-pharmacological EBI is needed. For example, the Community-Academic Aging Research Network’s pipeline for dissemination [116] provides a framework, inclusive of community, academic, and intermediary stakeholder perspectives, to create a contextually suitable implementation plan and to leverage cross-sectoral partnerships that facilitate EBI implementation and continuation.

Future research in this area would benefit from employing more rigorous evaluative methodology, and future reviews may perform meta-analyses to further evaluate the impact of implementation strategies on implementation outcomes. Lastly, scoping reviews focused on implementation literature often report limitations due to heterogenous implementation reporting [132, 133]. Therefore, promoting the use of standardized implementation reporting guidelines (e.g., STaRI [134]) in future studies will enable reviewers produce more clear, consistent, and reliable results.

## Conclusion

The novel combination of three implementation frameworks in the context of evidenced interventions to support informal caregivers of PwD has offered a first analysis of the implementation strategies and mechanisms applied to actualize implementation and the multi-level implementation barriers and facilitators that directly impact implementation success (or otherwise) of these interventions. This review provides a systematic overview that can be used as a foundation to inform and guide implementation researchers to structurally examine outer setting facilitators and implementation strategies, at multiple levels and across sectors, and can guide implementation agents to strategically leverage existing resources and regional networks to streamline local implementation. Mapping local evidence ecosystems will facilitate more structured implementation planning and support for HCBS interventions, and new evidence will also contribute to strengthening implementation science theory and application in dementia care.

### Supplementary Information

**Additional file 1.** Method Overview **Figure 1.** Preferred Reporting Items for Systematic reviews and Meta-Analyses extension for Scoping Reviews (PRISMA-ScR) Checklist. **Figure 2.** Method Flow Chart.

**Additional file 2: Table 1.** Search strategy.

**Additional file 3.** Results overview (detailed) **Table 1.** Results overview. **Table 2.** Overview of constructs found in included studies’ frameworks. **Table 3.** Barriers to Implementation. **Table 4.** Facilitators to Implementation. **Table 5.** Implementation strategies identified across studies (n=67) based on the ERIC compilation. **Table 6.** Implementation actions and corresponding strategies employed in included studies according to Waltz’s implementation clusters and ERIC taxonomy strategies. **Table 7.** Implementation Outcomes. **Table 8.** Common trends identified between implementation strategies and implementation outcomes. 

## Data Availability

All data generated or analyzed during this study are included in this published article and its supplementary information files.
