# Supplementary material for: Implementation and dissemination of home- and community-based interventions for informal caregivers of people living with dementia: a systematic scoping review
Source: Implement Sci. 2023 Nov 8;18:60. doi: 10.1186/s13012-023-01314-y (PMC10631024; doi:10.1186/s13012-023-01314-y)
Supplement: Supplementary file 1 — Additional file 1. Method Overview Figure 1. Preferred Reporting Items for Systematic reviews and Meta-Analyses extension for Scoping Reviews (PRISMA-ScR) Checklist. Figure 2. Method Flow Chart. [file 13012_2023_1314_MOESM1_ESM.pdf]

Additional file 1. Method Overview

Figure 1. Preferred Reporting Items for Systematic reviews and Meta-Analyses extension for Scoping Reviews (PRISMA-ScR) Checklist

SECTION	ITEM	PRISMA-ScR CHECKLIST ITEM	REPORTED ON PAGE #
**TITLE**			
Title	1	Identify the report as a scoping review.	1
**ABSTRACT**			
Structured summary	2	Provide a structured summary that includes (as applicable): background, objectives, eligibility criteria, sources of evidence, charting methods, results, and conclusions that relate to the review questions and objectives.	2-3
**INTRODUCTION**			
Rationale	3	Describe the rationale for the review in the context of what is already known. Explain why the review questions/objectives lend themselves to a scoping review approach.	4-7
Objectives	4	Provide an explicit statement of the questions and objectives being addressed with reference to their key elements (e.g., population or participants, concepts, and context) or other relevant key elements used to conceptualize the review questions and/or objectives.	7
**METHODS**			
Protocol and registration	5	Indicate whether a review protocol exists; state if and where it can be accessed (e.g., a Web address); and if available, provide registration information, including the registration number.	7
Eligibility criteria	6	Specify characteristics of the sources of evidence used as eligibility criteria (e.g., years considered, language, and publication status), and provide a rationale.	7-8
Information sources*	7	Describe all information sources in the search (e.g., databases with dates of coverage and contact with authors to identify additional sources), as well as the date the most recent search was executed.	8
Search	8	Present the full electronic search strategy for at least 1 database, including any limits used, such that it could be repeated.	8
Selection of sources of evidence†	9	State the process for selecting sources of evidence (i.e., screening and eligibility) included in the scoping review.	8-9
Data charting process‡	10	Describe the methods of charting data from the included sources of evidence (e.g., calibrated forms or forms that have been tested by the team before their use, and whether data charting was done independently or in duplicate) and any processes for obtaining and confirming data from investigators.	9
Data items	11	List and define all variables for which data were sought and any assumptions and simplifications made.	9
Critical appraisal of individual sources of evidence§	12	If done, provide a rationale for conducting a critical appraisal of included sources of evidence; describe the methods used and how this information was used in any data synthesis (if appropriate).	n/a
Synthesis of results	13	Describe the methods of handling and summarizing the data that were charted.	9
**RESULTS**			
Selection of sources of evidence	14	Give numbers of sources of evidence screened, assessed for eligibility, and included in the review, with reasons for exclusions at each stage, ideally using a flow diagram.	10
Characteristics of sources of evidence	15	For each source of evidence, present characteristics for which data were charted and provide the citations.	10
Critical appraisal within sources of evidence	16	If done, present data on critical appraisal of included sources of evidence (see item 12).	n/a
Results of individual sources of evidence	17	For each included source of evidence, present the relevant data that were charted that relate to the review questions and objectives.	10 - 19
Synthesis of results	18	Summarize and/or present the charting results as they relate to the review questions and objectives.	10 - 19
**DISCUSSION**			

SECTION	ITEM	PRISMA-ScR CHECKLIST ITEM	REPORTED ON PAGE #
Summary of evidence	19	Summarize the main results (including an overview of concepts, themes, and types of evidence available), link to the review questions and objectives, and consider the relevance to key groups.	19 - 21
Limitations	20	Discuss the limitations of the scoping review process.	21
Conclusions	21	Provide a general interpretation of the results with respect to the review questions and objectives, as well as potential implications and/or next steps.	22
**FUNDING**			
Funding	22	Describe sources of funding for the included sources of evidence, as well as sources of funding for the scoping review. Describe the role of the funders of the scoping review.	23

JBI = Joanna Briggs Institute; PRISMA-ScR = Preferred Reporting Items for Systematic reviews and Meta-Analyses extension for Scoping Reviews.
* Where *sources of evidence* (see second footnote) are compiled from, such as bibliographic databases, social media platforms, and Web sites.
† A more inclusive/heterogeneous term used to account for the different types of evidence or data sources (e.g., quantitative and/or qualitative research, expert opinion, and policy documents) that may be eligible in a scoping review as opposed to only studies. This is not to be confused with *information sources* (see first footnote).
‡ The frameworks by Arksey and O'Malley (6) and Levac and colleagues (7) and the JBI guidance (4, 5) refer to the process of data extraction in a scoping review as data charting.
§ The process of systematically examining research evidence to assess its validity, results, and relevance before using it to inform a decision. This term is used for items 12 and 19 instead of "risk of bias" (which is more applicable to systematic reviews of interventions) to include and acknowledge the various sources of evidence that may be used in a scoping review (e.g., quantitative and/or qualitative research, expert opinion, and policy document).

From: Tricco AC, Lillie E, Zarin W, O'Brien KK, Colquhoun H, Levac D, et al. PRISMA Extension for Scoping Reviews (PRISMAScR): Checklist and Explanation. Ann Intern Med. 2018;169:467–473. doi: 10.7326/M18-0850.

Figure 2. Method Flow Chart

Literature screening and inclusion/exclusion process

Stage 1: Develop literature search strategy and conduct search across databases (Embase, Medline (Ovid), Web of Science and Cochrane Central Register of Controlled trials (Wiley))

Stage 2: Import title and abstracts for all unique search results into ASReview software.

Stage 3: Two reviewers included or excluded (i.e. screened) the imported titles and abstracts of studies based on pre-determined selection

Stage 3.1: Reviewer 1 manually screened the titles and abstracts of all imported studies.

Stage 3.2: Reviewer 2 manually screened the titles and abstracts of studies that were excluded by Reviewer 1 (to avoid false negatives). **Screening by Reviewer 2 was completed once 50 successive studies were excluded.**

Stage 4: Full-text of all 'included' studies were obtained; Reviewer 1 and 2 both assessed the full-text of each individual study to confirm each fit the inclusion criteria. Any conflicts at this stage were resolved by a third reviewer.

Stage 5: Reviewer 1 screened all titles from the reference lists of all final included studies to avoid fragmented literature. Following, abstracts of potential inclusions were screened, and, if study was accepted at abstract screening stage, full-texts were obtained for further assessment by both Reviewers 1 and 2. Final agreed-upon included studies were collected for data extraction.

Data extraction process

Stage 6: Reviewer 1 conducted raw data extraction, guided by a) ERIC taxonomy, b) IOF, and c) CFIR, for 10 pilot studies. Justification and reasoning for extractions was shared and discussed with entire review team until consensus was reached.

Stage 7: Reviewer 1 extracted raw data (for implementation strategies, implementation outcomes, and barriers and facilitators to implementation) based on TMFs for all included studies (collated in Microsoft Excel). Studies were grouped based on the type of intervention (e.g., psychoeducation, respite care, eHealth).

Stage 8(a): Implementation strategies from each study were a) extracted based on the reported action/mechanisms, b) matched to the corresponding ERIC implementation strategies (based on the taxonomy description) and c) classified further into each respective clusters.

Final results reveal the prevalence of each implementation strategy/cluster employed across all studies.

Stage 8 (b): Extraction of the barriers and facilitators to implementation were guided by CFIR. Determinants were categorized based on corresponding domain and (sub)construct.

Following, all extracted and categorized barriers were compiled to compare findings across studies and identify both similar and unique themes within each construct. This process was repeated to examine the facilitators.

Final results reveal the prevalence of each construct and the overarching determinants that impede/facilitate intervention implementation.

Stage 8 (c): Reported implementation outcomes from each study were extracted and matched to each respective the IOF indicator.

Final results reveal the prevalence of each implementation outcome across implementation-focused literature to determine the priorities and gaps in existing implementation research.
