# Supplementary material for: Implementation and dissemination of home- and community-based interventions for informal caregivers of people living with dementia: a systematic scoping review
Source: Implement Sci. 2023 Nov 8;18:60. doi: 10.1186/s13012-023-01314-y (PMC10631024; doi:10.1186/s13012-023-01314-y)
Supplement: Supplementary file 2 — Additional file 2: Table 1. Search strategy. [file 13012_2023_1314_MOESM2_ESM.docx]

**Table 1. Search strategy**

| **Embase.com**  ('dementia'/exp OR 'mild cognitive impairment'/de OR (dementia* OR Alzheimer* OR Mild-cognitive-impairment*):ab,ti,kw) **AND** ((((caregiv* OR care-giv* OR carer* OR in-home-care* OR care-provider*) NEAR/3 (spous* OR famil* OR child* OR parent* OR informal* OR program* OR elder*)) OR dementia*-carer*):ab,ti,kw) **AND** ('implementation science'/de OR (implement* OR adopt* OR uptake OR Appl* OR Carry-out OR Perform* OR Usage OR Practice OR Enactment OR Fulfil* OR knowledge-transfer* OR feasib* OR adapt* OR accept* OR appropriate* OR cost* OR fidelit* OR sustainab* OR penetrat* OR reach* OR utili* OR sustained-integration* OR intention-to-chang* OR embed* OR normali* OR Disseminat* OR diffus* OR (translat* NEAR/3 knowledge*) OR scale-up OR scaling OR barrier* OR change-in-practice* OR Obstruct* OR Obstacle* OR Facilitat* OR Threat* OR access* OR retention OR retain* OR compatib* OR brakes OR levers OR (perception* NEAR/3 attitude*)):ab,ti,kw) **AND** ('intervention study'/de OR 'training'/de OR 'health program'/exp OR 'education'/de OR 'learning'/de OR (intervent* OR training* OR educat* OR coach* OR learn* OR platform* OR innovation* OR program* OR session* OR technolog* OR audit OR reminder* OR ((web OR mobile*) NEAR/3 (application*)) OR trainer* OR coach* OR psychoeducation* OR audit* OR feedback* OR psychotherap* OR (occupation* NEAR/3 therap*) OR respite* OR day-care* OR evidence-based-practice* OR (modif* NEAR/3 environment*) OR information-management-system* OR consultation* OR local-champion* OR local-leader* OR local-network* OR organi*ational-change* OR cognit*-behav*-therap*):ab,ti,kw) *NOT ([conference abstract]/lim OR [editorial]/lim OR [letter]/lim OR [note]/lim OR [Review]/lim OR (review):ti) NOT (acetylcholinesterase* OR donepezil* OR huntington* OR Parkinson):ti* |
| --- |
| **Medline (Ovid)**  (exp Dementia/ OR Cognitive Dysfunction/ OR (dementia* OR Alzheimer* OR Mild-cognitive-impairment*).ab,ti,kf.) **AND** ((((caregiv* OR care-giv* OR carer* OR in-home-care* OR care-provider*) ADJ3 (spous* OR famil* OR child* OR parent* OR informal* OR program* OR elder*)) OR dementia*-carer*).ab,ti,kf.) **AND** (Implementation Science/ OR (implement* OR adopt* OR uptake OR Appl* OR Carry-out OR Perform* OR Usage OR Practice OR Enactment OR Fulfil* OR knowledge-transfer* OR feasib* OR adapt* OR accept* OR appropriate* OR cost* OR fidelit* OR sustainab* OR penetrat* OR reach* OR utili* OR sustained-integration* OR intention-to-chang* OR embed* OR normali* OR Disseminat* OR diffus* OR (translat* ADJ3 knowledge*) OR scale-up OR scaling OR barrier* OR change-in-practice* OR Obstruct* OR Obstacle* OR Facilitat* OR Threat* OR access* OR retention OR retain* OR compatib* OR brakes OR levers OR (perception* ADJ3 attitude*)).ab,ti,kf.) **AND** (Education/ OR education.fx. OR Learning/ OR (intervent* OR training* OR educat* OR coach* OR learn* OR platform* OR innovation* OR program* OR session* OR technolog* OR audit OR reminder* OR ((web OR mobile*) ADJ3 (application*)) OR trainer* OR coach* OR psychoeducation* OR audit* OR feedback* OR psychotherap* OR (occupation* ADJ3 therap*) OR respite* OR day-care* OR evidence-based-practice* OR (modif* ADJ3 environment*) OR information-management-system* OR consultation* OR local-champion* OR local-leader* OR local-network* OR organi*ational-change* OR cognit*-behav*-therap*).ab,ti,kf.) *NOT (letter* OR news OR comment* OR editorial* OR congres* OR abstract* OR book* OR chapter* OR dissertation abstract*).pt. NOT (review).ti. NOT (acetylcholinesterase* OR donepezil* OR huntington* OR Parkinson).ti.* |
| **Web of Science**  (TI=(dementia* OR Alzheimer* OR Mild-cognitive-impairment*) OR AB=(dementia* OR Alzheimer* OR Mild-cognitive-impairment*)) **AND (**TI=(((caregiv* OR care-giv* OR carer* OR in-home-care* OR care-provider*) NEAR/2 (spous* OR famil* OR child* OR parent* OR informal* OR program* OR elder*)) OR dementia*-carer*) OR AB=(((caregiv* OR care-giv* OR carer* OR in-home-care* OR care-provider*) NEAR/2 (spous* OR famil* OR child* OR parent* OR informal* OR program* OR elder*)) OR dementia*-carer*)) **AND (**TI=(implement* OR adopt* OR uptake OR Appl* OR Carry-out OR Perform* OR Usage OR Practice OR Enactment OR Fulfil* OR knowledge-transfer* OR feasib* OR adapt* OR accept* OR appropriate* OR cost* OR fidelit* OR sustainab* OR penetrat* OR reach* OR utili* OR sustained-integration* OR intention-to-chang* OR embed* OR normali* OR Disseminat* OR diffus* OR (translat* NEAR/2 knowledge*) OR scale-up OR scaling OR barrier* OR change-in-practice* OR Obstruct* OR Obstacle* OR Facilitat* OR Threat* OR access* OR retention OR retain* OR compatib* OR brakes OR levers OR (perception* NEAR/2 attitude*)) OR AB=(implement* OR adopt* OR uptake OR Appl* OR Carry-out OR Perform* OR Usage OR Practice OR Enactment OR Fulfil* OR knowledge-transfer* OR feasib* OR adapt* OR accept* OR appropriate* OR cost* OR fidelit* OR sustainab* OR penetrat* OR reach* OR utili* OR sustained-integration* OR intention-to-chang* OR embed* OR normali* OR Disseminat* OR diffus* OR (translat* NEAR/2 knowledge*) OR scale-up OR scaling OR barrier* OR change-in-practice* OR Obstruct* OR Obstacle* OR Facilitat* OR Threat* OR access* OR retention OR retain* OR compatib* OR brakes OR levers OR (perception* NEAR/2 attitude*))) **AND** (TI=(intervent* OR training* OR educat* OR coach* OR learn* OR platform* OR innovation* OR program* OR session* OR technolog* OR audit OR reminder* OR ((web OR mobile*) NEAR/2 (application*)) OR trainer* OR coach* OR psychoeducation* OR audit* OR feedback* OR psychotherap* OR (occupation* NEAR/2 therap*) OR respite* OR day-care* OR evidence-based-practice* OR (modif* NEAR/2 environment*) OR information-management-system* OR consultation* OR local-champion* OR local-leader* OR local-network* OR organi*ational-change* OR cognit*-behav*-therap*) OR AB=(intervent* OR training* OR educat* OR coach* OR learn* OR platform* OR innovation* OR program* OR session* OR technolog* OR audit OR reminder* OR ((web OR mobile*) NEAR/2 (application*)) OR trainer* OR coach* OR psychoeducation* OR audit* OR feedback* OR psychotherap* OR (occupation* NEAR/2 therap*) OR respite* OR day-care* OR evidence-based-practice* OR (modif* NEAR/2 environment*) OR information-management-system* OR consultation* OR local-champion* OR local-leader* OR local-network* OR organi*ational-change* OR cognit*-behav*-therap*)) *AND DT=Article NOT TI=(acetylcholinesterase* OR donepezil* OR huntington* OR Parkinson)* |
| **Cochrane Central**  ((dementia* OR Alzheimer* OR Mild NEXT cognitive NEXT impairment*):ab,ti,kw) **AND** ((((caregiv* OR care NEXT giv* OR carer* OR in NEXT home NEXT care* OR care NEXT provider*) NEAR/3 (spous* OR famil* OR child* OR parent* OR informal* OR program* OR elder*)) OR dementia* NEXT carer*):ab,ti,kw) **AND** ((implement* OR adopt* OR uptake OR Appl* OR Carry NEXT out OR Perform* OR Usage OR Practice OR Enactment OR Fulfil* OR knowledge NEXT transfer* OR feasib* OR adapt* OR accept* OR appropriate* OR cost* OR fidelit* OR sustainab* OR penetrat* OR reach* OR utili* OR sustained NEXT integration* OR intention NEXT to NEXT chang* OR embed* OR normali* OR Disseminat* OR diffus* OR (translat* NEAR/3 knowledge*) OR scale NEXT up OR scaling OR barrier* OR change NEXT in NEXT practice* OR Obstruct* OR Obstacle* OR Facilitat* OR Threat* OR access* OR retention OR retain* OR compatib* OR brakes OR levers OR (perception* NEAR/3 attitude*)):ab,ti,kw) **AND** ((intervent* OR training* OR educat* OR coach* OR learn* OR platform* OR innovation* OR program* OR session* OR technolog* OR audit OR reminder* OR ((web OR mobile*) NEAR/3 (application*)) OR trainer* OR coach* OR psychoeducation* OR audit* OR feedback* OR psychotherap* OR (occupation* NEAR/3 therap*) OR respite* OR day NEXT care* OR evidence NEXT based NEXT practice* OR (modif* NEAR/3 environment*) OR information NEXT management NEXT system* OR consultation* OR local NEXT champion* OR local NEXT leader* OR local NEXT network* OR organi*ational NEXT change* OR cognit* NEXT behav* NEXT therap*):ab,ti,kw) *NOT (acetylcholinesterase* OR donepezil* OR huntington* OR Parkinson):ti* |
