# Supplementary material for: Implementation and dissemination of home- and community-based interventions for informal caregivers of people living with dementia: a systematic scoping review
Source: Implement Sci. 2023 Nov 8;18:60. doi: 10.1186/s13012-023-01314-y (PMC10631024; doi:10.1186/s13012-023-01314-y)
Supplement: Supplementary file 3 — Additional file 3. Results overview (detailed) Table 1. Results overview. Table 2. Overview of constructs found in included studies’ frameworks. Table 3. Barriers to Implementation. Table 4. Facilitators to Implementation. Table 5. Implementation strategies identified across studies (n=67) based on the ERIC compilation. Table 6. Implementation actions and corresponding strategies employed in included studies according to Waltz’s implementation clusters and ERIC taxonomy strategies. Table 7. Implementation Outcomes. Table 8. Common trends identified between implementation strategies and implementation outcomes. [file 13012_2023_1314_MOESM3_ESM.pdf]

Table 1. Results overview

EBI Title	Author(s), Year / Country of study origin	Study Design & *Implementation Framework (IF)*	MMAT Score	Participants (P) and Outcomes (O) reported	Implementation Clusters (1-9)	ERIC Taxonomy Discrete Strategies (1-73)	Implementation Outcomes	Barriers to Implementation [Domain / Construct]	Facilitators to Implementation [Domain / Construct]
eHealth interventions (n=15)									
Caring for Carers of People with Dementia study	Banbury et al. (2019) / Australia	Pre-post study design (Staggered randomized waitlist design)	100%	P: 16 groups with 69 participants<br>O: Changes in perceptions in using technology; Differences in communicating by videoconferencing; Technical support required	1 - Use evaluative and iterative strategies<br>2- Provide interactive assistance<br>3 - Use evaluative and iterative strategies	4<br>33, 8<br>51, 63	Acceptability<br>Appropriateness<br>Penetration<br>Sustainability	[I/G] Design Quality & Packaging<br>-<br>[II/A] Patient Needs & Resources	[I/D] Adaptability<br>[I/G] Design Quality and Packaging<br>-<br>[II/A] Patient Needs & Resources<br>-<br>[IV/B] Self-efficacy<br>[IV/E] Other Personal Attributes<br>-<br>[V/B] Engaging
iSupport	Baruah et al. (2020) / India	Focus group	100%	P: 13 caregivers<br>O: Expectations, experiences, challenges, and motivation of end-users	No implementation strategies identified	n/a	Acceptability<br>Appropriateness<br>Penetration	[I/D] Adaptability<br>[I/G] Design Quality & Packaging<br>-<br>[II/A] Patient Needs & Resources<br>-<br>[IV/E] Other Personal Attributes	No facilitators identified
	Teles et al. (2020) / Portugal	Mixed methods; online survey (Questionnaire)	100%	P: 88 informal caregivers<br>O: Caregiver training needs and preferences of application features	3 - Adapt and tailor to context<br>5 - Train and educate stakeholders	51<br>19, 29, 31	Appropriateness<br>Penetration	[I/D] Adaptability<br>[I/G] Design Quality & Packaging	[II/A] Patient Needs & Resources<br>[II/B] Cosmopolitanism
	Xiao et al. (2020) / Australia	Focus group	100%	P: 16 informal caregivers, 20 staff members<br>O: Opportunity for an online one-stop shop for informal caregivers; integrated online caregiver network; role of dementia care staff in promoting the iSupport to informal caregivers; time commitment to participate in the iSupport program; expectations of user-friendly iSupport	3 - Adapt and tailor to context<br>5 - Train and educate stakeholders	51, 63<br>29	Acceptability<br>Appropriateness	[I/D] Adaptability<br>[I/G] Design Quality & Packaging<br>-<br>[II/A] Patient Needs & Resources<br>[II/B] Cosmopolitanism<br>[II/D] External Policy and Incentives<br>-<br>[IV/B] Self-efficacy<br>[IV/E] Other Personal Attributes	[I/G] Design Quality & Packaging<br>[I/H] Cost<br>-<br>[II/A] Patient Needs & Resources<br>[II/D] External Policy & Incentives<br>-<br>[III/E2] Available Resources
Partner in Balance	Boots et al. (2017) / the Netherlands	Pre-post study design (Randomized	100%	P: 49 Participants (caregivers)<br>O: First-order implementation process (Sampling quality, intervention quality)	1 - Use evaluative and iterative strategies<br>2- Provide interactive assistance<br>3 - Adapt and tailor to context	5<br>33<br>51	Acceptability<br>Appropriateness<br>Feasibility<br>Penetration	[I/D] Adaptability<br>[I/G] Design Quality & Packaging<br>-	[I/D] Adaptability<br>[I/G] Design Quality & Packaging<br>-

		waiting-list controlled trial)<br><br>*(IF) Medical Research Council framework*		Second-order process data: implementation knowledge - obtained through barriers and facilitators questionnaire.	4 - Develop stakeholder interrelationships<br>5 - Train and educate stakeholders<br>6 - Support clinicians<br>7 - Engage consumers<br>9 - Change infrastructure	6, 52<br>71, 43, 16, 55, 19, 31<br>59<br>69<br>12	Sustainability	[II/A] Patient Needs & Resources<br>[II/B] Cosmopolitanism<br>-<br>[III/D2] Compatibility<br>[III/E2] Available Resources<br>[III/E3] Access to Knowledge & Information<br>-<br>[V/B] Engaging	[II/A] Patient Needs & Resources<br>[II/B] Cosmopolitanism<br>-<br>[III/D2] Compatibility<br>-<br>[IV/C] Individual State of Change
InLife	Dam et al. (2019) / the Netherlands	Pre-post study design (Randomized waiting-list controlled trial)<br><br>*(IF) Medical Research Council framework*<br><br>*(IF) Leontjevas Process Evaluation Model*	100%	P: 96 primary caregivers<br>O: Reasons for InLife use, user-friendliness of InLife, usage of InLife, relevance of InLife, satisfaction and recommendations for improvement of InLife, adherence to protocol, determinants for InLife use, determinants of the level of innovation, determinants at the level of InLife users, determinants at the level of the socio-political context	1 - Use evaluative and iterative strategies<br>2- Provide interactive assistance<br>3 - Adapt and tailor to context<br>5 - Train and educate stakeholders<br>7 - Engage consumers<br>9 - Change infrastructure	46<br>8<br>63<br>31<br>69<br>12	Acceptability<br>Adoption<br>Appropriateness<br>Penetration<br>Sustainability	[I/D] Adaptability<br>[I/G] Design Quality & Packaging<br>-<br>[II/B]<br>[II/D] External Policy and Incentives<br>-<br>[III/D2] Compatibility<br>-<br>[IV/A] Knowledge and Belief about the Intervention<br>[IV/E] Other Personal Attributes	[I/D] Adaptability<br>[I/G] Design Quality & Packaging<br>-<br>[II/A] Patient Needs & Resources<br>[II/B] Cosmopolitanism<br>-<br>[IV/B] Self-efficacy
eMR-ABC	Frame et al. (2013) / USA	Focus group (Case study)<br><br>*(IF) Reflective Adaptive Process*	100%	P: (unclear)<br>O: The eMR-ABC captures and monitors the cognitive, functional, behavioral, and psychological symptoms of a registry of patients suffering from dementia or depression as well as the burden of patients' family caregivers. It provides decision support to care coordinators to create a personalized care plan that includes evidence-based nonpharmacological protocols, self-management handouts, and alerts of medications with potentially adverse cognitive effects.	1 - Use evaluative and iterative strategies<br>3 - Adapt and tailor to context<br>5 - Train and educate stakeholders<br>6 - Support clinicians<br>9 - Change infrastructure	26<br>51<br>71<br>32<br>12	Adoption<br>Appropriateness<br>Sustainability	No barriers identified	[III/D6] Learning Culture
Alzheimer's Caregiver Support Online (AlzOnline)	Glueckauf and Loomis (2003) / USA	Pre-post study design with case report	100%	P: Phase 1 (AlzOnline staff members), Phase 2: 11 caregivers, 8 senior service professionals, 2 DOEA staff), process evaluation (40 caregivers)	1 - Use evaluative and iterative strategies<br>2- Provide interactive assistance<br>3 - Adapt and tailor to context<br>4 - Develop stakeholder interrelationships<br>5 - Train and educate stakeholders	5, 46, 4, 18<br>33<br>51, 63<br>6, 38<br>29, 43, 31	Appropriateness<br>Penetration<br>Sustainability	[I/D] Adaptability<br>[I/G] Design Quality & Packaging	[I/D] Adaptability<br>[I/G] Design Quality & Packaging<br>-<br>[II/B] Cosmopolitanism<br>-

				O: Clarity, usefulness, and ease of navigation of website reading materials, live caregiving classes, message board, and state and national web links.	9 - Change infrastructure	11			[III/E2] Available Resources<br>-<br>[V/B] Engaging<br>[V/B3] Champions<br>[V/B4] External Change Agents<br>[V/D] Reflecting & Evaluating
iGeriCare (Clinician's perspective)	Levinson et al. (2020) / Canada	Qualitative study (semi-structured interviews)<br><br>*(IF) Consolidated Framework for Implementation Research (CFIR)*	100%	P: 12 individuals involved in dementia care and caregiver education<br><br>O: Outer setting, Inner setting and personal barriers and facilitators to implementation of iGeriCare	1 - Use evaluative and iterative strategies	4	Acceptability<br>Adoption<br>Appropriateness<br>Penetration<br>Sustainability	[II/B]<br>[II/D] External Policy and Incentives<br>-<br>[III/E] Implementation Readiness – General<br>[III/E2]<br>-<br>[IV/D] Individual identification with the organization<br>-<br>[V/B] Engaging	[I/C] Relative Advantage<br>[I/F] Complexity<br>[I/G] Design Quality & Packaging<br>-<br>[II/A] Patient Needs & Resources<br>-<br>[III/D2] Compatibility<br>[III/E1] Leadership Engagement<br>[III/E2] Available Resources<br>-<br>[IV/A] Knowledge & beliefs about the intervention<br>[IV/C] Individual state of change<br>[IV/D] Individual Identification with Organization
					4 - Develop stakeholder interrelationships	38			
					5 - Train and educate stakeholders	19, 31, 43, 29			
Tele.TAnDem	Meichsner et al. (2018) / Germany	Pre-post study design (Randomized Quantitative)	100%	P: 19 participants in intervention group; 6 completed study<br><br>O: Acceptability, treatment effects, and changes in emotional well-being	2- Provide interactive assistance	33	Acceptability<br>Feasibility	No barriers identified	[I/G] Design Quality & Packaging<br>-<br>[II/A] Patient Needs & Resources
					3 - Adapt and tailor to context	51			
					5 - Train and educate stakeholders	29, 31, 55			
					9 - Change infrastructure	13			
RAM (Remote Activity Monitoring)	Mitchell et al. (2017) published in (2020) / USA	Parallel convergent mixed methods design employing both qualitative and quantitative method	100%	P: 30 caregivers included, 7 caregivers completed study<br><br>O: Acceptability and utility	2- Provide interactive assistance	33, 54	Acceptability<br>Appropriateness<br>Penetration<br>Sustainability	[I/G] Design Quality & Packaging<br>-<br>[II/A] Patient Needs & Resources<br>-<br>[IV/A] Knowledge & beliefs about the intervention	[I/G] Design Quality & Packaging<br>-<br>[III/E1] Leadership Engagement<br>-<br>[V/C] Executing
					3 - Adapt and tailor to context	51			
					9 - Change infrastructure	11, 12			
Cuidate Cuidador	Pagan-Ortiz et al. (2014) / USA	Quasi-experimental pre-post study design	80%	P: 72 participants (caregivers)	1 - Use evaluative and iterative strategies	18, 4	Acceptability<br>Appropriateness<br>Penetration	[I/D] Adaptability<br>[I/G] Design Quality & Packaging	[I/D] Adaptability<br>[I/G] Design Quality & Packaging
					2- Provide interactive assistance	8			

			Divergence between qualitative and quantitative data unclear	O: Website effectiveness and user acceptability/feedback (Appeal, usability and effectiveness of communication)	3 - Adapt and tailor to context<br>4 - Develop stakeholder interrelationships<br>5 - Train and educate stakeholders<br>7 - Engage consumers	51, 63, 67<br>52<br>29, 43, 31<br>69		-<br>[V/B] Engaging	-<br>[II/B] Cosmopolitanism<br>-<br>[V/B] Engaging
mastery over dementia (MoD)	Pot et al. (2015) / The Netherlands	Pre-post study design (Quantitative descriptive)	100%	P: 68 participants<br><br>O: Reach, Adherence and User evaluations	1 - Use evaluative and iterative strategies<br>2- Provide interactive assistance<br>5 - Train and educate stakeholders<br>6 - Support clinicians<br>7 - Engage consumers<br>9 - Change infrastructure	5<br>33<br>29, 43, 31, 55, 19<br>59<br>39<br>12, 13	Acceptability<br>Appropriateness<br>Penetration	[I/D] Adaptability<br>[I/G] Design Quality & Packaging<br>-<br>[II/A] Patient Needs & Resources<br>-<br>[V/C] Executing	[I/G] Design Quality & Packaging<br>-<br>[II/A] Patient Needs & Resources
Partner in Sight (PsyMate)	van Knippenberg et al. (2017) / The Netherlands	Pre-post study design (Process evaluation)<br><br>*Leontjevas Process Evaluation Model*	80%<br><br>Low participation rate (31.4%) may affect nonresponse bias	P: 295 caregivers recruited; 76 involved in study, and 26 participants in intervention group.<br><br>O: Sampling quality (recruitment barriers and facilitators, reach, sample characteristics) and intervention quality (relevance and feasibility, adherence to protocol)	3 - Adapt and tailor to context<br>5 - Train and educate stakeholders<br>7 - Engage consumers<br>9 - Train and educate stakeholders	51, 63<br>19, 71, 43<br>50<br>11, 12	Acceptability<br>Appropriateness<br>Penetration	[I/G] Design Quality & Packaging<br>-<br>[II/A] Patient Needs & Resources<br>-<br>[V/B] Engaging	[I/D] Adaptability<br>[I/G] Design Quality & Packaging<br>-<br>[V/B] Engaging
FamTechCare	Williams et al. (2020) / USA	Parallel convergent mixed methods design employing both qualitative and quantitative method	100%	P: 83 caregivers; 43 in intervention group<br><br>O: Satisfaction, utilization, feasibility of FamTechCare	1 - Use evaluative and iterative strategies<br>2- Provide interactive assistance<br>5 - Train and educate stakeholders<br>6 - Support clinicians<br>9 - Change infrastructure	26<br>8, 33<br>31, 43<br>21, 59<br>11	Acceptability<br>Appropriateness<br>Adoption<br>Feasibility	[I/G] Design Quality & Packaging<br>-<br>[II/B] Cosmopolitanism<br>[II/D] External Policy and Incentives<br>-<br>[III/E2] Implementation Readiness – Available Resources<br>-<br>[IV/E] Other Personal Attributes	[I/G] Design Quality & Packaging
Respite care (n=5)									
Adult day care - On Lok project / Program of All-Inclusive Care for the Elderly (PACE)	Beisecker et al. (1996) / USA	Qualitative study (semi-structured interviews)	100%	P: 104 participants (52 experimental/52 control)<br><br>O: Barriers and facilitators of adult day care from caregiver perspectives	No implementation strategies identified	n/a	Acceptability<br>Penetration	[I/G] Design Quality & Packaging<br>[I/H] Cost<br>-<br>[II/A] Patient Needs & Resources<br>-<br>[III/E2] Available Resources<br>[III/E3] Access to Knowledge & Information<br>-	[II/A] Patient Needs & Resources<br>[II/B] Cosmopolitanism<br>-<br>[III/E2] Available Resources

								[V/B] Engaging	
Caring for the Caregiver	Brandao et al. (2016) / Portugal	Cross-sectional design (Questionnaires; quantitative descriptive)	100%	P: 223 informal caregivers<br><br>O: Reasons for underuse and unawareness of residential respite care services (barriers)	No implementation strategies identified	n/a	Acceptability<br>Penetration	[I/H] Cost<br>-<br>[II/A] Patient Needs & Resources	[II/A] Patient Needs & Resources<br>[II/D] External Policy & Incentives<br>-
Adult day service (ADS)	Gaugler (2014) / USA	Qualitative study (semi-structured interviews)	100%	P: 12 ADS staff members, 14 family members of clients<br><br>O: Programmatic philosophy, positioning, and environment of ADS, reasons for ADS use, process of use, pathways to negative/positive outcomes	6 - Support clinicians<br>7 - Engage consumers<br>9 - Change infrastructure	59<br>39, 50<br>13	Acceptability<br>Appropriateness	[II/A] Patient Needs & Resources<br>-<br>[IV/D] Individual Identification with Organization<br>-<br>[V/B] Engaging	[I/C] Relative Advantage<br>[I/D] Adaptability<br>[I/G] Design Quality & Packaging<br>-<br>[II/A] Patient Needs & Resources<br>[II/B] Cosmopolitanism<br>-<br>[IV/E] Other Personal Attributes<br>-<br>[V/A] Planning<br>[V/B] Engaging
Adult day service Plus (ADS Plus)	Gitlin et al. (2019) / USA	Pre-post study design (Cluster randomized trial design)	100%	P: 49 sites, 26 intervention groups<br><br>O: Adult day service Plus consists of five key components: taking care of self, education, validation and support, referral and linkage, identification of care challenges, skill building and strategies.	1 - Use evaluative and iterative strategies<br>2- Provide interactive assistance<br>3 - Adapt and tailor to context<br>4 - Develop stakeholder interrelationships<br>5 - Train and educate stakeholders<br>6 - Support clinicians<br>8 - Utilize financial strategies	4, 5, 18, 23, 26, 56<br>33<br>63<br>35, 57<br>71, 15, 19, 43, 29, 31<br>59<br>2	Fidelity<br>Implementation Cost	No barriers identified	[V/B2] Formally Appointed Implementation Leaders
Adult Day Care (Respite Programming)	Roberts and Struckmeyer (2017) / USA	Qualitative study (semi-structured interviews)	100%	P: 33 participants<br><br>O: Family dynamics, caregiver isolation, financial struggles, seeking respite, caregiver acceptance and resilience	No implementation strategies identified	n/a	Acceptability<br>Appropriateness<br>Implementation Cost<br>Sustainability	[I/H] Cost<br>-<br>[II/A] Patient Needs & Resources<br>[II/D] External Policy & Incentives<br>-<br>[IV/E] Other Personal Attributes	[I/C] Relative Advantage<br>[I/G] Design Quality & Packaging<br>[I/H] Cost<br>-<br>[II/D] External Policy & Incentives<br>-<br>[III/E2] Available Resources
Psychoeducation (n=12)									
The booklet, Information for Families and Friends of People with Severe and	Chang et al. (2010) / Australia	Questionnaire	100%	P: 233 caregivers<br><br>O: Response to dementia information booklet, caregivers' preferred method of timing and	1 - Use evaluative and iterative strategies<br>4 - Develop stakeholder interrelationships<br>5 - Train and educate stakeholders	4<br>36, 52<br>29	Acceptability<br>Appropriateness<br>Penetration	[I/G] Design Quality & Packaging	[I/G] Design Quality & Packaging<br>-<br>[II/A] Patient Needs & Resources

End Stage Dementia (Palliative Care Dementia Interface: Enhancing Community Capacity				distribution, comments on effect of reading the booklet					
START (STrAtegies for RelaTives)	Foley et al. (2020) / UK	Pre-post study design (Quantitative descriptive)	80%<br><br>Small sample size (n=21) risks response bias	P: 21 carers<br><br>O: Quality of life, anxiety; written feedback revealing themes of a) appreciating having their difficulties recognized, b) enjoying being able to share concerns, c) improved confidence in their ability to care and feeling more prepared for the future.	3 - Adapt and tailor to context<br>4 - Develop stakeholder interrelationships<br>5 - Train and educate stakeholders<br>8 - Utilize financial strategies<br>9 - Change infrastructure	63, 51<br>35<br>19, 43<br>1<br>13	Acceptability<br>Appropriateness<br>Feasibility	No barriers identified	[IV/B] Self-efficacy
	Sommerlad et al. (2014) / UK	Questionnaire (qualitative)	100%	P: 75 respondents / 132 respondents for qualitative sub-study<br><br>O: Carers identified several different components as important: relaxation techniques, education about dementia, strategies to help manage the behaviour of the person with dementia, contact with the therapist and changing unhelpful thoughts.	5 - Train and educate stakeholders<br>9 - Change infrastructure	43, 31<br>13	Acceptability<br>Appropriateness<br>Sustainability	[I/G] Design Quality & Packaging<br>-<br>[II/A] Patient Needs & Resources	[I/G] Design Quality & Packaging<br>-<br>[II/A] Patient Needs & Resources<br>-<br>[IV/B] Self-efficacy
Tele-Savvy for Dementia Caregivers / The Savvy Caregiver Program	Griffiths et al. (2015) / USA	Pre-post study design	80%<br><br>Small sample size may influence effectivenes s results	P: 22 caregivers, 6 experts reviewing the adaptation<br><br>O: Fidelity of adapted intervention;<br>Caregiver evaluation - Burden, Depression, Anxiety, Caregiving competency, and behavioral/psychological symptoms of dementia and caregivers' responses	1 - Use evaluative and iterative strategies<br>2- Provide interactive assistance<br>5 - Train and educate stakeholders<br>7 - Engage consumers<br>9 - Change infrastructure	4, 46<br>33<br>19, 31, 43, 29<br>50<br>11	Acceptability<br>Appropriateness<br>Fidelity<br>Penetration<br>Sustainability	No barriers identified	[I/G] Design Quality & Packaging
	Kovaleva et al. (2017) / USA	Qualitative study (semi-structured interviews)	100%	P: 42 caregivers recruited; 36 completed intervention<br><br>O: End-user acceptability	3 - Adapt and tailor to context<br>5 - Train and educate stakeholders	63<br>19, 55, 29, 43, 31	Acceptability<br>Appropriateness<br>Penetration<br>Sustainability	[I/D] Adaptability<br>[I/G] Design Quality & Packaging<br>-<br>[III/E2] Available Resources	[I/G] Design Quality & Packaging
ANSWERS	Judge et al. (2010) / USA	Pre-post study design (Quantitative descriptive)	100%	P: 52 dyads	1 - Use evaluative and iterative strategies<br>3 - Adapt and tailor to context	26, 27, 5, 56<br>51	Acceptability<br>Appropriateness<br>Fidelity	[I/D] Adaptability	[I/D] Adaptability<br>-

				O: Acceptability and feasibility of ANSWERS for dementia patient and caregiver dyads	4 - Develop stakeholder interrelationships	57			[II/A] Patient's Needs & Resources
					5 - Train and educate stakeholders	19, 31, 71			
					9 - Change infrastructure	13			
REACH II	Lykens et al. (2014) / USA	Pre-post study design (without control group)	100%	P: 494 families, 177 families completed study<br><br>O: Domains assessed in pre-post test include caregiver burden, depression, self-care, social support	1 - Use evaluative and iterative strategies	4, 26, 27	Acceptability<br>Appropriateness<br>Penetration<br>Sustainability	[II/D] External Policy & Incentives	[I/G] Design Quality & Packaging<br>-<br>[II/A] Patient Needs & Resources<br>[II/B] Cosmopolitanism<br>[II/D] External Policy & Incentives<br>-<br>[III/E2] Available Resources<br>[III/E3] Access to Knowledge & Information<br>-<br>[IV/E] Other Personal Attributes<br>-<br>[V/B] Engaging
					2- Provide interactive assistance	54, 8			
					3 - Adapt and tailor to context	51			
					4 - Develop stakeholder interrelationships	52, 47, 6			
					5 - Train and educate stakeholders	73, 71, 43, 31			
					6 - Support clinicians	21, 59			
					9 - Change infrastructure	12			
REACH into Indian Country	Martindale-Adam et al. (2017) / USA	Randomized Waiting-list controlled trial (Pre-Post study design) / Qualitative Process evaluation<br><br>*Fixsen and Blasé implementation process model*	100%	P: (unclear)<br><br>O: Six implementation stages of exploration and adoption, program installation, initial implementation, full operation, innovation, and sustainability.	1 - Use evaluative and iterative strategies	61, 56, 26	Acceptability<br>Appropriateness<br>Adoption<br>Implementation Cost<br>Penetration<br>Sustainability	[IV/E] Other Personal Attributes	[I/G] Design Quality & Packaging<br>[I/H] Cost<br>-<br>[II/B] Cosmopolitanism<br>[II/C] Peer Pressure<br>[II/D] External Policy & Incentives<br>-<br>[III/D2] Compatibility<br>[III/E] Readiness for Implementation<br>[III/E1] Leadership Engagement<br>[III/E2] Available Resources<br>[III/E3] Access to Knowledge<br>-<br>[IV/B] Self-efficacy<br>[IV/D] Individual Identification with Organization<br>-<br>[V/B3] Champions<br>[V/C] Executing<br>[V/D] Reflecting & Evaluating
					2- Provide interactive assistance	33			
					3 - Adapt and tailor to context	51, 63			
					4 - Develop stakeholder interrelationships	72, 6, 40, 35			
					5 - Train and educate stakeholders	71, 29, 31, 15			
					6 - Support clinicians	30			
					7 - Engage consumers	37, 69			
					8 - Utilize financial strategies	34, 2, 70, 42			
					9 - Change infrastructure	44, 13, 22, 62			

Star-C	McCurry et al. (2015) / USA	Pre-post study design (without control group)	100%	P: 60 dyads completed 6 month follow up<br><br>O: caregiver depression, quality of life, and likelihood to institutionalize	1 - Use evaluative and iterative strategies	5, 56, 14	Acceptability<br>Appropriateness<br>Adoption<br>Feasibility<br>Fidelity<br>Penetration<br>Sustainability	[II/B] Cosmopolitanism<br>-<br>[III/D1] Tension for Change<br>-<br>[IV/E] Other Personal Attributes<br>-<br>[V/B] Engaging	[I/G] Design Quality & Packaging<br>-<br>[III/D2] Compatibility<br>-<br>[V/B3] Champions<br>[V/C] Executing<br>[V/D] Reflecting & Evaluating
					2- Provide interactive assistance	33			
					3 - Adapt and tailor to context	63			
					4 - Develop stakeholder interrelationships	35, 7, 40, 52			
					5 - Train and educate stakeholders	19, 29, 31, 43, 71			
					8 - Utilize financial strategies	34			
					7 - Engage consumers	69			
Medway Carers Course	Milne et al. (2014) / UK	Questionnaire (qualitative)	100%	P: 113 participants within 7 courses; 73 evaluation questionnaires<br><br>O: Changed approach, improved coping skills, therapeutic value of the course, social support and enjoyment, support services, style/timing/content of the course, valuable role of empathic experts, weakness of the course	2- Provide interactive assistance	33	Acceptability<br>Appropriateness<br>Sustainability	[I/G] Design Quality & Packaging	[I/G] Design Quality & Packaging<br>-<br>[II/A] Patient Needs & Resources<br>[II/D] External Policy & Incentives<br>-<br>[III/D2] Compatibility
					4 - Develop stakeholder interrelationships	6			
					5 - Train and educate stakeholders	29, 19, 31, 43			
					6 - Support clinicians	59, 21			
CARES Dementia Basics Program	Pleasant et al. (2016) / USA	Pre-post study design (Quantitative descriptive)	80%<br><br>24% completion rate risks response bias	P: 51 caregivers completed pre-test to follow-up test<br><br>O: Dementia knowledge questionnaire, the SCIDS scale (Sense of competence in Dementia care staff scale)	4 - Develop stakeholder interrelationships	52	Acceptability<br>Appropriateness<br>Penetration	No barriers identified	[II/B] Cosmopolitanism<br>[II/D] External Policy & Incentives
					5 - Train and educate stakeholders	43, 31			
					7 - Engage consumers	50			
					9 - Change infrastructure	13, 22			
Taking Care of YOU: Self-Care for Family Caregivers Toolkit	Smith and Graves (2020) / USA	Pre-post study design (without control group)	80%<br><br>Small sample size (n=35)	P: 35 caregivers<br><br>O: Stress inventory test (CSAQ questionnaire)	1 - Use evaluative and iterative strategies	4	Acceptability<br>Appropriateness<br>Penetration	[II/A] Patient Needs & Resources<br>[II/D] External Policy & Incentives	No facilitators identified
					2- Provide interactive assistance	33			
					4 - Develop stakeholder interrelationships	64			
					5 - Train and educate stakeholders	19, 29			
Exercise (n=3)									
TAiChI for people with demenTia (TACIT trial)	Barrado-Martin et al. (2019) / UK	Focus group	100%	P: 53 people contacted, 8 completed pilot program<br><br>O: Acceptability of class and home based tai chi practice; participant feelings toward the intervention and their dyadic participation	1 - Use evaluative and iterative strategies	4, 56	Acceptability<br>Appropriateness<br>Penetration	[I/D] Adaptability<br>[I/G] Design Quality & Packaging<br>-<br>[IV/A] Knowledge & Beliefs about the Intervention<br>[IV/B] Self-efficacy	[I/D] Adaptability<br>[I/G] Design Quality & Packaging<br>-<br>[V/B] Engaging
					2- Provide interactive assistance	33			
					3 - Adapt and tailor to context	63, 51			
					4 - Develop stakeholder interrelationships	57			
					5 - Train and educate stakeholders	31, 16, 29, 19			
					9 - Change infrastructure	12			
	Barrado-Martin et al. (2020) / UK	Questionnaire	100%	P: 86 dyads, 22 dyads observed, 15 interviewed<br><br>O:1) Supportive materials (educational booklet)<br>2) Behavior change techniques to support home practice	2- Provide interactive assistance	33	Acceptability<br>Appropriateness<br>Penetration	[I/D] Adaptability<br>[I/G] Design Quality & Packaging<br>-<br>[IV/B] Self-efficacy	[I/D] Adaptability<br>[I/G] Design Quality & Packaging<br>-<br>[IV/E] Other Personal Attributes
					4 - Develop stakeholder interrelationships	57			
					5 - Train and educate stakeholders	55, 31, 43			
					9 - Change infrastructure	11, 12			

				(action and coping plan, clock, home-exercise log)<br>3) Ways of practicing (preferences, role of routine, progress over practice)				[IV/E] Other Personal Attributes	
Reducing Disability in Alzheimer Disease (RDAD) program	Prick et al. (2014) / The Netherlands	Pre-Post study design / Process evaluation<br><br>*(IF) Process evaluation model by Reelick and colleagues*	100%	P: 111 dyads involved, 57 dyads in intervention group<br><br>O: Quantitative outcome measures were directed at mood, burden, and general health for caregivers	1 - Use evaluative and iterative strategies<br>3 - Adapt and tailor to context<br>4 - Develop stakeholder interrelationships<br>5 - Train and educate stakeholders<br>6 - Support clinicians<br>7 - Engage consumers<br>8 - Utilize financial strategies<br>9 - Change infrastructure	56<br>63, 51<br>52<br>19, 31<br>30<br>69<br>49<br>13	Acceptability<br>Appropriateness<br>Feasibility<br>Penetration<br>Sustainability	[I/G] Design Quality & Packaging<br>-<br>[II/A] Patient Needs & Resources<br>-<br>[IV/E] Other Personal Attributes<br>-<br>[V/B] Engaging	[I/G] Design Quality & Packaging
Care coordination and case management (n=6)									
Cleveland Alzheimer's managed Care Demonstratio n	Bass et al. (2003) / USA	Pre-post study design	100%	P: 157 participants<br><br>O: Caregiver utilization, satisfaction, depression and strain outcomes	1 - Use evaluative and iterative strategies<br>2- Provide interactive assistance<br>3 - Adapt and tailor to context<br>4 - Develop stakeholder interrelationships<br>5 - Train and educate stakeholders<br>6 - Support clinicians<br>7 - Engage consumers<br>8 - Utilize financial strategies	27, 4<br>33<br>63<br>52<br>55, 71, 19, 15, 43<br>59, 21, 30<br>39<br>49, 66, 34	Acceptability<br>Appropriateness<br>Feasibility<br>Penetration	[I/C] Relative Advantage	[I/C] Relative Advantage<br>[I/D] Adaptability<br>[I/G] Design Quality & Packaging<br>-<br>[II/A] Patient Needs & Resources<br>[II/B] Cosmopolitanism<br>-<br>[V/B] Engaging<br>[V/B2] Formally Appointed Implementation Leaders
Partners in Dementia Care	Bass et al. (2014) / USA	Pre-post study design (non-randomized)	80%<br><br>Adherence to study protocol varied	P: 122 participants (12-month follow-up)<br><br>O: Outcome of intervention examined: unmet need, embarrassment, isolation, relationship strain, depression	2- Provide interactive assistance<br>4 - Develop stakeholder interrelationships<br>5 - Train and educate stakeholders<br>6 - Support clinicians<br>7 - Engage consumers<br>8 - Utilize financial strategies<br>9 - Change infrastructure	8<br>52, 6, 36, 72, 24<br>73, 19, 71<br>59, 30, 21<br>50, 41<br>66<br>22, 12, 13	Acceptability<br>Implementation Cost<br>Penetration<br>Sustainability	No barriers identified	[I/G] Design Quality & Packaging<br>-<br>[II/B] Cosmopolitanism<br>[II/D] External Policy & Incentives<br>-<br>[III/E] Readiness for Implementation<br>-<br>[V/B2] Formally Appointed Internal Implementation Leaders<br>[V/C] Executing
Aged Care Assessment Teams	Bruce and Patterson (2000) / Australia	Qualitative study (semi-structured interviews)	100%	P: 24 (carers) respondents completed interview<br><br>O: Impact of caring role; source of stress for caregivers; service access and general practitioner	No implementation strategies identified	n/a	Acceptability<br>Appropriateness<br>Penetration<br>Sustainability	[I/B] Evidence Strength & Quality<br>[I/G] Design Quality & Packaging<br>-<br>[II/A] Patient Needs & Resources<br>[II/B] Cosmopolitanism	[II/B] Cosmopolitanism<br>[II/D] External Policy & Incentives

Community Outreach Education Program (COEP)	Connell and Kole (1999) / USA	Focus group	100%	P: (unclear); health care professionals, service providers, staff of community organizations and voluntary agencies, and family caregivers<br><br>O: The process of planning and implementing the community-based educational interventions.	1 - Use evaluative and iterative strategies<br>4 - Develop stakeholder interrelationships<br>5 - Train and educate stakeholders<br>6 - Support clinicians<br>7 - Engage consumers<br>8 - Utilize financial strategies<br>9 - Change infrastructure	4, 56<br>47, 52, 17, 24, 64, 6, 38, 40, 48<br>29, 15<br>30, 59<br>37, 69<br>1, 34<br>13	Acceptability<br>Penetration<br>Sustainability	[II/B] Cosmopolitanism<br>-<br>[III/D1] Tension for Change<br>[III/D2] Compatibility<br>[III/D3] Relative Priority<br>[III/E1] Leadership Engagement<br>[III/E2] Available Resources<br>-<br>[V/A] Planning<br>[V/B] Engaging<br>[V/C] Executing	[II/B] Cosmopolitanism<br>-<br>[III/E1] Leadership Engagement<br>[III/E2] Available Resources<br>-<br>[V/A] Planning<br>[V/B] Engaging<br>[V/B1] Opinion Leaders<br>[V/B2] Formally Appointed Internal Implementation Leaders<br>[V/B3] Champions<br>[V/B4] External Change Agents<br>[V/C] Executing<br>[V/D] Reflecting & Evaluating
Healthcare Professional Support	Laparidou et al. (2018) / UK	Focus group	100%	P: 17 informal caregivers of patients with dementia and 17 HCPs, including eight general practitioners (GPs), four practice nurses, a phlebotomist, an occupational therapist, a ward sister, a ward manager and a charge nurse.<br><br>O: The challenge of diagnosing dementia (primary stressor); caregivers' needs and expectations of an in-depth knowledge and understanding of dementia from HCPs (primary stressor); need for carer education (primary stressor); lack of support and mismatch of communication and expectations (secondary role strain); and carer involvement in monitoring care and disease (potential mediator tool).	2- Provide interactive assistance<br>4 - Develop stakeholder interrelationships<br>6 - Support clinicians	33<br>24, 52, 36, 64<br>59, 21	Acceptability<br>Penetration	[II/A] Patient Needs & Resources<br>[II/B] Cosmopolitanism<br>-<br>[III/E3] Access to Knowledge & Information	No facilitators identified
SUSTAIN program	Mavandadi et al. (2017) / USA	Pre-post study design (without control group)	80%<br><br>Program personnel were not blind to arm assignment	P: 440 older adults enrolled in SUSTAIN<br><br>O: Evaluation of outcomes for caregiver and care recipient	1 - Use evaluative and iterative strategies<br>2- Provide interactive assistance<br>3 - Adapt and tailor to context<br>4 - Develop stakeholder interrelationships<br>5 - Train and educate stakeholders<br>8 - Utilize financial strategies	4<br>33<br>51, 63<br>52<br>29, 43, 31, 55<br>34	Acceptability<br>Appropriateness<br>Penetration<br>Sustainability	No barriers identified	[I/D] Adaptability<br>[I/G] Design Quality & Packaging<br>[I/H] Cost<br>-<br>[V/B] Engaging

					9 - Change infrastructure	13			
Occupational Therapy (n=3)									
Community Occupational Therapy in Dementia (COTiD) program	Burgess et al. (2020) / UK	Qualitative study (semi-structured interviews)	100%	P: 22 dyads, 7 occupational therapists<br><br>O: The following themes were identified: (1) Valuing the occupational focus of COTiD-UK, (2) timing and relationships and (3) achieving goals.	1 - Use evaluative and iterative strategies<br>3 - Adapt and tailor to context<br>5 - Train and educate stakeholders<br>7 - Engage consumers<br>9 - Change infrastructure	4<br>51<br>19, 43<br>50<br>13	Acceptability<br>Appropriateness<br>Penetration	[II/A] Patient Needs & Resources<br>-<br>[III/E2] Available Resources<br>-<br>[IV/E] Other Personal Attributes	[I/G] Design Quality & Packaging<br>-<br>[II/A] Patient Needs & Resources<br>-<br>[IV/B] Self-efficacy
VALID - Occupational Therapy	Field et al. (2019) / UK	Qualitative study (semi-structured interviews)	100%	P: 34 participants (17 dyads)<br><br>O: Four themes identified:<br>1) How uptake was influenced by the impact of dementia on people who wanted support to adjust or cope with symptoms **(impact of dementia and wanting support)**<br>2) Concerning whether or not people felt they had enough activities (subtheme identified that previous experience with interventions may affect uptake)<br>3) limited initial expectations people appeared to have with the intervention<br>4) positive attitudes toward trying the intervention (despite feeling uncertain or worried)	1 - Use evaluative and iterative strategies<br>3 - Adapt and tailor to context<br>4 - Develop stakeholder interrelationships<br>5 - Train and educate stakeholders<br>6 - Support clinicians	4, 18<br>63, 51<br>52<br>19<br>21	Acceptability<br>Appropriateness<br>Penetration	[II/A] Patient Needs & Resources<br>-<br>[IV/E] Other Personal Attributes	[I/G] Design Quality & Packaging<br>-<br>[II/A] Patient Needs & Resources<br>[II/B] Cosmopolitanism<br>[II/D] External Policy & Incentives<br>-<br>[IV/E] Other Personal Attributes
Environmenta l skill-building program (ESP)	Gitlin et al. (2010) / USA	Pre-post study design<br><br>*(IF) RE-AIM (Reach, Effectiveness, Adoption, Implementation and Maintenance)*	100%	P: 41 caregivers<br><br>O: Reach, Effectiveness, Adoption, Implementation, Maintenance	1 - Use evaluative and iterative strategies<br>3 - Adapt and tailor to context<br>4 - Develop stakeholder interrelationships<br>5 - Train and educate stakeholders<br>8 - Utilize financial strategies	4, 18, 56<br>63<br>17, 6, 25<br>20, 73, 43, 71<br>49, 70	Acceptability<br>Adoption<br>Appropriateness<br>Feasibility<br>Fidelity<br>Implementation Cost<br>Penetration<br>Sustainability	[I/G] Design Quality & Packaging<br>-<br>[II/D] External Policy & Incentives<br>-<br>[III/D] Implementation Climate<br>-<br>[V/B] Engaging<br>[V/C] Executing	[I/G] Design Quality & Packaging<br>-<br>[II/D] External Policy & Incentives<br>-<br>[III/D] Implementation Climate [III/E] Readiness for Implementation<br>-<br>[V/A] Planning
Multi-component (n=18)									
REACH	Burgio et al. (2001) / USA	Qualitative study (case study)<br><br>*(IF) Lichstein's TI model*	80%<br><br>Missing data on confounders	P: (unclear)<br><br>O: REACH TI strategies; the approaches taken in REACH vary with the intervention protocols and include using treatment manuals, training and certification of interventionists,	1 - Use evaluative and iterative strategies<br>2- Provide interactive assistance<br>3 - Adapt and tailor to context<br>4 - Develop stakeholder interrelationships<br>5 - Train and educate stakeholders	27, 5<br>53<br>63, 51, 68<br>57, 52<br>29, 31, 71, 43	Feasibility<br>Sustainability	[I/F] Complexity<br>-<br>[III/E2] Available Resources<br>-<br>[IV/E] Other Personal Attributes<br>-	[I/G] Design Quality & Packaging<br>-<br>[V/A] Planning<br>[V/C] Executing<br>[V/D] Reflecting & Evaluating

				and continuous monitoring of actual implementation.	8 - Utilize financial strategies<br>9 - Change infrastructure	34, 1<br>12, 22		[V/A] Planning	
REACH OUT (Offering Useful Treatments) - Adaptation of REACH II for use in Area Agencies on Aging	Burgio et al. (2009) / USA	Quasi-experimental pre-post test design (without control group; non-randomized)	100%	P: 6 focus group participants; 227 dyads enrolled<br><br>O: Average of 95.2% completion rate; minimal improvement seen in PWD memory. Caregivers showed positive outcomes (Zarit Burden Scale; apart from ADL stress and behavioral bother)	1 - Use evaluative and iterative strategies<br>2- Provide interactive assistance<br>3 - Adapt and tailor to context<br>4 - Develop stakeholder interrelationships<br>5 - Train and educate stakeholders<br>9 - Change infrastructure	27<br>33, 8<br>63, 51<br>47, 24, 6, 40, 64, 25<br>31, 16, 71, 43, 55<br>12, 11	Acceptability<br>Adoption<br>Appropriateness<br>Feasibility<br>Fidelity<br>Penetration<br>Sustainability	[I/D] Adaptability<br>[I/G] Design Quality & Packaging<br>-<br>[III/D] Implementation Climate	[I/G] Design Quality & Packaging<br>-<br>[II/B] Cosmopolitanism
REACH-TX (a community-based translation of REACH II)	Cho et al. (2019) / USA	Pre-post study design (non-randomized)	100%	P: 895 participants (post-12 month follow up)<br><br>O: This study provides evidence of the impact of a community-based service program modeled on a leading evidence-based intervention for family caregivers for persons living with Alzheimer's disease and dementia. Evaluation data of a modified version of the REACH II intervention (REACH-TX) presented here supports the feasible translation and implementation of REACH II by a community-based agency that yields positive benefits for family caregivers. Family caregivers for care recipients) were positively affected by REACH-TX as demonstrated by change over time on the core QOL instruments used in the clinical trial of the evidence-based caregiver education and skill-training program.	1 - Use evaluative and iterative strategies<br>4 - Develop stakeholder interrelationships<br>5 - Train and educate stakeholders	4, 56<br>47, 52<br>71, 55, 15, 43, 31, 29	Acceptability<br>Feasibility<br>Penetration<br>Sustainability	No barriers identified	[II/B] Cosmopolitanism
iMCSP	Droes et al. (2019) / the Netherlands	Pre-post study design	100%	P: 16 Meeting Centers; 82 participants in 11 MC - DemenTalent- 39 participated in effect evaluation<br><br>O: Comparing MCSP and iMCSP; comparing DemenTalent and MCSP; comparing STAR and MCSP, satisfaction with DemenTalent compared to MCSP; Effect evaluation of iMCSP and DemenTalent	1 - Use evaluative and iterative strategies<br>4 - Develop stakeholder interrelationships<br>5 - Train and educate stakeholders<br>7 - Engage consumers<br>8 - Utilize financial strategies	4, 18<br>24, 6, 35, 7<br>19, 71<br>69<br>1	Acceptability<br>Appropriateness<br>Implementation Cost<br>Penetration<br>Sustainability	No barriers identified	[II/B] Cosmopolitanism<br>-<br>[III/E3] Access Knowledge<br>-<br>[V/B] Engaging<br>[V/B4] External Change Agents<br>[V/C] Executing
Care of Persons with		Pre-post study design	80%	P: 290 CHCPE (in trial)	1 - Use evaluative and iterative strategies	4, 18, 5, 26	Fidelity<br>Penetration	No barriers identified	[I/D] Adaptability<br>-

Dementia in their Environment (COPE) integrated in Connecticut Home Care Program for Elders (CHCPE)	Fortinsky et al. (2016) / USA	*(IF) guided by Normalization Process Theory (NPT)*	Study only presented a rationale for a hybrid effectiveness-implementation study without results	O: Determine COPE effect on perceived CG well-being 4 months after randomization; COPE effects on CG confidence in using dementia management strategies 4 months after randomization; COPE effects on CG perceived well-being, confidence in using activities, and ability to keep client at home, 12 months after randomization.	2- Provide interactive assistance<br>3 - Adapt and tailor to context<br>4 - Develop stakeholder interrelationships<br>5 - Train and educate stakeholders<br>6 - Support clinicians<br>9 - Change infrastructure	33, 54, 53<br>63<br>52, 6<br>43, 29, 31, 16<br>21, 32, 30, 59<br>11, 12	Sustainability		[II/B] Cosmopolitanism<br>-<br>[V/A] Planning
NYU Caregiver-Adult Child Intervention	Gaugler et al. (2018) / USA	Parallel convergent mixed methods design employing both qualitative and quantitative method<br><br>*(IF) Medical Research Council Framework*	100%	P: 54 participants (treatment group)<br><br>O: Adult children receiving the NYUCI-AC completed a mean of 5.19 individual and family counseling sessions; it took on average a little over 11 months to do so. All NYUCI-AC counseling components were generally well-received and improved caregivers' management of care-related stress. The individual counseling sessions' clinical benefits in reducing primary subjective stress were most apparent in the first year of the intervention. Caregivers who experienced negative outcomes over time used more family sessions.	2- Provide interactive assistance<br>5 - Train and educate stakeholders<br>7 - Engage consumers	33<br>43, 19<br>50	Acceptability<br>Appropriateness<br>Feasibility	[II/A] Patient Needs & Resources	[I/C] Relative Advantage<br>[I/G] Design Quality & Packaging<br>-<br>[IV/E] Other Personal Attributes<br>-<br>[V/D] Reflecting & Evaluating
Unforgettable (interactive museum program)	Hendriks et al. (2018) / The Netherlands	Qualitative study (semi-structured interviews); multiple case study<br><br>*(IF) Adaptive Implementation Model*	100%	P: 23 museums, 12 stakeholders [staff]<br><br>O: Program implementation and barriers and facilitators	1 - Use evaluative and iterative strategies<br>3 - Adapt and tailor to context<br>4 - Develop stakeholder interrelationships<br>5 - Train and educate stakeholders<br>6 - Support clinicians<br>7 - Engage consumers	61, 4<br>63, 51<br>57, 6, 24, 72, 36<br>43, 71<br>59, 30<br>41	Acceptability<br>Fidelity<br>Sustainability	[III/E1] Leadership Engagement<br>[III/E3] Access to Knowledge & Information<br>-<br>[V/B] Engaging<br>[V/B2] Formally Appointed Internal Implementation Leaders	[II/B] Cosmopolitanism<br>-<br>[III/B] Network and Communications<br>[III/D2] Compatibility<br>[III/E3] Access to Knowledge<br>-<br>[IV/E] Other Personal Attributes<br>-<br>[V/A] Planning<br>[V/B] Engaging<br>[V/B3] Champions
RDAD	Menne et al. (2014) / USA	Pre-post study design (without	100%	P: 219 participants	1 - Use evaluative and iterative strategies<br>3 - Adapt and tailor to context	5, 56<br>51, 63	Appropriateness<br>Feasibility	No barriers identified	[I/D] Adaptability<br>-<br>[II/B] Cosmopolitanism

		control group; non-randomized)		O: Two caregiver outcomes include unmet needs and care efficacy, improved for RDAD enrollees, regardless of the number of sessions of exercise, behavior management, and dementia education. RDAD was associated with decreases in a broad range of unmet needs including in areas such as understanding memory and dementia-related behavior problems. Improved care efficacy meant caregivers who used RDAD felt more capable and confident to manage their caregiving tasks and responsibilities after completing the 12 program sessions.	4 - Develop stakeholder interrelationships<br>5 - Train and educate stakeholders	57, 64, 52<br>31, 19, 71, 43, 29	Penetration<br>Sustainability		-<br>[III/D2] Compatibility<br>-<br>[V/B] Engaging<br>[V/B4] External Change Agents
Savvy Caregiver + REACH II	Meyer et al. (2018) / USA	Qualitative study (semi-structured interviews)	100%	P: 16 participants (15 interviews), 21 key stakeholders<br><br>O: Recruitment and engagement of participants AND Intervention content (refinements)	2- Provide interactive assistance<br>4 - Develop stakeholder interrelationships<br>5 - Train and educate stakeholders<br>7 - Engage consumers	33<br>38<br>19<br>39, 41	Acceptability<br>Adoption<br>Appropriateness<br>Penetration<br>Sustainability	[II/A] Patient Needs & Resources<br>-<br>[IV/E] Other Personal Attributes	[I/C] Relative Advantage<br>[I/D] Adaptability<br>-<br>[II/A] Patient Needs & Resources<br>-<br>[IV/A] Knowledge & Beliefs about the Intervention<br>[IV/E] Other Personal Attributes<br>-<br>[V/B] Engaging<br>[V/B1] Opinion Leaders<br>[V/B3] Champions<br>[V/C] Executing
multicomponent non-pharmacological interventions (NPIs)	Milders et al. (2016) / UK	Pre-post study design (Treatment group and waitlist (delayed treatment group); non-randomized)	100%	P: 30 dyads<br><br>O: The focus in Study 1 was treatment fidelity, costs and acceptability of the intervention and in Study 2 the feasibility of implementing the intervention in the community.	1 - Use evaluative and iterative strategies<br>3 - Adapt and tailor to context<br>4 - Develop stakeholder interrelationships<br>5 - Train and educate stakeholders	5, 56<br>51, 63<br>57, 64, 52<br>31, 19, 71, 43, 29	Acceptability<br>Appropriateness<br>Fidelity<br>Implementation Cost<br>Penetration<br>Sustainability	No barriers identified	[I/G] Design Quality & Packaging<br>-<br>[III/E3] Access to Knowledge & Information<br>-<br>[V/B4] External Change Agents
REACH VA	Nichols et al. (2011) / USA	Pre-post study design	100%	P: 127 enrollees (24 facilities)<br><br>O: The caregivers showed significant improvements in burden, depression, impact of depression on daily lives, and caregiving frustrations (screaming or yelling, feeling like hitting or slapping). The	2- Provide interactive assistance<br>4 - Develop stakeholder interrelationships<br>5 - Train and educate stakeholders<br>6 - Support clinicians<br>8 - Utilize financial strategies	33<br>6<br>71, 43, 31<br>59<br>34	Acceptability<br>Appropriateness<br>Penetration<br>Sustainability	[II/D] External Policy & Incentives<br>-<br>[III/E2] Available Resources	[I/G] Design Quality & Packaging<br>-<br>[II/D] External Policy & Incentives<br>-<br>[III/D2] Compatibility

				difference of almost 2 hours in the amount of time per day spent on duty trended toward significance.					[III/E3] Access to Knowledge & Information<br>-<br>[V/B] Engaging
	Nichols et al. (2016) / USA	Pre-post study design (RCT/Process Evaluation)<br><br>*(IF) Fixsen and Blasé implementation process model*	100%	P: 125 participants analyzed<br><br>O: REACH VA implementation process (exploration and adoption, Program installation, Initial implementation, Full operation, Innovation, Sustainability)	1 - Use evaluative and iterative strategies<br>2- Provide interactive assistance<br>4 - Develop stakeholder interrelationships<br>5 - Train and educate stakeholders<br>6 - Support clinicians<br>7 - Engage consumers<br>8 - Utilize financial strategies<br>9 - Change infrastructure	4, 56, 61, 14<br>8<br>47, 17, 35<br>29, 19, 43, 31, 71<br>59<br>69, 37<br>34, 49<br>22, 44	Acceptability<br>Adoption<br>Feasibility<br>Fidelity<br>Penetration<br>Sustainability	[II/B] Cosmopolitanism<br>-<br>[III/D1] Tension for Change<br>-<br>[V/D] Reflecting & Evaluating	[I/D] Adaptability<br>[I/E] Trialability<br>[I/G] Design Quality & Packaging<br>-<br>[II/A] Patient's Needs and Resources<br>[II/B] Cosmopolitanism<br>[II/D] External Policy & Incentives<br>-<br>[III/D1] Tension for Change<br>[III/D2] Compatibility<br>[III/E1] Leadership Engagement<br>-<br>[IV/B] Self-efficacy<br>-<br>[V/C] Executing
New York University Caregiver Intervention (NYUCI) - Minnesota Family Memory Care	Mittelman and Bartel (2014) / USA	Pre-post study design (Qualitative case study)	100%	P: 103 participants completed all 6 sessions<br><br>O: caregiver stress, depression; PWD time to institutionalization	4 - Develop stakeholder interrelationships<br>5 - Train and educate stakeholders<br>6 - Support clinicians<br>7 - Engage consumers<br>8 - Utilize financial strategies<br>9 - Change infrastructure	52, 35, 48<br>19, 71<br>59<br>69<br>1, 34, 49<br>12	Acceptability<br>Appropriateness<br>Penetration<br>Sustainability	[III/D2] Compatibility<br>[III/E2] Available Resources<br>-<br>[V/A] Planning<br>[V/C] Executing	[II/D] External Policy & Incentives<br>-<br>[III/E3] Access to Knowledge & Information<br>-<br>[V/A] Planning<br>[V/B4] External Change Agents<br>[V/C] Executing<br>[V/D] Reflecting & Evaluating
SHARE Program	Orsulic-Jeras et al. (2016) / USA	Pre-post study design (without control group)	80%<br><br>Small sample size (n=40) risks response bias	P: 40 caregivers completed interviews; 5 SHARE counselors<br><br>O: Findings examined in this paper include: (1) general acceptability and feasibility of the program; (2) whether or not the strategies of building rapport, establishing buy-in, and effective communication were helpful in supporting early-stage decision making and care planning; (3) emerging themes coded from CG and PWD comments on the benefits	1 - Use evaluative and iterative strategies<br>2- Provide interactive assistance<br>5 - Train and educate stakeholders<br>6 - Support clinicians	4<br>33<br>19, 59, 71, 55, 31 , 43<br>59	Acceptability<br>Appropriateness<br>Feasibility<br>Fidelity<br>Penetration	[I/G] Design Quality & Packaging<br>-<br>[II/A] Patient Needs & Resources	[I/G] Design Quality & Packaging<br>-<br>[III/D2] Compatibility<br>[III/E3] Access to Knowledge & Information

				and drawbacks of SHARE; and (4) data from baseline SHARE Care Circles derived from the Care Preferences Scale (CPS)					
New York University Caregiver Intervention (NYUCI) - Minnesota Family Memory Care	Paone (2014) / USA	Pre-post study design (Mixed method;<br><br>*(IF) RE-AIM (Reach, Effectiveness, Adoption, Implementation and Maintenance)*	100%	P: 137 participants (completed program)<br><br>O: The evaluation focused on: Whether/how program sites were able to follow the intervention protocol set forth by the researchers who created the NYUCI intervention; Whether/how the intervention was becoming embedded into the pro-gram sites and into the practices of the interventionists (maintenance/sustainability of the program); The cost to produce the intervention in the Minnesota program sites; The perceived value of and satisfaction with the program by caregivers.	1 - Use evaluative and iterative strategies<br>2- Provide interactive assistance<br>4 - Develop stakeholder interrelationships<br>5 - Train and educate stakeholders<br>6 - Support clinicians<br>7 - Engage consumers<br>8 - Utilize financial strategies<br>9 - Change infrastructure	27<br>33<br>65<br>55<br>59<br>69<br>1, 34<br>11, 22	Acceptability<br>Adoption<br>Fidelity<br>Implementation Cost<br>Penetration<br>Sustainability	[I/C] Relative Advantage<br>[I/F] Complexity<br>[I/H] Cost<br>-<br>[II/A] Patient Needs & Resources<br>-<br>[III/D2] Compatibility<br>[III/E2] Available Resources<br>-<br>[IV/A] Knowledge & Beliefs about the Intervention<br>[IV/E] Other Personal Attributes<br>-<br>[V/A] Planning<br>[V/B] Engaging<br>[V/B3] Champions<br>[V/D] Reflecting & Evaluating	[I/C] Relative Advantage<br>[I/D] Adaptability<br>-<br>[II/D] External Policy & Incentives<br>-<br>[III/C] Culture<br>[III/D2] Compatibility<br>[III/E2] Available Resources<br>-<br>[IV/B] Self-efficacy<br>[IV/E] Other Personal Attributes<br>-<br>[V/A] Planning<br>[V/B4] External Change Agents<br>[V/D] Reflecting & Evaluating
Maine Savvy Caregiver	Samia et al. (2014) / USA	Pre-post study design (Quasi-experimental)<br><br>*(IF) RE-AIM (Reach, Effectiveness, Adoption, Implementation and Maintenance)*	100%	P: 164 participants<br><br>O: Reach, Effectiveness, Adoption, Implementation, Maintenance	1 - Use evaluative and iterative strategies<br>4 - Develop stakeholder interrelationships<br>5 - Train and educate stakeholders<br>7 - Engage consumers<br>8 - Utilize financial strategies<br>9 - Change infrastructure	61<br>64, 24, 52, 35, 36, 6<br>29, 71, 73<br>69<br>1<br>22	Acceptability<br>Adoption<br>Appropriateness<br>Fidelity<br>Penetration<br>Sustainability	[II/B] Cosmopolitanism<br>[II/D] External Policy & Incentives<br>-<br>[V/B3] Champions	[II/B] Cosmopolitanism<br>[II/D] External Policy & Incentives<br>-<br>[III/C] Culture<br>[III/E2] Available Resources<br>[III/E3] Access to Knowledge & Information<br>-<br>[V/A] Planning<br>[V/B] Engaging<br>[V/B4] External Change Agents
REACH II - implemented in Scott & White Family Caregiver Program (a non-profit collaborative healthcare system)	Stevens et al. (2012) / USA	Pre-post study design (Process evaluation; Quantitative descriptive)<br><br>(IF) *RE-AIM (Reach, Effectiveness, Adoption,*	100%	P: 164 participants<br><br>O: Reach, Effectiveness, Adoption, Implementation, Maintenance	1 - Use evaluative and iterative strategies<br>2- Provide interactive assistance<br>3 - Adapt and tailor to context<br>4 - Develop stakeholder interrelationships<br>5 - Train and educate stakeholders<br>6 - Support clinicians<br>7 - Engage consumers	23, 56, 4<br>33<br>51, 63<br>52, 47, 24, 35, 6, 48, 64<br>43, 19, 71, 29<br>30, 32, 59<br>50, 39	Acceptability<br>Adoption<br>Fidelity<br>Implementation Cost<br>Penetration<br>Sustainability	[I/C] Relative Advantage<br>-<br>[II/E2] Available Resources	[I/G] Design Quality & Packaging<br>-<br>[II/B] Cosmopolitanism<br>-<br>[III/B] Network and Communications<br>[III/D2] Compatibility<br>[III/E2] Available Resources

		Implementation and Maintenance)			8 - Utilize financial strategies<br>9 - Change infrastructure	1<br>13			-<br>[V/A] Planning<br>[V/B] Engaging
Israeli NYUCI	Werner et al. (2020) / Israel	Pre-post study design (without control group)	100%	P: 54 (intervention group)<br><br>O: Implementation process and effectiveness in treating depressive symptoms	4 - Develop stakeholder interrelationships<br>5 - Train and educate stakeholders<br>8 - Utilize financial strategies<br>9 - Change infrastructure	6, 36, 57, 24<br>71, 29<br>34, 1<br>22	Appropriateness<br>Adoption<br>Penetration<br>Sustainability	[III/E2] Available Resources<br>-<br>[V/B3] External Change Agents	[I/D] Adaptability<br>-<br>[II/D] External Policy & Incentives<br>-<br>[III/B] Network and Communications<br>[III/C] Culture<br>[III/D2] Compatibility<br>[III/E3] Available Knowledge & Information<br>-<br>[V/C] Executing
		Support Interventions (e.g., Support groups) (n=5)							
Meeting Center Support Program (MCSP/MEETINGDEM)	van Haeften-van Dijk et al. (2015) / The Netherlands	Pre-post study design (Process evaluation / Qualitative)<br><br>*(IF) Adaptive implementation model*	100%	P: 40 participants (stakeholders)<br><br>O: Barriers and facilitators to implementation	1 - Use evaluative and iterative strategies<br>3 - Adapt and tailor to context<br>4 - Develop stakeholder interrelationships<br>5 - Train and educate stakeholders<br>6 - Support clinicians	4, 5, 18, 56<br>51<br>35, 36, 64, 65, 6, 52, 35, 38, 47<br>73, 19, 20, 71<br>59	Adoption<br>Feasibility<br>Penetration<br>Sustainability	[II/B] Cosmopolitanism<br>[II/D] External Policy & Incentives<br>-<br>[III/B]<br>[III/D2] Compatibility<br>[III/E1] Leadership Engagement<br>[III/E2] Available Resources<br>-<br>[V/B2] Formally Appointed Internal Implementation Leaders<br>[V/B4] External Change Agents	[I/C] Relative Advantage<br>[I/G] Design Quality & Packaging<br>-<br>[II/B] Cosmopolitanism<br>[II/D] External Incentives & Policy<br>-<br>[III/D1] Tension for Change<br>[III/E1] Leadership Engagement<br>[III/E3] Access to Knowledge & Information<br>-<br>[IV/E] Other Personal Attributes<br>-<br>[V/A] Planning<br>[V/B] Engaging<br>[V/B2] Formally Appointed Internal Implementation Leaders<br>[V/B4] External Change Agents
	van Mierlo et al. (2017) / The Netherlands	Qualitative study (semi-structured interviews); multiple case study<br><br>*(IF) Adaptive Implementation*	100%	P: (unclear)<br><br>O: barriers and facilitators to program implementation across three countries	No implementation strategies identified	n/a	Adoption<br>Penetration<br>Sustainability	[II/B] Cosmopolitanism<br>[II/D] External Policy & Incentives<br>-<br>[III/B] Network and Communications<br>[III/D1] Tension for Change<br>[III/D2] Compatibility<br>-	[I/C] Relative Advantage<br>[I/D] Adaptability<br>[I/G] Design Quality & Packaging<br>-<br>[II/A] Patient Needs & Resources<br>[II/B] Cosmopolitanism<br>[II/C] Peer Pressure

								[V/A] Planning<br>[V/B] Engaging<br>[V/B4] External Change Agents<br>[V/C] Executing	[II/D] External Policy & Incentives<br>-<br>[V/B] Engaging<br>[V/B2] Formally Appointed Internal Implementation Leaders<br>[V/B3] Champions<br>[V/B4] External Change Agents
	Mazurek et al. (2019) / Poland	Pre-post study design<br><br>*(IF) Adaptive implementation*	100%	P: 22 participants (intervention group)<br><br>O: (Un)met needs of people with mild-to-moderate dementia and their carers before and after the implementation of MCSP	1 - Use evaluative and iterative strategies<br>2- Provide interactive assistance<br>3 - Adapt and tailor to context<br>4 - Develop stakeholder interrelationships<br>5 - Train and educate stakeholders<br>7 - Engage consumers<br>8 - Utilize financial strategies<br>9 - Change infrastructure	61<br>33<br>63, 51<br>35, 57, 38, 47, 17, 52<br>43, 19, 55, 71<br>37, 39<br>34<br>13	Acceptability<br>Appropriateness<br>Feasibility<br>Penetration	[II/D] External Policy & Incentives	[I/G] Design Quality & Packaging<br>-<br>[V/C] Executing
	Meiland et al. (2005) / The Netherlands	Focus group<br><br>*(IF) Adaptive Implementation*	100%	P: 23 participants<br><br>O: barriers and facilitators to implementation of MCSP; Several factors proved to play a facilitating role in all phases of implementation, for example: motivated people, financial resources, continuous and varied PR-activities, and cooperation between organizations.	1 - Use evaluative and iterative strategies<br>3 - Adapt and tailor to context<br>4 - Develop stakeholder interrelationships<br>5 - Train and educate stakeholders<br>7 - Engage consumers<br>9 - Change infrastructure	23<br>63<br>35, 6, 52, 24, 64, 47<br>19, 55, 43<br>39<br>13	Adoption<br>Penetration<br>Sustainability	[I/C] Relative Advantage<br>-<br>[II/B] Cosmopolitanism<br>[II/D] External Policy & Incentives<br>-<br>[III/A] Structural Characteristics<br>[III/B] Network and Communications<br>[III/D1] Tension for Change<br>[III/D3] Relative Priority<br>[III/E2] Available Resources<br>-<br>[IV/A] Knowledge & Beliefs about the Intervention<br>-<br>[V/A] Planning<br>[V/B] Engaging<br>[V/C] Executing	[I/C] Relative Advantage<br>[I/G] Design Quality & Packaging<br>-<br>[II/B] Cosmopolitanism<br>[II/C] Peer Pressure<br>[II/D] External Policy & Incentives<br>-<br>[III/A] Structural Characteristics<br>[III/D2] Compatibility<br>[III/E2] Available Resources<br>-<br>[IV/D] Individual Identification with Organization<br>-<br>[V/A] Planning<br>[V/B] Engaging<br>[V/B2] Formally Appointed Internal Implementation Leaders<br>[V/C] Executing
DemenTalent	van Rijn et al. (2019) / The Netherlands	Qualitative study (semi-structured interviews); multiple case study	100%	P: 22 participants (stakeholders)<br><br>O:	1 - Use evaluative and iterative strategies<br>4 - Develop stakeholder interrelationships<br>5 - Train and educate stakeholders	5, 27, 4<br>35, 57, 6, 52<br>71	Adoption<br>Feasibility<br>Penetration<br>Sustainability	[I/G] Design Quality & Packaging<br>-<br>[II/A] Patient Needs and Resources	[II/B] Cosmopolitanism<br>[II/D] External Policy & Incentives<br>-<br>[III/D2] Compatibility

		(IF) Adaptive implementation		Barriers and facilitators to implementation; recommended modifications; Implementation strategies	7 - Engage consumers	39		[II/B] Cosmopolitanism<br>[II/D] External Policy & Initiatives`<br>-<br>[III/A] Structural Characteristics<br>[III/E1] Leadership Engagement<br>[III/E2] Available Resources<br>-<br>[V/B] Engaging<br>[V/C] Executing<br>[V/D] Reflecting & Evaluating	[III/E1] Leadership Engagement<br>[III/E2] Available Resources<br>-<br>[V/A] Planning<br>[V/B] Engaging<br>[V/B2] Formally Appointed Internal Implementation Leaders<br>[V/B4] External Change Agents<br>[V/C] Executing

Table 2. Overview of constructs found in included studies' frameworks				
**Framework**	**Intervention characteristics**	**Implementation setting**	**implementation process**	**Iterative and reflexive monitoring and (re-) evaluating strategies and outcomes**
**Leontjevas Process Evaluation Model [1]**	external/internal validity; cost-effectiveness; i. sampling quality (recruitment and randomization, barriers and facilitators, reach); ii. intervention quality (reasons for use, user friendliness, usage and relevance of intervention, Satisfaction and recommendations of intervention,	Barriers and facilitators: determinants at the level of users, determinants at the level of socio-political context	a) adherence: (end-user) adherence to protocol; implementation components delivered/received (facilitator adherence); b) barriers and facilitators to implementing: determinants for intervention use (determinants of level of innovation, users and sociopolitical context)	Decision: Consider implementation or improvement
**Multimethod Assessment Process (MAP)/Reflective Adaptive Process (RAP) [2]**	Not mentioned	Creating time and space for learning and reflection is necessary.<br><br>Improvement teams should include a variety of systems agents with different perspectives of the system and its environment, including patients.<br><br>System change requires supportive leadership that is actively involved in the change process, ensuring full participation from all members and protecting time for reflection.	a) use of an external facilitator to guide practices and describe mechanisms, b) RAP team uses iterative cycles to improve processes and to improve internal coordination (trust/communication), c) facilitators gather information and stimulate self-reflection and action; resources needed are usually unavailable so facilitator supports team	The team's skill development in group process, conflict management, meeting management, team building, and reflection-action cycles is potentially an important factor in the sustainability of new behaviors for the team and practice
**Consolidated Framework for Implementation Science Research [3]**	Intervention characteristics	Outer setting<br>Inner setting	Process domain (Planning, engaging, executing, reflecting and evaluating)	Process domain (reflecting and evaluating)
**Fixsen and Blasé Process Model [4]**	Not mentioned	staff selection, staff training, consultation and coaching, staff evaluation, administrative support, program evaluation/fidelity, and systems interventions	Exploration and adoption, program installation, Initial implementation, full operation, innovation	Sustainability; initial implementation, full operation, innovation, and sustainability. The model provides a structured way to present both formative evaluation information (progress monitoring of implementation) and summative evaluation information (findings and recommendations to decide future directions).
**Adaptive implementation model [5]**	Existing conditions that influence implementation process [characteristics of interventions]	Time/operational conditions, human/financial resources, organizational conditions<br><br>Factors influencing the a) preparation, b) execution or c) continuation phase of implementation, across three levels of the implementation setting (executive, organizational/cooperation, and health system [legislation/policy])	External factors influencing the a) preparation, b) execution or c) continuation phase of implementation, across three levels of the implementation setting (executive, organizational/cooperation, and health system [legislation/policy])	factors at the micro, meso and macro levels of the successive phases of the implementation process had facilitated or impeded the implementation
**Reach, Efficacy, Adoption, Implementation, Maintenance (RE-AIM) [6]**	Efficacy and effectiveness of intervention	Adoption by implementing organization; number and characteristics (representativeness) of settings or staff members who initiate the program/intervention.	Implementation (program protocol, fidelity, systems and organizational factors)	Maintenance refers to the extent to which the program becomes part of the routine practices of the organization or practitioners.
**Medical Research Council Framework [7]**	Intervention (description, causal assumptions & influence on implementation)	Mechanisms of outer context	[how delivery is achieved], description of content [fidelity, frequency and duration of intervention components delivered, adaptations to the intervention]	Evaluate mechanisms of implementation and influences between context and intervention mechanisms of impact.<br><br>i. Implementation then influences mechanisms of impact, or, participant responses to the intervention, as well as unexpected pathways and consequences and/or possible mediators of intervention effects.

**Lichstein's Treatment Implementation model [8]**	Not mentioned	Treatment delivery (interventionist fidelity/capabilities), treatment receipt (user fidelity), treatment enactment (treatment outcomes; behavior change)	**Guide relationship between provider-user;** TI strategies are used to facilitate and monitor activities between two actors, the interventionist and the client, so that the action of an intervention can be understood. Typically, the term treatment implementation refers to a class of strategies to document the implementation of individual treatment components	Strategies to induce and assess treatment receipt (record of contacts and system utilization, assessing caregiver knowledge of key treatment concepts and skills, interventionist documentation, feedback from caregivers)
**Normalization Process Theory [9]**	Coherence of intervention (easy to describe, distinct characteristics, clear purpose/ToC, clear benefits)	Cognitive participation (are participants willing to invest time/energy to learn and practice intervention); Collective action (how compatible intervention is to organization)	Engaging is related to cognitive participation as both describe the relational work to embed and sustain a new practice into existing workflows. Coherence work intersects with enrolling people in participating and in understanding what their tasks and contributions may be.	Reflexive monitoring (do participants believe the intervention adds value?)
**Process Evaluation model by Reelick and colleagues [10]**	Not mentioned	Quality of delivery of the interventional components (a) The part of each component and the home visits delivered by the coaches, b) Satisfaction with delivery of home visits) 2. Barriers and facilitators for delivery of interventional components (Reasons for diverging from or applying intervention components)<br><br>Experience of participants and instructors with interventional components (a) Perceived benefit, b) Strong and weak aspects of the interventional components and total intervention)	1) Reach (recruitment screening and barriers/facilitators)<br>2) Follow-up (attrition rate and barriers/facilitators)<br>3) Adherence to interventional components (barriers/facilitators)	Data acquisition<br>1. Outcome measures: coverage of interventional components (Average number of outcomes per component)<br>2. Completeness of data collection (a) Number and characteristics of missing data, b) Feasibility of outcome measures, c) Reasons why data were missing)<br>3. Barriers and facilitators for data collection (Comparison of qualitative and quantitative effectiveness data)

[1] Leontjevas R, MSc, Gerritsen DL, PhD, Koopmans RTCM, PhD, Smalbrugge M, PhD, Vernooij-Dassen MJFJ, PhD: Process Evaluation to Explore Internal and External Validity of the "Act in Case of Depression" Care Program in Nursing Homes. Journal of the American Medical Directors Association 2012, 13(5):488.e1-488.e8.

[2] Stroebel CK, McDaniel RR, Crabtree BF, Miller WL, Nutting PA, Stange KC: How Complexity Science Can Inform a Reflective Process for Improvement in Primary Care Practices. Joint Commission journal on quality and patient safety 2005, 31(8):438-446.

[3] Damschroder LJ, Aron DC, Keith RE, Kirsh SR, Alexander JA, Lowery JC: Fostering implementation of health services research findings into practice: a consolidated framework for advancing implementation science. Implementation Science : IS 2009, 4(1):50.

[4] Fixsen DL, Blase KA, Naoom SF, Wallace F: Core Implementation Components. Research on social work practice 2009, 19(5):531-540.

[5] Meiland FJM, Dröes R, Lange Jd, Vernooij-Dassen MJFJ: Facilitators and barriers in the implementation of the meeting centres model for people with dementia and their carers. Health policy (Amsterdam) 2005, 71(2):243-253.

[6] Glasgow RE, Vogt TM, Boles SM: Evaluating the public health impact of health promotion interventions: the RE-AIM framework. American journal of public health (1971) 1999, 89(9):1322-1327.

[7] Campbell M, Fitzpatrick R, Haines A, Kinmonth AL, Sandercock P, Spiegelhalter D, Tyrer P: Framework for design and evaluation of complex interventions to improve health. BMJ 2000, 321(7262):694-696.

[8] Lichstein KL, Riedel BW, Grieve R: Fair tests of clinical trials: A treatment implementation model. Advances in behaviour research and therapy 1994, 16(1):1-29.

[9] Murray E, Treweek S, Pope C, MacFarlane A, Ballini L, Dowrick C, Finch T, Kennedy A, Mair F, O'Donnell C, Ong BN, Rapley T, Rogers A, May C: Normalisation process theory: a framework for developing, evaluating and implementing complex interventions. BMC Medicine 2010, 8(1):63.

[10] Reelick MF, MSc, Faes, Miriam C., MD, MSc, Esselink, Rianne A.J., MD, PhD, Kessels RPC, PhD, Olde Rikkert, Marcel G.M., MD, PhD: How to Perform a Preplanned Process Evaluation for Complex Interventions in Geriatric Medicine: Exemplified With the Process Evaluation of a Complex Falls-Prevention Program for Community-Dwelling Frail Older Fallers. Journal of the American Medical Directors Association 2011, 12(5):331-336.

Table 3. Barriers to Implementation			
CFIR Domain and Constructs	**Frequency in 67 studies**	**Details**	**Studies that identified this theme as a barrier**
[I] Intervention Characteristics			
[I/A] Intervention Source	0/67; 0%		
[I/B] Evidence Strength & Quality	1/67; 1.49%	1- Carers received the wrong information via the intervention and there were concerns about the quality of the residential respite program.	1- Bruce and Patterson (2000)
[I/C] Relative Advantage	4/67; 5.97%	1- Existing **usual care is already sufficient** so there is no need for a substitute.<br>2- **Market saturation** due to increasing number of initiatives concerning support (e.g., meeting centres).<br>3- **Missing components that were valued in original intervention; need to adhere to original intervention -** Scott & White Family Resource Center did not provide home visits **due to resource limitations** whereas REACH II (original) did.	1- Bass et al. (2003), Paone (2014)<br>2- Meiland et al. (2005)<br>3- Stevens et al. (2012)
[I/D] Adaptability	12/67; 17.91%	1- **Delivery modality:** Intervention should be delivered more conveniently via mobile application or web-based platform, compatible with mobile phones to enhance wide usability; **adaptable user experience (e.g. adaptable font type and size, language options)**<br>**2-** **Lack of tailored, individualized support:** Intervention was not appropriate to user groups (e.g. exercises prescribed should be allocated based on stage of disease and with co-morbidities in mind);<br>**3-** **Lack of location/setting adaptability:** Home visits were difficult to arrange<br>**4-** **Need for homogenous grouping of participants:** Caregivers should be grouped according to appropriate age groups and/or based on PwD stage of disease progression.<br>**5-** Lack of support for **technological illiteracy**<br>**6-** **Lack of time flexibility and patient-centered care provision –** Need for continued care beyond scheduled visits; rigid scheduling that do not fit dyads' schedules and/or are overwhelming for either care partner.<br>**7-** Interventions should provide **culturally adapted, context-specific resources (e.g. local resources, legal information, guidance on practical support)**<br>**8-** **Need for human facilitator**	1- Baruah et al. (2020), Burgio et al. (2009), Dam et al. (2019), Glueckauf and Loomis (2003), Xiao et al. (2020)<br>2- Barrado-Martin et al. (2019), Baruah et al. (2020)<br>3- Barrado-Martin et al. (2019),<br>4- Boots et al. (2017), Kovaleva et al. (2017),<br>5- Kovaleva et al. (2017)<br>6- Burgio et al. (2009), Judge et al. (2010)<br>7- Baruah et al. (2020), Pagan-Ortiz et al. (2014), Teles et al. (2020)<br>8- Dam et al. (2019)
[I/E] Trialability	0/67; 0%		
[I/F] Complexity	2/67; 2.99%	1- **Intervention components (REACH, Family Memory Care) too complex to implement**, forcing implementation sites to extend their intervention phase for over 12 months. **Staff required more time and skills than originally anticipated** by organizations or state. Consultants needed knowledge and skills in caregiver assessment, care planning, family systems and group facilitation, communication strategies, understanding Alzheimer's disease symptoms, accessing local resources, and providing guidance on options for addressing issues at home.	1- Burgio et al. (2001), Paone (2014)
[I/G] Design Quality & Packaging	25/67; 37.31%	1- **User Experience** – Visual issues/readability (manual content need bigger font; content should be suitable for lower reading levels); language should be clear, simple, plain and easy to understand. **Improvements may include** a) step-by-step manual at first login to help members use application, better user-friendly chat function & videos/animations to access professionals and other caregivers, c) reduce number of log-ins required, d) bigger navigational buttons on internet platforms, e) incorporate reminder functions, f) incorporate user-friendly keywords for searching, g) provide group-learning modules that suit caregiver needs, h) provide auto-save function, i) allow open-ended responses since some answers do not fall into given categories, j) add an 'Ask an Expert' section for direct feedback<br>2- **Technical difficulties (infrastructure and user illiteracy) - poor audio quality** for headset users (two-way chat); **slow connectivity speed** (e.g. sporadic buffering, poor audio and visual quality of user submitted videos due to user technological illiteracy; users encounter	1- Burgio et al. (2009), Orsulic-Jeras et al. (2016), Baruah et al. (2020), Teles et al. (2020), van Knippenberg et al. (2017), Boots et al. (2017), Dam et al. (2019), Glueckauf and Loomis (2003), Teles et al. (2020), Xiao et al. (2020), Pagan-Ortiz et al. (2014)

		difficulties with computer software and firewalls, different variations of internet browsers, general equipment failure, empty battery, defective touch screen.<br>3- **Content delivery format** - Reading was preferred over audio to save time; short videos with voice-over explanations may be quicker for informal caregivers than reading information. Physical printed books were also preferred (in addition to digital).<br>**4- Service fragmentation & poorly integrated care –** Need for close involvement of partners that facilitate components of the intervention.<br>**5- Intervention programming (respite care) –** Inadequate nutrition, lack of exercise & stimulating activity, failure to produce AD-specific programming, lack of accommodation for PwD demands<br>**6- Presentation of intervention –** Some user-identified deterrents include the name of program (DemenTalent), unclear marketing tools/brochures (program components and capacity of program),<br>**7- Lack of content and sufficient information for users –** Need for additional resources (e.g. how to discuss end-of-life issues with caregivers); content needs to be more **diverse (e.g. more evidence-based & derived from lived experiences (e.g. complex real-experience vignettes)), and suitable for context and for specific stages of disease progression**.<br>**8- More sessions for programs (or embedded into usual care for long-term use) with shorter duration per session –** attrition occurred due to poor fit and/or duration of intervention and length of each session.<br>**9- Intervention components may not be best suited for current business model (need to find solutions to minimize revenue loss)** *["The first involved balancing training needs with limited therapist time. Although ESP builds upon OT foundational knowledge and skills, it is not traditional practice and requires training in its assessments, protocols, and treatment principles. As agency revenue and therapist salaries are based on patient contact, we needed to develop a training approach that minimized revenue loss"*<br>**10- Class-based exercises** faced transportation and logistic issues & home-based exercises were guided with a booklet that had unclear photos (e.g. booklet images portrayed exercise movements); some content was repetitive & components were not used as intended (e.g. action plan, coping plan).	2- Banbury et al. (2019), Glueckauf and Loomis (2003), van Knipenberg et al. (2017), Williams et al. (2020)<br>3- Pagan-Ortiz et al. (2014), Xiao et al. (2020), Boots et al. (2017), Dam et al. (2019)<br>4- Bruce and Patterson (2000)<br>5- Beisecker et al. (1996)<br>6- van Rijn et al. (2019)<br>7- Chang et al. (2010), Kovaleva et al. (2017), Milne et al. (2014)<br>8- Sommerlad et al. (2014), Kovaleva et al. (2017), Pot et al. (2015), Teles et al. (2020), van Knippenberg et al. (2017)<br>9- Gitlin et al. (2010)<br>10- Barrado-Martin et al. (2019), Barrado-Martin et al. 92020), Prick et al. (2014)
[I/H] Cost	4/67; 5.97%	1- Cost of intervention was sensitive to caseload & most costs were to cover **salary of consultants**<br>2- Cost to use intervention was mentioned as a barrier to use; 60.7% of respondents reported the daily cost of service should be less than US$11 per day.<br>3- **Respite care vouchers were provided to support costs but they are inconsistent**. Lack of financial security increases stress among users.	1- Paone (2014)<br>2- Brandao et al. (2016), Beisecker et al. (1996)<br>**3-** Roberts and Struckmeyer (2017)
<u>[II] Outer Setting</u>			
[II/A] Patient Needs & Resources	**24/67; 35.82%**	**1- Sociocultural taboo** surrounding dementia discourages seeking a diagnosis and care options outside of the immediate support circles;<br>**2- Inconvenience** (logistical, transportation issues, scheduling, internet connectivity, technological illiteracy, need for training for caregivers to adopt intervention) accessing/utilizing services; lack of time for program due to caregiving responsibilities also a factor.<br>3- Caregivers **did not determine there was a need for external support** or were **not informed about the availability of the programs** (e.g. organizations are unaware of service fragmentation due to lack of referral between GP & care coordination/case management programs and the lack of **information** available)<br>4- **Financial burden** (e.g. self-financed care, cost of wheelchairs)<br>5- **Caregivers' personal barriers (e.g. time constraints, emotional readiness)** and **need for individualized support** (recommend for a social worker to assess the caregivers' availability and intervention suitability individually (case-by-case)).<br>6- **Misalignment between intervention and patient needs/capabilities** (e.g. caregivers do not have time to use other programs due to usual care) – *["In the Netherlands, caregivers also have support from usual care, provided by respite care (day care facilities), home visits from a case manager, which leaves little free time for other programs (e.g. exercise)."]* - <u>Consider region-specific contextual conditions to determine the burden-benefit ratio of an intervention in certain contexts</u><br>7- **Poor timing of intervention** (e.g. introducing Occupational therapy immediately after diagnosis may confused caregivers OR introducing an intervention/component too late will be less effective, such as monitoring)	1- Van Rijn et al. (2019), Brandao et al. (2016)<br>2- Beisecker et al. 91996), Gaugler (2014), Banbury et al. (2019), Baruah et al. (2020), Meichsner et al. (2019), Pot et al. (2015), Xiao et al. (2020), Mitchell et al. (2017), Xiao et al. (2020)<br>3- Brandao et al. (2016), Laparidou et al. (2018), Bruce and Patterson (2000), Smith and Graves (2020), Baruah et al. (2020), Boots et al. (2017)<br>4- Laparidou et al. (2018), Paone (2014)<br>5- Gaugler et al. (2018), Orsulic-Jeras et al. (2016), Meyer et al. (2018),<br>6- Sommerlad et al. (2014), Barrado-Martin et al. (2019), Prick et al. (2014)

			**7-** Field et al. (2019), Boots et al. (2017), Mitchell et al. (2017), van Knippenberg et al. (2017)
[II/B] Cosmopolitanism	**14/67; 20.90%**	**1- Complexity resulting from vast network/size of implementing agency** (e.g. *Veteran Affairs' (USA) size, number of facilities, and large catchment areas add a complexity that smaller agencies would not have. Training, certification, and caregiver interactions would be on-site or more likely to be local, rather than national or regional.)*<br>**2- Difficulties creating and maintaining partnerships (across multiple levels) beyond an established network (e.g. ageing network) due to different interests/agendas & fit/misalignment** *("culture of care organisations and welfare organizations were so different that collaboration remained difficult and the process was time consuming (NL)") – NL Care, welfare and municipal organisations are not "on the same page"*<br>**3- Lack of coordination/referral between GP (e.g. hospital/Alzheimer's unit) and community-based services** due to lack of dementia diagnosis, failing to realize extent of carers' problems, GPs unawareness of available support options (e.g. ACAT as an assessment agency for community care), carers' own shame and concealment. GPs also have high workload so involvement/relationships are difficult to maintain.<br>***4-*** **Difficulty involving/collaborating with physicians/GPs in participation (e.g. they act as gatekeepers to target participants) –** physicians **should be included in all phases of outreach activities** including community assessment and planning phase so their input can be maximized and potential barriers can be addressed. *GPs (or referrers) are unfamiliar with differences between meeting centres, and day care, and psychogeriatric treatment.*<br>5- **Project initiator's position within the region determines the success of dissemination** (e.g. if he/she belongs to local organization or regional/provincial; reputation of initiator) - *If the initiating organization has a bad reputation or positioning with referrers (GP/Case manager), then this impedes the cooperation among organizations not convinced of surplus value*<br>6- **Absence of regional (and cross sectoral) partners impedes uptake and reduces publicity/visibility of programs** *(e.g. limited partnership involvement between Alzheimer's Association Chapter and Area Agencies on Aging (AAA) impeded the referral process)*	1- Nichols et al. (2016),<br>2- Samia et al. (2014), Meiland et al. (2005), van Haeften-van Dijk et al. (2015),<br>3- Laparidou et al. (2018), Bruce and Patterson (2000), van Mierlo et al. (2017), van Rijn et al. (2019)<br>4- Connell and Kole (1999), van Mierlo et al. (2017), Meiland et al. (2005)<br>5- Meiland et al. (2005), van Haeften – van Dijk et al. (2015)<br>**6-** Van Haeften- van Dijk et al. (2015), McCurry et al. (2015)
[II/C] Peer Pressure	**0/67; 0%**		
[II/D] External Policy & Incentives	**15/67; 22.39%**	**1- Widespread infrastructure shortage –** Poor internet acceptance across layers (age/computer literacy) of society; attitudes toward interventions and educational methods/formats remain rigid despite policy guidelines;<br>**2- Health system infrastructure** is unable to adopt new innovations *(e.g. Poland lacks a multidisciplinary, coordinated system of care for people with dementia in the form of an evidence-based structured pathway. The post-diagnostic support is very limited. There are no services to support carers emotionally, socially, or practically during the dementia journey.)*<br>**3- Insufficient incentive to adopt in traditional healthcare system (e.g. hospitals) –** *new innovations cannot be streamlined into clinical practice because providers do not have sufficient time nor do they prioritize dementia care; FamTechCare (eHealth) is more feasible outside of the traditional healthcare system and more as a community-based resource that does not operate within the healthcare system.*<br>**4- Insufficient resource allocation/funding and payment schemes for community-based care –** Community-level resources are constrained by limited public funds.<br>**5- Existing health financing mechanisms/coverage not comprehensive of caregiver needs –**<br>→ **(USA)** PwD do not have Medicare and cannot afford co-pay *(e.g. caregiver training must be linked to treatment goals but patients were not referred to [occupational therapy] for dementia but rather other comorbid chronic conditions contributing to functional decline. **Referral for OT for dementia diagnosis should not be denied**.)*<br>→ **(USA)** Previous government care coverage mandates are not comprehensive and does not cover **caregiver** support for REACH VA *(The Congressionally mandated of caregiver support for caregivers of seriously injured post 9/11 veterans who were receiving stipends had **separate rules and regulations that did not necessarily apply to caregivers who were receiving REACH VA services or other non-mandated services**.)*<br>→ **(NL) AWBZ (**The Exceptional Medical Expenses Act, 2003) resulted in consequences for the financing of meeting centres (e.g. only various functions/types of support qualify for reimbursement). Meeting centres will be paid on the **basis of performed services**, which means only days participants join are reimbursed, despite staff numbers remain consistent regardless of attendance.	1- Dam et al. (2019), Levinson et al. (2020),<br>2- Mazurek et al. (2019)<br>3- Williams et al. (2020),<br>4- Lykens et al. (2014)<br>5- Gitlin et al. (2010), Meiland et al. (2005), van Mierlo et al. (2017), Nichols et al. (2011), van Rijn et al. (2019), van Haeften – van Dijk et al. (2015)<br>6- Smith and Graves (2020)<br>**7-** Samia et al. (2014), van Rijn et al. (2019)

		→ **(NL) Fragmented funding –** Patients may need to **reapply for additional aid** from a different funding Act; fragmentation of funding impedes implementation process & is complicated/time consuming. Also, there is no financing for people without a formal diagnosis of dementia (which presents a diagnostic barrier to care).<br>→ **(UK) Financing mechanisms unclear & difficult to know where to secure structural funding from**<br>**6- Lack of use of community support services** (e.g. (USA) 62% of participants reported using no community support services, such as Meals on Wheels)<br>**7- Macro-level influences - Macro-level events** (e.g. economic downturn) influences funding and program demand; **changes in the political parties** may alter funding mechanisms for government funded community-based care (creating volatile funding and anxiety).	
[III] Inner Setting			
[III/A] Structural Characteristics	**2/67; 2.99%**	1- Rigid organizational structure presents adoption/development of program *(e.g. being trapped in the rules of the organization; hierarchal organizations with limited freedom & no room for developing new programs)*<br>2- **Financing mechanisms within the organization** – *welfare organizations often work with budgets for more than one year which makes it difficult for projects to be financed in the interim*.	1- Van Rijn et al. (2019)<br>2- Meiland et al. (2005)
[III/B] Network and Communications	**2/67; 2.99%**	1- Lack of clarity between staff members regarding responsibilities (poor information transfer) - The programme coordinators experienced the fact that potential referrers sometimes had little time to be informed, and **that information is not adequately distributed among other workers within organizations**	1- Van Haeften- van Dijk et al. (2015), Meiland et al. (2005)
[III/C] Culture	**0/67; 0%**		
[III/D] Implementation Climate		1- **Need to adapt intervention to fit the agency (that serves diverse communities)** while maintaining fidelity – Intervention in clinical trial not feasible for use in the AAA; case managers/supervisors were near saturation with services already being provided. Interventions need to be modified significantly while maintaining effectiveness. However, controlled trial methodology fidelity checks (rating audio-taped sessions and direct observation) within agencies were not realistic due to inefficient costs or poor fit with agency culture.	1- Burgio et al. (2009), Mittelman and Bartel (2014), Gitlin et al. (2010),
[III/D1] Tension for Change	**5/67; 7.46%**	**1- Staff/clinicians reluctant to adopt new interventions** due to high caseload & existing repertoire/usual care (difficult to convince care providers to deliver new program due to organization rigidity and workload)<br>2- Implementing into hospital systems is difficult since interventionists are viewed as outsiders and competitors rather than collaborators in a mutually beneficial development process<br>3- Resistance from existing end-users at the agencies<br>4- Lack of awareness of intervention within the organization	1- Nichols et al. (2016), McCurry et al. (2015), Meiland et al. (2005)<br>2- Connell and Kole (1999)<br>3- Van Mierlo et al. (2017)<br>**4-** Van Mierlo et al. (2017)
[III/D2] Compatibility	**7/67; 10.45%**	**1- Misalignment between support program components and end-users/agency** (e.g. day care users are usually passive and not interested in activities; nursing homes faced difficulties implementing outpatient care services in more inpatient-oriented organisation)<br>**2-** Practical operational challenges impede implementation (e.g. how to staff, supervise and finance a new service while continuing usual care); implementation sites (e.g. home care agencies, clinics, hospital based agencies) may be ill-equipped to adopt new interventions (e.g. Family Memory Care Program) as they were new to providing caregiver support.<br>→ Existing financing mechanisms do not suffice to cover new interventions: *These organizations provided medical services to patients and were* ***accustomed to receiving reimbursement for services rendered through patients' insurance plans****. These medically-focused facilities* ***had little prior experience with caregiver services****. The* ***unbillable world*** *of caregiver support and aging services* ***through grant programs*** *was unfamiliar territory.* ***These organizations struggled with how to incorporate FMC into their organizations****. (…) For example, one hospital that was participating as a host site organization moved the FMC Consultant from one department to another three times in the course of 18 months, trying to find the right fit for the program within its operational structure. Another home-care/clinic organization found it hard to designate time of a key staff person (a nurse) to the program and eventually dropped out. (USA)*<br>3- Agencies' understanding of end-users may not reflect reality (e.g. how technologically literate users are)<br>4- **Comparable to usual care:** Hesitancy to share new interventions due to a similar product already on the market (e.g. Functions of InLife can be fulfilled by WhatsApp); *local coordinator was concerned that the project did not duplicate services/programs already implemented by local chapter*	1- Van Mierlo et al. (2017), van Haeften- van Dijk et al. (2015)<br>2- Mittelman and Bartels 2014), Paone (2014)<br>3- Boots et al. (2017)<br>4- Dam et al. (2019), Connell and Kole (1999)
[III/D3] Relative Priority	**2/67; 2.99%**	1- **Physicians prioritize delivering care & not innovation -** Gaining commitment for program was challenging due to the composition of advisory group & their demanding schedules as physicians	1- Connell and Kole (1999)<br>2- Meiland et al. (2005)

		2- **Agencies are most likely to cut back on latest implemented programs, thus risking continuation** (reducing innovations on the list of priorities)	
[III/D4] Organizational Incentives & Rewards	**0/67; 0%**		
[III/D5] Goals and Feedback	**0/67; 0%**		
[III/D6] Learning Culture	**0/67; 0%**		
[III/E] Readiness for Implementation			
[III/E1] Leadership Engagement	**4/67; 5.97%**	**1- Ambiguity around who is responsible for managing the project (poor management/organization) -** *Manager: lack of pioneer spirit and creativity to think outside borders of own institution. Absence of programme coordinator or team manager to support staff.*<br>**2- Leadership contracts did not include enough hours fully focus on the implementation of the project**<br>**3- Lack of leadership involvement:** Project coordinator of the museum did not take part in the training & Advisory boards were not involved in the organizational/community development aspects of the project.	1- Van Haeften- van Dijk et al. (2015)<br>2- Van Rijn et al. (2019)<br>3- Hendriks et al. (2018), Connell and Kole (1999)
[III/E2] Available Resources	**15/67; 22.39%**	**1- Location to deliver intervention: cleanliness, inadequate space, unpleasant atmosphere, unsafe environment and unsuitable furniture were perceived as barriers**<br>**2- Finances -** No natural source of funding for program; financial cutbacks that prevent the adoption of new tools or inter-agency collaboration. Project leaders need to obtain funds to start up intervention, which was time consuming and most leaders were untrained for it. (e.g. Funding mechanisms (i.e. grants) are given for more than a year so programs cannot be financed in the interim)<br>**3- Insufficient time commitments or human resources (e.g. due to staff turnover) to facilitate training or perform other functions (e.g. case management tasks, organize additional tasks). Consultants struggled with the time the program demanded** (e.g. training, ongoing mentoring, documentation requirements, skills relating to working with families and addressing serious issues of caregivers)<br>**4- Logistical issues with training clinical counselors and new hires and recruiting participants**<br>**5- Success depends on availability of resources and services, such as equipment provision or a therapeutic group.**	1- Connell and Kole (1999), Stevens et al. (2012), Beisecker et al. (1996)<br>2- Paone (2014), Boots et al. (2017), van Haeften – van Dijk et al. (2015), van Rijn et al. (2019), Meiland et al. (2005)<br>3- Burgio et al. (2001), Nichols et al. (2011), Paone (2014), Kovaleva et al. (2017), Meiland et al. (2005), van Haeften – van Dijk et al. (2015), Beisecker et al. (1996)<br>4- Werner et al. (2020), Nichols et al. (2011)<br>5- Burgess et al. (2020)
[III/E3] Access to Knowledge & Information	**5/67; 7.46%**	1- **Training resources (unaware of caregiver needs) or inadequate training for staff members.**<br>2- **No implementation manual**<br>3- **Transparency for staff to know the budget plan beforehand to anticipate costs involved**<br>4- **Access to case files (i.e. patient with dementia at all stages) and information on caregivers**	1- Laparidou et al. (2018), Beisecker et al. (1996)<br>2- Mittelman and Bartels (2014)<br>3- Hendriks et al. (2018)<br>4- Boots et al. (2017)
[IV] Characteristics of Individuals			
[IV/A] Knowledge & Beliefs about the Intervention	**5/67; 7.46%**	1- Participants felt skeptical about the intervention or felt they did not need it.<br>2- Managers felt skeptical and resistant toward the intervention if the region was generally conservative or if people preferred to develop their own concepts (e.g. do their own doctoring). *The problem is that everybody then starts reinventing the wheel, and that is not nearly as successful as implementing a proven concept*<br>3- End-users had privacy concerns for eHealth interventions	1- Barrado-Martin et al. (2019), Paone (2014), Dam et al. (2019)<br>2- Meiland et al. (2005)<br>3- Dam et al. (2019), Mitchell et al. (2017)
[IV/B] Self-efficacy	**3/67; 4.48%**	1- Caregivers lacked confidence in their ability to guide PwD (and execute the exercises at home via exercise recordings/photos) and discontinued engagement in practice	1- Barrado-Martin et al. (2019), Barrado-Martin et al. (2020)
[IV/C] Individual State of Change	**0/67; 0%**		

[IV/D] Individual Identification with Organization	**2/67; 2.99%**	**1-** Misalignment between staff members' perception of their roles within the organizations' mission and the organizations' strategy (e.g. marketing strategy) *(e.g. The need to adopt a more business-like approach in marketing and managing ADS programs was also mentioned in interviews with staff, but this also contrasted with how some direct care staff viewed themselves and their mission)*<br>**2- Primary implementation agent are decision-makers and determine the longevity of intervention** (which may deter implementation); *physicians in independent practices may not strongly identify with their affiliated health organization (e.g. hospital) but successful implementation relies on physicians.*	1- Gaugler (2014)<br>2- Levinson et al. (2020)
[IV/E] Other Personal Attributes	**15/67; 22.39%**	1- Dynamic between dyad determines intervention outcomes (e.g. less positive relationship makes intervention difficult)<br>2- **Too busy and distracted by external variables:** Caregivers felt busy with usual care and caregiving responsibilities and had no time for intervention.<br>3- **Physical complaints from caregivers & PwD being unable to complete the tasks set (e.**g. exercise regimen)<br>4- **Staff competencies:** Staff (e.g. coaches) may not be aware of memory concerns or be able to manage memory concerns; the staff efficacy directly determines the success of an intervention, which may be difficult as they are required to take on multiple roles. Some staff may be ill-equipped to respond to co-morbidities if it is beyond the scope of the intervention. *(Interventionists should be trained to locate appropriate referral sources, make referrals and follow-up to encourage continuity of care)*<br>5- **Socio-**cultural context determines receptivity of caregivers/end-users - *Nontraditional familial structures (e.g. re-marrying followed by dementia diagnosis creating a shift in roles and dynamics), caregiver isolation and guilt for using respite services (leading to self-doubt as a CG), and financial impact from CG responsibilities*<br>→ Personal beliefs about dementia also influence the use of services; lack of awareness of symptoms may be undermined as "part of ageing"<br>→ Mistrust toward government/government-related institutions (cultural)<br>6- End users and technological literacy determines the outcome of the intervention program.	1- Burgess et al. (2020)<br>2- Field et al. (2019), Barrado-Martin et al. (2020), Burgio et al. (2001)<br>3- Prick et al. (2014)<br>4- Martindale-Adam et al. (2017), McCurry et al. (2015), Burgio et al. (2001)<br>5- Roberts and Struckmeyer (2017), Meyer et al. (2018), Paone (2014)<br>6- Baruah et al. (2020), Dam et al. (2019), Xiao et al. (2020), Williams et al. (2020)
[V] Process			
[V/A] Planning	**6/67; 8.96%**	1- Ensure suitable fidelity monitoring mechanisms are implemented *(although the use of induction and assessment techniques is essential, the specific techniques selected can have profound implications for the feasibility of implementing the intervention in the real world. For example, videotaping client behavior in the home, although an excellent enactment assessment strategy, may be too intrusive for some types of interventions and may even undermine intervention goals)*<br>2- **Implementation manual was not available** prior to implementation (execution phase).<br>3- Tension between adapting and maintain fidelity to tested protocol; translational steps are needed to deliver interventions in the community (and other) settings including a) bring together interdisciplinary teams to integrate basic science and treatment approaches and b) translate evidence-based treatments into service delivery settings.<br>4- Weather disruptions in Upper Peninsula posed a barrier to planning meetings<br>5- Mistrust within team (e.g. inner reluctance to adopt program due to mistrust of collaborators during strategizing/planning phase)<br>6- Logistical planning was difficult due to scheduling conflicts with participants<br>7- Need for systemic approach and careful plan consultation with stakeholders during development phase (co-creation) & a collaboration protocol should be established to help sustainment.<br>8- Need to invest time in **familiarizing with the environment & care/welfare organisations/networks to create support**; not investing time will impede implementation<br>9- Select appropriate location (e.g. implementing in agency with usual care may have high existing operating costs, an unsuitable atmosphere (e.g. safety issues) or not accept PwD. Relocating creates barriers to access – moving to a more distant location may force users to relocate and create too many hurdles to access.	1- Burgio et al. (2001)<br>2- Mittelman and Bartels (2014)<br>3- Paone (2014)<br>4- Connell and Kole (1999)<br>5- Connell and Kole (1999)<br>6- Baruah et al. (2020)<br>7- Xiao et al. (2020), van Mierlo et al. (2017)<br>8- Meiland et al. (2005)<br>9- Meiland et al. (2005)
[V/B] Engaging	**13/67; 19.40%**	1- **Ineffective recruitment strategies –** a) advertising in national/local newspapers and geriatric websites, b) sending personal letters ot caregivers of people with dementia via caregiver organisations, c) promotions efforts were only at the local program site level & had difficulties reaching people (leading to fewer participants), d) marketing and outreach required more effort than planned & most caregivers needed multiple engagement before enrolling, e) lack of paid advertisements and active dissemination to develop familiarity and interest in the program.	1- McCurry et al. (2015), Prick et al. (2014), Hendriks et al. (2018), Paone (2014), Levinson et al. (2020), van Mierlo et al. (2017)

		→ Multi-modal engagement strategy is needed; targeting organisations, clinicians, trainees, and family caregivers. However, **costs required to sustain this (e.g. marketing, personnel, cost of time for champions)** was a major barrier.<br>**2- Participants (e.g. caregivers) refused to participate** because they felt they did not take on the role or there was no need<br>→ *Recruitment was difficult, and there was little awareness about the program (Meeting Centres) (UK); convincing people with dementia and their caregivers to attend was difficult since it was a big step to accept a formal diagnosis of dementia*<br>→ *Taboo, referral of people with more advanced stages of dementia, and unexpected decline in cognitive abilities of participants led to fewer volunteers signing up or people dropping out within several months.*<br>→ *Rigidity of program reduced client engagement*<br>**3- Continuing medical education programs helped disseminate information to large audience but were ineffective in long-term change at community level.**<br>**4- Referral partnerships were difficult to establish or were not reliable (possibly due to lack of knowledge about intervention); dissemination of programs was impeded if initiator did not belong to a regional/provincial organization. Program coordinators also noted that referrers had little time to be informed and information is not adequately distributed among other workers within cognizations.**<br>→ *Lack of welfare organization in region and lack of PR activities in neighborhood were also barriers.*<br>**5- Enrollment process was lengthier and more complex than anticipated or experienced in RCTs.**<br>**6- PwD lived alone or was not registered with a partner**<br>**7- Caregiver did not own a computer (eHealth) or experienced technological challenges with finding the program (e.g. international search engines)**	2- Gitlin et al. (2010), Paone (2014), van Mierlo et al. (2017), van Rijn et al. (2019), Gaugler (2014)<br>3- Connell and Kole (1999)<br>4- Hendriks et al. (2018), Meiland et al. (2005), van Mierlo et al. (2017)<br>5- Paone (2014)<br>6- Van Knippenberg et al. (2017)<br>7- Boots et al. (2017), Pagan-Ortiz et al. (2014)
[V/B1] Opinion Leaders	**0/67; 0%**		
[V/B2] Formally Appointed Internal Implementation Leaders	**2/67; 2.99%**	**1- Implementation leaders quit the program amid execution phase & program coordinator did not take part of the trainings (for volunteer guides)**<br>**2- Budget was insufficient to appoint a project leader**	1- Hendriks et al. (2018)<br>2- Van Haeften- van Dijk et al. (2015)
[V/B3] Champions	**3/67; 4.48%**	**1- Time and effort spent on recruitment was challenging for champions & trainer attrition was an issue to sustain the program.**<br>**2- Counselors (who undergone 2-day courses) were hindered from participation due to scheduling conflicts; care providers did not feel properly compensated/reimbursed for their efforts.**	1- Paone (2014), Samia et al. (2014)<br>2- Werner et al. (2020)
[V/B4] External Change Agents	**2/67; 2.99%**	1- Italy – National associations (e.g. Alzheimer's Association) strongly supported the project but the collaboration with these organizations was **difficult as these supporters would participate in the initiative group, but the execution phase was not supported.**<br>2- Referrers (e.g. GP) lack up-to-date information about support program and target group so it is **difficult to cooperate with external agents**	1- Van Mierlo et al. (2017)<br>2- Van Haeften- van Dijk et al. (2015)
[V/C] Executing	**7/67; 10.45%**	**1- Translation of programs were labor intensive and required integrated activities that involved key stakeholders from research and agency sites.**<br>**2- Italy and Poland MCSP – Waitlist was established since interested registrants exceeded the program capacity;** *participants often deteriorated so they no longer met the criteria by the time there was room available.*<br>**3- Unexpected changes in the organization** – MCSP underwent unexpected organizational change during starting phase of project (e.g. change in management and location); nursing strike disrupted training session<br>**4- High attrition rate from users -** *["Almost 25 percent of enrollees discontinued counseling before completing the minimum four counseling sessions, and 54.8 percent discontinued counseling before completing all six sessions. This contrasts with the completion rates in the initial randomized controlled trial of 98.3 percent"] ["it is possible that the intervention is less likely to be fully implemented when provided outside of the context of a controlled research study."]*	1- Gitlin et al. (2010)<br>2- Van Mierlo et al. (2017), Meiland et al. (2005)<br>3- Van Rijn et al. (2019), Connell and Kole (1999)<br>4- Pot et al. (2015), Mittelman and Bartels (2014)
[V/D] Reflecting & Evaluating	**3/67; 4.48%**	**1- Real world evidence differs from RCT result –** *12-in home sessions for caregivers is difficult to facilitate and limited the number of families who were exposed to and benefited from the program*<br>**2- Real costs were calculated with non-completers; later costs per participant rose year to year due to attrition.**<br>**3- Few meeting centres reported the lack of users as an influential factor that had them reconsider continuing the program (e.g. large financial investment for few people).**	1- Nichols et al. (2016)<br>2- Paone (2014)<br>3- Van Rijn et al. (2019)

Table 4. Facilitators to Implementation			
CFIR Domain and Constructs	Frequency reported as a facilitator in 67 studies	Details	Studies that identified this theme as a facilitator
[I] Intervention Characteristics			
**[I/A] Intervention Source**	0/67; 0%		
**[I/B] Evidence Strength & Quality**	0/67; 0%		
**[I/C] Relative Advantage**	10/67; 14.93%	1- Culturally inclusive interventions are based on the users (e.g. "interdependence and collectivistic values of Vietnamese [culture]")<br>2- More comprehensive than traditional approaches & more robust than usual care; [respite care] had more positive atmosphere compared to nursing homes due to staff training and care routines. Small, permanent team of professionals was an asset. [support care] traditional nursing home-based day care are located in a specialized unit of nursing home where they offer social, psychological and paramedical services (w/ social activities, reminiscence, music therapy, etc.)<br>3- Intervention (e.g. care consultation) may decrease depression by tending to needs of caregivers whereas usual care only focuses on patient (PwD) needs.<br>4- Patient-centred: Flexibility offered due to more suitable opening times (e.g. 9-5, traditional business hours); families can create their own attendance schedule. [Meeting centres compared to institutionalized care] format of care is more accessible and aligned with user needs. [eHealth/iGeriCare] increased ease to access dementia education.<br>5- Geospatial proximity/location was advantageous (e.g. community centres and centres for the elderly); activities were also conducted in the same building or neighborhood to promote networking and socializing.<br>6- Fills service fragmentation gaps – delays in transfer from MCSP to other facilities (e.g. day care) occur so users prefer to stay at Meeting Centres. Combined packaging of support groups and counselling reduces fragmentation by providing relevant information to caregivers that seek additional assistance (e.g. emotional support, functional support)	1- Meyer et al. (2018)<br>2- Paone (2014), Gaugler (2014), Meiland et al. (2005), van Haeften – van Dijk et al. (2015)<br>3- Bass et al. (2003)<br>4- Roberts and Struckmeyer (2017), Meiland et al. (2005), Levinson et al. (2020)<br>5- Meiland et al. (2005), van Mierlo et al. (2017), van Haeften – van Dijk et al. (2015)<br>**6-** Van Mierlo et al. (2017), Gaugler (2018)
**[I/D] Adaptability**	19/67; 28.36%	1- Adapting intervention (e.g. to fit education level or cultural groups) to local participants to promote bigger engagement and positive atmosphere to help users and therapists provide care through local evaluation and/or co-creation; care plan developed through the collaboration of care consultants and families is used by other Association services as a blueprint for guiding interactions with caregivers and focusing on common service goals and objectives. Care consultants and other Association staff and volunteers also may jointly discuss cases and establish unified and consistent strategies for working with families; [eHealth] an adaptable, accommodating approach helps users with various degrees of digital literacy & clear, verbal instructions would help clarify.<br>→ adapt intervention to fit local context without compromising fidelity (e.g. changing from 6 sessions to 4 or 2 while demonstrating effectiveness)<br>2- Improve appropriateness of intervention and grouping – PwD were separated during meal time so staff can assist them properly<br>3- Individualized client engagement (e.g. one-on-one interaction, personal names and emotional validation to spark positive emotional affect/engagement; adaptable scheduling for dyad; scheduling calls based on caregivers' availability improves access to care and engagement rates); staff work hours were adapted to accommodate client/family schedules to conduct	1- Van Mierlo et al. (2017), Bass et al. (2003), Meyer et al. (2018), Werner et al. (2020), Pagan-Ortiz et al. (2014), van Knippenberg et al. (2017)<br>2- Gaugler (2014)<br>3- Gaugler (2014), Barrado-Martin et la. (2019), Judge et al. (2010), Mavandadi et al. (2017), Paone (2014)<br>4- Barrado-Martin et al. (2020), Fortinsky et al. (2016), Menne et al. (2014) Paone (2014), Banbury et al. (2019), van Knippenberg et al. (2017), Glueckauf and Loomis (2003), Nichols et al. (2016)

		components of program. [eHealth] multi-modal delivery (web-based and mobile platforms + printed information) allow users to respond and provide feedback according to their own schedules & facilitates faster knowledge transfer<br>4- Access - Location of intervention (flexibility) – home visits by the coach/instructors were reportedly helpful; [eHealth] home-based access was allowed by using video communication platforms (e.g. Zoom) & adapting to users' needs by transitioning from live sessions to pre-recorded, web-based classes followed by live toll-free telephone follow up discussion group. Scaling up can occur by using multi-modal delivery (e.g. face-to-face in facility paired with iPads linking caregivers in the home to clinicians in the facility..	
**[I/E] Trialability**	1/67; 1.49%	1- Testing an intervention as a component in the VA's outpatient medical home; requests are made to expand to other settings (e.g. adult day healthcare/medical foster care)	1- Nichols et al. (2016)
**[I/F] Complexity**	1/67; 1.49%	1- Lack of complexity and clear guidance improves implementation outcomes; most participants felt they could implement iGeriCare immediately by using collateral promotional materials that had been developed (e.g. sharing website URL) & the simplicity of the implementation was major facilitator. Little additional planning was needed.	1- Levinson et al. (2020)
**[I/G] Design Quality & Packaging**	43/67; 64.18%	1- Programming & appropriate content delivered in an appropriate way (to fit user needs) – e.g. Social club for PwD (3 times per week) to participate in recreational activities and psychomotor therapy & caregivers can also participate in social activities and consultation/meetings where they share experiences, receive helpful/practical information and offer feedback to program coordinators; [respite] programming (e.g. staff training, care routine, scheduling) offered a positive and engaging (socialization and meals) experience and a sense of trust, safety and security for caregivers; Comfortable lessons that allow socialization between caregivers; [Stevens et al. (2012)] additional components were added to support caregivers directly – Resources (local/national resource links) & My Family Profile.<br>2- Clear criteria of program inclusion to create homogenous/uniform groups<br>3- Useful, comprehensive (unfragmented), well-curated, well-timed, suitable and clear content in educational/promotional materials (e.g. relaxation CDs, manuals, group classes, daily video classes, resource notebooks used to address quality of life indicators & enable program delivery across various groups in multiple languages; a caregiver notebook, based on research and caregiver experiences, written in 'fifth grade reading level'; exercise logs for home-based exercise to record practices; manuals for exercises with clear photos as guidance)<br>→ The Memphis VAMC investigators (L.O.N. and J.M.-A.) condensed voluminous REACH II materials into a manual of operations/training manual, interventionist and support group leader manuals, and a caregiver notebook, which each participating facility staff member received. Notebooks were also provided for each caregiver (Nichols et al. (2011))<br>→ Intervention components were well preserved: The materials are arranged into nine sections: Home Safety, Using Social Support, Managing Stress, Pleasant Things For You, Healthy Living, Understanding Your Feelings, Skillful Communication, Relating Memory Problems to Behaviors, & Legal and Medical Information. Each section includes all materials necessary to deliver the intervention component according to the REACH II protocol. (Stevens et al. (2012))<br>→ clinicians are providing the intervention face-to-face in the facility and with iPads linking the caregiver in the home and the clinician in the facility (Nichols et al. (2016))<br>4- A human instructor (e.g. therapist, coach, interventionist, IT person/technical support, advisor) was a facilitator in the interventions as they provide personal attributes with an empathetic approach that validates caregivers' feelings & allows users to ask individual questions in real-time. Good use of personal experience to relate to participants (PwD & provide verbal positive reinforcement through good presentation/speech/clear tone of voice. Private consultation for caregivers alone is also important for successful implementation* & for caregivers to develop their own coping strategies.<br>→ The caregiver training addressed the components of the intervention and caregivers were shown how to use the manual and how to engage the person with dementia in stimulating activities (Milders et al. (2016))<br>→ Ongoing consultation with staff members (e.g. coaching calls) facilitated the use of materials (Nichols et al. (2011))<br>→ Having a trained professional, the SHARE Counselor, who creates a structure of support and a foundation of trust gently guides care dyads' toward exploring the PWDs' values and ensuring the CGs' understanding of what their loved ones think	1- Mazurek et al. (2019), Meiland et al. (2005), van Haeften – van Dijk et al. (2015), Gaugler (2014), Roberts and Struckmeyer (2017), Kovaleva et al. (2017)<br>2- Van Mierlo et al. (2017)<br>3- Chang et al. (2010), Sommerlad et al. (2014), Griffiths et al. (2015), Milne et al. (2014), Lykens et al. (2014), Martindale-Adam et al. (2017), Barrado-Martin et al. (2020), Prick et al. (2014), Burgio et al. (2001), Burgio et al. (2009), Nichols et al. (2011), Stevens et al. (2012), Orsulic-Jeras et al. (2016), Nichols et al. (2016), Gaugler (2018)<br>4- Sommerlad et al. (2014), Martindale-Adam et al. (2017), Milne et al. (2014), Barrado-Martin et al. (2019), Prick et al. (2014)*, Milder et al. (2016), Nichols et al. (2011), Orsulic-Jeras et al. (2016), Burgess et al. (2020), Gitlin et al. (2010), Bass et al. (2003), Banbury et al. (2019), Boots et al. (2017), Pot et al. (2015), van Knippenberg et al. (2017), Glueckauf and Loomis (2003), Xiao et al. (2020)<br>5- McCurry et al. (2015), Milders et al. (2016), Stevens et al. (2012)<br>6- Milne et al. (2014), Bass et al. (2014)<br>7- Barrado-Martin et al. (2019)<br>8- Burgess et al. (2020), Field et al. (2019)<br>9- Dam et al. (2019), Meichsner et al. (2018), Pagan-Ortiz et al. (2014), van Knippenberg et al. (2017), Glueckauf and Loomis (2003), Xiao et al. (2020), Mitchell et al. (2017)

			is important. Once understanding is achieved, discussion can more easily progress toward identifying other potential helpers in addition to the CG, as a strategy for reducing CG burden while assuring that the PWD's values and preferences are documented (Orsulic-Jeras et al. (2016)<br>→ Care consultants help families identify personal strengths and resources within the family system/health plan/community; goal is to provide tools to enhance PwD and carers competency and self-efficacy. Care consultants also provide information about available community services to facilitate decisions about how to best utilize/apply to these services & may contact service agencies on behalf of patient and caregivers. (Bass et al. (2003))<br>→ [eHealth] an IT facilitator was helpful in resolving technical issues (e.g. audio settings, explain different displays, user interface, connectivity, …) and can be reached via SMS messaging if there were issues during the meetings. Coach facilitated intake sessions (introduction) allowed personal connections that motivated users (carers), reduced feelings of isolation & allowed users to ask questions. Providing a summary of questions and responses following educational sessions were helpful.<br>5- Co-creation<br>→ Behavioral plans and homework used as an audit and feedback tool: Consultant works with user to develop plans and behavior plans; homework and weekly progress notes from consultants & 4-monthly telephone calls that follow in-home session allowed an appropriate form of fidelity/adherence monitoring. Other resources used include crosswords, photograph.<br>→ Intervention was developed in consultation with healthcare professionals, PwD, caregivers and representatives of dementia organisations (Milders et al. (2016)).<br>→ Intervention components were co-created in collaboration with technology specialists from Scott & White Siemens Information Technology team and the nursing staff, two key questions were embedded into the hospital admissions EMR infrastructure across the entire hospital. If a nurse identified a person as having Alzheimer's or dementia or being a caregiver of a person with Alzheimer's or dementia, an automatic display would prompt the nurse to give the caregiver a "Caregiver Packet," our primary recruitment tool. These prompts electronically created daily reports of admitted eligible patients for the FCP staff and were critical for self-evaluation of the program methodology. (Stevens et al. (2012)).<br>6- Program embedded in existing services within health system – Service provided as part of existing post-diagnostic services as a support group ('Carers Course) met less resistance; interagency coordination of care within existing systems facilitates implementation (e.g. One care coordinator worked in the local VA medical center (healthcare organization) and the other worked in the partnering Alzheimer's Association chapter (community service organization)<br>7- In-person, on-site classes were facilitated by a good venue (convenient location, well-connected via public transport, ample parking space), suitable class size and duration of class.<br>8- Timing of intervention delivery is a facilitator – e.g. delivering Occupational therapy at an early stage may overwhelm the caregiver so providing information steadily/gradually is facilitator; some interventions should be introduced early on after diagnosis to ensure necessary resource are clear and available.<br>9- Digital platform had an intuitive user experience – User-focused format/layout ("circular structure") was valued for the privacy and autonomy carers had as they could decide what was shared and with whom (e.g. within groups, across social networks); privacy can also be protected by issuing each user a unique user identification and password to participate in classes. The platform should also provide clear deadlines for assignments/tasks. Platform navigation should be clear for all levels of literacy; font size, content, and audio should be clear (e.g. sufficiently loud audio, big/clear text/font, clear contrast between text and background, no distracting content) for users. Important relevant information should be centralized on the platform (e.g. clear, useful links to federal, state, local community resources & community elder care organizations across the implementation setting (e.g. frontline services, support with ADL, attendant care, crisis management). Useful functions should be available (e.g care book that streamlines coordination/transfer care information; in-app messaging function & chat rooms (e.g. AlzChat), calendar for planning/appointments; support request functions (convey request/offer support, discussion forums for caregivers, message board, library and helpful links to resources).	10- Levinson et al. (2020)<br>11- Lykens et al. (2014), Mavandadi et al. (2017)

		Application service provider can be used to deliver secure live classes, slide presentations and chat room. Real-time information for longitudinal care tracking (e.g. health data, biometrics, video recordings) can support clinicians with diagnosis/care planning. Allow users to share via social media to disseminate intervention & maintain worldwide presence.<br>10- Customizable aspects of intervention should be easily adapted to be implemented into existing workflow to reduce barriers to implementation.<br>11- Removing communication/information sharing barriers to adapt to end-users: Adapting terminology to 'counselors' from 'dementia care specialist' to avoid miscommunication/misunderstanding; creating flexible/tailored and manualized program materials to support (localized) individual users' needs/preferences/co-morbidities	
**[I/H] Cost**	4/67; 5.97%	1- Funding came from grants provided by private foundations or government-support (e.g. (USA) Veteran Affair, respite vouchers for caregivers to cover costs of residential respite provider).<br>2- Cost of intervention – Telephone-based caregiver support interventions are cost efficient and can improve outreach in smaller clinics/rural settings. Internet based platforms are low cost options for caregivers.	1- Martindale-Adam et al. (2017), Roberts and Struckmeyer (2017)<br>2- Mavandadi et al. (2017), Xiao et al. (2020)
[II] Outer Setting			
**[II/A] Patient Needs & Resources**	22/67; 32.84%	1- Convenience for carers – Service opening hours, location of service, transportation options, scheduling flexibility all allow caregivers to use service, appropriate & easy-to-use materials, external memory aids.<br>2- Caregivers need more support due to increasing dependency level of PwD and personal health problems<br>3- Realistic approach to care - Dyads felt included in goal-setting process (co-creating realistic goals), respite care (temporary institutionalization) is more suitable for carers and programming (i.e. activities) stimulates PwD<br>4- Direct engagement with ADS staff benefited caregivers by providing information and training; carers also appreciated the direct assistance, acknowledgement and emotional support.<br>5- Caregiver involvement facilitated implementation & co-created adaptations to suit cultural and religious needs and streamline implementation (e.g. filial piety enforces a sense a responsibility for caregiving to elders/parents) so implementation consultants need to be aware of the social and cultural dynamics of the family/community.<br>6- Raising awareness, enhance receptivity by routinizing interventions & supporting participants through sharing more information about dementia and available support services (e.g. filling forms and accessing financial support such as Attendance Allowance)<br>7- Timing of intervention as it relates to what and when caregivers need support – Depending on the type of intervention, carers may prefer to receive information at the onset of dementia or at time of diagnosis (e.g. earlier engagement with the START programme would have helped them improve their communication and thus care better or avoid making major decisions regarding social care without being equipped with the necessary knowledge of dementia), whereas end-of-life planning may not be suitable at this stage.<br>8- End users' good digital literacy facilitates the successful operation of technology (e.g. connecting to meeting rooms, trouble shoot difficulties without IT support, access internet)<br>9- Age of caregiver facilitates use – younger caregivers are more inclined to use eHealth services due to their need for flexibility and work-related computer literacy. Web-based format provides flexibility and clinicians and carers to overcome the time barrier. Majority of messages were sent outside of regular office hours which suggests that participants only found time for themselves after caregiving duties were completed; asynchronous communication with therapist allows continued use to therapeutic support.	1- Beisecker et al. (1996), Judge et al. (2010)<br>2- Brandao et al.(2016),<br>3- Brandao et al. (2016), Beisecker et al. (1996), Burgess et al. (2020)<br>4- Gaugler (2014), Nichols et al. (2016)<br>5- Van Mierlo et al. (2017), Lykens et al. (2014), Meyer et al. (2018)<br>6- Milne et al. (2014), Field et al. (2019), Bass et al. (2003)<br>7- Sommerlad et al. (2014)<br>8- Banbury et al. (2019), Teles et al. (2020), Pot et al. (2015), Xiao et al. (2020)<br>9- Boots et al. (2017), Dam et al. (2019), Meischner et al. (2018), Levinson et al. (2020)
**[II/B] Cosmopolitanism**	29/67; 43.28%	1- Community integration of new services – (e.g. interaction and participation in activities/events outside of the program)<br>2- Maintain network within community (e.g. relationship between support officer, local organization, be active in care networks and taking initative with care innovations)	**1- Van Mierlo et al. (2017), Gaugler (2014)**<br>**2- Meiland et al. (2005)**<br>**3- Meiland et al. (2005), Van Rijn et al. (2019), van Haeften – van Dijk et al.**

		3- Harness existing partnerships/collaboratives to a) develop intervention, b) facilitate referrals, tuning and c) placement in institutional facilities and execute the delivery of intervention(s) or establish new partnerships and collaborative arrangements to execute, continue and sustain implementation<br>→ Invite various stakeholders to the table (i.e. information meeting, discussion groups, initiative group) when developing and translating projects; [“Examples of cooperating organizations were the local Alzheimer association, mental health organizations, general practitioners, home care organizations, case managers and the local carer support organization. The members of the project group together with representatives of the cooperating organizations formed the initiative group and this group worked according to a step-by-step guide. Various workgroups, consisting of staff of the daycare centre and/or other employees of the initiating organization and representatives of the cooperating organizations, addressed different topics that they specialized in.<br>→ Implementing partners with various levels and across sectors (e.g. community assessment, secure funding) -<br>(NL) Good collaboration with municipality organizations facilitates intervention as they are also potential referrers (e.g. Alzheimer Nederland emphasized importance of working with case managers, who are close to families) (e.g. collaborate between care and welfare organisations at a local level via existing active networks or developing new networks; use a collaboration protocol/agreements to clarify and solidify formal partnerships without need for further negotiation);<br>(USA) Network with Area Agencies on Ageing (AAA) – Interventionists (AlzOnline) made regular contact with AAA personnel & caregiver advocates; (USA) Partnering with local advisory boards and conduct a community assessment to identify resources, strengths, gaps in services, and strategies to coordinate among service providers; (USA) Partnering with universities and other agencies: REACH OUT program was controlled by the AAAs, UA’s Center for Mental Health and Aging provided materials, training and consultation for case managers;<br>(USA) Government agency- third sector organisation- Research center collaboration: RDAD is a collaborative project between Ohio Department of Aging, Alzheimer’s Association Chapters serving Ohio, and the Benjamin Rose Institute on Aging, in partnership with the original RDAD researcher, Linda Teri, PhD.<br>4- Recruitment/referrals to intervention –<br>Health professionals (e.g. clinicians/GP) need to be willing to recommend intervention as appropriate service;<br>(NL) recruitment of users was facilitated through large networks (via flyers, Alzheimer Nederland (Facebook/Website ad), online newsletters, local parish newsletters, community services, Alzheimer’s cafes, case manager referrals, and pre-established networks);<br>(NL) referrals made between network organisations (e.g. care/support organisations and caregiver organisations);<br>(Portugal) Patients were recruited through community projects or National Alzheimer’s Association (pre-acquaintance with support & information on available services);<br>(USA) Partnerships with hospitals and local churches to coordinate dissemination and registration process;<br>(USA) partnerships with Alzheimer’s Association local chapters (“catchment area”); (USA) Referrals made to community services from Area Agencies on Ageing (AAA);<br>(USA) “REACH VA was exposed to a wide variety of VHA leaders in geriatrics, home care, health services research, mental health, and social work”;<br>(USA) Home care workers, Alzheimer’s help line and support groups refer users to respite care services<br>(USA) “Agencies were most successful with reach and adoption when they partnered with entities that could assist with recruitment and participants could be drawn from existing membership or contacts. Thus, adult education programs advertised the MSCP in their marketing materials; hospitals advertised on their outside signage, in newsletters, and flyers; faith-based communities drew from their congregations. Partners having a local champion, whether internal to the organization or a former MSCP participant, contributed to success.”<br>5- Implementing partners – Initiators sign contract with “application service provider” that offers solution that was inexpensive and versatile; boost role efficiency by partnering with supplemental agencies to support functions.	**(2015), Menne et al. (2014), Burgio et al. (2009), Cho et al. (2019)**<br>**4- Gaugler (2014), Dam et al. (2019), Boots et al. (2017), Teles et al. (2020), Connell and Kole (1999), Bruce and Patterson (2000), Stevens et al. (2012); Hendriks et al. (2018), Meyer et al. (2018), Nichols et al. (2016), Beisecker et al. (1996), Brandao et al. (2016), Samia et al. (2014)**<br>**5- Glueckauf and Loomis (2003), Bass et al. (2014), Bass et al. (2003), Fortinsky et al. (2016), Droes et al. (2019), Lykens et al (2014), Martindale-Adam et al. (2017)**<br>**6- Glueckauf and Loomis (2003), Pagan-Ortiz et al. (2014)**<br>**7- Bass et al. (2014), Pleasant et al. (2016)**

		→ A key feature of PDC is its basis in a formal partnership between a healthcare organization (for example, VA medical centers) and a community service organization (for example, Alzheimer's Association chapters). One care coordinator worked in the local VA medical center and the other worked in the partnering Alzheimer's Association chapter. The VA care coordinator also focused on helping families access VA services and benefits (including medical-related concerns [medication, access to services]), whereas the Alzheimer's Association care coordinator focused on helping families use community services, including those offered by the Alzheimer's Association. This division of labor between care coordinators capitalized on the complementary strengths of each partner organization and represented a bridge between health care and community services. (Bass et al. (2014))<br>→ Embedding care into existing services via networks - The partnership tested in this investigation adds care consultation from the Cleveland Area Alzheimer's Association (improve the quality of information and support services for patients with dementia and their family members) to usual managed care services offered to members of Kaiser Permanente. (Bass et al. (2003))<br>→ Receiving training (via separate agency & university) to adopt and embed intervention - To implement the new interventions, the staff of the Meeting Centers who were responsible for the implementation received a two-day training, followed by "coaching on the job" provided by the private company Dirkse Anders Zorgen (DAZ), the Dementel coach cooperative association, and the Amsterdam Center on Aging of VU University Medical Center (VUmc). (Droes et al. (2019))<br>→ United Way (a nonprofit organization) of Tarrant County contracted with the Alzheimer's Association to implement the REACH II program as a component of support services to Alzheimer families. (Lykens et al. (2014))<br>→ Networks embedding program (Academic-Public partnership)- A partnership of Indian Health Service (IHS), primarily the Division of Nursing Services, the Administration on Community Living (ACL) through the Administration on Aging's Native American Caregiver Support Services Program, and the University of Tennessee Health Science Center (UTHSC), was formed to implement REACH for caregivers of persons with dementia regardless of veteran status. (Martindale-Adam et al. (2017))<br>6- Active dissemination – Initiators made presentations, conducted workshops (e.g. statewide caregiver forums, elder leadership institutes, Alzheimer's Disease Initiative Advisory Committee meeting). Use of social media to notify users of new content (via Facebook, Twitter); social media creates a sense of community for users through shared articles, written responses and user-suggested topics to guide future content<br>7- Licensure and training institute to disseminate intervention (i.e. dissemination agency) - Benjamin Rose Institute on Aging holds the copyright to BRI Care Consultation and currently licenses and trains organizations to deliver the program. Since completion of PDC, more than two dozen diverse organizations have been licensed to deliver BRI Care Consultation, including healthcare organizations, Alzheimer's Association chapters, family counseling agencies and Area Agencies on Aging; (USA) Oregon Care Partners was established to implement free training for all caregivers in the state, reflecting the Oregon Alzheimer's Disease State Plan recommendations.	
**[II/C] Peer Pressure**	3/67; 4.48%	1- Agencies face pressure to implement new interventions by national agenda (e.g. National Alzheimer's Project Act) – strategies should be shared with state, tribal and local governments<br>2- Interventions that have positive image become interesting to other organisations that seek similar programs that fit their mission. An effective pilot program will enhance acceptance as it provides a clear image of the model and how other organisations can benefit from the program.	**1- Martindale-Adams et al. (2017)**<br>**2- Van Mierlo et al (2017), Meiland et al (2005)**
**[II/D] External Policy & Incentives**	20/67; 29.85%	1- Successful recruitment to financiers facilitates implementation –<br>(NL) municipal welfare departments were often approached to finance support for carers & preparations were facilitated if the organizations involved were abreast of the laws and regulations concerning financing from the care and welfare sectors. For example, the government had set up various incentive schemes, such as 'waiting list subsidy scheme', 'tailor-made care funds', and the 'informal care subsidy scheme'. In addition the meeting centres can appeal to structural funds,	**1- Meiland et al. (2005), van Mierlo et al. (2017), van Rijn et al. (2019), Roberts and Struckmeyer (2017), Werner et al. (2020)**

		such as those for day care. In The Netherlands this is done through the AWBZ (the Exceptional Medical Expenses Act). Meeting centres can qualify as a day treatment, which is financed by the care administration office. The umbrella organization of Homes for the Elderly and Nursing Homes, and the Welfare umbrella organization campaigned jointly, to accomplish an adequate fee for continuation and implementation of meeting centres;<br>(NL) In 2015 the laws and regulations involving elderly care were divided into three new, separate support and care acts. As mentioned, most Meeting Centers received funds from their municipality as part of the Social Support Act(WMO), which proved a facilitating factor for the implementation of DemenTalent;<br>(Poland and Italy) Meeting centres were financed by the municipality as a social welfare program that integrates social and health aspects.<br>(USA) respite care programs were funded by quarterly-distributed vouchers (if caregivers get their paperwork in on time and if there are sufficient funds available in the small state-level allocation)<br>(Israel) Program funded by National Insurance Institute to the Alzheimer's Association, covering the salary of the coordinator, the reimbursement to the counselors, and the evaluation of the effectiveness of the intervention, which was conducted by researchers who were not members of the Israeli Alzheimer's Association<br>2- National dementia strategy/agenda facilitated implementation (e.g. Government Acts/Policies) –<br>(NL) Interventions (e.g. MSCP, DemenTalent) that qualify under health and social care (or align with multi-level (i.e. municipal/national) government policy) can be funded locally but national regulations need to be updated to include new interventions.<br>(USA) 2013 National Plan to Address Alzheimer's Disease (required by the National Alzheimer's Project Act) mandated lessons learned through VA caregiver support strategies be shared with state, tribal and local governments.<br>(USA) Amendments to the Older Americans Act calls for care coordination to link healthcare services and community services<br>(USA) On January 4, 2011, the National Alzheimer's Project Act (NAPA) was signed into law. The National Plan to Address Alzheimer's Disease mandated that lessons learned through VA caregiver support strategies, specifying REACH VA, should be shared with other federal agencies. This has continued through each update of the Plan. REACH VA is also expanding in the Federal Government, in partnerships through NAPA. REACH VA is currently being pilot tested with several Tribal Nations sites through the Indian Health Service and the Administration for Community Living and Administration on Aging<br>(USA) In May 2010, Public Law 111-163 Caregivers and Veterans Omnibus Health Services Act of 2010 was signed into law. It will allow the Veteran Affairs (VA) to provide unprecedented benefits to caregivers who support the veterans who have sacrificed for this nation. The VA is discussing the feasibility of implementing national options, including REACH VA, for caregivers. On a local level, several VAMCs that have not previously implemented REACH VA have requested training. This growing interest in assisting caregivers reflects the statements of a REACH VA interventionist who said, "Investment in the caregiver is a direct investment inpatient care. These larger changes were part of the mission of the national Caregiver Support Program (CSP), of which the Memphis VA Caregiver Center was a part.<br>(USA) Medicare provides long-term care reimbursements and funds part-time skilled homecare (e.g. physical , speech, occupational therapy)<br><br>3- Health systems shift from hospital-based care toward community based care; (e.g. In Australia, Aged Care Assessment Teams (ACAT) were set up by the Commonwealth Government of Australia initially for assessment for institutional care, but their role has developed to include assessment for a variety of community support programs)<br>→ Statewide scale up of MSCP program to serve all 16 of Maine's counties with ADRC/AAAs assuming the lead role in partnerships with various community organisations.<br>4- Government-initiated programs & regulatory policies– (USA) The Minnesota project (called Minnesota Family Memory Care, or FMC) is administered by the Minnesota Board on Aging and the Minnesota Department of Human Services. The	**2- Van Mierlo et al. (2017), van Haeften-van Dijk et al. (2015), van Rijn et al. (2019), Martindale- Adams et al. (2017), Bass et al. (2014) Nichols et al. (2011), Nichols et al. (2016)**<br>**3- Milne et al. (2014), Bruce and Patterson (2000), Samia et al. (2014)**<br>**4- Mittelman and Bartels (2014), Paone (2014)**<br>**5- Xiao et al. (2020)**

		FMC program began in 2008 at four sites; five new sites were added in each of the second and third years. The State also set a requirement for clinical supervision of the consultant. In response to these and other translation issues, the NYUCI program protocol, assessment, training, and reporting tools were modified or enhanced.<br>5- Consumer-directed care model facilitates implementation; Online support network model applied to 'Carer Gateway', a platform that accommodates online support for caregivers from all care areas nationwide & managed by Department of Social Service. The health-professional led integrated network model overcomes disadvantages in the private blogs model and the generic online support model; this study suggests informal caregivers preferred health professional-led integrated network model for informal caregivers.	
[III] Inner Setting			
**[III/A] Structural Characteristics**	1/67; 1.49%	1- Structural financing was a facilitator : Structural funding increased if the project was successful and was scientifically proven to be effectiveness	1- Meiland et al. (2005)
**[III/B] Network and Communications**	3/67; 4.48%	1- Regular internal communication & updates facilitate the implementation (e.g. all staff members are informed and keep in contact with project coordinator); (e.g. regular internal meetings and training (with support from information packets and nursing education) across levels of management is necessary to ensure all parties are able to operate new EMR system, which will enhance the adoption of the program)	1- Hendriks et al. (2018), Werner et al. (2020), Stevens et al. (2012)
**[III/C] Culture**	3/67; 4.48%	1- Staff enthusiasm about intervention - (e.g. Family Memory Care consultants expressed support toward program and understood its importance to their end-users to improve their capability to cope)<br>2- Program was a good cultural fit for the organisation's mission – (e.g. MSCP is a good fit and was well-integrated in Aging and Disability Resource Centers (ADRC) /AAA's family care program	1- Paone (2014),<br>2- Samia et al. (2014), Werner et al. (2020)
**[III/D] Implementation Climate**			
**[III/D1] Tension for Change**	2/67; 2.99%	1- Existing cooperation between care and welfare organisations reduces tension for change and other parties are willing to cooperate if partnering organisations are trustworthy (e.g. VU medical centre and municipality)<br>2- Support from clinical staff and leadership facilitates implementation and reduces tension for change.	1- Van Haeften – van Dijk et al. (2015)<br>2- Nichols et al. (2016)
**[III/D2] Compatibility**	15/67; 22.39%	1- Timing of implementation within organization (e.g. coordination - clinicians and facility administration should all align at time of implementation)<br>2- Modifying components of intervention to enhance organizational fit (e.g. fitting into staff's/organisation's routine, practices and budget ensures sustainability):<br>3- Interventions' effect should align and fit with the vision/policy of the organization to facilitate implementation & Ability to routinize the intervention in the organisation is essential to translation of programs – "Variables to consider include primary purpose and mission for the organization, geographic location, size, familiarity with issues presented by caregivers of persons with Alzheimer's disease, and existence of caregiver services (usual care)." – if program fits (or can improve) these variables, then it is compatible. (e.g. [RDAD] Ohio trainers came from a variety of disciplines, including nursing, social work, counseling, and gerontology, and functioned as regular employees of Alzheimer's Association chapters. The goal was to implement RDAD as part of usual Alzheimer's Association services.)<br>→ Interventions should fit the clinical workflow and staff member's daily functions/roles (e.g. repurposing/modifying roles of existing staff)<br>4- The implementing organization should have a streamlined administrative structure that is ready to implement a) registration of caregiving independently from PwD, b) appropriate financing mechanisms and compensation, c) existing integration of similar programs, d) clinical infrastructure (e.g. billing and workload codes). (e.g. Medicare Current Procedural Terminology codes allow providers to be paid to communicate with caregivers about beneficiary's care). IHS is addressing these issues by developing coding guidelines to insure that nurses receive workload credit for REACH and can	1- Nichols et al. (2016)<br>2- Nichols et al. (2016)<br>3- Hendriks et al. (2018), Paone (2014), Stevens et al. (2012), Menne et al. (2014), Milne et al. (2014), McCurry et al. (2015), van Rijn et al. (2019)<br>4- Boot set al. (2017), Levinson et al. (2020), Martindale-Adam et al. (2017)<br>5- Meiland et al. (2005)

		document in the electronic health record. In addition, REACH implementation has been added to staff performance evaluations and instituted as a quality improvement project.<br>5- Ability to repurpose existing infrastructure (e.g. meeting locations) facilitates implementation to reduce resource constraints	
**[III/D3] Relative Priority**	0/67; 0%		
**[III/D4] Organizational Incentives & Rewards**	0/67; 0%		
**[III/D5] Goals and Feedback**	0/67; 0%		
**[III/D6] Learning Culture**	1/67; 1.49%	1- Internal training is necessary to successfully adopt new software within private company	1- Frame et al. (2013)
**[III/E] Readiness for Implementation**			
**[III/E1] Leadership Engagement**	7/67; 10.45%	1- Enthusiastic and motivated leadership engagement with support from clinical administration and physicians is critical (e.g. project leader who is proactive in seeking cooperation with other parties in the network, possess an intrapreneurial mindset ("out-of-the-box" thinking); involved managers should support/facilitate planning of project and have a clear agenda)<br>2- Leaders (e.g. Director of nursing and technology services [a licensed vendor of intervention] support developing a care plan that customizes services for users & is responsible for preparing reports/manuscripts.	1- Levinson et al. (2020), Martindale-Adam et al. (2017), van Haeften-van Dijk et al. (2015), van Rijn et al. (2019), Nichols et al. (2016), Connell and Kole (1999)<br>2- Mitchell et al. (2017), Connell and Kole (1999)
**[III/E2] Available Resources**	13/67; 19.40%	1- Human resources (e.g. well-trained staff members who were informed about dementia, long-term/committed team [low turnover rates], contract extensions for personnel to share their tasks (e.g. add more hours that they are paid for) and repurpose staff members to implement new program, …): varied and integrated support is offered to end-users;<br>2- Location (accessible, convenient, good atmosphere, situated in a region with effective care network that facilitated dissemination to target groups, …)<br>3- Local government support (e.g. financing, accommodation for program): (USA) Veteran Affairs funds ADS programs; (USA) National Institute on Ageing supports community outreach education programs as part of Alzheimer's Disease Research Centers in collaboration with Michigan Alzheimer's Disease Research Center at the University of Michigan in Ann Arbor & supplemental funding came from Health Resources and Services Administration via a grant to the Michigan Department of Mental Health<br>4- Facilitating strategies to obtain funds also include announcing the plan at an early stage, demonstrating the program is widely supported (gain traction through successful pilot program), clarify its surplus value; funding can also be pursued via additional ADSSP awards, private donors, and small grants.<br>→ (e.g. The northernmost ADRC/AAA is working with a community hospital to seek funding to continue the MSCP and strengthen its marketing capacity. ADRC/AAAs also discussed opportunities to work with retirement communities, especially in the more affluent communities, to secure future program support. At the time of this evaluation, sustainability within the Alzheimer's Association is tenuous pending opportunities to cover a smaller territory and replenish trainer resources. (Samia et al. (2014))<br>→ Financing for a strategic marketing initiative was provided by a private foundation (Robert Wood Johnson Foundation)<br>5- Resources for recruitment include 24/7 telephone helpline, support groups, case managers, the Aging & Disabilities Resource Center, partnering agencies, and home health agencies<br>6- Readily available resources include office space, Technology to facilitate implementation (computer/telephone; allows training for staff), administrative support (existing human resources); eHealth interventions can be easily disseminated using a website URL or using various promotional materials without needing new investment/resources.	1- Meiland et al. (2005), van Rijn et al. (2019), Paone (2014), Xiao et al. (2020)<br>2- Meiland et al. (2005), van Rijn et al. (2019), Beisecker et al. (1996), Stevens et al. (2012)<br>3- Meiland et al. (2005), van Rijn et al (2019), Roberts and Struckmeyer (2017)<br>4- Meiland et al. (2005), van Rijn et al (2019), Samia et al. (2014), Glueckauf and Loomis (2003)<br>5- Lykens et al. (2014)<br>**6-** Martindale-Adam et al. (2017), Paone (2014), Levinson et al. (2020)

**[III/E3] Access to Knowledge & Information**	11/67; 16.42%	1- Re-train staff members & redefine roles using a cascade model of training: "A formal train-the-trainer model with a specific protocol for associate trainer certification was developed and implemented"<br>→ Trainers were instructed by the research coordinator (developer/initiators) and/or a healthcare professional; trainers were provided manuals with step-by-step instructions to deliver caregiver training across several sessions. Cascade model of training reduced time and resource expenditure; training manuals allowed efficient and timely training while maintaining treatment fidelity. Primary cohort of trainers would be responsible for training future cohorts of trainers.<br>2- Trainer certification (formal train-the-trainer model with specific protocol for associate trainer) facilitate implementation & allows implementer to utilize employees and volunteer trainers, who had dementia knowledge/experience and group facilitation experience.<br>3- External training agency - To implement the new interventions, the staff of the Meeting Centers who were responsible for the implementation received a two-day training, followed by "coaching on the job" provided by the private company Dirkse Anders Zorgen (DAZ), the Dementelcoach cooperative association, and the Amsterdam Center on Aging of VU University Medical Center (VUmc).<br>→ In VA, clinical staff, predominantly psychologists, social workers, and nurses, are trained and certified by the Memphis Caregiver Center to deliver the intervention. In community, agency staff deliver the intervention. (Martindale-Adam et al. (2017))<br>4- Training sessions and coaching-on-the-job was provided by the consultant	1- Milders et al. (2016), Mittelman and Bartels (2014), Samia et al. (2014), Werner et al. (2020), Hendriks et al. (2018), Nichols et al. (2011), Orsulic-Jeras et al. (2016), Lykens et al. (2014)<br>2- Samia et al. (2014)<br>3- Droes et al. (2019), Martindale-Adam et al. (2017)<br>**4-** Van Haeften-van Dijk et al. (2015)
[IV] Characteristics of Individuals			
**[IV/A] Knowledge & Beliefs about the Intervention**	2/67; 2.99%	1- By whom the intervention was created also influenced staff and end-user acceptance/perception – (e.g. participants who identified more with the organization that developed the intervention were more enthusiastic about implementation, although some of this may also reflect the construct of intervention source [e.g. internally developed]) (e.g. community empowerment and ownership of the intervention would help its uptake, effectiveness and sustainment)<br>2- If the implementation agent does not identify with the organization, then the individual characteristics of the agent (e.g. physician/coach) will affect outcomes. (e.g. Many physicians in independent practice might not strongly identify with their affiliated health care organizations (eg, hospitals), highlighting the added importance of individual characteristics as a construct in an organization's potential implementation that may rely on physicians)	1- Levinson et al. (2020), Meyer et al. (2018)<br>**2-** Levinson et al. (2020)
**[IV/B] Self-efficacy**	8/67; 11.94%	1- Intervention improved caregivers' sense of competency in their role & more equipped with communication skills and coping strategies.<br>2- Training helped consultants develop skills and confidence in both usual care and when delivering the intervention	1- Foley et al. (2020), Sommerlad et al. (2014), Nichols et al. (2016), Burgess et al. (2020), Banbury et al. (2019), Dam et al. (2019)<br>**2-** Paone (2014), Martindale-Adam et al. (2017)
**[IV/C] Individual State of Change**	2/67; 2.99%	1- End users (carers) became more comfortable, acquainted and confident with their coach as the intervention progressed	1- Boots et al. (2017), Levinson et al. (2020)
**[IV/D] Individual Identification with Organization**	3/67; 4.48%	1- Staff members who clearly identified with the organizations of the developers of the intervention were more positive toward the intervention and its implementation; staff who were more enthusiastic on the part of various parties involved (e.g. organization, initiators, staff) were a facilitating factor.<br>2- In a fully operational program, staff are comfortable in their roles and the organization supports the intervention.	1- Levinson et al. (2020), Meiland et al. (2005)<br>**2-** Martindale-Adam et al. (2017)
**[IV/E] Other Personal Attributes**	10/67; 14.93%	1- Staff members ability to adapt to ad hoc needs (e.g. make unexpected modifications to accommodate program implementation, new volunteers and staff turnover): staff had to assume multifaceted care roles including staff as "serving"(e.g., preparing and serving food with an eye toward hospitality), working together collaboratively, provision of	1- Lykens et al. (2014), van Haeften- van Dijk et al. (2015), Gaugler (2014)

		intensive activity of daily living care, and offering flexible care to meet the needs of clients as well as family caregivers (Gaugler (2014))<br>2- Counsellor/trainer/coach/project coordinators competencies (as perceived by caregivers) - Caregivers pointed to several counselor activities and skills that made the NYUCI-AC useful, such as counselors' ability to encourage them to discuss challenging issues related to behavioral management or coping with stressful aspects of care. (...) Adult child caregivers noted that having a counselor who has had caregiving experiences of his or her own was invaluable; providers who understand the norms and cultural preferences of caregivers in the area (e.g. language competencies to adequately support cultural groups), strong existing relationships with local providers, active and resourceful in identifying potential referral sources, and persistent in promoting the program community wide; caregivers perceive the IT support person as "helpful, patient and able to talk to patients calmly through issues, thereby increasing their confidence and willingness to use technology in the future<br>3- Caregiver's attitudes during and toward the intervention: facilitator includes having a positive attitude and willingness to try the intervention ("giving it a go")	2- Gaugler (2018), Hendriks et al. (2018), Meyer et al. (2018), Paone (2014), van Haeften- van Dijk et al. (2015), Banbury et al. (2019)<br>**3-** Field et al. (2019)
[V] Process			
**[V/A] Planning**	13/67; 19.40%	1- Plan for adapting and translating the intervention to fit local context facilitates implementation: Translating clinical interventions into a consumer-friendly program in a format that is used in healthcare settings will help achieve acceptance from clinical delivery staff, who are key referral sources and potential end-users. Program translation into real-world settings may require language translation, collaborative planning in work groups/advisory committees, collaborative implementation sites selection, and supplemental adapted materials (e.g. manuals) from the intervention to create community intervention program.<br>2- Planning to embed program into routine operation (preparation phase) – The process of planning the routinization of care is facilitated by effective leadership and communication between project leader and study coordinator; planning and scheduling between care managers/providers and end-users (e.g. carers) is a facilitator. Exploring the region/community to determine landscape (e.g. reimbursement requirements), contextual needs, finding an accessible location with sufficient facilities and room to house the program, and creating support for the program (e.g. informing the (existing or new) local networks and find agency partners/advisory boards) & organizing initiators. Determine needs through focus groups with service providers, family caregivers, and primary care physicians.<br>3- A clear project plan and implementation guide (e.g. manual) facilitates implementation – "Linking scientific research to the implementation process also facilitated implementation because it inspired confidence in the participants that bottlenecks would be demonstrated and improved"<br>4- Marketing plan & applying business-like strategies facilitates implementation (e.g. creating protocols, evaluation tools, schedules, and marketing strategies)<br>5- Make program a priority on the organization agenda and engage staff to start preparing at an early stage	**1- Stevens et al. (2012), Mittelman and Bartels (2014), Burgio et al. (2001), Burgio et al. (2009), van Haeften- van Dijk et al. (2015)**<br>**2- Fortinsky et al. (2016), Meiland et al. (2005), van Rijn et al. (2019), Connell and Kole (1999), Gitlin et al. (2010)**<br>**3- Hendriks et al. (2018), Paone (2014), Meiland et al. (2005) van Rijn et al. (2019)**<br>**4- Samia et al. (2014), van Rijn et al. (2019), Gaugler et al. (2014)**<br>**5- Meiland et al. (2005)**
**[V/B] Engaging**	21/67; 31.34%	1- Recruitment of participants/end-users (e.g. Carers) – a) Active dissemination - Recruitment materials include leaflets/program brochures and announcements, key facts sheet, participant information sheet (e.g. intervention guide & how to perform tasks such as exercise, fall prevention, …), information on participants' role in the program (contextual placement), confirmation cards to engage participants, (digital) newsletter articles and press releases at State and county level, provide incentives to attend (e.g. continuing medical education credits) and conduct workshops/presentations (e.g. forums, leadership institutes, advisory committee meetings)	**1- Barrado-Martin et al. (2019), Connell and Kole (1999), - Banbury et al. (2019), Glueckauf and Loomis (2003), van Knippenberg, Lykens et al. (2014), Meiland et al. (2005), van**

		b) Referral through engaging partners/network – Aged care providers, Alzheimer's Association, Alzheimer' s Cafes, ARDC; care and welfare organizations facilitate continuation & formal contracts should be established to begin collaborative agreement and protocol; involvement of religious/faith-based institutions are important to engage/recruit.<br>2- Using existing case files from medical records with specific diagnosis (e.g. Kaiser members (USA)), via clinicians/GP file referrals, or participants who were already enrolled in previous/partnering program (e.g. PACE/PACENET)(e.g. recruited from a pool of caregivers who received social support services at a neurology teaching hospital)<br>3- Marketing plans/strategies used to promote the program (e.g. business-like approach to marketing and management): Establish working groups for a) publicity/promotion and b) program/curriculum development; harness power of social media to spread information (e.g. Cuidate Cuidador – users shared website using Facebook, Twitter, and Youtube to enhance active engagement and expand website through UGC; Pagan-Ortiz et al. (2014) – using facebook advertising space and re-directing users to program webpage & engage users based on listed interest, such as Caregiving or Alzheimer's. Using site analytics to quantify reach.) Users and referrers feel the program is essential if promotion is continuous.	**Haeften – van Dijk et al. (2015), van Mierlo et al. (2017), Bass et al. (2014), Bass et al. (2003), Connell and Kole (1999), Droes et al. (2019), Nichols et al. (2011), Meyer et al. (2018), Samia et al. (2014), Stevens et al. (2012)**<br>**2- Bass et al. (2003), Mavandadi et al. (2021), Pagan-Ortiz et al. (2014), Paone (2014), Samia et al. (2014)**<br>**3- Gaugler (2014), Connell and Kole (1999), Pagan-Ortiz et al. (2014), van Mierlo et al. (2017), van Rijn et al. (2019), Hendriks et al. (2018), Samia et al. (2014)**
**[V/B1] Opinion Leaders**	2/67; 2.99%	1- Engaging local religious leaders and influential members of community or family to promote and normalize idea/program; involve local coordinator, who serves as the primary contact person and who provides leadership and strong advocacy to increase availability of services	**1- Meyer et al. (2018), Connell and Kole (1999)**
**[V/B2] Formally Appointed Internal Implementation Leaders**	8/67; 11.94%	1- The presence of a project manager (selected by leaders of the agency) to guide implementation is a strong facilitator who will attune the program to the population's needs; initial roles were revised to adopt the new program in addition to their regular duties. The project coordinator/initiator should be enthusiastic and positive about the program, and he/she should be fully professionally up-to-date and possess management experience. A multidisciplinary staff/advisory team leading the program is also important for successful continuation, and the project team is well-trained (e.g. full-time health educators trained at the master's level in publica health with expertise in community programs).<br>2- Project leaders (e.g. hires from external agencies such as Alzheimer's Association or consultants) follow a protocolized manual that supports in conducting an initial assessment, identifying problems/challenges and developing strategies used in the specific context. Project directors are also responsible for overseeing the program, managing partnerships with external agencies, supervising the project team and preparing reports/manuscripts.	**1- Van Mierlo et al. (2017), van Rijn et al. (2019), Meiland et al. (2005), Connell and Kole (1999), Gitlin et al. (2019)**<br>**2- Bass et al. (2014), Bass et al. (2003), Connell and Kole (1999), van Haeften-van Dijk et al. (2015)**
**[V/B3] Champions**	7/67; 10.45%	1- "influence the influencer" strategies engage local champions to reach secondary target groups and extended audiences due to the champion's position in local communities, including his/her reputation, trustworthiness, credibility, local awareness, and routine contact within the community.<br>2- Volunteers facilitate the implementation (e.g. Unforgettable program was facilitated by volunteer guides and hosts; they were motivated for the program and signed up with the program coordinator)<br>3- Advisory board members may include staff (e.g. healthcare professionals, dementia service providers, representatives of community/voluntary agencies) of local hospital, home health agency, information and referral agency, Alzheimer's Association chapters, county hospital, newspaper, and community health agencies. Multidisciplinary agents possess unique specializations to target various aspects/barriers to implementation. These agents should be involved across all phases of implementation to assure the data is locally relevant and to inform follow up activities after the funding period ends.	**1- Glueckauf and Loomis (2003), Meyers et al. (2018), McCurry et al. (2015), Martindale-Adam et al. (2017)**<br>**2- Van Mierlo et al. (2017), Hendriks et al. (2018)**<br>**3- Connell and Kole (1999),**
**[V/B4] External Change Agents**	11/67; 16.42%	1- Academic partners support dissemination, registration of new participants, and provide technical support.<br>2- External training for staff members was conducted by experts contracted by the government (e.g. Minnesota Board on Aging) or by private coaching/training organisations, which was described as substantial training and extremely important for program adoption and ensuring adherence.	**1- Droes et al. (2019)**<br>**2- Paone (2014), van Rijn et al. (2019), Droes et al. (2019), Samia et al. (2014)**<br>**3- van Mierlo et al. (2017), Connell and Kole (1999)**

		3- National organizations (associations, local municipality, Alzheimer's associations) provided active dissemination that made it possible to organize support and extend awareness. Faith based groups also support in facilitating through dissemination or providing accommodation (i.e. place to host intervention)<br>4- Recruiting staff from external charities and support organizations within networks/formal partnerships (e.g. Alzheimer's Association, social service providers for caregivers) was also a prominent facilitator (e.g. staff from AA chapters were trainers for programs (e.g. RDAD) due to their local positioning and multidisciplinary background and training in dementia.);<br>5- Hiring external agencies to disseminate program (e.g. marketing agencies) – "Shortly after obtaining the RWJF grant, we hired the Wilson Agency located in Jacksonville, florida to serve as our marketing consultants. Following their advice, we convened a small working group conference consisting of statewide leaders from DOEA, the state's Area Agencies on Ageing, Florida' chapter of the Alzheimer's Association, senior service organisations and ementia caregivers. The working group unanimously agreed that the best way to inform caregivers about AlzOnline was to encourage grassroots senior service organisations (i.e., those agencies who influence and support dementia caregivers) to refer their clients to our website."	**4- Menne et al. (2014), Milders et al. (2016), Mittelman and Bartel (2014), van Haeften – van Dijk et al. (2015)**<br>**5- Glueckauf and Loomis (2003)**
**[V/C] Executing**	14/67; 20.90%	1- Conduct program across multiple study sites and monitor regularly (e.g. documenting the intervention by recording contracts, providing assessment for caregivers, using interventionist documentation to assess treatment and seek feedback from user); adapt intervention on an ad hoc basis (e.g. language clarification, modifying terminology and content of written material to enhance professionalism)<br>2- Support from local advisory board to identify community resources & receive funding from various sources (e.g., annual fundraising event by National Alzheimer's Association); local hospitals and cable tv companies contributed to publicity and promotion<br>3- Execution phase was facilitated by secure financing and sound agreements about sustained costs; successful implementation followed collaborative community engagement; framing the program as 'community-driven' and patient centred<br>4- Continued PR throughout implementation process was facilitating – continued discussion about intervention within their network was effective.<br>5- Execution required adjustments facilitated by competent, attentive and reliable support staff; "To implement the new interventions, the Meeting centers who were responsible for the implementation received a two-day training, followed by "coaching on the job" provided by the private company DirkseAnders Zorgen (DAZ), the Dementelcoach cooperative association, and the Amsterdam Center on Ageing of VU University Medical Center."<br>➔ Execution was also successful when drivers were considered: training, consultation and coaching, administrative support and system innovations (Nichols et al. (2016))<br>6- Partnering agencies are supportive in adapting to lack of funding (e.g. face-to-face training was not included but it was valuable so they found ways to provide it; adopting alternate modalities to reduce costs, requiring fewer in-home visits); Continued execution beyond the duration of the grant was possible but long-term financing solutions need to be anticipated (e.g. gaps in funding).	**1- Bass et al. (2014), Mittelman and Bartel (2018), Burgess et al. (2001), Werner et al. (2020)**<br>**2- Connell and Kole (1999)**<br>**3- Meiland et al. (2005), Mazurek et al. (2019), Meyer et al. (2018),**<br>**4- Van Rijn et al. (2019)**<br>**5- Mitchell et al. (2017), Droes et al. (2019), Nichols et al. (2016)**<br>**6- Martindale-Adam et al. (2017), McCurry et al. (2015)**
**[V/D] Reflecting & Evaluating**	8/67; 11.94%	1- Assess treatment enactment (e.g. observe caregivers, collect data from caregiver self-report, document the process of intervention use [caregiver adherence, rating of intervention effectiveness, progress notes] and prepare to act responsively to unexpected circumstances that deter from 'roadmap' [Connell and Kole (1999)]<br>2- Reflecting on caregiver preferences when adapting intervention programs ("when translating or implementing complex, multi-component protocols for dementia caregivers, the incorporation of caregiver perspectives to effectively tailor the program for diverse cultural, familial, or socioeconomic contexts may be similarly warranted") [Gaugler (2018)]; adapting language (e.g. intervention has always been associated with drugs so 'program' would be more appealing) [Martindale-Adam et al. (2017)]; incorporating multi-perspectives (e.g. clergy members were not included in the community assessment because it was planned before the advisory board decided how the program would be targeted. Ideally, clergy	**1- Paone (2014), Burgio et al. (2001)**<br>**2- Gaugler (2018), Martindale-Adam et al. (2017), Connell and Kole (1999), Glueckauf and Loomis (2003)**<br>**3- Mittelman and Bartel (2014), McCurry et al. (2015)**

		members would have played an integral part in all phases of program planning." [Connell and Kole (1999)]; refining program based on real-world findings (e.g. changing components of intervention that created barriers to use, such as removing video feed & replacing with instructor photo to reduce bandwidth, refine UX/UI based on user feedback) [Glueckauf and Loomis (2003)]<br>3- Sustainment of program by developing economic model to build case for sustainable funding of program. Scale up the program by targeting specific groups (e.g. social workers) that can be trained to adopt new intervention; develop methods to sustain core elements of intervention through ongoing expert and peer clinical supervision. Develop new delivery mechanisms to reduce costs (e.g. digitalizing processes, changing location);	

Table 5. Implementation strategies identified across studies (n=67) based on the ERIC compilation	
**Implementation strategies**	**No. of studies identified (out of 67)**
1.Use evaluative and iterative strategies	
4 Assess for readiness and identify barriers and facilitators	25
5 Audit and provide feedback	11
56 Purposefully re-examine the implementation	13
26 Develop and implement tools for quality monitoring	7
27 Develop and organise quality monitoring systems	7
23 Develop a formal implementation blueprint	3
18 Conduct local needs assessment	8
61 Stage implementation scale up	5
46 Obtain and use patients/consumers and family feedback	3
14 Conduct cyclical small tests of change	2
2.Provide interactive assistance	
33 Facilitation	26
54 Provide local technical assistance	3
53 Provide clinical supervision	3
8 Centralise technical assistance	8
3.Adapt and tailor to context	
63 Tailor strategies	26
51 Promote adaptability	27
67 Use data experts	1
68 Use data warehousing techniques	1
4.Develop stakeholder interrelationships	
35 Identify and prepare champions	12
48 Organize clinician implementation team meetings	2
57 Recruit, designate, and train for leadership	9
38 Inform local opinion leaders	5
6 Build a coalition	19
47 Obtain formal commitments	9
36 Identify early adopters	7
17 Conduct local consensus discussions	4
7 Capture and share local knowledge	2
64 Use advisory boards and workgroups	9
65 Use an implementation advisor	2
45 Model and simulate change	0
72 Visit other sites	3
40 Involve executive boards	4
25 Develop an implementation glossary	2
24 Develop academic partnerships	11
52 Promote network weaving	25
5.Train and educate stakeholders	
19 Conduct ongoing training	30
55 Provide ongoing consultation	13
29 Develop educational materials	27
43 Make training dynamic	34
31 Distribute educational materials	31
71 Use train-the-trainer strategies	26
15 Conduct educational meetings	5
16 Conduct educational outreach visits	4
20 Create a learning collaborative	2
60 Shadow other experts	0
73 Work with educational institutions	5
6.Support clinicians	
32 Facilitate relay of clinical data to providers	3
58 Remind clinicians	0
30 Develop resource sharing agreements	7
59 Revise professional roles	21
21 Create new clinical teams	8
7.Engage consumers	
41 Involve patients/consumers and family members	3
39 Intervene with patients/consumers to enhance uptake and adherence	8

50 Prepare patients/consumers to be active participants	8
37 Increase demand	4
69 Use mass media	12
8.Utilise financial strategies	
34 Fund and contract for the clinical innovation	12
1 Access new funding	10
49 Place innovation on fee for service lists/formularies	4
2 Alter incentive/allowance structures	2
42 Make billing easier	1
3 Alter patient/consumer fees	0
70 Use other payment schemes	2
28 Develop disincentives	0
66 Use capitated payments	2
9.Change infrastructure	
44 Mandate change	3
12 Change record systems	14
11 Change physical structure and equipment	9
22 Create or change credentialing and/or licensure standards	8
13 Change service sites	15
9 Change accreditation or membership requirements	1
62 Start a dissemination organization	1
10 Change liability laws	0

*Implementation strategies in *Table 2* were obtained from Powell, B.J., Waltz, T.J., Chinman, M.J. et al. A refined compilation of implementation strategies: results from the Expert Recommendations for Implementing Change (ERIC) project. Implementation Sci 10, 21 (2015). https://doi.org/10.1186/s13012-01

Table 6. Implementation actions and corresponding strategies employed in included studies according to Waltz's implementation clusters and ERIC taxonomy strategies					
**Intervention/ Project name**	**Author(s), Year / Country**	**Actor**	**Action**	**Waltz's Implementation Cluster (1-9)**	**ERIC Implementation Strategy**
eHealth (n=15)					
Caring for Carers of People with Dementia study	Banbury et al. (2019) / Australia	Study team and IT support staff	IT support contacted participants individually to arrange test VC calls to ensure that their technology was satisfactory and that individuals were able to use Zoom VC software successfully (4)	1 - Use evaluative and iterative strategies	4
			They connected to a virtual room for meetings. Slides and videos provided information and were a tool to encourage group discussions where participants shared their knowledge and experiences (33)<br><br>Typically, the IT support person was required for the first 10 minutes of each meeting, after which they left. The facilitator would contact IT support by SMS during the meeting if any participants required calling by phone to resolve any problems (8 / 33)	2- Provide interactive assistance	33, 8
			Recruitment procedures were changed to enable verbal consent over the phone or traditional mail when returning the consent form when email was not possible (51 / 63)	3 – Adapt and tailor to context	51, 63
iSupport	Baruah et al. (2020) / India	-	-	-	-
Partner in Balance	Boots et al. (2017) / The Netherlands	Memory clinics, mental health organizations and caregiver support services<br><br>Actual care delivered through coaches	The participants' detailed input on the assignments enabled coaches to empathize with their situation and focus on their feedback. (5)<br><br>Their tasks were familiarizing participants with the online program, supporting them in module choice and goal setting, and giving feedback on the self-reflective assignments through the online messaging portal of the program, which was accessed via e-mail. (5)	1 - Use evaluative and iterative strategies	5
			Their tasks were familiarizing participants with the online program, supporting them in module choice and goal setting, and giving feedback on the self-reflective assignments through the online messaging portal of the program, which was accessed via e-mail. (33)	2- Provide interactive assistance	33
			The flexibility to provide feedback whenever and wherever via email was considered positive; it fitted their busy schedule and offered them the time to reflect on their words. (51)	3 - Adapt and tailor to context	51
			Caregivers were recruited from memory clinics (MUMC+,Elkerliek Hospital Helmond, Catharina Hospital Eindhoven), ambulatory mental health clinics (Virenze-RIAGG Maastricht, MET ggz Roermond), caregiver support services in the southern regions of the Netherlands, and the Dutch Alzheimer Association. (6, 52)<br><br>The caregivers were invited to participate by the clinician who treated their family member, were informed about the program's existence by the Dutch Alzheimer Association, or knew caregivers or family members already involved in the program. (52)	4 - Develop stakeholder interrelationships	6, 52
			Personal coaches were trained, experienced professionals (psychologist/psychiatric nurses) from a participating organization. (71)<br><br>They [personal coaches] attended a 2-hour training session in self-management techniques, goal setting and online help, and regular supervision meetings. (43, 16)<br><br>Their [personal coaches] tasks were familiarizing participants with the online program, supporting them in module choice and goal setting, and giving feedback on the self-reflective assignments through the online messaging portal of the program, which was accessed via e-mail.(19, 55)<br><br>Others reported difficulties fitting their answers into the assignment structure. Some participants mentioned using the website when they had time and often revisited the examples,	5 - Train and educate stakeholders	71, 43, 16, 55, 19, 31

			their own answers, or the feedback. (43, 31)<br><br>Coaches considered the **face-to-face intake session** crucial for developing a personal connection with the participant. (43)		
			Personal coaches were trained, experienced professionals (psychologist/psychiatric nurses) from a participating organization. (59)	6 - Support clinicians	59
			Others requested information based on editorials in health magazines, local newspapers, and information stands in the southern parts of the Netherlands. The Dutch Alzheimer Association disseminated information about the program via the following: (1) monthly meeting spots for people with dementia and their caregivers, (2) newsletters, and(3) their website and social media platforms, including Facebook and Twitter. (69)	7 - Engage consumers	69
			Others reported difficulties fitting their answers into the assignment structure. Some participants mentioned using the website when they had time and often revisited the examples, their own answers, or the feedback. (12)	9 - Change infrastructure	12
InLife	Dam et al. (2019) / the Netherlands	Community-based organization; DAZ conducted training	Inlife is an online social support platform for caregivers of PwD aiming to enhance positive interaction, involvement and social support. Inlife was developed in an iterative development process together with potential users, clinicians and web-designers. (46)	1 - Use evaluative and iterative strategies	46
			The platform consists of the following functionalities: Profile, Circles, Timeline, Calendar, Helping, Personal Messages, Care book and Compass. These functionalities provide opportunities to share care information, messages, pictures and requests for support. (8)	2- Provide interactive assistance	8
			Inlife is an online social support platform for caregivers of PwD aiming to enhance positive interaction, involvement and social support. Inlife was developed in an iterative development process together with potential users, clinicians and web-designers. (63)	3 - Adapt and tailor to context	63
			The platform consists of the following functionalities: Profile, Circles, Timeline, Calendar, Helping, Personal Messages, Care book and Compass. These functionalities provide opportunities to share care information, messages, pictures and requests for support. Participants could access the platform via (tablet) computer and smartphone by using a personal username and password. (31)	5 - Train and educate stakeholders	31
			For the RCT, 475 primary informal caregivers of PwDs were recruited via the Dutch Alzheimer Association (Alzheimer Nederland) and caregiver support services (e.g.day care centres, caregiver support groups), online advertisements(e.g., Facebook, online newsletters), written advertisements (e.g.,local newspapers), and via brochures distributed on regional memory and mental health clinics (69)	7 - Engage consumers	69
			The platform consists of the following functionalities: Profile, Circles, Timeline, Calendar, Helping, Personal Messages, Care book and Compass. These functionalities provide opportunities to share care information, messages, pictures and requests for support. Participants could access the platform via (tablet) computer and smartphone by using a personal username and password. (12)	9 - Change infrastructure	12
eMR-ABC	Frame et al. (2013) / USA	Regenstrief Institute's network (academic partners)	As a result, the eMR-ABC records all our patient encounters, allowing for more efficient tracking of patient interactions and permitting us to monitor progress in achieving patient and caregiver goals. Clinical providers are better equipped to adhere to a patient's visit schedule and to respond quickly (within 48-72 hours) to caregiver stress and acute care utilization events. (26)	1 - Use evaluative and iterative strategies	26
			We are currently exploring other automated data entry solutions, such as a mini-eMR-ABC application that clinical care providers can use via iPAD or Tablet PC and Web-based or iPAD applications that patients or family caregivers can use directly. (51)<br><br>The team translated the Healthy Aging Brain Care Monitor (HABC-M) and other forms into electronic versions to collect social and family history; medical and surgical history; a list of over-the-counter and prescribed medications; and caregiver burden and needs. (51)	3 - Adapt and tailor to context	51
			To date, we have successfully trained all 25 members of our clinical team at Wishard Health in the use of the software. (71)	5 - Train and educate stakeholders	71
			As a result, the eMR-ABC records all our patient encounters, allowing for more efficient tracking of patient interactions and permitting us to monitor progress in achieving patient and	6 - Support clinicians	32

			caregiver goals. Clinical providers are better equipped to adhere to a patient's visit schedule and to respond quickly (within 48-72 hours) to caregiver stress and acute care utilization events. (32)		
			As a result, the eMR-ABC records all our patient encounters, allowing for more efficient tracking of patient interactions and permitting us to monitor progress in achieving patient and caregiver goals. Clinical providers are better equipped to adhere to a patient's visit schedule and to respond quickly (within 48-72 hours) to caregiver stress and acute care utilization events. (12)	9 - Change infrastructure	12
Alzheimer's Caregiver Support Online (AlzOnline)	Glueckauf and Loomis (2003) / USA	AlzOnline staff	The primary focus of Phases 2 and 3 was the evaluation of website educational materials, caregiver classes, message board, and web links. (…) Toward the end of Phase 3, we incorporated reviewers' recommendations into the website (e.g., improving audio clarity) and the first generation of AlzOnline.net was ready to be rolled out to the public. (5, 41)<br><br>Although the bulk of our efforts was devoted to the development and evaluation of the website, an important goal for AlzOnline Year 1 was to establish a parallel, toll-free telephone service. As discussed previously, the primary objective of the toll-free telephone line was to provide easy and rapid access to dementia caregiver information and support. A sizeable minority of the dementia caregiver population, particularly older spouses and those who lived in rural areas, did not have access to the Internet, or if given the option, would prefer to receive educational services over the telephone rather than the Internet. Thus, it was essential to provide an alternative mode of service delivery for this subpopulation of caregivers. Furthermore, we needed to have a back up for our web-based programs. If AlzOnline.net experienced temporary transmission difficulties or if our server needed repair, toll-free telephone would ensure continuity of service during both unanticipated and planned down times. (4, 18)	1 - Use evaluative and iterative strategies	5, 46, 4, 18
			Caregivers were able to hear and see the facilitator (i.e., instructor) throughout the class, as well as to view simultaneously a slide show that emphasized the key points of the presentation. In addition, they had the option of communicating with one another and the facilitator using a chat box that was conveniently located at the bottom of the web page. (33)	2- Provide interactive assistance	33
			To reduce the demand for bandwidth from our dedicated AlzOnline server, we employed an application service provider (ASP) to deliver the secure, live classes, slide presentations and chat room. (51, 63)<br><br>Although the bulk of our efforts was devoted to the development and evaluation of the website, an important goal for AlzOnline Year 1 was to establish a parallel, toll-free telephone service. As discussed previously, the primary objective of the toll-free telephone line was to provide easy and rapid access to dementia caregiver information and support. A sizeable minority of the dementia caregiver population, particularly older spouses and those who lived in rural areas, did not have access to the Internet, or if given the option, would prefer to receive educational services over the telephone rather than the Internet. Thus, it was essential to provide an alternative mode of service delivery for this subpopulation of caregivers. Furthermore, we needed to have a back up for our web-based programs. If AlzOnline.net experienced temporary transmission difficulties or if our server needed repair, toll-free telephone would ensure continuity of service during both unanticipated and planned down times. (51)	3 - Adapt and tailor to context	51, 63
			During this six-month interval, AlzOnline staff members made regular telephone and face-to-face contacts with elder care organization personnel (e.g.,service directors and coordinators of the Area Agencies on Aging and their lead organizations) and caregiver advocates across Florida. We also made several presentations and conducted workshops at DOEA-sponsored events, such as their statewide caregiver forums, elder leadership institutes, and Alzheimer's Disease Initiative Advisory Committee meetings. (6, 38)	4 - Develop stakeholder interrelationships	6, 38
			The classes were bundled into three separate packages or modules of four to five classes each. These web-based materials were collectively labeled "Positive Caregiving" classes to emphasize the beneficial aspects of the caregiving experience, as well as to underscore the educational focus of the AlzOnline website. (29)	5 - Train and educate stakeholders	29, 43, 31

			Packages 1 and 2 (see Table 1) included one prerecorded class (i.e., Basics of Dementia), followed by five, 30- to 45-minute live classes, consisting of didactic presentations interspersed with group discussion and suggested outside activities. Packages 1 and 2 also were available live over a toll-free telephone line. The beta version of our first generation website was completed at the end of Phase 1 and consisted of three main areas. (43)<br><br>The first area contained our live, interactive, web-based Positive Caregiving classes. Caregivers were able to hear and see the facilitator (i.e., instructor) throughout the class, as well as to view simultaneously a slide show that emphasized the key points of the presentation**. In addition, they had the option of communicating with one another and the facilitator using a chat box that was conveniently located at the bottom of the web page.** (43)<br><br>The second area of the website was the library. This section provided caregivers with links to authoritative publications on dementia care, frequently asked questions about Internet-based communication, and a public message board. (31)<br><br>**The final area of the website contained links to helpful federal, state, and community resources**. We made a concerted effort to include links to community elder care organizations across Florida. These **senior service organizations play a critical role in the lives of both caregivers and care recipients with progressive dementia, providing frontline services and support in activities of daily living, attendant care, and crisis management.** (31)		
			Although the bulk of our efforts was devoted to the development and evaluation of the website, an important goal for AlzOnline Year 1 was to establish a parallel, toll-free telephone service. As discussed previously, the primary objective of the toll-free telephone line was to provide easy and rapid access to dementia caregiver information and support. A sizeable minority of the dementia caregiver population, particularly older spouses and those who lived in rural areas, did not have access to the Internet, or if given the option, would prefer to receive educational services over the telephone rather than the Internet. Thus, it was essential to provide an alternative mode of service delivery for this subpopulation of caregivers. Furthermore, we needed to have a back up for our web-based programs. If AlzOnline.net experienced temporary transmission difficulties or if our server needed repair, toll-free tele-phone would ensure continuity of service during both unanticipated and planned down times. (11)	9 - Change infrastructure	11
iGeriCare	Levinson et al. (2020) / Canada	research team from McMaster University	In this study, we performed a qualitative examination to identify recurrent themes, including facilitators and barriers, that might inform other organizations planning and implementation efforts with regard to web-based dementia caregiver education. (4)	1 - Use evaluative and iterative strategies	4
			We **targeted opinion leaders** who actively work with caregivers from a range of disciplines, including geriatrics, neurology, psychiatry, family medicine, and community care. (38)	4 - Develop stakeholder interrelationships	38
			iGeriCare consists of 10 multimedia e-learning lessons, curated resources, a series of weekly microlearning emails with small segments of content to reinforce material from the lessons and monthly web-streamed live events that allow participants to post questions to subject matter experts. (19, 31, 43)<br><br>iGeriCare has been designed to assist health care providers in providing high-quality education efficiently and effectively to caregivers of people living with dementia. We applied best practices in e-learning instructional design, such as the use of instructional graphics, audio narration, and personalization, which have been shown to be more effective than e-learning methods that do not conform to best-evidence instructional design. (29)	5 - Train and educate stakeholders	19, 31, 43, 29
Tele.TAnDem (iCBT intervention)	Meichsner et al. (2018) / Germany	Therapist / Clinical Psychologists	If the participant did not reply, a phone call was made to clarify if they were willing to continue participation and explore potential barriers. (33)<br><br>In each following message, the therapist conveyed her appreciation for the participant's fulfillment of caregiving tasks and validated all experiences. Against the background of viewing the caregiver as an expert for the own caregiving situation, the therapist initiated a	2- Provide interactive assistance	33

			search for solutions to problems (e.g., dealing with behavior problems or utilizing additional support). (33)		
			The intervention was delivered via a secure internet platform, the Tele.TAnDem.online blog. Participants received a personalized username and password that allowed them to log into a personal account where they could read messages from their therapist and post replies. (51)<br><br>However, caregivers also used the option to break up their session: 37.30% of participants reported to have taken a break during writing that ranged from a few minutes until 1.6 hr. Two-thirds of messages (66.67%) were sent outside the routine working hours (9 a.m. to 5 p.m.) and participants frequently wrote their messages between 9 p.m. and 1 a.m. (31.72% of messages were sent during that time). (51)	3 - Adapt and tailor to context	51
			The manual for the telephone-based Tele.TAnDem intervention was adapted to be delivered as a written intervention via the internet. The manual follows cognitive-behavioral principles and consists of 10 therapy modules. (29, 31)<br><br>Throughout the intervention period, the participant was asked to reply to the therapist's message within 4 days and could expect a reply within the next 3 days. Therefore, both participant and therapist wrote one message per week over a total planned intervention duration of 8 weeks. If a participant missed the deadline by >2 days, a follow-up message was sent asking about any problems with the response. (55)	5 - Train and educate stakeholders	29, 31, 55
			The intervention was delivered via a secure internet platform, the Tele.TAnDem.online blog. Participants received a personalized username and password that allowed them to log into a personal account where they could read messages from their therapist and post replies. (13)	9 - Change infrastructure	13
RAM (Remote Activity Monitoring)	Mitchell et al. (2017) published in (2020) / USA	director of nursing and technology services (DNT), a licensed vendor of the RAM technology. The DNT installed the RAM system and pro-vided caregivers with RAM instructions, maintenance, and support as needed.	Following completion of a baseline survey and random assignment to receive RAM for an 18-month period for free, dyads received a home visit from the director of nursing and technology services (DNT), a licensed vendor of the RAM technology. The DNT installed the RAM system and provided caregivers with RAM instructions, maintenance, and support as needed. (33)<br><br>Adapting the RAM system often involved a trial-and-error process of identifying the underlying problem and making changes accordingly. In the majority of cases, the process of adjustment was facilitated by staff support. Having a competent, proactive human being to talk to and troubleshoot was crucial for adjusting the system to fit users' needs. (54)	2- Provide interactive assistance	33, 54
			In addition, users can configure alerts based on unexpected or potentially dangerous activity. (51)	3 - Adapt and tailor to context	51
			The RAM system examined in this study includes six unobtrusive motion sensors (door sensors, motion sensors, a toilet flush sensor, and a bed mattress sensor) placed in the home to detect daily activity, as well as an emergency call pendant. (11)<br><br>By logging in, users can view charts that visually summarize the care recipient's activity. Users can view the last 60 days of data, as well as 24-hr snapshots of any one day's activity. (12)	9 - Change infrastructure	11, 12
Cuidate Cuidador	Pagan-Ortiz et al. (2014) / USA	self-help website with audio components and Ask an Expert function	To address the unmet educational and social support needs of Hispanic caregivers of persons with dementia, a website – Cuidate Cuidador – was developed and evaluated. (18)<br><br>Spanish-language content was developed for the website by native speakers from Puerto Rico and then translated into English, guided by a team with expertise in ADRD, caregiving, and elder populations that also worked with a web site developer to design a user-friendly website. (4)<br><br>This was achieved by making sure that the site was not overcrowded with text, topics could be easily accessed, and that the site included photographs of Hispanic caregivers. (4)	1 - Use evaluative and iterative strategies	18, 4
			Dementia experts need to be available to answer caregivers' questions within a 1- to 2-day time frame. A web designer and management team need to be available in case, for example, the web site has a technical glitch. Further, a team needs to be available to manage e-mail, and	2- Provide interactive assistance	8

			other user-generated content. In addition to the support staff mentioned, content creators are needed for the web site to stay fresh and relevant. (8)		
			The name directly translates to Caregiver, take care of yourself and the concept was based upon increasing computer trends of Hispanic online access. Cuidate Cuidador offers culturally competent information about ADRD, in both Spanish and English, practical how-to instructions on managing dementia-related behaviors and symptoms, real stories from caregivers, and information for caregivers on how to take care of themselves. (51,63)<br><br>The site also has an audio component that offers the option of listening to content, in either Spanish or English, for those who face literacy challenges. (51, 63)<br><br>Dementia experts need to be available to answer caregivers' questions within a 1- to 2-day time frame. A web designer and management team need to be available in case, for example, the web site has a technical glitch. Further, a team needs to be available to manage e-mail, and other user-generated content. In addition to the support staff mentioned, content creators are needed for the web site to stay fresh and relevant. (67, 8)	3 - Adapt and tailor to context	51, 63, 67
			Participants from Puerto Rico and Massachusetts were recruited via outreach strategies that included: letters, press releases, flyers, as well as phone calls to agencies in contact with caregivers. In Mexico, participants were recruited from a pool of caregivers who received social support services at a neurology teaching hospital. (52)	4 - Develop stakeholder interrelationships	52
			The name directly translates to Caregiver, take care of yourself and the concept was based upon increasing computer trends of Hispanic online access. Cuidate Cuidador offers culturally competent information about ADRD, in both Spanish and English, practical how-to instructions on managing dementia-related behaviors and symptoms, real stories from caregivers, and information for caregivers on how to take care of themselves. (29, 43, 31)<br><br>Other features include a comment section where caregivers can post and interact with other caregivers, an Ask an Expert resource section, information on national and international resources, and videos. CC aims to transform the current state of the typically isolated, home-based caregiver situation among Hispanic families, into a socially connected internet-based community. (43)<br><br>Spanish-language content was developed for the website by native speakers from Puerto Rico and then translated into English, guided by a team with expertise in ADRD, caregiving, and elder populations that also worked with a web site developer to design a user-friendly website. (29)	5 - Train and educate stakeholders	29, 43, 31
			The use of social media technology can provide support to caregivers in a variety of ways: improving knowledge and skills, reducing isolation, linking caregivers with each other, and with other resources needed for caregiving. (69)	7 - Engage consumers	69
mastery over dementia (MoD)	Pot et al. (2015) / The Netherlands	self-help guided support from coach (a psychologist with a training in CBT and experience with family caregivers of people with dementia)	After each lesson the caregiver is asked to send the finished homework exercises to the coach. If required, there is room to send a personal note as well. The coach provides feedback within three working days. (5)	1 - Use evaluative and iterative strategies	5
			Coach: MoD is a so-called guided self-help intervention, i.e. feedback is provided by a coach, because in face-to-face interventions this is an important ingredient. (33)	2- Provide interactive assistance	33
			The website of MoD is public domain, however, for participating in the intervention one needs a login. The public domain contains information for caregivers on dementia, caregiving, asking for help, and facts and figures. It also contains information on the intervention, costs, privacy, and how to register, and it shows a demo, self-test, and two short films of people who finished the intervention. (29, 43, 31)<br><br>Caregivers are advised to take one lesson per week in order to have enough time to practise and do the homework exercises. Reminders to login are sent by email when the caregiver does not login for three weeks. Access to the booster session is provided four weeks after finishing the eighth session. (19)	5 - Train and educate stakeholders	29, 43, 31, 55, 19

			Lessons and homework: MoD consists of eight lessons and a booster session with a summary of what has been taught. (55)		
			In MoD the coach is a psychologist with a training in CBT and experience with family caregivers of people with dementia. (59)	6 - Support clinicians	59
			Caregivers are advised to take one lesson per week in order to have enough time to practise and do the homework exercises. Reminders to login are sent by email when the caregiver does not login for three weeks. Access to the booster session is provided four weeks after finishing the eighth session. (39)	7 - Engage consumers	39
			All correspondence between caregiver and coach takes place within the secured, closed environment. There is no email exchange or telephone contact between them during the intervention. (12, 13)<br><br>Before opening a next lesson, the caregiver is asked to read the coach's feedback first. Only after opening and presumably reading the feedback, the caregiver gets access to the next lesson. Care diary: Caregivers are asked to fill out the care diary after each session. (...) In the care diary the caregiver describes the most striking event of the day, for example difficult behavior of the person with dementia, conflicts in the family about the care provided, or the inability to get professional support. (12)	9 - Change infrastructure	12, 13
iSupport	Teles et al. (2020) / Portugal	Research team from University of Porto	Good practices in digital engagement are embedded in iSupport, which allows the caregivers to personalise the educational plan according to idiosyncratic needs. (51)	3 - Adapt and tailor to context	51
			iSupport resorts to problem-solving and cognitive behavioural therapy techniques (Pot et al., 2019). Throughout 23 lessons, the programme covers well-established topics on dementia and caregiver support. (19)<br><br>By considering such encouraging evidence, the World Health Organization (WHO) developed "iSupport," a self-help online train- ing and support programme aimed at providing education and skills training to informal dementia caregivers (29, 31)	5 - Train and educate stakeholders	19, 29, 31
iSupport	Xiao et al. (2020) / Australia	Care staff of participating organisations	Adapting iSupport for use in an Australian context is an opportunity to address the gap in dementia education for informal caregivers and assist them to better utilise care services. (51, 63)	3 - Adapt and tailor to context	51, 63
			To facilitate the adaptation of the iSupport programme, the WHO has built an online generic version and is accessible by users via the website: https://www.isupportfordementia.org/en(WHO, 2019a). A booklet of the iSupport for Dementia has also been published for caregivers who are unable to accessor do not use the internet. (29)	5 - Train and educate stakeholders	29
Partner in Sight	van Knippenberg et al. (2017) / The Netherlands	Clinicians and case managers facilitated recruitment; feedback sessions facilitated by personal coach	Every two weeks participants received ESM-derived feedback from a personal coach (psychologist) according to standardized protocol. The standardized protocol was based on a previous study that used ESM-derived feedback on positive affect in persons with depression and it was adapted to the context of caregivers of PwD (51, 63)	3 - Adapt and tailor to context	51, 63
			Feedback was provided both graphically and verbally and contained information on experienced levels of positive affect and their relationship with daily activities and social interactions in daily life. (43)<br><br>Before the start of the study, all coaches received training with detailed instructions on how to provide the feedback. (71)	5 - Train and educate stakeholders	19, 71, 43
			Participants were encouraged to implement new insights into their daily lives. (50)	7 - Engage consumers	50
			During the six-week intervention period, the PsyMate was programmed to generate 10 alerts (sound and vibration) per day for three consecutive days per week (6*10*3 = 180 beeps in total). Alerts were emitted at random intervals between 7:30 AM and 10:30 PM. (12)<br><br>The 'PsyMate', a palmtop, was used to digitally collect momentary assessments and to provide visualized feedback on daily life situations that elicit positive emotions (11)	9 - Train and educate stakeholders	11, 12

FamTechCare	Williams et al. (2020) / USA	The interventionist was either a nurse or social worker with a master's degree, who was also a member of the expert team.	The uploaded video recordings were all screened by a designated team member within 24-hours of submission to ensure there were no immediate safety concerns and to identify the portions of videos for weekly expert review. (26)	1 - Use evaluative and iterative strategies	26
			The uploaded video recordings were all screened by a designated team member within 24-hours of submission to ensure there were no immediate safety concerns and to identify the portions of videos for weekly expert review. (33)<br><br>FamTechCare is a multicomponent telehealth intervention engaging family caregivers of persons living with dementia in video recording challenging care situations and behaviors. Caregivers Submit videos that are reviewed by an interdisciplinary team who provide tailored feedback to improve care. (33)	2- Provide interactive assistance	8, 33
			Along with an individual training session in the caregiver's home on the use of the VMU, the caregiver received a binder with detailed instructions, including step-by-step images, as a resource for the VMU. The innovative video recording application, Behavior Capture (https://behaviorimaging.com, Boise, ID), utilizes buffering technology that captures events before the recording is initiated. (43, 31)	5 - Train and educate stakeholders	31, 43
			The Interdisciplinary video review team included research and clinical professionals from the fields of nursing, geriatric psychiatry, social work, and psychology. (21)<br><br>The interventionist was either a nurse or social worker with a master's degree, who was also a member of the expert team. (59)	6 - Support clinicians	21, 59
			Dementia caregivers were provided with a telehealth video monitoring unit(VMU) that includes an iPad mini with the video recording application, a Bluetooth remote, and an iPad stand. (11)	9 - Change infrastructure	11
Respite Care (n=5)					
Adult day care - On Lok project / Program of All-Inclusive Care for the Elderly (PACE)	Beisecker et al. (1996) / USA	-	-	-	-
Caring for the Caregiver	Brandao et al. (2016) / Portugal	-	-	-	-
Adult day services (ADS) Blue Lake Adult Day Center (BLADC) and Century Adult Day Services (CADS)	Gaugler (2014) / USA	Blue Lake Adult Day Center (BLADC) and Century Adult Day Services (CADS)	In order to achieve the sometimes precarious balance between client preference and client need, staff had to assume multifaceted care roles including staff as "serving"(e.g., preparing and serving food with an eye toward hospitality), working together collaboratively, provision of intensive activity of daily living care, and offering flexible care to meet the needs of clients as well as family caregivers. (59)	6 - Support clinicians	59
			The process of use and service delivery within ADS also interacted with the role of family members; families' main engagement with ADS appeared to largely revolve around preparing the relative to attend ADS (which posed a number of challenges) as well as balancing ADS with other community-based services. (39, 50)	7 - Engage consumers	39, 50
			BLADC is affiliated with a local nursing home operator but is physically located in a nearby church. (13)<br>CADS is also not-for-profit, has been in operation since 1990, and served 53 clients. CADS cost clients approximately $83 per day of attendance and is not affiliated with a long-term care operator. CADS occupies space in a former community hospital which has been transformed to include CADS (which is located in the former nurses' station area), a nursing home, and several other community organizations. (13)	9 - Change infrastructure	13
Adult day service Plus (ADS Plus)	Gitlin et al. (2019) / USA	30 program sites; ADS sites - on site staff	Phase I (months 1–3) is the most intensive involving up to 8 sessions of approximately one hour each. These sessions are preferably con-ducted in-person and at times convenient to caregivers (e.g., at drop off or pick up of the client or at another time that works for them and the staff interventionist). (51-G3) The First two sessions occur within a two-week timeframe and involve focused interviewing and systematic assessment of caregiver needs and challenges. (4, 18)	1 - Use evaluative and iterative strategies	4, 5, 18, 23, 26, 56

			An investigator developed tool, Caregiver Assessment of Management Problems–Revised (CAMP-R), asks caregivers their level of difficulty (a lot difficult, somewhat difficult, not difficult at all) in managing each identified area from a list reflecting three domains: daily basic and instrumental activities of daily living of the client, behavioral symptoms, and caregiver-centered concerns (e.g., own health, home safety, respite needs, managing other responsibilities and so forth). (26)<br><br>The assessment process provides a roadmap from which a "care" plan is developed that includes: n1) identification of the 3 to 5 specific problem areas to work on and their priority order; 2) schedule of contacts for next 3 months; 3) agreed upon mechanisms for working together (face-to-face; email; telephone) and 4) type of education materials and referrals and linkages that will be needed. (23)<br><br>Sessions three through eight occur approximately two weeks apart over the 3-month period. Each of these contacts involve the following components: 1) review of stress reduction techniques; 2) education related to identified problem areas; 3) referral and linkage if needed to address targeted problem areas; 4) problem solving and brainstorming regarding targeted problem areas and the creation of the ADS Plus Prescription for each care challenge that lists key strategies tailored to the problem area; 5) review of the prescriptive strategies provided in the previous sessions; 6) reinforcement of taking care of self as a priority; and 7) on-going validation and support. (5)<br><br>At the conclusion of 3 months (8th session), a reassessment is conducted to determine whether each targeted problem area has been resolved (level of difficulty managing), as well as caregiver upset and confidence (1 = no confidence to 10 = a lot of confidence). Caregivers also receive the Gitlin and Piersol Caregiver Guide to Managing Dementia (2014) that provides checklists of nonpharmacological strategies for common behavioral and psychological symptoms along with helpful information about using activities and managing common health challenges (pain, hydration, constipation and so forth). (5, 56)<br><br>During these sessions, strategies offered previously are reinforced, education and support are continuously provided, and new challenges are identified and tackled following a similar approach as in the first three months (brainstorming, problem-solving, education, ADSPlus prescription, practice of new strategies). At six months, a re-assessment is conducted to determine if targeted problem areas have been resolved, their level of difficulty, as well as the caregiver's level of upset and confidence with each targeted area as well as for new problem areas that may have emerged. (5, 56)<br><br>In Phase III of ADS Plus (months 7–12), the interventionist follow up with the caregiver on a monthly basis, either in person, by tele-phone, or email to determine how the caregiver is managing and if new challenges have emerged that he/she wishes to address. Caregivers are also encouraged to contact the staff interventionist to schedule an appointment during this time if needed. At 12 months (conclusion of program), a reassessment is conducted to determine if targeted problem areas identified throughout the year are resolved, and level of difficulty, upset and confidence with each. (5)		
			Also, in these first two sessions, basic stress reduction techniques are provided to and practiced with the caregiver. (33)<br><br>Sessions three through eight occur approximately two weeks apart over the 3-month period. Each of these contacts involve the following components: 1) review of stress reduction techniques; 2) education related to identified problem areas; 3) referral and linkage if needed to address targeted problem areas; 4) problem solving and brainstorming regarding targeted	2- Provide interactive assistance	33

			problem areas and the creation of the ADS Plus Prescription for each care challenge that lists key strategies tailored to the problem area; 5) review of the prescriptive strategies provided in the previous sessions; 6) reinforcement of taking care of self as a priority; and 7) on-going validation and support. (33)<br><br>In Phase III of ADS Plus (months 7–12), the interventionist follow up with the caregiver on a monthly basis, either in person, by tele-phone, or email to determine how the caregiver is managing and if new challenges have emerged that he/she wishes to address. Caregivers are also encouraged to contact the staff interventionist to schedule an appointment during this time if needed. At 12 months (conclusion of program), a reassessment is conducted to determine if targeted problem areas identified throughout the year are resolved, and level of difficulty, upset and confidence with each. (33)		
			ADS Plus consists of five key components: taking care of self, education, validation and support, referral and linkage, identification of care challenges, skill building and strategies. Each component is tailored to caregiver-identified unmet needs and challenges in providing care at home. (63)	3 - Adapt and tailor to context	63
			Each participating site assigns a designated staff member to serve as research liaison. (35, 57)	4 - Develop stakeholder interrelationships	35, 57
			The research liaisons participate in 4 h of paid training(via webinar) to learn about study procedures, scripts for describing the study and outreach efforts to their family caregivers that are ethical and not coercive. (71, 15)<br><br>Training consists of readings, viewing 16 brief videos with each describing a component of the intervention and two webinars of two-hour duration each followed by monthly coaching calls upon implementing the program. (43, 29)<br><br>The assessment process provides a roadmap from which a "care" plan is developed that includes: n1) identification of the 3 to 5 specific problem areas to work on and their priority order; 2) schedule of contacts for next 3 months; 3) agreed upon mechanisms for working together (face-to-face; email; telephone) and 4) type of education materials and referrals and linkages that will be needed. (31)<br><br>Also, in these first two sessions, basic stress reduction techniques are provided to and practiced with the caregiver. (19)<br><br>Sessions three through eight occur approximately two weeks apart over the 3-month period. Each of these contacts involve the following components: 1) review of stress reduction techniques; 2) education related to identified problem areas; 3) referral and linkage if needed to address targeted problem areas; 4) problem solving and brainstorming regarding targeted problem areas and the creation of the ADS Plus Prescription for each care challenge that lists key strategies tailored to the problem area; 5) review of the prescriptive strategies provided in the previous sessions; 6) reinforcement of taking care of self as a priority; and 7) on-going validation and support. (19)<br><br>At the conclusion of 3 months (8th session), a reassessment is conducted to determine whether each targeted problem area has been resolved (level of difficulty managing), as well as caregiver upset and confidence (1 = no confidence to 10 = a lot of confidence). Caregivers also receive the Gitlin and Piersol Caregiver Guide to Managing Dementia (2014) that provides checklists of nonpharmacological strategies for common behavioral and psychological symptoms along with helpful information about using activities and managing common health challenges (pain, hydration, constipation and so forth). (19)<br><br>In Phase II (months 4–6), the interventionist follows up with the caregiver every other week for up to six occasions either in person or by telephone or email depending upon the caregiver's needs and preferences. (43)	5 - Train and educate stakeholders	71, 15, 19, 43, 29, 31

			During these sessions, strategies offered previously are reinforced, education and support are continuously provided, and new challenges are identified and tackled following a similar approach as in the first three months (brainstorming, problem-solving, education, ADS Plus prescription, practice of new strategies). At six months, a re-assessment is conducted to determine if targeted problem areas have been resolved, their level of difficulty, as well as the caregiver's level of upset and confidence with each targeted area as well as for new problem areas that may have emerged. (19)<br><br>In Phase III of ADS Plus (months 7–12), the interventionist follow up with the caregiver on a monthly basis, either in person, by tele-phone, or email to determine how the caregiver is managing and if new challenges have emerged that he/she wishes to address. Caregivers are also encouraged to contact the staff interventionist to schedule an appointment during this time if needed. At 12 months (conclusion of program), a reassessment is conducted to determine if targeted problem areas identified throughout the year are resolved, and level of difficulty, upset and confidence with each. (43)		
			Each participating site assigns a designated staff member to serve as research liaison. (59)	6 - Support clinicians	59
			Additionally, we have developed incentives for sites to continuously engage in outreach to families. This includes offering $500 to the first site from which 15 caregivers are enrolled, $50 for every 3 participants enrolled per site, the provision of ongoing coaching calls to share effective recruitment outreach activities, and a quarterly newsletter reporting enrollment progress. (2)	8 - Utilize financial strategies	2
Adult Day Care (Respite Programming)	Roberts and Struckmeyer (2017) / USA	-	-	-	-
Psychoeducation (n=12)					
The booklet, Information for Families and Friends of People with Severe and End Stage Dementia (Palliative Care Demen-tia Interface: Enhancing Community Capacity	Chang et al. (2010) / Australia	Dementia advisory services and Residential Aged care Facilities	The booklet, Information for Families and Friends of People with Severe and End Stage Dementia (Palliative Care Dementia Interface: Enhancing Community Capacity Project, 2006), formed the fundamental material of this project (see Table 1). To evaluate its application and usage, a questionnaire was purposely designed for this project. The questionnaire development process included a review of the existing literature and similar tools and further key participant consultation to address issues of validity. (4)	1 - Use evaluative and iterative strategies	4
			This project utilized a two-stage recruitment process to access family caregivers. In the first instance, services and facilities that dealt with family caregivers were approached to participate. (52)<br><br>Second, family caregivers who met the inclusion and exclusion criteria were contacted and subsequently recruited via the participating services and facilities (36)	4 - Develop stakeholder interrelationships	36, 52
			The booklet, Information for Families and Friends of People with Severe and End Stage Dementia (Palliative Care Dementia Interface: Enhancing Community Capacity Project, 2006), formed the fundamental material of this project (see Table 1). To evaluate its application and usage, a questionnaire was purposely designed for this project. The questionnaire development process included a review of the existing literature and similar tools and further key participant consultation to address issues of validity. (29)	5 - Train and educate stakeholders	29
START (STrAtegies for RelaTives)	Foley et al. (2020) / UK	Clinical service for LBD carers	We adapted START manuals by modifying the vignettes and psychoeducation to focus on the cognitive and neuropsychiatric symptoms characteristic of LBD, rather than episodic memory impairment. (63, 51)	3 - Adapt and tailor to context	63, 51
			A psychology graduate was appointed, trained and supervised by a clinical neuropsychologist. (35)	4 - Develop stakeholder interrelationships	35
			STrAtegies for RelaTives (START) is an 8-week, manualised, individual psychological intervention designed for carers of people with dementia. (19)	5 - Train and educate stakeholders	19, 43

			Five carers chose to have all sessions by telephone, four carers chose to have them in person, and one chose a combination of both. (43)<br><br>In light of LBD carers' increased burden, and in contrast with the original START programme, we also offered START over the telephone as an alternative to the carer having to leave the patient to attend our centre.(43)		
			We piloted the use of START in a clinical service for LBD carers for 2.5 months using local funding. (1)	8 - Utilize financial strategies	1
			In light of LBD carers' increased burden, and in contrast with the original START programme, we also offered START over the telephone as an alternative to the carer having to leave the patient to attend our centre. (13)	9 - Change infrastructure	13
START (STrAtegies for RelaTives)	Sommerland et al. (2014) / UK	The intervention was delivered by psychology graduates without clinical qualifications as a face-to-face, individual intervention at a location chosen by the carer, usually their home	The Sessions consisted of psychoeducation about dementia, carer stress and access to emotional support; exploration of behaviours or situations that the carer found difficult and potential management strategies; challenging unhelpful thoughts; relaxation techniques accompanied by CDs of relaxation exercises; communication skills; planning pleasurable activities; future planning and maintaining skills learnt. The carers were also given homework to complete and a manual of the interventions in which to record their work. The participants kept the CD and manual to allow their continued use. (43, 31)	5 - Train and educate stakeholders	43, 31
			The intervention was delivered by psychology graduates without clinical qualifications as a face-to-face, individual intervention at a location chosen by the carer, usually their home.	9 - Change infrastructure	13
Tele-Savvy for Dementia Caregivers / The Savvy Caregiver Program	Griffiths et al. (2015) / USA	Department of Veterans Affairs–supported clinical T-21 Non-Institutional Alternatives to Long Term Care clinical demonstration project.<br><br>Staff in research service (not clinical staff, thus affecting the real-world sustainment)	Home visits were conducted to deliver the iPads and class materials (manual, workbook, and journal) and conduct the baseline interview. (4)<br><br>Caregivers were taught and practiced how to access their daily lessons and how to participate in the weekly tele-video conference group calls on the iPad. Within 2 weeks after program completion, a project coordinator returned to participants' homes to collect the iPads and conduct follow-up interviews. (46)	1 - Use evaluative and iterative strategies	4, 46
			We selected con-tent for the videoconferences that would be best presented in real time, to encourage and allow for caregiver responses and questions. During these group sessions, we conducted, for example, a guided imagery exercise designed to develop caregivers' appreciation for the central role of confusion in the lives of persons with dementia. We use character vignettes to illustrate the progression of the disease through various stages. The group provides an effective venue for brainstorming strategies for guiding a person through activities of daily living or for designing tasks or activities to keep the person contentedly involved during the day. (33)	2- Provide interactive assistance	33
			These six modules (on self-care practices such as therapeutic breathing and guided meditations) draw upon evidence-based practice and were taught by experienced practitioners. The hour-long weekly videoconferences were devoted, in roughly equal measure, to caregiver self-reports of homework activities, with facilitator coaching (to promote mastery), addressing questions that may have arisen from the video modules, and introduction to new content. (19)<br><br>The hosts closed each day's "lesson" by reiterating its take-away message. In segmenting the Savvy curriculum, we drew on prior experiences to add content to the program's coverage of family and informal care and caregiver self-care. We incorporated an explicit use of the nursing process (Assess–Plan–Implement–Evaluate) as the general framework for savvy caregiving and a three-segment component on formal and informal resource mapping with the existing component on strengthening family resources for caregiving (Samia, Hepburn, & Nichols, 2012). Building on the original pro-gram's attention to the emotional impact of caregiving on the caregiver, we added weekly self-care video modules developed for use in an ongoing VA caregiving study to the curriculum. (29)<br><br>Through vignettes at the family home or the favorite restaurant, we sought to illustrate the losses in cognition, emotional and behavioral control, and the ability to perform everyday	5 - Train and educate stakeholders	19, 31, 43, 29

			tasks that are produced by the disease across the progressive stages of a dementing illness. The vignettes also portrayed effective caregiving strategies in these everyday situations. (43)<br><br>This segmentation allowed us to envision a 6-week structure that combined stand-alone daily internet-delivered video modules (six per week) with weekly group videoconferences (one per week throughout the 6 weeks). For the daily video modules, we adopted a two-host talk show format. From behind the hosts' desk, we delivered, each day, a brief (6–13 minute) lesson on a single topic. This didactic approach (linked to the caregiver manual and accompanied with slides) was enacted in the lives of a fictional dementia family (portrayed by trained amateur actors). (19, 31, 43)		
			Caregivers were trained on the use of the iPad technology during and immediately following this visit. (50)	7 - Engage consumers	50
			For the implementation project, Tele-Savvy was delivered on iPads loaned to participants. (11)	9 - Change infrastructure	11
Tele-Savvy for Dementia Caregivers / The Savvy Caregiver Program	Kovaleva et al. (2017) / USA	Intervention was delivered online instructor (professional role unclear)	Tele-Savvy is based on social cognitive (Bandura, 1977) and stress and coping theories (Lazarus & Folkman, 1984). Tele-Savvy reformatted the in-per-son Savvy Caregiver Program's curriculum into a 7-week program delivered synchronously and asynchronously to groups of four to eight caregivers. (63)	3 - Adapt and tailor to context	63
			Tele-Savvy is based on social cognitive (Bandura, 1977) and stress and coping theories (Lazarus & Folkman, 1984). Tele-Savvy reformatted the in-per-son Savvy Caregiver Program's curriculum into a 7-week program delivered synchronously and asynchronously to groups of four to eight caregivers. (19)<br><br>The synchronous portion included weekly scheduled videoconferences (60-80 min) that served as an online classroom in which instructors led lectures and discussions and provided a venue for caregivers' interactions and sharing of their experiences. (19, 55)<br><br>The lessons' didactic messages are delivered through expert presentations that are usually augmented by vignettes enacted by amateur actors playing a "caregiving family" in various dementia-stage-specific caregiving situations and using caregiving strategies in familiar settings. Caregivers can watch the lessons whenever and as often as they wish. A 200+ page Tele-Savvy Caregiver Manual served as a reference, and "non-mandatory homework" was assigned, which, following principles of social cognitive theory (Bandura, 1986), was meant to enhance caregivers' self-efficacy by asking them to apply what they were learning and to experience success in doing so. (29, 43, 31)	5 - Train and educate stakeholders	19, 55, 29, 43, 31
ANSWERS	Judge et al. (2010) / USA	Intervention specialists	At the beginning of each session, the previous session's action plan was used to assess how each skill was implemented and discuss barriers encountered. (26, 27)<br><br>To ensure fidelity to the protocols, intervention specialists received supervision and feedback when initially working with dyads. (5)<br><br>On-going monitoring of ANSWERS was accomplished through biweekly conference calls, which allowed for discussion of cases and review of protocols, including length and content of sessions, modifying techniques, and working with dyads. (56)	1 - Use evaluative and iterative strategies	26, 27, 5, 56
			This distinguishing feature of the protocol was important because it allowed dyads with diverse care needs (i.e., communication and stress) or characteristics (i.e., level of impairment and living arrangement) flexibility and time to select and practice skills. (51)<br><br>Additional features that were thought to lead to the success of ANSWERS include (a) implement-ing the program in dyads' homes, (b) scheduling sessions around dyads' preferences and schedules, and (c) providing dyads with easy-to-use materials (i.e., intervention manuals and activity notebook) and individualized external memory aids. (51)	3 - Adapt and tailor to context	51
			Intervention specialists (n = 4) held at least a master's degree in a counseling-related field and had prior training and experience in implementing traditional counseling techniques	4 - Develop stakeholder interrelationships	57

			that ranged from working with children and families to adults and older adults. For this project, intervention specialists received extensive training in the following areas: memory and cognitive processes, symptoms of dementia, implementing cognitive rehabilitation techniques, and working with CGs and CRs as a dyad. Intervention specialists received a total of 22 hr of initial training consisting of lecture, model-ing, role playing, case examples, feedback, and discussion. (57)		
			The ANSWERS protocol consisted of six 90-min curriculum-guided sessions between an intervention specialist, the primary family CG, and the CR. (19)<br><br>Dyads, in consultation with the intervention specialist, selected a core set of skills to practice each session that addressed the dyad's specific care needs and issues. Each of the six intervention sessions built upon the previous session, allowing dyads to become more experienced and comfortable with each technique. (19)<br><br>After each session, an action plan was used to list each chosen skill, along with how and when each skill would be practiced. Dyads were asked to keep the action plan in a prominent location and to document any difficulties they experienced when practicing skills. (31)<br><br>Intervention specialists (n = 4) held at least a master's degree in a counseling-related field and had prior training and experience in implementing traditional counseling techniques that ranged from working with children and families to adults and older adults. **For this project, intervention specialists received extensive training in the following areas: memory and cognitive processes, symptoms of dementia, implementing cognitive rehabilitation techniques, and working with CGs and CRs as a dyad. Intervention specialists received a total of 22 hr of initial training consisting of lecture, modeling, role playing, case examples, feedback, and discussion.** (71)	5 - Train and educate stakeholders	19, 31, 71
			Additional features that were thought to lead to the success of ANSWERS include **(a) implementing the program in dyads' homes,** (b) scheduling sessions around dyads' preferences and schedules, and (c) providing dyads with easy-to-use materials (i.e., intervention manuals and activity notebook) and individualized external memory aids. (13)	9 - Change infrastructure	13
REACH II	Lykens et al. (2014) / US	The Alzheimer's Association Caregiver Education and Counseling REACH II staff consisted of two dementia care specialists (counselors), who received training in the REACH II program implementation.	The REACH II counselors assessed the caregivers at home and followed up in-person and by telephone counseling, covering topics including home safety, feelings and stress management, behavioral skills training, and provided additional resources in conjunction with material from caregiver notebooks provided by the REACH II program which were tailored to specific needs of the family. (4)<br><br>The resource notebook was used when the counselors visited or contacted the caregivers by phone. The notebook was composed of sections covering each of the areas addressing the quality of life indicators. The sections included pictures and information with particular attention to the needs identified by the caregivers as their priorities. (26, 27)	1 - Use evaluative and iterative strategies	4, 26, 27
			Referral sources to the program included a 24/7 telephone helpline, support groups, case managers, the Aging & Disabilities Resource Center (ARDC), partnering agencies, and home health agencies were utilized to recruit program participants. (54, 8)	2- Provide interactive assistance	54, 8
			The REACH II staff made modifications in the delivery of REACH II services to accommodate program implementation in the community setting. One example was that in order to resolve communication barriers with families by changing terminology in interactions substituted'' dementia care specialists'' for ''counselors''. A professionally printed caregiver notebook in Spanish was developed based on a former program in Pittsburgh, PA, to enable program delivery to Hispanic families. (51)	3 - Adapt and tailor to context	51
			The United Way of Tarrant County contracted with the Alzheimer's Association to implement the REACH II program as a component of support services to Alzheimer families. (52, 47, 6)<br><br>**Referral sources to the program included a 24/7 telephone helpline, support groups, case**	4 - Develop stakeholder interrelationships	52, 47, 6

			**managers, the Aging & Disabilities Resource Center (ARDC), partnering agencies, and home health agencies were utilized to recruit program participants.** (52)		
			The resource notebook was used when the counselors visited or contacted the caregivers by phone. The notebook was composed of sections covering each of the areas addressing the quality of life indicators. The sections included pictures and information with particular attention to the needs identified by the caregivers as their priorities. (31)<br><br>The REACH II counselors assessed the caregivers at home and followed up in-person and by telephone counseling, covering topics including home safety, feelings and stress management, behavioral skills training, and provided additional resources in conjunction with material from caregiver notebooks provided by the REACH II program which were tailored to specific needs of the family. (31)<br><br>The workshop included training on the content of the resource book, role playing, and discussion of proper responses to various situations raised by caregivers. (43)<br><br>The counselors were trained during a two day workshop offered by a co-investigator of the original REACH development and implementation team. (71)<br><br>The United Way of Tarrant County contracted with the Alzheimer's Association to implement the REACH II program as a component of support services to Alzheimer families. (73)	5 - Train and educate stakeholders	73, 71, 43, 31
			The Alzheimer's Association Caregiver Education and Counseling REACH II staff consisted of two dementia care specialists (counselors), who received training in the REACH II program implementation. (21, 59)	6 - Support clinicians	21, 59
			The resource notebook was used when the counselors visited or contacted the caregivers by phone. The notebook was composed of sections covering each of the areas addressing the quality of life indicators. The sections included pictures and information with particular attention to the needs identified by the caregivers as their priorities. (12)	9 - Change infrastructure	12
REACH into Indian Country	Martindale-Adam et al. (2017) / USA	Tribal communities - HIS Clinical support staff (public health nurses)	In addition, REACH implementation has been added to staff performance evaluations and instituted as a quality improvement project. (26)<br><br>With positive feedback from public health nurses and caregivers of veterans came requests to provide the program to non-veteran caregivers. A partnership of IHS, primarily the Division of Nursing Services, the Administration on Community Living (ACL) through the Administration on Aging's Native American Caregiver Support Services Program, and the University of Tennessee Health Science Center (UTHSC), was formed to implement REACH for caregivers of persons with dementia regardless of veteran status. (61)<br><br>REACH VA materials, with permission from the VA, were modified. Photographs for the Caregiver Notebook and REACH Coach manual now reflect only AI/AN diversity and not the broader diversity of the VA.(56)	1 - Use evaluative and iterative strategies	61, 56, 26
			Community practitioners who are serving as REACH Coaches have presented talks on Alzheimer's and dementia symptoms to help caregivers recognize that dementia symptoms are not normal aging. (33)	2- Provide interactive assistance	33
			More training time was allotted to review stress management techniques, and electronic handouts will be made available to all participants in future training sessions. A brief video of certified REACH staff sharing their experience in training and the benefits of the program will be used during the training introduction to increase the level of comfort and acceptance of new trainees. (63)<br><br>As REACH has been implemented, staff needs and suggestions have reframed the program. This cultural targeting recognizes and reinforces community values, beliefs, and behaviors and	3 - Adapt and tailor to context	51, 63

			builds on them to provide context and meaning to the health message of support for the caregiver. (51)		
			In 2013, exploration of REACH into Indian County began when IHS and the VA Caregiver Support Program and its Memphis VA Caregiver Center began to train IHS and Tribal public health nurses in the modified four session REACH VA intervention to work with caregivers of AI/AN veterans. (72)<br><br>With positive feedback from public health nurses and caregivers of veterans came requests to provide the program to non-veteran caregivers. A partnership of IHS, primarily the Division of Nursing Services, the Administration on Community Living (ACL) through the Administration on Aging's Native American Caregiver Support Services Program, and the University of Tennessee Health Science Center (UTHSC), was formed to implement REACH for caregivers of persons with dementia regardless of veteran status. (6)<br><br>As for any program offered for implementation in AI/AN communities, Tribal organizations have control over whether the program is accepted and how it is implemented. (40)<br><br>For one Coach and caregiver, a solution to family and tribal buy-in involved publicly identifying the caregiver's older brother, a Tribal elder, as a caregiver involved in REACH. This designation provided validation and credibility for the program and the sister's participation. (35)<br><br>In response to requests for additional information and post-training guidance, the Caregiver Center holds Audio Chats on a monthly basis to discuss implementation of the program. Audio Chats participants have identified the need for a refresher course to help participants gain a better understanding of how to get into the community and suggested implementing a volunteer Marketing/Strategy Committee to help with publicizing the program to local caregivers. (35)	4 - Develop stakeholder interrelationships	72, 6, 40, 35
			In 2013, exploration of REACH into Indian County began when IHS and the VA Caregiver Support Program and its Memphis VA Caregiver Center began to train IHS and Tribal public health nurses in the modified four session REACH VA intervention to work with caregivers of AI/AN veterans. (71)<br><br>REACH VA materials, with permission from the VA, were modified. Photographs for the Caregiver Notebook and REACH Coach manual now reflect only AI/AN diversity and not the broader diversity of the VA. (29)<br><br>Although this model is still being used, states and Tribes have requested large, in-person trainings followed by on-site certifications. These face-to-face trainings allow staff to interact with the Caregiver Center Coordinator in a more personal way and to interact and share with each other about strategies to deliver the program. Watching certifying role plays provides additional examples of both concerns that may occur and how other staff may handle these concerns. (71)<br><br>Similar to other REACH implementers, staff report enthusiasm for teaching skill building techniques, such as problem solving and mood management, delving into the Caregiver Notebook, and helping caregivers with strategies for self-care. (31)<br><br>In response to requests for additional information and post-training guidance, the Caregiver Center holds Audio Chats on a monthly basis to discuss implementation of the program. Audio Chats participants have identified the need for a refresher course to help participants gain a better understanding of how to get into the community and suggested implementing a volunteer Marketing/Strategy Committee to help with publicizing the program to local	5 - Train and educate stakeholders	71, 29, 31, 15

			caregivers. (15)<br><br>More training time was allotted to review stress management techniques, and electronic handouts will be made available to all participants in future training sessions. A brief video of certified REACH staff sharing their experience in training and the benefits of the program will be used during the training introduction to increase the level of comfort and acceptance of new trainees. (71)		
			REACH Coaches are asked to provide information on their delivery of the program, including topics discussed, to determine fidelity. (30)	6 - Support clinicians	30
			With positive feedback from public health nurses and caregivers of veterans came requests to provide the program to non-veteran caregivers. A partnership of IHS, primarily the Division of Nursing Services, the Administration on Community Living (ACL) through the Administration on Aging's Native American Caregiver Support Services Program, and the University of Tennessee Health Science Center (UTHSC), was formed to implement REACH for caregivers of persons with dementia regardless of veteran status. (37)<br><br>The Marketing/Strategy Committee initially identified two areas of focus. The first is to assist REACH Coaches in recruitment using strategies and language that will resonate with AI/AN dementia caregivers. For example, a REACH Coach participated in a local radio program providing information on Alzheimer's and dementia and highlighted REACH as a community resource available for family caregivers. Coaches have requested a publicity brochure for distribution throughout the community. (69)<br><br>The Marketing/Strategy committee is working on developing a brochure template that can be customized to a community. This brochure could be used to encourage primary care providers to make referrals of caregivers who are in need of help, encourage IHS, Tribal, and AoA/ACL staff to become Coaches, and encourage local agencies and services that support elders or family caregivers to include REACH in their programming. However, for other areas without well-frequented community gathering places, other ways to publicize, such as radio and television PSAs and social media, may be a better strategy to reach caregivers. (37, 69)	7 - Engage consumers	37, 69
			In addition, patient referral processes and networks must be developed, and initial data on the intervention process and outcomes are gathered. With funding for 3 years from the private Rx Foundation, the process of tailoring the intervention for AI/AN caregivers begun in February 2015. (34)<br><br>The initial process of implementation is slow and dependent on agency leadership support, publicity about the program, and word of mouth. Staff are taking increasing ownership, and several strategies have been implemented to increase participation by staff and by caregivers. For staff, the IHS Clinical Support Center made it possible for training to be accredited for nursing continuing education credits. (2)<br><br>The partnering agencies are supporting REACH through developing needed clinical infrastructure such as billing and workload codes. System infrastructure, especially around payment, is critical in encouraging provider interactions with caregivers. Medicare Cur-rent Procedural Terminology (CPT) codes allow providers to be paid to communicate with caregivers about a beneficiary's care. (70-G8)<br><br>However, services caregivers need may not be reimbursable, such as comprehensive assessment. IHS is addressing these issues by developing coding guidelines to insure that nurses receive workload credit for REACH and can document in the electronic health record. In addition, REACH implementation has been added to staff performance evaluations and instituted as a quality improvement project. (70, 42)	8 - Utilize financial strategies	34, 2, 70, 42
			The initial process of implementation is slow and dependent on agency leadership support, publicity about the program, and word of mouth. Staff are taking increasing ownership, and several strategies have been implemented to increase participation by staff and by caregivers.	9 - Change infrastructure	44, 13, 22, 62

			For staff, the IHS Clinical Support Center made it possible for training to be accredited for nursing continuing education credits. (22)<br><br>Program installation—During program installation, the organization begins to modify and install infrastructure. This infrastructure will likely include funding for staff training and for implementation. (44)<br><br>The 2013 National Plan to Address Alzheimer's Disease, required by the National Alzheimer's Project Act (NAPA), mandated that lessons learned through VA caregiver support strategies, specifying REACH VA, should be shared with state, tribal, and local governments. (44, 13)<br><br>Although this model is still being used, states and Tribes have requested large, in-person trainings followed by on-site certifications. These face-to-face trainings allow staff to interact with the Caregiver Center Coordinator in a more personal way and to interact and share with each other about strategies to deliver the program. Watching certifying role plays provides additional examples of both concerns that may occur and how other staff may handle these concerns. While funding did not originally include face-to-face trainings, they have proved so valuable that the Caregiver Center and the partnering agencies are finding ways to provide them. (44)<br><br>In response to requests for additional information and post-training guidance, the Caregiver Center holds Audio Chats on a monthly basis to discuss implementation of the program. Audio Chats participants have identified the need for a refresher course to help participants gain a better understanding of how to get into the community and suggested implementing a volunteer Marketing/Strategy Committee to help with publicizing the program to local caregivers. (62)		
Star-C	McCurry et al. (2015) / USA	Community AAAs and case managers.<br><br>Two Oregon AAAs serving one urban and two rural Oregon counties participated in the study (Multnomah County Aging & Disability Services [ADS]; Rogue Valley Council of Governments Senior & Disability Service [RVCOG]).	After attendance at these workshops, STAR-C consultants completed one pilot case for which the eight in-home sessions were audio-recorded. Recordings were either uploaded to an electronic site that could be immediately accessed by UW trainers, or transferred to CDs and mailed to UW the next day. This allowed consultants to receive corrective feedback and implement changes in a timely fashion. UW trainers reviewed the audio recordings, completed a consultant adherence checklist, and provided individualized feed-back to consultants. This feedback to consultants included comments about any STAR-C material that was omitted or incorrectly presented. Feedback included suggestions to enhance presentation of the clinical material and to help caregivers brainstorm their own problem solutions with consultant guidance. (5, 56, 14)	1 - Use evaluative and iterative strategies	5, 56, 14
			STAR-C is one of a series of theoretically grounded treatment protocols for training caregivers to improve care and reduce behavioral distress for persons with dementia. Family members are taught a set of core behavioral strategies that include dementia education, effective communication, ABC (activator–behavior–consequence) problem-solving strategies, and implementation of pleasant events to reduce mood and behavioral disturbances in community-dwelling persons with dementia. Caregivers also learn strategies to improve their own emotional well-being and reduce their adverse reactions to challenging behaviors. STAR-C is con-ducted in eight weekly sessions in participants' homes over 2 months, followed by four monthly phone calls. (33)	2- Provide interactive assistance	33
			In response to the need for the AAAs to reduce the costs of service delivery, **UW investigators reconfigured the original STAR-C from eight in-person sessions to four in-person sessions plus two telephone sessions** (STAR-C2). This reconfigured STAR-C2 program—with in-home visits during Weeks 1, 2, 4, and 6, and telephone sessions in Weeks 3 and 5—includes the same content as the original STAR-C program. (63)	3 - Adapt and tailor to context	63
			The Multnomah County ADS identified 11 case managers from five district centers to be trained as STAR-C consultants. Rogue Valley Council of Governments Senior & Disability Services serves a rural two-county area in southern Oregon and identified four existing AAA staff case managers and two private geriatric care managers to be trained as STAR-C consultants. (35)	4 - Develop stakeholder interrelationships	35, 7, 40, 52

			In response to the need for the AAAs to reduce the costs of service delivery, UW investigators reconfigured the original STAR-C from eight in-person sessions to four in-person sessions plus two telephone sessions (STAR-C2). This reconfigured STAR-C2 program—with in-home visits during Weeks 1, 2, 4, and 6, and telephone sessions in Weeks 3 and 5—includes the same content as the original STAR-C program. (7, 40)<br><br>Printed brochures were developed in the first year of the grant. Existing AAA case-managed clients were reviewed to identify potential participants. AAA regional coordinators also distributed brochures at community presentations to medical providers, elder law attorneys, adult day and other dementia care programs, faith-based organizations, senior meal sites, and libraries. Lastly, information about STAR-C was provided to the Alzheimer's Association National Helpline, as well as to local newspapers that featured articles about the program. (52)		
			STAR-C is conducted in eight weekly sessions in participants' homes over 2 months, followed by four monthly phone calls. (19)<br><br>STAR-C is one of a series of theoretically grounded treatment protocols for training caregivers to improve care and reduce behavioral distress for persons with dementia. Family members are taught a set of core behavioral strategies that include dementia education, effective communication, ABC (activator–behavior–consequence) problem-solving strategies, and implementation of pleasant events to reduce mood and behavioral disturbances in community-dwelling persons with dementia. Caregivers also learn strategies to improve their own emotional well-being and reduce their adverse reactions to challenging behaviors. (71, 43)<br><br>Printed brochures were developed in the first year of the grant. (29)<br><br>Potential STAR-C consultants participated in a 1½-day group training conducted by University of Washington (UW) trainers (S.M., R.L., D.L.F.). (71)<br><br>This training included presentation of information about the original RCT results, description of the core STAR-C behavioral components, review of the treatment manual, role playing, and structured practice creating behavioral problem-solving plans using a video series developed by Dr. Linda Teri and colleagues. (43)	5 - Train and educate stakeholders	19, 29, 31, 43, 71
			In 2009, the Oregon Department of Human Services–State Unit on Aging received an AoA Alzheimer's Disease Supportive Services Program (ADSSP) grant (#90AE0334, J.M., PI) to implement STAR-C in the state of Oregon. (34)	8 - Utilize financial strategies	34
			AAA regional coordinators also distributed brochures at community presentations to medical providers, elder law attorneys, adult day and other dementia care programs, faith-based organizations, senior meal sites, and libraries. Lastly, information about STAR-C was provided to the Alzheimer's Association National Helpline, as well as to local newspapers that featured articles about the program. (69)	7 - Engage consumers	69
Medway Carers Course	Milne et al. (2014) / UK	Memory Clinic staff - clinical psychologist and 'speakers'	Although four of the total number of ten sessions are presented by 'invited speakers', the Clinical Psychologist leading the Course was present throughout the programme, introducing speakers and facilitating discussion. (33)	2- Provide interactive assistance	33
			The service was designed and delivered by members of the psychology team for older adults working in a large NHS Health and Social Care Partnership Trust in SE England. (6)	4 - Develop stakeholder interrelationships	6
			The 'Carers Courses' have been running in Medway since the early 2000s. They were originally developed and delivered by very experienced specialist psychologists in response to clinical need, demand from carers, and a growing recognition of the value of interventions that focus on both the person with dementia and their relatives. (29)<br><br>As each Course cohort set their own ground rules, there were slight differences to the pattern of each 2-hour session. However, they usually started with tea/coffee and social mixing (15"),	5 - Train and educate stakeholders	29, 19, 31, 43

			followed by a 45"presentation/discussion, a 15"minute break (tea/coffee, comfort) followed by 45"discussion. Although sessions were formal there was also an informal social component. Carers Courses ran 3 times a year sequentially; they included 10 weekly sessions of two hours each. (19)<br><br>One participant commented that, 'All the information given either in verbal or handout form was very good and could be used in the future as well'. Related comments included: 'The presentation methods...all presenters went slowly and carefully, were ideal for this type of audience','...it was so good to be able to ask questions of the session presenters'and 'group discussion helped understanding of different needs and issues'. (31, 43)		
			Although four of the total number of ten sessions are presented by 'invited speakers', the Clinical Psychologist leading the Course was present throughout the programme, introducing speakers and facilitating discussion. (59)<br><br>In terms of services for people with early stage dementia, the Medway Memory Service was established in 2003. It was developed as a part of a broader shift from hospital-based to community-based services incorporating hospital staff under the umbrella of secondary Mental Healthcare Services. Most referrals to the Memory Service are from a GP. Following a thorough multi-disciplinary assessment of the referred patient's cognitive –and related – functioning the Memory Service staff hold a meeting with the person and their family (if they so wish) to discuss the outcomes of their investigation(s). (21)	6 - Support clinicians	59, 21
CARES Dementia Basics Program	Pleasant et al. (2016) / USA	**Oregon Care Partners** was established to implement free training for all caregivers in the state, reflecting the Oregon Alzheimer's Disease State Plan recommendations	Oregon Care Partners was established to implement free training for all caregivers in the state, reflecting the Oregon Alzheimer's Disease State Plan recommendations (SPADO Force,2012). Equal opportunity training among caregivers was funded to promote better quality care for all Oregonians with Alzheimer's disease regardless of setting. Participants were recruited through existing consumers of CARES® in Oregon and through networking with the Oregon Health Care Association.	4 - Develop stakeholder interrelationships	52
			The CARES®Dementia Basics program offers a systematic approach to assess multiple caregiving scenarios. CARES® was created by Health Care Interactive and consists of four modules: (1)Meet Clara Jones, an introduction to person-centered care; (2) introduction to dementia; (3),understanding behavior as communication; and(4) the CARES® approach to Connect, Assess, Respond, Evaluate, and Share with other team members when providing care. (43)<br><br>The present evaluation was one of the main educational tools, the CARES® Dementia Basics Program, offered to train Oregon caregivers. (31)	5 - Train and educate stakeholders	43, 31
			To progress through the program, a minimum of 40 minutes must be been spent in each module and all screens viewed. A notable strength is the systematic framework of CARES® that encompasses the lessons of person-centered care, changes with dementia, and behavior as communication. Caregivers can refer to the CARES® model when in a new or particularly challenging care scenario. (50)	7 - Engage consumers	50
			The CARES®Dementia Basics Program has the support of the Alzheimer's Association, and users have the option of completing the Alzheimer's Association **essentiALZ certification exam offered after completing the CARES® program**. (22)<br><br>The online training is both time- and cost-effective, and in past evaluations direct care workers largely report enhanced feelings of self-efficacy and demonstrated knowledge gains. (13)	9 - Change infrastructure	13, 22
Taking Care of YOU: Self-Care for Family CaregiversToolkit	Smith and Graves (2020) / USA	FCA - community-based nonprofit organization that combines research, policy, and practice to advance development of high-quality, cost-effective programs and	Sessions were held in the primary care office and guided by "Taking Care of YOU:Self-Care for Family Caregivers Toolkit"(FCA, 2006). The FCA recommends seven domains for caregiver assessment which are caregiver background, caregiver perception of health, caregiver values, the caregiver health and well-being, consequences of caregiving, care provision requirements, and caregiver resources to support serv-ices (FCA, 2006) (4)	1 - Use evaluative and iterative strategies	4
			The investigator waited behind to meet with any participant individually to conduct a postintervention CSAQ and to answer any questions or concerns of the participant. (33)	2- Provide interactive assistance	33

		policies for caregivers in the United States	The FCA organized a national conference that gathered leaders in health care and long-term care to advance policy and practice on behalf of informal caregivers(FCA, 2006). Through a consensus process the "Caregivers Count Too! A Toolkit to Help Practitioners Assess the Need of Family Caregivers" was developed. (64)	4 - Develop stakeholder interrelationships	64
			The FCA organized a national conference that gathered leaders in health care and long-term care to advance policy and practice on behalf of informal caregivers(FCA, 2006). Through a consensus process the "Caregivers Count Too! A Toolkit to Help Practitioners Assess the Need of Family Caregivers" was developed. (29)<br><br>During the participants office and follow-up visits, the investigator engaged caregivers in two 1-hour education sessions over an 8-week period. (19)	5 - Train and educate stakeholders	19, 29
Exercise (n=3)					
TAiChI for people with demenTia (TACIT trial)	Barrado-Martin et al. (2019) / UK	Tai Chi instructor and research group	Venues were chosen after checking their suitability against various criteria: size (able to accommodate between 14 and 20 people), maintenance conditions, accessibility by car and/or public transport, time slots availability, flexible booking, availability of on-site kitchen facilities, and general accessibility within the venue (i.e., lifts and toilets). (4)<br><br>we learnt that for the future trial phase of this study, classes lead by the same instructor would need to start at least 2 weeks apart and home visits would only take place after the two first weeks of class practice. This way we could ensure enough time for the instructors to conduct these home visits without fail. In addition, information given to the participants has been revised to make clear that there is to be no home practice until the home visit is made. (56)	1 - Use evaluative and iterative strategies	4, 56
			Classes were led by a professionally trained Tai Chi instructor with experience of working with older participants living with and without dementia. (33)<br><br>He regularly provided positive feedback during the classes to encourage participants' engagement in the activity and reassured the participants when they verbalized difficulties while doing Tai Chi at home or during classes. Sometimes, this positive feedback was given when some participants were struggling to perform the movements or doing them incorrectly. This approach was chosen by the instructor for this study to facilitate their engagement. (33)	2- Provide interactive assistance	33
			Classes were delivered during working days around midday on a weekly basis, following advice from the public and patient involvement advisory group that was involved in the TACIT trial's design (63)<br><br>The instructor adapted the intervention to participants' needs and responded to their requests (i.e., introducing breathing while practicing one movement, as requested by a carer during the class)to make the intervention accessible for both people living with dementia and their carers. (51)<br><br>Venues were chosen after checking their suitability against various criteria: size (able to accommodate between 14 and 20 people), maintenance conditions, accessibility by car and/or public transport, time slots availability, flexible booking, availability of on-site kitchen facilities, and general accessibility within the venue (i.e., lifts and toilets). (51)	3 - Adapt and tailor to context	63, 51
			Classes were led by a professionally trained Tai Chi instructor with experience of working with older participants living with and without dementia. (57)	4 - Develop stakeholder interrelationships	57
			A booklet was provided to act as a prompt for participants' home practice, reminding participants how to perform the movements. This booklet contained several pictures of each pattern, supported by explanatory text below each picture. (31)<br><br>Dyads were supposed to receive a home visit from the instructor during the second week of their participation in the study to ensure a safe practice at home and complete an action and a coping plan. (16)	5 - Train and educate stakeholders	31, 16, 29, 19

			The action plan was introduced to identify a suitable time for home practice and the coping plan to develop strategies to over-come possible barriers to home practice (Chase, 2015). (29)<br><br>The classes were to run over 4 weeks to allow the study of the acceptability of the classes, the home visit conducted by the instructor, and the home-based practice and the data collection methods used during dyad's involvement in the study in the short-term. (19)		
			The action plan is the document where both members of the dyad specify which days of the week, at what times (morning/afternoon/evening), for how long, where specifically, and with whom will they practice Tai Chi. The coping plan is the document where both members of the dyad identify the anticipated barriers for practicing Tai Chi at home. (12)	9 - Change infrastructure	12
TAiChI for people with demenTia (TACIT trial)	Barrado-Martin et al. (2020) / UK	Tai Chi instructor and research group	The classes were led by two fully trained and experienced Tai Chi instructors, who were in charge of making a home visit with each dyad after their second week attending classes. (33)	2- Provide interactive assistance	33
			The classes were led by two fully trained and experienced Tai Chi instructors, who were in charge of making a home visit with each dyad after their second week attending classes. (57)	4 - Develop stakeholder interrelationships	57
			Participants in the intervention arm were invited to join weekly Tai Chi classes for 20 weeks, as well as practising at home for 20 minutes a day, as per protocol (43)<br><br>The classes were led by two fully trained and experienced Tai Chi instructors, who were in charge of making a home visit with each dyad after their second week attending classes. (55)<br><br>Dyads completed an action and coping plan with the instructor, who left a copy of these at participants' home for their reference. The action plan reflected what days of the week, when, where and for how long would participants be practising at home; whilst the coping plan was designed to anticipate barriers to their home practice as well as ways of overcoming such challenges (43)<br><br>At the end of the group sessions, participants were provided with a total of three colourful ,professionally produced, illustrated booklets as well as nine additional sheets designed to point participants towards the exercises to be practised each week. Participants were due to practise at home according to the week number, with some additional sheets covering several weeks. (43, 31)	5 - Train and educate stakeholders	55, 31, 43
			Similarly, during these initial home visits, instructors provided participants with an alarm clock to remind their practice at their agreed time of practice and 18 exercise logs to report their weekly home practice (11, 12)	9 - Change infrastructure	11, 12
dyadic intervention study with exercise and support	Prick et al. (2014) / The Netherlands	coaches were five MSc students of the Department of Clinical Psychology, VU University, who followed a special training program on geropsychology and extensive training to provide the present intervention	After a pilot study, using a translated version of the Teri intervention, we made changes to the intervention components and duration to adapt the intervention to the Dutch care situation. Because the evaluation of the pilot study showed that a proper execution of the intervention took more than 1 hour, we decided to concentrate on physical exercise, education and pleasant activities training and to drop the time-consuming ABC training. (56)	1 - Use evaluative and iterative strategies	56
			To improve the attractiveness of the exercises for people with dementia, we used additional materials like a ball, weights and elastic bands, which we integrated with the original exercises. Further-more, in contrast to the intervention of Teri, we taught caregivers not only to plan pleasant activities for people with dementia but also for caregivers themselves to reduce psychological distress. To the user manual, we added a list of pleasant activities ideas especially for caregivers to stimulate planning pleasant activities for themselves. (63, 51)<br><br>In addition, we used elements of a Dutch exercise program for people with dementia and their caregivers designed by Dutch physiotherapists. (63, 51)	3 - Adapt and tailor to context	63, 51
			Therefore, we changed our recruitment strategy into a personal approach to the dyads by giving presentations at local Alzheimer cafes (public meetings for people with dementia, their caregivers and others). We started with visiting Alzheimer cafes in the neighborhood of Amsterdam. Later, we expanded our visits to Alzheimer cafes throughout the Netherlands.	4 - Develop stakeholder interrelationships	52

			Personally contacting potential participants or case managers was more successful. When contacting case managers, we asked them to look out for potential participants in their own caseload. Case managers contacted clients that seemed to be potential participants for this study and, if they were interested, their permission was asked as to whether the researchers were allowed to contact them. (52)		
			In line with the intervention of Teri and colleagues [14], four types of exercises were instructed and practiced: flexibility, strengthening, balance and endurance exercises. The exercises were introduced gradually, session-by-session by an individual coach. (19)<br><br>Alongside the instruction visits, all dyads received a user manual with pictures of the exercises and easy-to-read instructions (31)	5 - Train and educate stakeholders	**19**, 31
			Therefore, we changed our recruitment strategy into a personal approach to the dyads by giving presentations at local Alzheimer cafes (public meetings for people with dementia, their caregivers and others). We started with visiting Alzheimer cafes in the neighborhood of Amsterdam. Later, we expanded our visits to Alzheimer cafes throughout the Netherlands. Personally contacting potential participants or case managers was more successful. When contacting case managers, we asked them to look out for potential participants in their own caseload. Case managers contacted clients that seemed to be potential participants for this study and, if they were interested, their permission was asked as to whether the researchers were allowed to contact them. (30)	6 - Support clinicians	30
			Recruitment was time consuming and complicated in this geriatric population. We started the recruitment with advertisements in national and local newspapers and on geriatric websites (69)	7 - Engage consumers	69
			The number of home visits was decreased to eight instead of the original 12 (in order to comply with Dutch health insurance regulations about the number of home visits typically reimbursed), and the frequency of home visits in the first month was decreased to one instead of two home visits per week. (49)	8 - Utilize financial strategies	49
			A personal coach visited the dyads in their own homes for eight 1-hour home visits over 3 months. In the first month the dyads were visited weekly, followed by bi-weekly home visits over the next 8 weeks.(13)	9 - Change infrastructure	13
Care coordination and case management (n=6)					
Cleveland Alzheimer's managed Care Demonstration	Bass et al. (2003) / USA	Care consultation is delivered by one of three Association staff members, two of whom are master's prepared licensed social workers	Regularly scheduled follow-ups monitor progress and add new tasks to the care plan as needed. Follow-ups are initially done biweekly, decreasing to 1-month and 3-month intervals unless needs dictate more frequent contacts. In difficult periods, daily contact with care consultants may be necessary. Alternatively, if care consultants, patients, and caregivers agree and there are no problems that have not been addressed or discussed, trained volunteers make follow-up contacts, with care consultants on call. (27)<br><br>Care consultants follow a standardized protocol for service delivery that includes conducting a structured initial assessment, identifying problems or challenges, and developing strategies for using personal, family, and community resources. (4)	1 - Use evaluative and iterative strategies	27, 4
			Care consultants also provide information about available community services, facilitate decisions about how to best utilize and apply for these services, and may contact service agencies on behalf of patients and caregivers. (33)<br><br>Care consultants work with families in a collegial fashion to help identify personal strengths, as well as resources within the family system, health plan, and community. (33)	2- Provide interactive assistance	33
			Care consultants work with families in a collegial fashion to help identify personal strengths, as well as resources within the family system, health plan, and community. (63)	3 - Adapt and tailor to context	63
			The partnership tested in this investigation adds care consultation from the Cleveland Area Alzheimer's Association to usual managed care services offered to members of Kaiser Permanente (52)	4 - Develop stakeholder interrelationships	52

			Regularly scheduled follow-ups monitor progress and add new tasks to the care plan as needed. Follow-ups are initially done biweekly, decreasing to 1-month and 3-month intervals unless needs dictate more frequent contacts. In difficult periods, daily contact with care consultants may be necessary. Alternatively, if care consultants, patients, and caregivers agree and there are no problems that have not been addressed or discussed, trained volunteers make follow-up contacts, with care consultants on call. (55)<br><br>Care consultants work with families in a collegial fashion to help identify personal strengths, as well as resources within the family system, health plan, and community. (55)<br><br>The goal is to provide tools to enhance patients' and caregivers' competence and self-efficacy (71)<br><br>The care plan outlines specific tasks to be completed; assigns patients, family members, or Association staff/volunteers to work on these tasks; and gives a time frame for task completion and reassessment. Tasks often include using other Association services, such as education and training programs, support groups, a respite reimbursement program, and a nationwide program to return wanderers safely home. (19, 15, 55.43)	5 - Train and educate stakeholders	55, 71, 19, 15, 43
			Care consultation is delivered by one of three Association staff members, two of whom are master's prepared licensed social workers (59)<br><br>This demonstration examines outcomes of a partnership between a managed care system and a community agency that provides information and support services. The intervention added Alzheimer's Association care consultation service as a supplement to managed care services. (21)<br><br>The partnership tested in this investigation adds care consultation from the Cleveland Area Alzheimer's Association to usual managed care services offered to members of Kaiser Permanente (30)	6 - Support clinicians	59, 21, 30
			Care consultants collaborate with patients and family caregivers to create an individualized plan of care. (39)	7 - Engage consumers	39
			The care plan outlines specific tasks to be completed; assigns patients, family members, or Association staff/volunteers to work on these tasks; and gives a time frame for task completion and reassessment. Tasks often include using other Association services, such as education and training programs, support groups, a respite reimbursement program, and a nationwide program to return wanderers safely home. (49)<br><br>All Association services are free-of-charge. (66, 34)<br><br>The partnership tested in this investigation adds care consultation from the Cleveland Area Alzheimer's Association to usual managed care services offered to members of Kaiser Permanente. **(embedded into existing services)** (49)	8 - Utilize financial strategies	49, 66, 34
Partners in Dementia Care	Bass et al. (2014) / USA	Two half-time care coordinators, with part-time administrative assistant support, delivered PDC at each intervention site.	VINs provide a unifying administrative structure for all VA services within a given geographic region. (8)	2- Provide interactive assistance	8
			Since completion of PDC, more than two dozen diverse organizations have been licensed to deliver BRI Care Consultation, including healthcare organizations, Alzheimer's Association chapters, family counseling agencies and Area Agencies on Aging. (36)<br><br>A key feature of PDC is its basis in a formal partnership between a healthcare organization (for example, VA medical centers) and a community service organization (for example, Alzheimer's Association chapters). (52)<br><br>There were five study sites: Boston, MA; Houston, TX; Providence, RI; Oklahoma City, OK; and Beaumont, TX, with all sites located in one of two selected Veterans Integrated Service Networks (VISNs). (72)	4 - Develop stakeholder interrelationships	52, 6, 36, 72, 24

			Study sites were matched by VIS to assure uniformity in this overarching ad- ministrative structure. One of the two selected VISNs (that is, VISN 16, which includes Houston, Oklahoma City and Beaumont) was chosen because it was the location of the study's VA principal investigator. The other selected VISN (that is, VISN 1, which includes Boston and Providence) had a similar array of VA services as VISN 16. (52, 6)<br><br>Within each of the two selected VISNs, VA study sites were matched to be similar in: size, services offered (both inpatient and outpatient), academic affiliations, research missions and medical-residency training programs. Alzheimer's Associations chapters within selected VISNs were similar in size, with comparable core programs and services. (24, 52)<br><br>One care coordinator worked in the local VA medical center (healthcare organization) and the other worked in the partnering Alzheimer's Association chapter (community service organization). (52)		
			One care coordinator worked in the local VA medical center (healthcare organization) and the other worked in the partnering Alzheimer's Association chapter (community service organization). (73)<br><br>Training for care coordinators consisted of a 1.5-day initial session on the PC philosophy, service-delivery protocol and the CIS that guides service delivery. Additionally, one- to two-hour biweekly refresher trainings were completed throughout the study period. (19, 71)	5 - Train and educate stakeholders	73, 19, 71
			Two half-time care coordinators, with part-time administrative assistant support, delivered PDC at each intervention site. (59)<br><br>Although from different organizations, the two care coordinators worked as a team, with one shared electronic Care Coordination Information System (CIS) and regularly scheduled planning and case-conference meetings. (30)<br><br>The care coordinator from the VA medical centers had primary responsibility for assisting veterans with medical- related concerns (for example, medications, accessing medical services, disease management) while the care coordinator from the Alzheimer's Association chapter had primary responsibility for assisting caregivers with nonmedical concerns (for example, care-related strain, accessing family support and information services). The VA care coordinator also focused on helping families access VA services and benefits, whereas the Alzheimer's Association care coordinator focused on helping families use community services, including those offered by the Alzheimer's Association. This division of labor between care coordinators capitalized on the complementary strengths of each partner organization and represented a bridge between health care and community services. (21)	6 - Support clinicians	59, 30, 21
			The care coordinator from the VA medical centers had primary responsibility for assisting veterans with medical- related concerns (for example, medications, accessing medical services, disease management) while the care coordinator from the Alzheimer's Association chapter had primary responsibility for assisting caregivers with nonmedical concerns (for example, care-related strain, accessing family support and information services). The VA care coordinator also focused on helping families access VA services and benefits, whereas the Alzheimer's Association care coordinator focused on helping families use community services, including those offered by the Alzheimer's Association. This division of labor between care coordinators capitalized on the complementary strengths of each partner organization and represented a bridge between health care and community services. (50)<br><br>PC is a coaching model driven by consumer choice, with care coordinators helping find solutions to concerns that are the priorities of veterans and caregivers. PDC followed a set, standardized protocol that required a minimum of at least one contact between care coordinators and consumers per month; more-frequent contacts occurred as needed. The	7 - Engage consumers	50, 41

			protocol required care coordinators to discuss with veterans and/or caregivers a broad range of medical and nonmedical concerns, although the specific content was customized to consumers' preferences and needs. (41)		
			All expenses to deliver PDC (that is, salaries, benefits, equipment, supplies, training, software, licensing, supervision, administrative overhead) can be recovered by charging a fee of $60 to $80 per month per family. (66)	8 - Utilize financial strategies	66
			The Benjamin Rose Institute on Aging holds the copyright to BRI Care Consultation and currently licenses and trains organizations to deliver the program. (22)<br><br>Although from different organizations, the two care coordinators worked as a team, with one shared electronic Care Coordination Information System (CIS) and regularly scheduled planning and case-conference meetings. (12)<br><br>PDC is a low-cost service delivered by telephone, mail and e-mail, with in-person contacts rarely needed. (13)	9 - Change infrastructure	22, 12, 13
Aged Care Assessment Teams	Bruce and Patterson (2000) / Australia	GP referral to ACAT; ACAT delivered by external (community level) service provider financed by government	-	-	-
COEP	Connell and Kole (1999) / USA	COEP staff; dementia service providers (community care)	After the results of the community assessment were shared with the advisory board, priorities for the intervention were identified, including providing dementia training for local information and referral staff and increasing knowledge and awareness of dementia among the public, service providers, and health professionals. Work groups, including publicity and promotion, local arrangements, and curriculum development, were established to facilitate implementation of the intervention. (56)<br><br>In collaboration with the local advisory boards, the COEP team conducted a dementia-specific community assessment to identify community resources and strengths, gaps in service availability, and strategies to increase coordination and communication among service providers. (4)<br><br>These two team members were responsible for conducting a comprehensive community assessment at each site, establishing local advisory boards, fostering networking and communication, designing program curriculum, and implementing the onsite interventions. (4)	1 - Use evaluative and iterative strategies	4, 56
			Program planning and implementation was greatly facilitated by the COEP's primary contact person and local leader. A local hospital provided funding to mail the conference brochures and a social service agency coordinated the registration process. Most of the COEP activities took place in a centrally located church. The major obstacle to gaining adequate representation and participation from this site was the geographic distance between the population centers in the three-county region. (52)<br><br>After the results of the community assessment were shared with the advisory board, priorities for the intervention were identified, including providing dementia training for local information and referral staff and increasing knowledge and awareness of dementia among the public, service providers, and health professionals. Work groups, including publicity and promotion, local arrangements, and curriculum development, were established to facilitate implementation of the intervention. (17)<br><br>To enhance a sense of community ownership of the project, the first advisory board meeting was devoted to gaining members' input about local goals and priorities and how to accomplish them via the COEP. (48)<br><br>Other members of the advisory board included staff of the local hospital, home health agency,	4 - Develop stakeholder interrelationships	47, 52, 17, 24, 64, 6, 38, 40, 48

			information and referral agency, county hospital, newspaper, and community mental health agency. (40, 52, 6)<br><br>The COEP team consulted with members of an advisory board established to guide this geriatric assessment team and decided it was well suited to represent the COEP as well. A local leader and strong advocate for increasing the availability of dementia services at the state level served as the primary COEP contact person in this site. (38)<br><br>The COEP was established as a community-based initiative, built on a partnership between the project team and the community. Such partnerships can facilitate the definition of locally relevant public health issues, the development of culturally appropriate interventions, a sense of project ownership, and the process of creating sustainable community change.(47, 52)<br><br>In collaboration with the local advisory boards, the COEP team conducted a dementia-specific community assessment to identify community resources and strengths, gaps in service availability, and strategies to increase coordination and communication among service providers. (17)<br><br>The advisory boards consisted of health care professionals, dementia service providers, and representatives of community and voluntary agencies that address the needs of people with dementia. The boards served as community-based coalitions, defined as “a group of individuals representing diverse organizations, factions, or constituencies who agree to work together in order to achieve a common goal”. (6, 64, 52)<br><br>These two team members were responsible for conducting a comprehensive community assessment at each site, establishing local advisory boards, fostering networking and communication, designing program curriculum, and implementing the onsite interventions. (17)<br><br>The COEP was conducted in collaboration with the Michigan Alzheimer’s Disease Research Center at the University of Michigan in Ann Arbor. (24)		
			Work groups, including publicity and promotion, local arrangements, and curriculum development, were established to facilitate implementation of the intervention. (29)<br><br>To enhance a sense of community ownership of the project, the first advisory board meeting was devoted to gaining members’ input about local goals and priorities and how to accomplish them via the COEP. (15)	5 - Train and educate stakeholders	29, 15
			the COEP team developed a telephone interview protocol to be used by advisory board members to assess the educational needs of local physicians. (30)<br><br>The COEP project team included two full-time health educators trained at the master’s level in public health with expertise in community-based approaches to outreach education. (59)	6 - Support clinicians	30, 59
			two strategies to increase public awareness of dementia were suggested: (a) a media advocacy session targeted to members of the board of the local chapter of the Alzheimer’s Association and (b) a speakers’ bureau training for volunteers willing to disseminate information about dementia to interested groups. (37, 69)	7 - Engage consumers	37, 69
			Primary funding for the COEP was provided by a grant from the National Institute on Aging designed to support community out-reach education programs as part of existing Alzheimer’s Disease Research Centers. (…)<br>Supplemental funding for the COEP was provided by the Health Resources and Services Administration via a grant to the Michigan Department of Mental Health. (1, 34)	8 - Utilize financial strategies	1, 34
			As a first step, local advisory boards were established at each of the six sites involved in the project. (13)	9 - Change infrastructure	13

Healthcare Professional Support	Laparidou et al. (2018) / UK	Staff from the local Dementia and Specialist Older Adult Mental Health Service	In addition, these staff had everyday contact with people with dementia and their informal caregivers, as they provided both specialist short-term in-patient assessment and treatment, as well as individualised care and management plans for patients with dementia and their caregivers. (33)	2- Provide interactive assistance	33
			Informal caregivers of people with dementia were recruited by various methods, including through carer support groups (from different geographical areas of the rural county of Lincolnshire), the University of Lincoln, and a Patient and Public Involvement (PPI)Group. (24)<br><br>HCPs were recruited from two Lincolnshire general practices (in different geographical areas) and the main mental health provider of care for patients and caregivers of people with dementia within Lincolnshire. (52)<br><br>Although our focus was on HCPs working in primary care, we included staff from the local Dementia and Specialist Older Adult Mental Health Service, as they received referrals from community health professionals regarding patients with dementia. (36, 64, 52)	4 - Develop stakeholder interrelationships	24, 52, 36, 64
			Although our focus was on HCPs working in primary care, we included staff from the local Dementia and Specialist Older Adult Mental Health Service, as they received referrals from community health professionals regarding patients with dementia. (59, 21)	6 - Support clinicians	59, 21
SUSTAIN program	Mavandadi et al. (2017) / USA	Health technicians	During the first contact, the BHP provided a general over-view of the format, content, and goals of the TEP program. The BHP also completed a brief intake assessment that included caregiver- and care recipient– related scales. Based on the caregiver's responses to this assessment, the BHP recommended TEP modules to the caregiver and then collaborated with the caregiver in determining the initial list of TEP modules to be covered throughout the course of the program. (4)	1 - Use evaluative and iterative strategies	4
			In SUSTAIN, TEP was modified for use with individual caregivers and was formatted so that care- givers could select from a menu of up to seven modules covering various content areas evaluated during the course of the care management assessments (communication skills, behavioral management techniques, stress management and coping skills, long-term planning, etc.).	2- Provide interactive assistance	33
			By scheduling calls based on the caregivers' availability and taking a tailored approach to the delivery of care, we were able to improve access to and engagement rates among caregivers who may otherwise not have participated. For example, tailoring all aspects of program delivery to individual needs and preferences may have led, in part, to the high engagement rates among caregivers who reported providing more hours of care and greater burden. Brief, telephone- based caregiver interventions also are cost-efficient and can improve outreach to those receiving primary care from smaller clinics or rural settings. (51, 63)<br><br>Offering caregivers a program that is telephone-based; individually tailored to take variability in care recipient/caregiver needs, preferences, and comorbidity into account; manualized (which enables immediate and convenient access to program material at the caregivers' convenience); and brief (i.e., 3 months) in duration may help overcome some of the logistical issues that serve as barriers to engagement in caregiver-based interventions and minimize caregiver strain. (51)	3 - Adapt and tailor to context	51, 63
			All caregivers were mailed contact information for the Alzheimer's Association of America Greater Pennsylvania chapter. (52)	4 - Develop stakeholder interrelationships	52
			Individuals assigned to ECS received all of the services described for the CAR group as well as service connection, psychoeducation, and support delivered by BHPs. When appropriate, BHPs provided to caregivers, either via mail or phone, information regarding service referrals and community resources and helped with coordination of services for needs that were identified during the needs assessment. BHPs also directly contacted and communicated with the prescribing provider when indicated. (29, 43)<br><br>The central component of the ECS arm, however, was the Telehealth Education Program	5 - Train and educate stakeholders	29, 43, 31, 55

			(TEP). TEP is a manualized, telephone-delivered program developed and validated with caregivers of veterans with moderate to severe dementia. In order to ensure fidelity to the model and facilitate training, supervision, and uniform delivery of care, the program material includes a care manager leader manual, which provides detailed scripts for the psychoeducation and skills training components of each module, and a corresponding caregiver workbook (29, 43, 31)<br><br>ECS intervention arm services were delivered for approximately 3 months from the date of the baseline assessment and caregivers typically engaged in two to three contacts per month. (55)		
			The SUSTAIN program was implemented by the state of Pennsylvania in 2008 to provide free, evidence-based, mental health and dementia care management services by telephone to low-income older adults (and, where appropriate, their informal caregivers) en- rolled in a statewide pharmaceutical assistance program and their primary care providers. To be eligible for SUSTAIN, enrollees must be newly prescribed a psychotropic medication. Notably, the program uses case-finding via pharmacy records, which enables enrollment of older adults and their caregivers from a geographically diverse community sample (34)	8 - Utilize financial strategies	34
			The TEP program has been delivered over the telephone in a group format in other settings. (13)	9 - Change infrastructure	13
Occupational Therapy (n=3)					
Community Occupational Therapy in Dementia (COTiD) programme	Burgess et al. (2020) / UK	occupational therapist	Each person identifies meaningful activities that are important to them through a narrative interview. (4)	1 - Use evaluative and iterative strategies	4
			The sessions are delivered either in the person with dementia's home or in the local community–depending on the goals that have been set. (51)	3 - Adapt and tailor to context	51
			COTiD-UK is a manualised intervention focused on enabling meaningful occupation. It consists of up to 10 h of community occupational therapy delivered over a period of approximately 10 weeks to the person with dementia and their family carer together. (19)<br><br>The COTiD-UK focus on meaningful occupation (activity) is core to occupational therapy. It involves facilitating a range of activities that participants can use inside and outside their home. (43)	5 - Train and educate stakeholders	19, 43
			The occupational therapist works in partnership with the pair, with each having their own need for meaningful activities; hence, COTiD-UK requires a high level of skill to deliver. (50)	7 - Engage consumers	50
			The sessions are delivered either in the person with dementia's home or in the local community–depending on the goals that have been set. (13)	9 - Change infrastructure	13
VALID - Occupational Therapy	Field et al. (2019) / UK	Occupational therapist	Assessment included both interviews with participants and structured observation of activity. (4, 18)	1 - Use evaluative and iterative strategies	4, 18
			This was followed by personalised goal-setting, based upon assessment findings, then sup- ported practice and strategy use to achieve goals. (63, 51)	3 - Adapt and tailor to context	63, 51
			Information about the intervention and research participation was provided to potential participants by clinicians working in NHS memory services or community mental health services. (52)	4 - Develop stakeholder interrelationships	52
			In the UK, intervention involved approximately 10 tailored sessions with an occupational therapist in people's homes or local communities. (19)	5 - Train and educate stakeholders	19
			These included nurses, doctors, clinical psychologists and occupational therapists. (21)	6 - Support clinicians	21
Environmental skill-building program (ESP)	Gitlin et al. (2010) / USA	Fox Rehabilitation, a private practice that provides physical therapy, occupational therapy, and speech-language pathology services to older adults in home, assisted living, or adult medical day centers	ESP involves up to eight home sessions over four months by occupational therapists who assess specific needs concerns and challenges of caregivers (4, 18)<br><br>"This translational phase was diagnostic and necessary prior to full implementation of ESP in a multistate homecare agency (Woolf, 2008). From this phase, we were able to determine training adequacy and identify areas for which additional therapist support (problem solving) and coaching (explaining ESP to reluctant caregivers) were necessary. (56)	1 - Use evaluative and iterative strategies	4, 18, 56
			the physical and social environment, caregiver management approaches, and dementia patient functionality. based on assessments and prioritization of caregiver identified concerns,	3 - Adapt and tailor to context	63

		in five mid-Atlantic states.	therapists tailor disease education instruction in problem solving and implementation of strategies (63)		
			We learned that translation was labor intensive, requiring a series of integrated activities involving key stakeholders from research agency sites. Consistent with other translational reports, open negotiations and shared understandings were essential to success. (17, 6) Also, as fidelity and evaluating caregiver outcomes must be embedded within agency practices for sustainability, developing and implementing all procedures required a collaborative approach. Thus, forming and sustaining a partnership based on a shared vision, mutual respect and trust was essential.(6, 25)	4 - Develop stakeholder interrelationships	17, 6, 25
			Specifically, research partners needed to obtain a fine-grained understanding of practice site characteristics and reimbursement requirements, whereas agency partners needed to understand the mutable and immutable components of ESP and its training requirements. (20, 73)<br><br>Therapists indicated training components supported learning ESP (100%), and face-to-face training strategies (role-play, videos, case stories, and PowerPoint presentations) helpful (95%) (43, 71)	5 - Train and educate stakeholders	20, 73, 43, 71
			ESP was integrated within a patient therapeutic contact and billed as a component of “self-care/home management training” that included caregiver training. (49, 70)	8 - Utilize financial strategies	49, 70
Multi-component (n=18)					
REACH	Burgio et al. (2001) / USA	interventionists	To guarantee a consistent level of accuracy in treatment delivery, all REACH interventions were guided by detailed treatment manuals. Each of the manuals was examined by the coordinating center for consistency across sites in format and level of detail. These extensive manuals described all aspects of treatment delivery and assessment. (27)<br><br>Thus, all REACH sites conducted periodic assessments of the interventionists’ performance. This was accomplished largely by audiotaping interactions with caregivers, either at every therapeutic session or on a random basis. These audiotaped interactions were coded by an individual at the site who was knowledgeable about the intervention to ensure accurate and consistent adherence with protocol. Coding of each interventionist’s performance was guided by a treatment delivery and accuracy checklist on which the coder rated the interventionist’s performance according to previously identified key treatment components. The completed checklist was then used to provide feedback to the interventionist on accuracy of performance. Thus, both the therapist and the supervisory checklists were used for delivery induction and assessment. (27)<br><br>This was done particularly at sites where multiple and, at times, rotating interventionists (e.g., psychological interns) were used, to ensure that treatments were delivered in a consistent manner by all involved throughout the life of the project. (27)<br><br>In addition, some REACH sites incorporated the practice of weekly group supervision sessions to allow very careful and consistent feedback and monitoring of their interventionists. (5)	1 - Use evaluative and iterative strategies	27, 5
			To increase the consistent application of the MSC protocol across sites, the project managers from each site participated in regular phone conferences where problems in delivery were discussed. (53)	2- Provide interactive assistance	53
			Language preference and translation issues go hand in hand: interventions cannot be delivered in the caregivers’ language of choice unless suitable translations of the material are available, along with bilingual (and preferably bicultural) staff to offer the programs. Because two of the six sites worked with Spanish-speaking caregivers (in Miami, Cuban Americans were the majority of Hispanics seen; in Palo Alto, Mexican Americans were the majority), efforts were made to coordinate translation processes across these sites. (63, 51)<br><br>This information was immediately entered into the REACH core database by data entry staff. A record of contacts allowed investigators to analyze outcomes based on type, number, and	3 - Adapt and tailor to context	63, 51, 68

			duration of contacts. It also allowed the supervisor to provide feedback to the interventionists about the degree to which the intervention was delivered to and received by the client. (68)		
			Training was followed by an evaluation procedure, supervised by the coordinating center, that certified the individual to serve as an interventionist.(57)<br><br>A professional translation company had to be used first for the forward translations, to get them into "generic Spanish" that would provide a culturally appropriate starting point. Then panels of bilingual and bicultural Hispanics representing different Hispanic subgroups were convened to do the back translations. (52)	4 - Develop stakeholder interrelationships	57, 52
			To guarantee a consistent level of accuracy in treatment delivery, all REACH interventions were guided by detailed treatment manuals. Each of the manuals was examined by the coordinating center for consistency across sites in format and level of detail. These extensive manuals described all aspects of treatment delivery and assessment. (29)<br><br>Manuals were used as training tools and to maintain accurate delivery over time. Interventionists were given a treatment manual that provided a detailed account of each treatment component and a step-by-step timeline to ensure timely delivery of all intervention activities. In many of the sites, therapists also carried into each session treatment delivery checklists to remind them of the necessary treatment components. Manuals were also a convenient and accurate source of guidance regarding special circumstances that might occur during intervention. (31, 71, 43)<br><br>A formal procedure for training interventionists was developed by the investigators at each site. Training consisted of independent readings, didactic instruction, hands-on demonstration, and performance feedback to enable interventionists to implement treatment with AD caregivers. (43, 31, 71)<br><br>A professional translation company had to be used first for the forward translations, to get them into "generic Spanish" that would provide a culturally appropriate starting point. Then panels of bilingual and bicultural Hispanics representing different Hispanic subgroups were convened to do the back translations. (29)<br><br>Overall, the process took about a year to accomplish. Second, once these translations had been accomplished effectively, interventionists who were bilingual (and in most instances, bicultural) had to be selected and trained to use the manuals and the treatment delivery assessment forms. (71)	5 - Train and educate stakeholders	29, 31, 71, 43
			In 1995, NIH funded six intervention sites and a coordinating center to develop interventions for family caregivers of individuals at the moderate level of impairment (see Appendix). Each site investigated a different intervention, although there were commonalities in the treatment components used across sites. (34, 1)	8 - Utilize financial strategies	34, 1
			Interventionists were provided with certificates acknowledging that they had acquired the skills necessary for delivering the intervention. (22)<br><br>At all REACH sites, a standard form was used to systematically document information related to contact with caregivers. This information included the number of contacts, duration, and method (e.g., telephone, face-to-face, group, access to computerized information system). The form was completed by the staff member involved in the contact; this individual also documented whether the contact was scheduled or unscheduled, who initiated the contact, who was involved in the contact (e.g., other family members, other professionals, the care recipient), and if the contact was "off protocol." This information was immediately entered into the REACH core database by data entry staff. A record of contacts allowed investigators to analyze outcomes based on type, number, and duration of contacts. It also allowed the supervisor to provide feedback to the interventionists about the degree to which the intervention was delivered to and received by the client. (12)	9 - Change infrastructure	12, 22
			The advisory committee also developed strategies for program evaluation (27)	1 - Use evaluative and iterative strategies	27

			A CMHA-run " hotline " was made available to case managers 2.5 days per week. During these times, either the UA project manager or the principal investigator was available to discuss unusual or difficult cases. Additionally, a project director at ADSS facilitated monthly conference calls attended by the case managers, UA staff (principal investigator and project manager), and ADSS staff (commissioner, grants specialist, and division chief). The conference calls were intended primarily to play a trans-project coordinating role; however, approximately half of each call was devoted to consultation on difficult cases in the field. (33, 8)	2- Provide interactive assistance	33, 8
			The goal of the advisory committee was to modify the intervention for feasibility. This included a reduction in available treatment components, a reduced number of home visits, and a shortened time span of the intervention. (63, 51)<br><br>During the first 4 months of the project, the advisory committee held a series of face-to-face meetings and conference calls to select REACH treatment components in response to unmet needs of their dementia caregiver clients and the available human and financial resources within the participating organizations. In the following 5-month period, CMHA staff, with consultation from the advisory committee, adapted materials from REACH II to create a tailored community intervention program including trainer and caregiver manuals. (63)	3 - Adapt and tailor to context	63, 51
REACH OUT (Offering Useful Treatments) - Adaptation of REACH II for use in Area Agencies on Aging	Burgio et al. (2009) / USA	Case managers from AAA -Area Agencies on Aging … / UA's Center for Mental Health and Aging	Alabama's Commissioner on Aging (Irene B. Collins) for the Alabama Department of Senior Services (ADSS) was aware that the University of Alabama (UA) was one of the REACH study sites and contacted Louis D. Burgio, PhD, principal investigator, to propose a partnership between ADSS and the university to develop a community intervention based on REACH. (47, 24, 6)<br><br>During the "partnering for translation" phase of the project, the full REACH II clinical trial intervention was presented to the advisory board (see below). (…) An advisory committee was formed that included the commissioner on aging, AAA directors and case managers, and the principle investigator and project manager from the university. (40, 64)<br><br>A standard written form was used for all behavioral prescriptions, emphasizing specific definition of the problem, the goal of the prescription, strategies for preventing the behavior, and therapeutic responses when the problem occurred. In designing this material, dozens of generic prescriptions were available from REACH II for behaviors such as wandering, aggression, and difficulty with personal hygiene. (25)	4 - Develop stakeholder interrelationships	47, 24, 6, 40, 64, 25
			Case managers taught caregivers how to use a booklet, America's Health Guide for Seniors and Caregivers (Securitec Publications, 2006), commonly called the " Health Passport " (available at www.securitec.com/products). The passport-sized booklet provided caregivers information about health maintenance activities (such as annual physical examinations) and a tool to record health information and health appointments for both themselves and the care recipients. (31)<br><br>With the caregiver's permission, the case manager toured the physical environment noting safety concerns, including such risks as the availability of weapons and sharp objects, excessive clutter, and types of shoes worn by the care recipient (e.g., rubber soles are preferable). At each subsequent contact, the case manager would " check in " to ascertain whether caregivers followed through with suggested changes.(16)<br><br>A standard written form was used for all behavioral prescriptions, emphasizing specific definition of the problem, the goal of the prescription, strategies for preventing the behavior, and therapeutic responses when the problem occurred. In designing this material, dozens of generic prescriptions were available from REACH II for behaviors such as wandering, aggression, and difficulty with personal hygiene. (31)	5 - Train and educate stakeholders	31, 16, 71, 43, 55

			**The University of Alabama CMHA provided training in the intervention and project procedures to 13 AAA case managers and their supervisors in a 2-day (12-hr) workshop at CMHA. (71-G5) [the university conducted the 12-hr case manager training work-shop and was available for phone consultation twice weekly.](71)**<br><br>It is critical to note that the material was presented didactically, but the emphasis was on active learning. Active learning is defined as including the audience in all aspects of training. Questions were encouraged, and both workshop leaders' modeling and participants' role-playing were used extensively. (43)<br><br>Training was repeated as new case managers were hired due to staff turnover. Detailed case manager training manuals and caregiver notebooks were provided to participants so that they could be used during the intervention sessions. (31)<br><br>The case managers were asked to conduct four hour-long home visits to introduce the treatment components to caregivers for a 3- to 4-month period. The home visits were supplemented by three therapeutic phone calls interspersed between each of the home visits. (43, 16, 55)		
			Case managers taught caregivers how to use a booklet, America's Health Guide for Seniors and Caregivers (Securitec Publications, 2006), commonly called the " Health Passport " (available at www.securitec.com/products). The passport-sized booklet provided caregivers information about health maintenance activities (such as annual physical examinations) and a tool to record health information and health appointments for both themselves and the care recipients. (12)<br><br>With the caregiver's permission, the case manager toured the physical environment noting safety concerns, including such risks as the availability of weapons and sharp objects, excessive clutter, and types of shoes worn by the care recipient (e.g., rubber soles are preferable). At each subsequent contact, the case manager would " check in " to ascertain whether caregivers followed through with suggested changes. (11)	9 - Change infrastructure	12, 11
REACH-TX is a community-based translation of REACH II	Cho et al. (2019) / USA	Certified dementia care specialists/dementia interventionists	After receiving referrals to the program from numerous com-munity sources, AANCC staff implementing the REACH-TX program contacted potential participants to describe the program and conduct eligibility screening which included administration of the RAM. When it was determined that the participant was eligible and agreed to participate, basic demo-graphic information was obtained over the phone and an initial home visit was scheduled within 2 weeks of the call. (4)<br><br>The determination for cutoff values were driven by a number of considerations. First, there was existing evidence that other versions of the REACH protocol using two fewer sessions still delivered similar outcomes in health care set-tings (Stevens et al., 2012; Nichols et al., 2016). Second, maximizing the feasibility of program implementation through reduction of therapeutic contacts was a recur-rent theme during planning discussions with community-based organizations charged with delivering the program. Finally, there were practical considerations for reducing the number of therapeutic contacts related to the funding constraints imposed by the funding agency and the need to service a sufficient number of clients to meet the initiatives broad goals. (56)<br><br>The Risk Appraisal Measure. (RAM) assessed personal/environmental challenges and needs that could contribute to negative outcomes for caregivers and increase the risk of placing a patient with dementia in an institutional setting. (4)	1 - Use evaluative and iterative strategies	4, 56
			Moreover, ongoing communication between the community agency and an evaluation team played a significant role in improving the DCSs' implementation skills as well as sustaining a partnership with the local funding agency for longer periods of time (i.e., 7 years). (47, 52)	4 - Develop stakeholder interrelationships	47, 52

			For REACH-TX, certified dementia care specialists (DCS; the community term for dementia interventionists), who were trained by the one of the coauthors, delivered the intervention for 6 months in English or Spanish via in-home and telephone sessions. (71, 55)<br><br>Educational information and skill-training tools in REACH-TX were provided via A Caregiver's Notebook (Stevens, Trickett, Smith, & Lancer, 2009), which contains problem-solving strategies and referral information (e.g., local caregiver support groups) on each component. (31, 43)<br><br>DCSs developed a personalized "Family Profile" using the RAM to prioritize and address issues in domains showing the highest risk. (29)<br><br>Caregivers were also encouraged to attend support groups and were provided relevant information from library handouts and other resources. (15)<br><br>The A Caregiver's Notebook included educational materials, interactive modules, and worksheets corresponding to all target areas of the original REACH II. This approach also provided the family care-giver with similar information delivered by a computerized telephone system used in a clinical trial (Belle et al., 2006). (43)	5 - Train and educate stakeholders	71, 55, 15, 43, 31, 29
iMCSP	Droes et al. (2019) / the Netherlands	Meeting Centers	For the implementation of DemenTalent, the training consisted of information on the implementation process (e.g., How to involve people with dementia in the implementation? How to uncover talents of people with dementia? How to involve workplaces? How to match volunteers to workplaces? Communication plan, collaboration network, financing) and information on the content of DemenTalent (eg, looking at abilities of people instead of care needs only, out-of-the-box thinking, change in attitude of professional caregivers, evaluation and monitoring). (4, 18)	1 - Use evaluative and iterative strategies	4, 18
			Information on the content of the STAR e-Learning course was provided to the Meeting Centers by VUmc, which also arranged the course registration of prospective participants and provided a technical helpdesk for support. (24)<br><br>To implement the new interventions, the staff of the Meeting Centers who were responsible for the implementation received a two-day training, followed by "coaching on the job" provided by the private company Dirkse Anders Zorgen (DAZ), the Dementelcoach cooperative association, and the Amsterdam Center on Aging of VU University Medical Center (VUmc). (6)<br><br>Furthermore, DAZ provided coaching for the quartermaster activities, such as exploring local partners, workplaces for volunteers, and specific needs and wishes of people with dementia; guiding, supporting, and training of local partners at workplaces/companies who were not familiar with dementia; and finding a professional project leader and a project leader with dementia. (35, 7)<br><br>The Dementelcoach cooperative association offered the Meeting Centers guidance on how to inform potential participants and referrers on this new support intervention for caregivers (by means of making local brochures and face-to-face contact and press releases) and explained the procedure of how to refer to the national Dementelcoach office to match participants and coaches. (35)	4 - Develop stakeholder interrelationships	24, 6, 35, 7
			Furthermore, DAZ provided coaching for the quartermaster activities, such as exploring local partners, workplaces for volunteers, and specific needs and wishes of people with dementia; guiding, supporting, and training of local partners at workplaces/companies who were not familiar with dementia; and finding a professional project leader and a project leader with dementia. (71)	5 - Train and educate stakeholders	19, 71

			To implement the new interventions, the staff of the Meeting Centers who were responsible for the implementation received a two-day training, followed by "coaching on the job" provided by the private company Dirkse Anders Zorgen (DAZ), the Dementelcoach cooperative association, and the Amsterdam Center on Aging of VU University Medical Center (VUmc). (71)<br><br>To implement the new interventions, the staff of the Meeting Centers who were responsible for the implementation received a two-day training, followed by "coaching on the job" provided by the private company Dirkse Anders Zorgen (DAZ), the Dementelcoach cooperative association, and the Amsterdam Center on Aging of VU University Medical Center (VUmc). (19)		
			The Dementelcoach cooperative association offered the Meeting Centers guidance on how to inform potential participants and referrers on this new support intervention for caregivers (by means of making local brochures and face-to-face contact and press releases) and explained the procedure of how to refer to the national Dementelcoach office to match participants and coaches. (69)	7 - Engage consumers	69
			In addition, support was provided to all Meeting Centers in the experimental group for the acquisition of (start-up and structural) funding from the municipalities (within the framework of the Social Support Act, WMO) for the implementation of the three new iMCSP interventions. (1)	8 - Utilize financial strategies	1
Care of Persons with Dementia in their Environment (COPE) integrated in Connecticut Home Care Programfor Elders (CHCPE)	Fortinsky et al. (2016) / USA	Care managers, advanced practice nurses, and occupational therapists	OTs initially interview CGs to identify client routines, previous and current roles, habits and interests, and CG concerns. OTs also conduct cognitive and functional testing to identify client capacities and deficits in cognitive functioning, including attention, initiation/perseveration, construction, conceptualization, memory, planning and problem solving, as well as in physical functioning, including fall risks and mobility performance. (4, 18)<br><br>In these instances, when the client returns home, care managers routinely re-evaluate the entire care plan and make changes accordingly. Accordingly, the COPE interventionists will re-evaluate such clients upon their return home and adjust COPE prescriptions as appropriate to the client's and CG's situation at that time. (5)<br><br>The CCCI electronic database is a repository of all clients' approved CHCPE care plan services, hospitalizations, ED visits, rehabilitation nursing home stays, and long-term nursing home stays, all updated monthly by CCCI care managers. All data stored in the CCCI database pertaining to use of these services for the 12 months following randomization will be linked to data from other study sources at the client level. Service use data from the CCCI database also will be used by investigators responsible for constructing analytic variables for purposes of conducting economic analyses. (26)	1 - Use evaluative and iterative strategies	4, 18, 5, 26
			OTs then train CGs how to modify home environments, simplify daily activities, and communicate effectively to support client capabilities, use problem-solving to identify solutions for CG-identified concerns (e.g., behavioral challenges, difficulties managing patient self-care), and use stress reduction techniques to lower their own distress. (33)<br><br>Care managers will also notify COPE interventionists if they learn, in the course of routine care plan monitoring activities, that a client experiences a change in location due to hospitalization or short-term nursing home admission. (53, 54)	2- Provide interactive assistance	33, 54, 53
			For each targeted concern jointly identified by CG and OT, a written action plan, referred to as a "COPE prescription", is devised describing treatment goals, client capacities, and specific strategies for the CG to implement. (63)	3 - Adapt and tailor to context	63
			In order to embed COPE within the routine operation of the Connecticut Home Care Program for Elders (CHCPE), which is essential to the translational effort of this study, the care manager responsible for the total care plan for each CHCPE client will be notified by the study coordinator when a client is assigned to the intervention group, as well as the name of the	4 - Develop stakeholder interrelationships	52, 6

			assigned OT and APN for that client. This will trigger contact between the care manager and assigned COPE interventionists, so that the care manager can help insure that interventionists do not visit client homes at the same time as service providers already providing care to clients per their approved care plans and that care managers can be in-formed of pertinent clinical information as needed (e.g., positive laboratory results). (52, 6)		
			COPE dyads receive up to 10 sessions over 4 months by occupational therapists (OT);and 1 face-to-face and 1 telephone session by an advanced practice nurse (APN). (43)<br><br>For each targeted concern jointly identified by CG and OT, a written action plan, referred to as a "COPE prescription", is devised describing treatment goals, client capacities, and specific strategies for the CG to implement. (29, 31)<br><br>In a separate home visit, the APN provides CGs with information to help them identify and monitor common health-related concerns (pain detection, hydration, constipation, medication management), and how to be a medical advocate. The APN also obtains blood/urine samples from the client, and examines the client for signs of dehydration. Laboratory evaluations include complete blood count, blood chemistry, and thyroid testing of serum samples, and culture and sensitivity of urine samples. Client medications are also reviewed for polypharmacy and dosing appropriateness. The purpose of these clinical tests is to rule out underlying medical conditions, infections or medication issues that may be negatively contributing to functioning at home. (16)	5 - Train and educate stakeholders	43, 29, 31, 16
			The APN informs CGs by telephone of laboratory results within 48 h and mails two copies of the results to CGs (one for their records and the other copy to share with clients' physicians). For positive laboratory results, CGs are asked if they prefer the APN to fax results to the physician and/or care manager, and discuss with them directly. A study geriatrician will be available to consult with the APN regarding any signs, symptoms, and laboratory tests requiring further clinical interpretation before the APN provides her assessments to CGs. (21, 32)<br><br>In order to embed COPE within the routine operation of the Connecticut Home Care Program for Elders (CHCPE), which is essential to the translational effort of this study, the care manager responsible for the total care plan for each CHCPE client will be notified by the study coordinator when a client is assigned to the intervention group, as well as the name of the assigned OT and APN for that client. This will trigger contact between the care manager and assigned COPE interventionists, so that the care manager can help insure that interventionists do not visit client homes at the same time as service providers already providing care to clients per their approved care plans and that care managers can be in-formed of pertinent clinical information as needed (e.g., positive laboratory results). (30, 59)	6 - Support clinicians	21, 32, 30, 59
			OTs then train CGs how to modify home environments, simplify daily activities, and communicate effectively to support client capabilities, use problem-solving to identify solutions for CG-identified concerns (e.g., behavioral challenges, difficulties managing patient self-care), and use stress reduction techniques to lower their own distress. (11)<br><br>The CCCI electronic database is a repository of all clients' approved CHCPE care plan services, hospitalizations, ED visits, rehabilitation nursing home stays, and long-term nursing home stays, all updated monthly by CCCI care managers. All data stored in the CCCI database pertaining to use of these services for the 12 months following randomization will be linked to data from other study sources at the client level. Service use data from the CCCI database also will be used by investigators responsible for constructing analytic variables for purposes of conducting economic analyses. (12)	9 - Change infrastructure	11, 12
NYU Caregiver-Adult Child Intervention	Gaugler (2018) / USA	Occupational therapist	Following the initial 4-month counseling period, spousal caregivers in the original study were referred to weekly support groups. In addition, NYUCI counselors are available to caregivers and families over the telephone or in-person (i.e., "ad hoc counseling") to help them deal with crises and the changing nature of the person with dementia's symptoms. (33)	2- Provide interactive assistance	33

			Following the initial 4-month counseling period, spousal caregivers in the original study were referred to weekly support groups. In addition, NYUCI counselors are available to caregivers and families over the telephone or in-person (i.e., “ad hoc counseling”) to help them deal with crises and the changing nature of the person with dementia’s symptoms. (43)<br><br>The NYUCI consists of three components: individual and family counseling, support groups, and ad hoc counseling. Six individual and family sessions are provided in the first 4 months of participation: one individual session with the spousal caregiver; four sessions that include the spousal caregiver and at least one other family member; and a second individual session with the spousal caregiver. (19)	5 - Train and educate stakeholders	43, 19
			The major aims of the counseling sessions include improving social support from family members and friends, education, promotion of communication, problem solving, strategies for responding to care recipient behavior, and concrete planning. (50)	7 - Engage consumers	50
Unforgettable (interactive museum program)	Hendriks et al. (2018) / The Netherlands	Led by a specially trained museum guide, who leads the discussion during the tour, and one or two trained hosts or hostesses, who assist the guide and/or support the participants.	After having first very positive experiences with the program in their own museum, the two Dutch museums decided to start a national implementation study. (61)<br><br>Because implementation of psychosocial interventions appears to not always be adequate in care practice, and successful implementation of psychosocial support often requires adaptive implementation, one of the aims of the implementation study was to get insight into the facilitators and barriers of successful implementation and possible solutions for barriers. (4)<br><br>This paper reports on the study that was carried out into the implementation of the Unforgettable program in 12 museums across the Netherlands. (61)	1 - Use evaluative and iterative strategies	61, 4
			The program was modeled after the successful Meet Me at MoMA program of the Museum of Modern Art in New York, which showed positive effects on people with dementia (positive change in mood, higher self-esteem) and their caregivers (positive change in mood, increased feeling of social support, less emotional problems) (63, 51)	3 - Adapt and tailor to context	63, 51
			The implementation project was led by a national project coordinator who was appointed by the Stedelijk Museum and the Van Abbemuseum (57, 6)<br><br>The implementation study was designed and conducted by the department of Psychiatry of the VU University medical center. (24)<br><br>This paper reports on the study that was carried out into the implementation of the Unforgettable program in 12 museums across the Netherlands. (72, 36)<br><br>However, one of the museum program coordinators experienced interest in their program by other museums in their region not involved in the implementation project. (72)	4 - Develop stakeholder interrelationships	57, 6, 24, 72, 36
			The Unforgettable program comprises an interactive guided museum tour, especially designed for people with dementia and their informal caregivers. (43)<br><br>The Unforgettable program is led by a specially trained museum guide, who leads the discussion during the tour, and one or two trained hosts or hostesses, who assist the guide and/or support the participants. The training was given by the project coordinator of the Unforgettable program (71)<br><br>For the guides, the training started with a two-day introduction in which information about the occurrence, symptoms, and consequences of dementia, and information about the Unforgettable method were provided. (71)<br><br>In the weeks following the two-day introduction, each trainee gave one pilot tour with people with dementia and caregivers participating. The hosts received a one-day introduction course about dementia and the Unforgettable method and joined a pilot tour. (71)	5 - Train and educate stakeholders	43, 71

			For the guides, the training started with a two-day introduction in which information about the occurrence, symptoms, and consequences of dementia, and information about the Unforgettable method were provided. (59)<br><br>Another museum already shared its knowledge and experience with a museum in their region. Two Unforgettable museums planned to share their knowledge with other museums through a platform and through a one-time meeting, respectively. (30)	6 - Support clinicians	59, 30
			In 2013, two Dutch museums, the "Stedelijk Museum Amsterdam" and the "Van Abbemu-seum" in Eindhoven implemented the Dutch "Unforgettable" program consisting of interactive guided museum tours for people living with dementia and their caregivers. (41)	7 - Engage consumers	41
RDAD	Menne et al. (2014) / USA	Interventionists delivering RDAD were clinical staff from the seven Alzheimer's Association chapters that served the state.	Although the core components of RDAD remained the same, some adaptations to the delivery of the program were made for this statewide implementation. (63, 51)	3 - Adapt and tailor to context	63, 51
			This replication of RDAD is a collaborative project between the Ohio Department of Aging, Alzheimer's Association Chapters serving Ohio, and the Benjamin Rose Institute on Aging, in partnership with the original RDAD researcher, Linda Teri, PhD. (24)<br><br>Alzheimer's Association chapters in Ohio promoted RDAD and recruited potential participants in a manner similar to how these chapters would promote their other service programs, such as through Help Line (information and referral) calls, flyers at community events, and presentations to local agencies and groups. (52)	4 - Develop stakeholder interrelationships	24, 52
			For the Ohio replication, the interventionists delivering RDAD were clinical staff from the seven Alzheimer's Association chapters that served the state, hereafter referred to as trainers. (59)	6 - Support clinicians	59
			Ohio trainers came from a variety of disciplines, including nursing, social work, counseling, and gerontology, and functioned as regular employees of Alzheimer's Association chapters. The goal was to implement RDAD as part of usual Alzheimer's Association services. (44)	9 - Change infrastructure	44
Savvy Caregiver + REACH II	Meyer et al. (2018) / USA	ACC Senior Services and Asian Resources, Inc.	The intervention is a six-week multicomponent program, structured into weekly two-hour sessions and is meant to reduce stress and promote culturally appropriate coping strategies. (33)	2- Provide interactive assistance	33
			Themes that emerged from recruitment and engagement into the intervention included the importance of faith-based institutions, the inclusion of multiple family members in the intervention, community empowerment/ownership, and the importance of credibility. (38)	4 - Develop stakeholder interrelationships	38
			The intervention is a six-week multicomponent program, structured into weekly two-hour sessions and is meant to reduce stress and promote culturally appropriate coping strategies. (19)	5 - Train and educate stakeholders	19
			the interventionist needed to have a solid understanding of who family members were, how they were involved in caregiving, and the dynamics of the family. Because family members each enacted different roles and responsibilities in the caregiving, it was important to understand and address multiple family members in the intervention. (39, 41)	7 - Engage consumers	39, 41
multi-component non-pharmacological intervention (NPI)	Milders et al. (2016) / UK	Fourteen trainers were recruited from charities and support organisations in the Northeast of Scotland. All trainers had experience of dementia care, but varied in their levels of education and experience. Only three trainers were qualified health care professionals.<br><br>The participants were recruited via the care team of the person with dementia, the database of	During the last two sessions caregivers had the opportunity to discuss progress or difficulties implementing the activities at home. (5, 56)	1 - Use evaluative and iterative strategies	5, 56
			The importance of a person-centred approach was emphasised, including tailoring activities to suit the individual and choosing activities that the person with dementia enjoyed. (51, 63)<br><br>The caregiver training addressed the components of the intervention and caregivers were shown how to use the manual and how to engage the person with dementia in stimulatingactivities. (51, 63))	3 - Adapt and tailor to context	51, 63
			Fourteen trainers were recruited from charities and support organisations in the Northeast of Scotland. All trainers had experience of dementia care, but varied in their levels of education and experience. Only three trainers were qualified health care professionals. (57)<br><br>The Intervention and manual had been developed in consultation with health care professionals, people with dementia, caregivers and representatives of dementia organisations. (64)	4 - Develop stakeholder interrelationships	57, 64, 52

		the ScottishDementia Clinical Research Network or locally through health and social careorganisations.	The participants were recruited via the care team of the person with dementia, the database of the Scottish Dementia Clinical Research Network or locally through health and social care organisations.(52)		
			All trainers were instructed by the research coordinator. Trainers were also provided with a trainers' manual containing step-by-step instructions on how to deliver the caregiver training. (31)<br><br>The instructions emphasised a person-centred approach, to individualize activities to suit the interests, experience and abilities of the person with dementia and caregiver. Trainers were instructed in a single session lasting approximately two hours. In total there were seven training sessions with one to four trainers per session. (71, 19)<br><br>The manual-based intervention comprised several components: educating caregivers on dementia and living with dementia, training caregivers in effective communication, coping with stress and training caregivers to stimulate the person with dementia both physically and mentally through activities. The manual for caregivers presented the above components and contained detailed descriptions of activities that the caregiver could do at home with the person with dementia, including reminiscence activities, household tasks, quizzes and light physical exercises. The manual also contained resource materials that could be used during the activities, e.g. crosswords, photographs. (43, 31)<br><br>The Intervention and manual had been developed in consultation with health care professionals, people with dementia, caregivers and representatives of dementia organisations. (29)<br><br>The caregiver training addressed the components of the intervention and caregivers were shown how to use the manual and how to engage the person with dementia in stimulating activities. (19)<br><br>Caregiver training took place in eight small groups of two to four caregivers, led by two trainers, over four weekly sessions lasting approximately 90 minutes each. (19)	5 - Train and educate stakeholders	31, 19, 71, 43, 29
New York University Caregiver Intervention (NYUCI) - Minnesota Family Memory Care	Mittelman and Bartel (2014) / USA	administered by the Minnesota Board on Aging and the Minnesota Department of Human Services.	Regular biweekly group-based telephone supervision was provided by the NYUCI team for the first eighteen months of the FMC program. Subsequently, supervision was provided by a Minnesota-based clinician who participated in quarterly calls with the NYUCI team. (48)<br><br>Seventeen counselors were trained in the NYUCI and participated in the FMC program. Nine of them had a master's degree in social work or an allied profession: Four were licensed clinical social workers. The other eight counselors had bachelor's degrees in social work, psychology, or occupational therapy.(35)<br><br>The Service was marketed locally by the sites through their network of referral sources, educational activities of consultants, radio programs, news-paper articles, and newsletters. (52)	4 - Develop stakeholder interrelationships	52, 35, 48
			In the first phase of implementation, counselors at the original four FMC sites participated in a day and a half of in-person training provided by Mittelman and Epstein. The second cohort of counselors was initially trained by the previously trained Minnesota providers. Twelve months later, the counselors in the second and third cohorts received in-person training from Epstein. (19)<br><br>Prior to receiving in-person training, the counselors participated in three discussion sessions of the book describing the NYUCI. (71)	5 - Train and educate stakeholders	19, 71
			Counselors were chosen from among the social service providers serving these caregivers. Marketing materials were developed jointly by the Minnesota Board on Aging and the sites. (59)	6 - Support clinicians	59

			The Service was marketed locally by the sites through their network of referral sources, educational activities of consultants, radio programs, news-paper articles, and newsletters. (69)	7 - Engage consumers	69
			Evidence of the effectiveness of the NYUCI led the Administration on Aging to fund translations of the NYUCI in six states—Minnesota, Florida, Georgia, California, Wisconsin, and Utah—through the Alzheimer's Disease Supportive Services Program. Minnesota was the earliest, and is the longest-running, implementation. (1, 34)<br><br>To ensure its sustainability after the grant funds ended, the FMC program has been embedded within the statewide network of caregiver consultation services funded by the Older Americans Act of 1965. (49)<br><br>Thus, in the NYUCI translation funded by the Alzheimer's Disease Supportive Services Program, participation in six sessions was the goal, but completion was defined as participation in a minimum of four sessions. (34)	8 - Utilize financial strategies	1, 34, 49
			Status tracking forms for each caregiver were filled out and updated by each counselor and submitted quarterly to the Minnesota Board on Aging. Information about the number of counseling sessions, dates of nursing home placement and death, and reasons for discontinuing the intervention was extracted from these forms and merged into the outcome database that was provided to the authors. (12)	9 - Change infrastructure	12
REACH VA	Nichols et al. (2011) / USA	REACH VA was based in Home-Based Primary Care (HBPC) programs, which provide primary and specialized care to home-bound veterans.	The interventionist taught problem solving and provided action-oriented behavioral strategies to address caregiving problems or patient behaviors identified by the risk assessment using a caregiver notebook. The notebook was written at a fifth-grade reading level, with educational information and practical strategies for 30 behavioral and 18 stress/coping topics that could be personalized for the caregiver. (33)	2- Provide interactive assistance	33
			Through the auspices of the VHA's Office of Home and Com-munity Care in Geriatrics and Extended Care, REACH VA was based in Home-Based Primary Care (HBPC) programs, which provide primary and specialized care to home-bound veterans. (6)	4 - Develop stakeholder interrelationships	6
			Members of the HBPC staff from 24 facilities in 15 states (Table1) were trained and certified using Live Meeting by members of the staff of the Memphis VA Medical Center (VAMC), Memphis, Tennessee, including Memphis REACH investigators(L.O.N. and J.M.-A.) as well as other REACH site investigators (Lou Burgio, David Coon, Sara Czaja, and Dolores Gallagher-Thompson). (71)<br><br>The interventionist also provided training on stress management (signal breath, stretching, guided imagery, pleas-ant events, and mood management). The structured telephone support group sessions with 5 to 6 caregivers and a leader provided support, skills, and education on self-care, resources, financial and legal issues, and communication with patient and service providers. (43)<br><br>The interventionist taught problem solving and provided action-oriented behavioral strategies to address caregiving problems or patient behaviors identified by the risk assessment using a caregiver notebook. The notebook was written at a fifth-grade reading level, with educational information and practical strategies for 30 behavioral and 18 stress/coping topics that could be personalized for the caregiver. (31)	5 - Train and educate stakeholders	71, 43, 31
			The HBPC staff members provided the intervention in addition to their usual clinical duties. (59)<br><br>There were 78 staff members who were certified as an interventionist (n=35), a support group leader (n=12), or both (n=31);however, only 53 staff members actually performed the intervention. For these, education ranged from associate to doctoral degrees, with 44 staff members holding a master's degree or higher. Most (n=51) were social workers, psychologists, or nurses. (59)	6 - Support clinicians	59

			To investigate the feasibility of extending caregiver assistance into the Veterans Health Administration (VHA), VHA Patient Care Services, National Caregiver Support Program, through Public Law 109-461, funded a clinical translation of the REACH II RCT from September 2007 through August 2009. (34)	8 - Utilize financial strategies	34
REACH VA	Nichols et al. (2016) / USA	Many of the interventionists were mental health professionals newly integrated into HBPC	The purpose of exploration is to determine if the new evidence-based practice is needed, assess the potential match between the organization and the practice, and determine whether or not the practice can be implemented. (4)<br><br>Staff at the VA Medical Center at Memphis translated the REACH II research materials for clinical care use (intervention manuals, scripts, certification procedures, and evaluation materials), developed comprehensive caregiver materials for each topic, developed training materials, trained and coached clinical staff, and collected data from caregivers and staff for evaluation. (56, 4)<br><br>In June 2012, a modified REACH intervention, REACH VA Program Phase 2 was rolled out to the VHA system based on staff and caregiver comments. Staff identified stressed and burdened caregivers of patients from their panels based on their clinical judgment. The modified inter-vention has four core sessions during two to three months, with the option for additional sessions based on caregiver need, desires, goal attainment, and clinician judgment. (4, 56)<br><br>Since roll-out of the four session model, the Memphis Caregiver Center has provided training, certification, and coaching to 151 VA sites in providing the intervention, a 500% increase, with 444 staff trained and 265 certified. (61)<br><br>Timing of implementation is critical. When the clinicians were ready, a facility administration might have other priorities and when leadership was ready, clinicians might not be ready. Securing coordination became part of site readiness preparation—to encourage discussion between clinicians and administration before training and certification.(4)<br><br>The intervention had been reduced from twelve to four sessions, telephone support groups were optional, comple-mentary, or standalone, and the mode of delivery could be face to face or by telephone or telehealth. (14)	1 - Use evaluative and iterative strategies	4, 56, 61, 14
			Technical assistance calls were regularly scheduled. (8)	2- Provide interactive assistance	8
			Administration on all levels must buy into the intervention. It is not enough for top level leadership, in our case, Congress or VHA, to be supportive of the program, although this certainly helps. Local facility and clinic leadership and local clinicians must all be onboard. (47, 17)<br><br>Implementation leaders must work to ensure that the intervention survives and remains effective without losing its essential components. (35)	4 - Develop stakeholder interrelationships	47, 17, 35
			Staff at the VA Medical Center at Memphis translated the REACH II research materials for clinical care use (intervention manu-als, scripts, certification procedures, and evaluation materi-als), developed comprehensive caregiver materials for each topic, developed training materials, trained and coached clinical staff, and collected data from caregivers and staff for evaluation. (29)<br><br>The intervention was 12 individual sessions plus five telephone support groups. (19)<br><br>Training and coaching implementation drivers were important during this period. At the onset of implementation, training sessions were archived online to provide on-demand and just in time training. (43)<br><br>The intervention can be offered in the home, in facility or clinic, or by telephone or telehealth modalities. (43)	5 - Train and educate stakeholders	29, 19, 43, 31, 71

			The Caregiver Notebook from the REACH VA translation, which includes 48 behavior and coping topics, continues to be an important source of strategies for action. (31)<br><br>The telephone support groups can now be a complementary or standalone intervention. (19)<br><br>The Caregiver Center included more role-playing, feedback, and examples to help staff better understand the intervention and their role. Certifying interventionists by videoconferencing has been extremely useful because it allows immediate feedback as interventionists role-play the intervention (71)		
			Many of the interventionists were mental health professionals newly integrated into HBPC (Karlin & Karel, 2014). (59)<br><br>The Caregiver Center included more role-playing, feedback, and examples to help staff better understand the intervention and their role. Certifying interventionists by videoconferencing has been extremely useful because it allows immediate feedback as interventionists role-play the intervention. (59)	6 - Support clinicians	59
			The Caregiver Center continues to train staff at new sites but also provides re-training for sites where certified staff have left. (57-G4?) In addition to re-training staff at these sites, new marketing must take place to highlight the benefits of the intervention and assistance must be provided in placing the intervention in the appropriate context in the clinical setting. (69, 37)	7 - Engage consumers	69, 37
			During program installation, the organization begins to function differently and install structural supports. This infrastructure will likely include funding for staff training and for implementation. (34)<br><br>Another important factor for implementation success was workload capture. The only care the VA previously provided was Veteran care, so there has been no administrative support for how to structure care and workload and obtain facility reimbursement. The Congressionally mandated part of caregiver support for caregivers of seriously injured post 9/11 veterans who were receiving stipends had separate rules and regulations that did not necessarily apply to car-egivers who were receiving REACH VA services or other non-mandated services. However, workload credit was extremely important in determining whether the program would and could be implemented at a site. The Caregiver Center became a clearinghouse for informal guidance to sites on coding caregiver services for reimbursement. (49)	8 - Utilize financial strategies	34, 49
			Since roll-out of the four session model, the Memphis Caregiver Center has provided training, certification, and coaching to 151 VA sites in providing the intervention, a 500% increase, with 444 staff trained and 265 certified. (22)<br><br>In 2007–2009, VHA Patient Care Services initiated The Pilot Program for Caregiver Assistance in response to Public Law 109–461, the Veterans Benefits, Health Care, and Information Technology Act of 2006 that required VHA to evaluate "feasibility and advisability" of approaches to improve and expand services that provide assistance to caregivers. (44)	9 - Change infrastructure	22, 44
SHARE Program	Orsulic-Jeras et al. (2016) / USA	SHARE Counselors (n¼5) included one master's level Counseling graduate with over15 years of experience working with older adults and PWDs, and four counselor	The core of this intervention centers around the initial assessment (before the sessions begin) of the PWD's care values and preferences and the CG's perceptions of those care values and preferences. (4)	1 - Use evaluative and iterative strategies	4
			With Assistance from a SHARE Counselor, this approach creates a safe and comfortable environment that enables care dyads to work at their own level of readiness to discuss and plan for what lies ahead. (33)	2- Provide interactive assistance	33
			Once this strong foundation is built, the focus moves to enhancing the care dyad's communication skills, increasing their knowledge about dementia and available services in the	5 - Train and educate stakeholders	19, 59, 71, 55, 31 , 43

		trainees studying for a master's degree who had no previous experience with the study population.	community, and ultimately developing a mutually agreed-upon future plan of care. (19)<br><br>All SHARE Counselors received extensive training from project staff on counseling aging persons, dementia signs and symptoms, caregiving, care values and preferences, and working with a dyadic unit. The SHARE Counselors also received training on SHARE protocols and procedures, and participated in weekly one-hour face-to-face supervision sessions with the Principal Investigator (PI) and the Project Director (PD) to maintain fidelity to study protocols and prevent drift. (71)<br><br>Supervision also helped SHARE Counselors to ensure all content was covered while accommodating each dyad's tolerance for discussing sensitive topics. In addition, counselor trainees met weekly with their licensed counseling supervisor to complete their clinical training requirements. (55)<br><br>Materials provided to SHARE Counselors included a SHARE Counselor Manual, Counselor's Guide, and set of magnetic boards used to facilitate the care values and preferences discussions. (31)<br><br>Prior to Session 2, the PWD's and the CG's CVS responses are used to generate individualized Care Values Magnet Boards,a visual representation of the PWD's care values and the CG's perceptions of the PWD's care values. This exercise helps to delineate PWDs' values for care while they still have the capacity to assert and discuss them. (43)		
			All SHARE Counselors received extensive training from project staff on counseling aging persons, dementia signs and symptoms, caregiving, care values and preferences, and working with a dyadic unit. The SHARE Counselors also received training on SHARE protocols and procedures, and participated in weekly one-hour face-to-face supervision sessions with the Principal Investigator (PI) and the Project Director (PD) to maintain fidelity to study protocols and prevent drift. (59)	6 - Support clinicians	59
New York University Caregiver Intervention (NYUCI) - Minnesota Family Memory Care	Paone (2014) / USA	Eighteen FMC Consultants were trained in the program protocol and in all of the tools and processes.<br><br>14 Minnesota organizations (program sites)	The State provided a clinical consultant (clinical neuropsychologist) who provided regular case reviews to monitor and mentor the FMC Consultants. (27)	1 - Use evaluative and iterative strategies	27
			During family sessions, the consultants worked with caregivers and families to help them respond to psycho-social, emotional and behavioral issues of the person with the disease, caregiver, and extended family members. (33)	2- Provide interactive assistance	33
			Minnesota consultants described the substantial training and clinical consultant mentoring that was provided through the State as "extremely important" to them for program adoption and ongoing implementation and to ensure adherence to the NYUCI program protocol. This training and mentoring by the NYUCI researchers likewise helped consultants "develop skills and build confidence." (65)	4 - Develop stakeholder interrelationships	65
			In this implementation experience, the availability of the research team to provide technical assistance to the FMC Consultants was invaluable. (55)<br><br>The State provided a clinical consultant (clinical neuropsychologist) who provided regular case reviews to monitor and mentor the FMC Consultants. (55)	5 - Train and educate stakeholders	55
			Most organizations did not hire a new staff member (five of the18 consultants were new hires). Program sites typically reassigned an existing staff member to provide the intervention and participate in the initiative. (59)	6 - Support clinicians	59
			Although all of the program sites actively pursued a variety of marketing and recruitment strategies to make the FMC program known to communities and caregivers, the total enrollment of 137 caregivers was less than was expected by program sites and state agents. (69)	7 - Engage consumers	69
			In Minnesota, the NYUCI program is called Family Memory Care(FMC).Implementation began in 2007 through a grant from the US Administration on Aging to the Minnesota Board on Aging. (1, 34)	8 - Utilize financial strategies	1, 34
			At start-up, all of the Minnesota sites had basic tools and resources at the disposal of the FMC Consultant, including office space, computer, telephone, and some administrative support	9 - Change infrastructure	11, 22

			(e.g., receptionist, human resources, payroll accounting, database and I.T. support, and/or supervisory support) for this program. (11)<br><br>The State also set a requirement for clinical supervision of the consultant. (22)		
MSCP	Samia et al. (2014) / USA	The MSCP commenced in November 2008 with the training of one lead and 11 master trainers by one author (Kenneth Hepburn) through a Webinar Training sponsored by the Rosalynn Carter Institute.	Of the four original lead partners, two ADRC/AAAs participated fully in adopting the MSCP. They supplied the necessary infrastructure for training development and support, intake, screening, marketing, and program delivery. These agencies embedded the MSCP in their existing Family Caregiver Program (FCP). (61)	1 - Use evaluative and iterative strategies	61
			These trainers, the program evaluator, and the project manager formed the implementation team. (64)<br><br>The MSCP is a statewide community-based, caregiver training partnership between Maine's Office of Aging and Disability Services; the Alzheimer Association, Maine Chapter (Association); Maine's Aging and Disability Resource Centers/Agencies on Aging (ADRC/AAA); and the University of Southern Maine's School of Nursing. (24)<br><br>Caregivers were recruited by the partner agencies using flyers, public service advertisements, community outreach, e-mail, Web-site postings, program cross fertilization, and professional presentations. (52)<br><br>Marketing and recruitment were resource-intensive and greater success was experienced by the agencies with established relationships and champions in the location in which the training was offered. (35, 6)<br><br>Of the four original lead partners, two ADRC/AAAs participated fully in adopting the MSCP. They supplied the necessary infrastructure for training development and support, intake, screening, marketing, and program delivery. These agencies embedded the MSCP in their existing Family Caregiver Program (FCP). (36, 35)<br><br>They created unique delivery models based on existing community resources such as satellite offices and senior and community centers while also forging new partnerships to reach beyond the traditional aging services network where 74%(n=78) of the MSCP trainings were offered. (6)	4 - Develop stakeholder interrelationships	64, 24, 52, 35, 36, 6
			Planning involved creation of protocols, evaluation tools, schedules, and marketing strategies to accomplish the goals and objectives of the AoA grant. (29)<br><br>A formal train-the-trainer model with a specific protocol for associate trainer certification was developed and implemented. Associate training and certification involved attendance at a one-day workshop, observation of a 6-week MSCP workshop, and then co-facilitation of a 6-week workshop with master trainer observation and feedback. The original intent was to utilize both employee and volunteer trainers, who had dementia knowledge and experience, and ideally group facilitation experience. In total, 37 trainers were trained (12 master and 25 associate). All master trainers and 18 associate trainers were certified. (71)<br><br>The MSCP is a statewide community-based, caregiver training partnership between Maine's Office of Aging and Disability Services; the Alzheimer Association, Maine Chapter	5 - Train and educate stakeholders	29, 71, 73

			(Association); Maine's Aging and Disability Resource Centers/Agencies on Aging (ADRC/AAA); and the University of Southern Maine's School of Nursing. (73)		
			Caregivers were recruited by the partner agencies using flyers, pub-lic service advertisements, community outreach, e-mail, Web-site postings,program cross fertilization, and professional presentations. (69)	7 - Engage consumers	69
			The MSCP commenced in November 2008 with the training of one lead and 11 master trainers by one author (Kenneth Hepburn) through a Webinar Training sponsored by the Rosalynn Carter Institute. (1)	8 - Utilize financial strategies	1
			A formal train-the-trainer model with a specific protocol for associate trainer certification was developed and implemented. Associate training and certification involved attendance at a one-day workshop, observation of a 6-week MSCP workshop, and then co-facilitation of a 6-week workshop with master trainer observation and feedback. The original intent was to utilize both employee and volunteer trainers, who had dementia knowledge and experience, and ideally group facilitation experience. In total, 37 trainers were trained (12 master and 25 associate). All master trainers and 18 associate trainers were certified. (22)	9 - Change infrastructure	22
REACH II - implemented in Scott & White Family Caregiver Program (a non-profit collaborative healthcare system)	Stevens et al. (2012) / USA	The FCP targeted one large hospital (636 beds) and one large ambulatory internal medicine primary care clinic within the Scott & White system. Implementation of the project was facilitated by a partnership with the Central Texas AAA.	To ensure fidelity to the REACH II clinical trial, all FCP intervention materials including the treatment delivery schedule (described in the "Delivering the REACH II Intervention Components via the Caregiver Notebook and Family Profile" section) were based on the REACH II intervention materials. (23)<br><br>Likewise, the use of a brief risk assessment tool allowed a rapid introduction to the unique needs of each family.(4)<br><br>Ensuring that our program was designed to align with the existing Mission and Vision of Scott & White was critical to receiving buy-in from the leadership and management (56)	1 - Use evaluative and iterative strategies	23, 56, 4
			In-person contacts occurred in the Scott & White Family Resource Center, not in the family home as done in REACH II. This was also due to the resource limitations. The Family Resource Center was designed to be a welcoming and comfortable place for family caregivers to find support and education and is ideally located within a large Scott & White internal medicine primary care clinic to assure easy access and convenience for patients and family caregivers. (33)	2- Provide interactive assistance	33
			The intervention materials presented to caregivers in the REACH II clinical trial were extracted from the REACH II intervention manuals and reformatted into "A Caregiver's Notebook" (available from the corresponding author). (51)<br><br>The format of the Family Profile paralleled A Caregiver's Notebook and included the same nine sections. The inclusion of the Family Profile in A Caregiver's Notebook was to ensure that all intervention components used in REACH II would be available to all enrolled caregivers. (51)<br><br>This adaptation was driven by resource limitations and our desire to make the intervention schedule more flexible and responsive to the needs of the caregiver, i.e., the frequency of therapeutic contacts was a function of the level of risk reported by the caregiver. (63)<br><br>In-person contacts occurred in the Scott & White Family Resource Center, not in the family home as done in REACH II. This was also due to the resource limitations. The Family Resource Center was designed to be a welcoming and comfortable place for family caregivers to find support and education and is ideally located within a large Scott & White internal medicine primary care clinic to assure easy access and convenience for patients and family caregivers. (63)<br><br>Ensuring that our program was designed to align with the existing Mission and Vision of Scott & White was critical to receiving buy-in from the leadership and management (51)	3 - Adapt and tailor to context	51, 63

			In partnership with the Area Agency on Aging (AAA), we systematically translated the REACH II intervention into a nonprofit integrated healthcare system. Embedding the intervention within an integrated healthcare setting facilitates the identification and support of family members who care for individuals with dementia at numerous health services contact points. (52)<br><br>The FCP targeted one large hospital (636 beds) and one large ambulatory internal medicine primary care clinic within the Scott & White system. Implementation of the project was facilitated by a partnership with the Central Texas AAA. (47)<br><br>Training and oversight of FCP staff by a member of the REACH II research team promoted fidelity to the intervention as well as specific training in the techniques used with caregivers.(24)<br><br>Furthermore, referrals to formal community services, such as respite care, mental health counseling, and benefits and options counseling were made to the partnering agency, the Central Texas AAA. (52)<br><br>As delineated using the RE-AIM framework, the implementation process required action at multiple levels of the organization, including building partnerships within and outside of Scott & White Healthcare. (35, 6, 52)<br><br>For example, A Caregiver's Notebook, created in collaboration with the Scott & White Strategy and Marketing team, served not only as a vital resource to our individual caregivers but also as a branding tool on an organizational level, supporting the Mission and Vision of Scott & White Healthcare (48, 64)<br><br>Likewise, members of the Central Texas AAA, our community partner for the FCP, were needed to design the most efficient and effective method for receiving caregiver referrals. (52)	4 - Develop stakeholder interrelationships	52, 47, 24, 35, 6, 48, 64
			The five REACH II intervention components, delivered through home visits and therapeutic phone calls, identified and addressed caregiving risks (i.e., safety, emotional well-being, health and self-care, social support, and patient problem behaviors of the care recipient/caregiver skills). (43, 19)<br><br>Training and oversight of FCP staff by a member of the REACH II research team promoted fidelity to the intervention as well as specific training in the techniques used with caregivers.(71)<br><br>The intervention materials presented to caregivers in the REACH II clinical trial were extracted from the REACH II intervention manuals and reformatted into “A Caregiver's Notebook” (available from the corresponding author). (29)<br><br>The Pleasant Things For You section outlines activities that help the caregiver learn to find time to do things they enjoy; Healthy Living focuses on ways to stay organized and aware of the health of caregivers and their loved one; Understanding Your Feelings includes activities to help caregivers stay in control of their negative feelings stemming from the unpleasant behaviors that can occur when caring for their loved one; Skillful Communication includes tips to help caregivers communicate more effectively with their loved one. (29)<br><br>Program staff were trained to deliver intervention components using the training material available in the REACH II intervention manual. This included a formal training program followed by routine supervision from the first author (a REACH II principal investigator). (71)	5 - Train and educate stakeholders	43, 19, 71, 29
			The FCP targeted one large hospital (636 beds) and one large ambulatory internal medicine primary care clinic within the Scott & White system. Implementation of the project was facilitated by a partnership with the Central Texas AAA. (30)	6 - Support clinicians	30, 32, 59

			FCP staff developed a streamlined referral system allowing them to automatically refer enrolled caregivers to the Central Texas AAA for formal services such as access to respite care and counseling services. (30, 32)<br><br>Staff members were master's-trained counselors.(59)		
			The five REACH II intervention components, delivered through home visits and therapeutic phone calls, identified and addressed caregiving risks (i.e., safety, emotional well-being, health and self-care, social support, and patient problem behaviors of the care recipient/caregiver skills). (50)<br><br>The Pleasant Things For You section outlines activities that help the caregiver learn to find time to do things they enjoy; Healthy Living focuses on ways to stay organized and aware of the health of caregivers and their loved one; Understanding Your Feelings includes activities to help caregivers stay in control of their negative feelings stemming from the unpleasant behaviors that can occur when caring for their loved one; Skillful Communication includes tips to help caregivers communicate more effectively with their loved one. (39, 50)	7 - Engage consumers	50, 39
			Generous support from the Rosalynn Carter Institute Caregiver Program as well as supplemental support from Scott & White Healthcare allowed for the translation of the REACH II intervention materials into A Caregiver's Notebook, a format that was familiar to consumers of support services and one that could be widely disseminated. (1)	8 - Utilize financial strategies	1
			The five REACH II intervention components, delivered through home visits and therapeutic phone calls, identified and addressed caregiving risks (i.e., safety, emotional well-being, health and self-care, social support, and patient problem behaviors of the care recipient/caregiver skills).(13)<br><br>Caregivers' requests for in-person or phone therapeutic contacts were never denied. (13)	9 - Change infrastructure	13
Israeli NYUCI	Werner et al. (2020) / Israel	counselors (recruited prior to implementation)	The research team communicated on a regular basis with the project coordinator who maintained steady contact with the counselors and reminded them to complete the assessments as required. (6)<br><br>At the beginning of the study, a training course was given in Israel to prospective providers. (36)<br><br>Potential NYUCI counselors were recruited for the two-day training via an advertisement posted on several professional websites; 24 social service providers (88% female) were selected for the training from among 100 applicants, based on level of relevant education (in social work, psychology or related disciplines) and competence as reflected by having previous experience working with persons with dementia and their family caregivers. (57)<br><br>The course was conducted by the developer of the original intervention program and a bilingual (English/ Hebrew) NYU clinician with experience implementing and providing training for the intervention with the assistance of a bilingual (English/Hebrew) clinical social worker/project manager with a clinical psychology background from the Israeli Alzheimer’s Association. (24)	4 - Develop stakeholder interrelationships	6, 36, 57, 24
			At the beginning of the study, a training course was given in Israel to prospective providers. (71)<br><br>This was similar to translations of the NYUCI in the United States, where it was required that counselors had a degree in social work or a related profession and 5years of clinical experience (Mittelman & Bartels, 2014). However, it should be noted that in the translations, since there was no control over the sites, sometimes counselors were not required to be licensed clinicians. (71)	5 - Train and educate stakeholders	71, 29

			The NYUCI manual was translated into Hebrew following a translation and back-translation process. (29)		
			The Association's funds come from fees payed by its members, donations, and grants received from foundations and governmental sources. The implementation of the Israeli NYUCI was funded by a grant from the National Insurance Institute to the Alzheimer's Association, covering the salary of the coordinator, the reimbursement to the counselors, and the evaluation of the effectiveness of the intervention, which was conducted by researchers who were not members of the Israeli Alzheimer's Association. (34, 1)	8 - Utilize financial strategies	34, 1
			This was similar to translations of the NYUCI in the United States, where it was required that counselors had a degree in social work or a related profession and 5years of clinical experience (Mittelman & Bartels, 2014). However, it should be noted that in the translations, since there was no control over the sites, sometimes counselors were not required to be licensed clinicians. (22)	9 - Change infrastructure	22
Support interventions (n=5)					
MCSP	Mazurek et al. (2019) / Poland	well-trained researcher	The pilot MCSP was successfully implemented in two MCs in Wroclaw in Poland following a 12-month period of collaborative community engagement and preparatory work according to the Dutch stepwise implementation procedure. (61)	1 - Use evaluative and iterative strategies	61
			As far as carers are concerned, there are psycho- educational meetings and discussion groups to participate in. Both groups can avail themselves of social activities, a weekly consultation hour and regular "center meetings" during which all participants, staff, as well as volunteers can share their experiences. (33)	2- Provide interactive assistance	33
			The program is offered in accessible locations that facilitate social inclusiveness and community integration and promoting social participation. This makes them more attractive than institutional day care and makes it easier for people to use support from an early stage of the disease. (63, 51)<br><br>Examples of activities that have developed in the centers spontaneously include playing billiards and having a drink with visitors at the coffee bar, painting together, and interacting with other generational groups using the same community facilities. In addition, family carers participate in activities in the community center. (51)	3 - Adapt and tailor to context	63, 51
			The pilot MCSP was successfully implemented in two MCs in Wroclaw in Poland following a 12-month period of collaborative community engagement and preparatory work according to the Dutch stepwise implementation procedure. (35, 57, 38, 47, 17, 52)	4 - Develop stakeholder interrelationships	35, 57, 38, 47, 17, 52
			There is a social club organized for the people with dementia (3 times per week). In the club people in question can take part in recreational activities and psychomotor therapy. As far as carers are concerned, there are psycho- educational meetings and discussion groups to participate in. Both groups can avail themselves of social activities, a weekly consultation hour and regular "center meetings" during which all participants, staff, as well as volunteers can share their experiences. (43, 19, 55, 71)	5 - Train and educate stakeholders	43, 19, 55, 71
			The pilot MCSP was successfully implemented in two MCs in Wroclaw in Poland following a 12-month period of collaborative community engagement and preparatory work according to the Dutch stepwise implementation procedure. (37)<br><br>The program is offered in accessible locations that facilitate social inclusiveness and community integration and promoting social participation. This makes them more attractive than institutional day care and makes it easier for people to use support from an early stage of the disease.	7 - Engage consumers	37, 39
			The pilot MCSP was successfully implemented in two MCs in Wroclaw in Poland following a 12-month period of collaborative community engagement and preparatory work according to the Dutch stepwise implementation procedure. (34)	8 - Utilize financial strategies	34

			The coordination of care services at home is also supported by the staff. (13)<br><br>The program is offered in accessible locations that facilitate social inclusiveness and community integration and promoting social participation. (13)	9 - Change infrastructure	13
MCSP	Meiland et al. (2005) / The Netherlands	community centres and centres for the elderly) by a small and permanent team of professionals (a programme coordinator, an activity therapist and one nursing assistant	However, a number of phases can be distinguished in the implementation of meeting cen- tres, irrespective of local conditions: (a) a preparation phase which includes an exploration of the region, creating support for the meeting centres model, and organizing initiators, (b) an execution phase during which staff is recruited, the location is organized and pre- pared, collaboration with other care and welfare organization is given shape and financing is arranged, and (c) a continuation phase in which the meeting centre is structurally embedded and secured within regular care. Four centres were set up in this way during a devel- opment and evaluation study, two centres were supervised and set up by implementation experts in the context of this study, and the remaining seven centres were organized by initiators from the different regions. (23)	1 - Use evaluative and iterative strategies	23
			The small-scale, integrated and intensive set-up of the support close to home stimulates the development of a relationship with the staff that is based on trust, and makes it easier for the family carer to accept help and share the care with others. In addition, the selected locations stimulate social integration with other people in the neighbourhood. (63)	3 - Adapt and tailor to context	63
			The innovative aspect is that a varied and integrated support offer for people with dementia and their caregivers is offered at an easily accessible location (community centres and centres for the elderly) by a small and permanent team of professionals (a programme coordinator, an activity therapist and one nursing assistant). (35, 6)<br><br>This team maintains intensive contacts with other care and welfare organizations in the area for the execution of the support programme (based on a collaboration protocol). (6, 52, 24, 64)<br><br>Setting up a meeting centre in a region requires close collaboration between the existing care and welfare organizations, and it is therefore dependent on local circumstances. (47, 52)	4 - Develop stakeholder interrelationships	35, 6, 52, 24, 64, 47
			The support programme for the people with dementia consists of a social club (three days a week); informative meetings and ongoing discussion groups for the carers; a monthly meeting for all participants, a consulting hour, case management, and social activities for both groups. (19, 55, 43)	5 - Train and educate stakeholders	19, 55, 43
			This collaboration enables effective case management. The small-scale, integrated and intensive set-up of the support close to home stimulates the development of a relationship with the staff that is based on trust, and makes it easier for the family carer to accept help and share the care with others. In addition, the selected locations stimulate social integration with other people in the neighbourhood. (39)	7 - Engage consumers	39
			The innovative aspect is that a varied and integrated support offer for people with dementia and their caregivers is offered at an easily accessible location (community centres and centres for the elderly) by a small and permanent team of professionals (a programme coordinator, an activity therapist and one nursing assistant). (13)	9 - Change infrastructure	13
MEETINGDEM (MCSP)	van Mierlo et al. (2017) / The Netherlands	-	-	-	-
			Meeting Center Support Program (MCSP)		
	van Haeften-van Dijk et al. (2015) / The Netherlands	The members of the project group together with representatives of the cooperating organizations formed the initiative group and this group worked according to a step-by-step guide. Various workgroups,	For both, there is a consultation hour, social activities and a regular centre meeting where all the people involved (patients, family carers, volunteers and professionals) can express their wishes concerning changes in the programme (5)<br><br>An adaptive implementation strategy was used, which means that every day care centre implemented the Meeting Centres model by making it compatible with the specific context (target group, location, dementia care chain) of the day care centre and the region (4)<br><br>The project group monitored the transition process and discussed subjects of concern with the	1 - Use evaluative and iterative strategies	4, 5, 18, 56

		consisting of staff of the daycare centre and/or other employees of the initiating organization and representatives of the cooperating organizations, addressed different topics.	initiating organization, such as 'staff training' and 'the need for this type of day care in the region' and 'possible solutions for barriers to successful implementation'. (4, 5, 18, 56)<br><br>Various workgroups, consisting of staff of the day care centre and/or other employees of the initiating organization and representatives of the cooperating organizations, addressed different topics. One group defined the 'target group' of the new day care centre, others elaborated the topics 'support programme', 'location', 'finances', 'public relations' and the development of a 'cooperation agreement'. The results of the workgroups were discussed in the monthly initiative group meeting and adjusted if necessary. (5)		
			An adaptive implementation strategy was used, which means that every day care centre implemented the Meeting Centres model by making it compatible with the specific context (target group, location, dementia care chain) of the day care centre and the region (51)	3 - Adapt and tailor to context	51
			Six Dutch nursing home-based day care centres made the transition to CO day care according to the Meeting centres support programme (36, 35)<br><br>The project group consisted of the project leader of the initiating care organization, a manager, the (future) programme coordinator of the new day care centre, the transition supervisor from VU University medical centre and a consultant from an existing Meeting centre, both very experienced in starting up Meeting centres and working according to the Meeting centres model, and a researcher from VU University medical centre. (6, 64, 65)<br><br>Local care and welfare organizations were invited to an information meeting at the start of the projects and were asked to participate in the initiative group to prepare the transition. Examples of cooperating organizations were the local Alzheimer association, mental health organizations, general practitioners, home care organizations, case managers and the local carer support organization. (52, 35, 38)<br><br>The members of the project group together with representatives of the cooperating organizations formed the initiative group and this group worked according to a step-by-step guide. (64)<br><br>The joint efforts of the initiative group and project group resulted in a product description of the CO day care. A cooperation agreement was signed by all relevant collaborating organizations. (47)	4 - Develop stakeholder interrelationships	35, 36, 64, 65, 6, 52, 35, 38, 47
			The MCSP Model includes a social club for people with dementia with special activity programmes and psychosocial and emotion-oriented approaches, such as recreational, creative and cognitively stimulating activities and psychomotor therapy, and offers case management for the persons with dementia. The carers are offered informative meetings about dementia-related topics, ongoing discussion groups, and respite care (19)<br><br>The project group consisted of the project leader of the initiating care organization, a manager, the (future) programme coordinator of the new day care centre, the transition supervisor from VU University medical centre and a consultant from an existing Meeting centre, both very experienced in starting up Meeting centres and working according to the Meeting centres model, and a researcher from VU University medical centre. (73)<br><br>Local care and welfare organizations were invited to an information meeting at the start of the projects and were asked to participate in the initiative group to prepare the transition. Examples of cooperating organizations were the local Alzheimer association, mental health organizations, general practitioners, home care organizations, case managers and the local carer support organization. (20)<br><br>Various workgroups, consisting of staff of the day care centre and/or other employees of the initiating organization and representatives of the cooperating organizations, addressed different topics. One group defined the 'target group' of the new day care centre, others	5 - Train and educate stakeholders	73, 19, 20, 71

			elaborated the topics 'support programme', 'location', 'finances', 'public relations' and the development of a 'cooperation agreement'. The results of the workgroups were discussed in the monthly initiative group meeting and adjusted if necessary. (20)<br><br>Staff of each day centre received a one to four-day training plus five refresher meetings on working according to the adaptation-coping model (a cornerstone of the Meeting centres model) and implementing psychomotor therapy. Also, training and coaching-on-the job were provided by the consultant. (19, 71)		
			The project group consisted of the project leader of the initiating care organization, a manager, the (future) programme coordinator of the new day care centre, the transition supervisor from VU University medical centre and a consultant from an existing Meeting centre, both very experienced in starting up Meeting centres and working according to the Meeting centres model, and a researcher from VU University medical centre. (59)	6 - Support clinicians	59
DemenTalent	van Rijn et al. (2019) / The Netherlands	Project leaders at Meeting Centers	making appointments with the volunteer locations about regular contact with the Meeting Centers to monitor if the person with dementia and the volunteer location were both happy with the work done and with the interaction with the person with dementia and the professionals of the Meeting Center. (5, 27)<br><br>recruiting potential locations based on the interests and talents of the persons with dementia (4)	1 - Use evaluative and iterative strategies	5, 27, 4
			In a 3-hour information meeting, the appointed project leaders of DemenTalent and other representative of all participating centers were informed about the research, DemenTalent and the implementation procedure. (35, 57, 6)<br><br>informing the personnel on the volunteer locations on how to deal with people with dementia in general and with the individual person appointed for the volunteer task;(35)<br><br>informing the network of care and welfare referrers (52, 6)<br><br>recruiting a project leader with dementia as ambassador of the project in the region; (35)<br><br>With each Meeting Center appointments were made about individual coaching of the centers personnel during the preparation and starting phases of the implementation of DemenTalent by an external coaching company. (52)	4 - Develop stakeholder interrelationships	35, 57, 6, 52
			With each Meeting Center appointments were made about individual coaching of the centers personnel during the preparation and starting phases of the implementation of DemenTalent by an external coaching company. (71)	5 - Train and educate stakeholders	71
			recruiting volunteers with dementia, exploring their interests and talents, and matching the volunteers to possible locations for volunteer work; (39)	7 - Engage consumers	39

Table 7. Implementation Outcomes

Author(s), Year / Country	Implementation outcomes	Details
eHealth (n=15)		
Banbury et al. (2019) / Australia	Acceptability	Most were very positive about VC and its potential use in the future; those who experienced difficulties, there was a feeling that the technology was not reliable and needs improvement<br><br>**Caregiver outcomes measured using the UCLA Loneliness Scale (UCLS-6) and three sub-scales from the e-Health Literacy Questionnaire**
	Appropriateness	Participants with no prior experience of using VC often reported feeling nervous about its use. Following the completion of the programme, most were very positive about VC and its potential use in the future:
	Penetration	Recruitment activities were time consuming. Meeting days and times which participants indicated as suitable when signing up were not necessarily still available when organizing the meetings. Recruitment procedures were changed to enable verbal consent over the phone or traditional mail when returning the consent form when email was not possible.
	Sustainability	To date, eight groups have self-organized to meet, including a group that codesigned the content of the programme which has been meeting monthly for more than 12 months.
Baruah et al. (2020) / India	Acceptability	Technical challenges such as slow loading webpages and delay in response time were considered off putting. Few other functionalities of the online program such as text heavy format, not striking a balance between texts, pictures and videos, and multilevel navigation were considered to be discouraging, especially for the elderly caregivers.
	Appropriateness	
	Penetration	Family caregivers were recruited from the Geriatric Clinic & Services, NIMHANS, and NGOs involved in dementia care like the Alzheimer's and Related Disorder's Society of India (ARDSI), Bengaluru Chapter.
Boots et al. (2017) / The Netherlands	Acceptability	Most participants were satisfied with the content because it suited their current concerns. (…) The personalized assignments and challenges were appreciated.
	Appropriateness	The program was rated as a useful addition for family caregivers and for the coach as a professional caregiver.<br><br>Some participants (caregivers) struggled with the primary focus on the caregiver because they felt the care recipient should change.<br><br>Age gap between participant groups; content should be more age appropriate<br><br>They [younger carers] could not identify with the older population in the examples because they were still employed and dealt with other issues in daily life, such as (young) children living at home.
	Feasibility	Coaches found it adequately feasible in daily practice and as fairly easy to integrate into their work related activities.
	Penetration	The caregivers were invited to participate by the clinician who treated their family member (n=122), were informed about the program's existence by the Dutch Alzheimer Association n=26),or knew caregivers or family members already involved in the program (n=4). Others (n=11) requested information based on editorials in health magazines, local newspapers, and information stands in the southern parts of the Netherlands.<br><br>The Dutch Alzheimer Association disseminated information about the program via the following: (1) monthly meeting spots for people with dementia and their caregivers, (2) newsletters, and(3) their website and social media platforms, including Facebook and Twitter.
	Sustainability	Embedding within agency by revising professional roles/retraining staff(...) out of the interested organizations, 40% (4/10) organizations choose to implement "PiB" and train staff members (psychologists or psychiatric nurses) to act as personal coaches.<br><br>Registration; insurance compensation; integrated online support<br>The facilitating aspects included registration of the caregiver independent from the person with dementia, insurance compensation, and integration of online support in already provided caregiver support.<br><br>Designating intervention as regular care<br>Training and selfstudy were considered substantial personal time investments. The coaches suggested training all staff members as coaches and designating the program as regular care to facilitate implementation. "
Dam et al. (2019) / the Netherlands	Acceptability	Participants reported that the structure and layout of Inlife were clear. The circular structure of Inlife was valued for the privacy and autonomy, since carers could decide for themselves what was shared with whom. This setup increased feelings of control.<br>(+) userfriendly interface (clear symbols, colors, circles)<br>() high number of login, need for upload function, increase text layout options, include chat box and videoconferencing function"
	Adoption	(User adoption) Some network members (end-users) were reluctant to post reactions on Inlife, which occasionally caused disappointment and frustration and, in some cases, prevented Inlife usage.

	Appropriateness	The goal, content and number of functionalities were considered appropriate and offered the possibilities that the participants expected. Inlife is a promising online tool to ease the organizational burden of care, serving as a convenient platform for central care organization
	Penetration	Data was collected on the recruitment method through which they were reached through flyers (38 recruited from 500flyers distributed), Alzheimer Netherlands Facebook, website ads and online newsletters (240recruited from 950,000 cumulative page views), as well as ads in local parish newsletters (19 recruited from 20,528 parish newsletters distributed), community services (56 recruited from 101 attendees of local Alzheimer Café's or caregiver meetings), casemanager referrals(25 recruited from 25 referrals), caregivers known to the research group through previous studies (82 recruited from 150) and communication with known relatives or acquaintances (n= 15).
	Sustainability	overall network size and the number of reactions or posts by network members determined continued use of Inlife.<br><br>Routinization: the presence of other widespread online tools (e.g., WhatsApp) influenced involvement with Inlife. People were sometimes inclined to use WhatsApp instead of Inlife because it was more routinely used in their daily practice.
Frame et al. (2013) / USA	Adoption	To date, we have successfully trained all 25 members of our clinical team at Wishard Health in the use of the software.
	Appropriateness	As a result, the eMRABC records all our patient encounters, allowing for **more efficient tracking of patient interactions and permitting us to monitor progress in achieving patient and caregiver goals.**<br><br>Clinical providers are better equipped to adhere to a patient's visit schedule and to respond quickly (within 4872 hours) to caregiver stress and acute care utilization events.
	Sustainability	(After implementation) Clinical providers are better equipped to adhere to a patient's visit schedule and to respond quickly (within 4872 hours) to caregiver stress and acute care utilization events.
Glueckauf and Loomis (2003) / USA	Appropriateness	The only products available for purchase at the time were Real Networks Producer and Real Presenter. They both offered live and prerecorded video and audio streaming, as well as the option of delivering presentation slides to caregivers. However, neither Real Networks Producer nor Real Presenter supported textbased chat, a required feature of our interactive classes. In addition to the two Real applications, we examined solutions from other corporations that claimed they had a product that would meet our requirements. (...) User interface required bigger font and less text
	Penetration	we hired the Wilson Agency located in Jacksonville, Florida to serve as our marketing consultants. Following their advice, we convened a smallworking group conference consisting of statewide leaders from DOEA, the state's Area Agencies on Aging, Florida chapters of the Alzheimer's Association, senior service organizations, and dementia caregivers. The working group unanimously agreed that the best way to inform caregivers about AlzOnline was to encourage grassroots senior service organizations (i.e., those agencies who influence and support dementia caregivers) to refer their clients to our website.
	Sustainability	Contracting external companies for tools: However, neither Real Networks Producer nor Real Presenter supported textbased chat, a required feature of our interactive classes. In addition to the two Real applications, we examined solutions from other corporations that claimed they had a product that would meet our requirements.<br><br>Contracting marketing agencies for dissemination: Other key strategies proposed in the Wilson's marketing plan were: (a) creating a public awareness media campaign that specifically focused on print, radio, and television outlets, (b) positioning www.AlzOnline.net supervisory staff as experts in telecommunications and in dementia caregiver education, and (c) promoting speaking engagements in statewide caregiver conferences, seminars, seniors expos and local senior service organizations.
Levinson et al. (2020) / Canada	Acceptability	Our findings showed that individuals who had taken the time to review iGeriCare were more positively predisposed toward it and placed a higher value on the intervention than those who were less familiar with it
	Adoption	**Need for alignment with physicians to implement innovation:** Many primary implementation agent (i.e. physicians) in independent practice might not strongly identify with their affiliated health care organizations (e.g., hospitals), highlighting the added importance of individual characteristics as a construct in an organization's potential implementation that may rely on physicians.
	Appropriateness	Most participants saw the iGeriCare intervention as a good fit with their existing workflows. Conversely, a few participants expressed concerns about its implementation within their practice settings and existing workflows.
	Penetration	Issues with engagement and reach A multimodal engagement strategy is required, targeting organizations, clinicians, trainees, and family caregivers. The costs associated with ongoing promotion and engagement (whether they be marketing costs, personnel, or the true costs of time for champions) are not trivial and may prove to be an important barrier with regard to the implementation
	Sustainability	Many participants stated that iGeriCare was presently being used or could easily be implemented because of its compatibility and relative priority.
Meichsner et al. (2018) / Germany	Acceptability	46.7% of participants evaluated the setting as 'very good', 33% 'good', 13.3% ' moderate'; one participant disapproved of the internet setting.<br><br>**(Outcome measurement) Satisfaction measured with Client Satisfaction Questionnaire (CSQ-8, German: ZUF- 8; Attkisson & Zwick, 1982), and contains eight items (e.g., "How would you rate the quality of service you received?") that are rated on a 4-point Likert scale**
	Feasibility	Low dropout rate [16.22% attrition rate] mean intervention duration was 11.4 weeks (SD = 3.25; range 8–20 weeks). Four therapies (26.67%) were completed within the scheduled 8 weeks, another three (20%) were completed within 10 weeks. The remaining eight therapies lasted between 11 and 20 weeks due to unforeseen circumstances such as hospitalization.<br><br>Time needed for breaks (inconsistent time) caregivers also used the option to break up their session: 37.30% of participants reported to have taken a break during writing that ranged from a few minutes until 1.6 hr. Two-thirds of messages (66.67%) were sent outside the routine working hours (9 a.m. to 5 p.m.) and participants frequently wrote their messages between 9 p.m. and 1 a.m. (31.72% of messages were sent during that time).

Mitchell et al. (2017) published in (2020) / USA	Acceptability	Only a little over half (57.1%) of participants reported that they were satisfied with RAM, and only 59.2% felt it met their care recipient's needs, emphasizing that some users do not find RAM satisfactory.<br><br>**(Outcome measurement) Caregiver outcomes were measured using the RAM system review checklist, a 21-item, close- ended checklist. The senior author adapted checklists administered in prior research to assess perceptions of acceptability and utility of the RAM system for persons with ADRD.**
	Appropriateness	Some users did not agree on appropriateness "The appeal of the RAM system depended critically on the fit between users' needs and the services offered. Fourteen participants suggested that RAM did not fit their caregiving situation. (...) Reasons for a mismatch were as follows: (a) the stage of the care recipient's disease progression or (b) if the caregiver lived with the care recipient.
	Penetration	Participants were recruited for the parent study via the University of Minnesota Caregiver Registry, advertisements, and community outreach
	Sustainability	Some who had a positive experience with RAM mentioned a desire to keep the system after the conclusion of the study. Some even felt anxiety over the idea of giving up the system because it had become an integral part of their caregiving routine.
PaganOrtiz et al. (2014) / USA	Acceptability<br>Appropriateness	sixty four percent of participants responded that they would return to the website regularly to learn more about the disease. Overall results from the formative evaluation showed that the website was easy to navigate and user friendly. Most participants suggested it to be a valuable resource for Hispanic caregivers and would recommend it to others
	Penetration	Participants from Puerto Rico and Massachusetts were recruited via outreach strategies that included: letters, press releases, flyers, as well as phone calls to agencies in contact with caregivers. In Mexico, participants were recruited from a pool of caregivers who received social support services at a neurology teaching hospital.<br><br>use of social media Maintaining a Spanishlanguage website presents a unique challenge, as there are many Spanishspeaking countries whose population we want to reach, but for one reason or another may not have easy access to our site, mainly due to barriers with international search engines. The constant creation of new content to keep users coming back to the website can also be a challenge, which was successfully addressed by encouraging usergenerated content.<br><br>Once Cuidate Cuidador was established on Facebook, various strategies were used to reach out to caregivers and organizations that were already registered on the site. Searches were made for particular organizations, such as different branches of Alzheimer's Association, as well as for individuals who had listed particular interests, such as "caregiving" or "Alzheimer's". By purchasing ad space on Facebook, we were also able to place ads, and redirect those who clicked on them either back to the Facebook page or website.
Pot et al. (2015) / The Netherlands	Acceptability<br>Appropriateness	Reasons for quitting include poor fit for user needs (24.1%), care receiver related issues (27.8%), caregiver overburden (24.1%) or needed other help (24.1%)<br><br>Completers Caregivers' evaluation of the intervention seemed satisfactory for those who completed the intervention (N = 66; 44.2%). They evaluated the lessons and the feedback they received from the coach at the top end of the scale.<br><br>**(Outcome measurement) Outcomes (comprehensibility and usefulness of the lesson) were measured on a 5-point scale ranging from (1) "not easy at all to understand" to (5) "very easy" and from (1) "not useful at all" to (5) "very useful". Before the start of a new lesson, caregivers were asked to score the comprehensibility and usefulness of the feedback from the coach.**
	Penetration	Family caregivers of people with dementia were recruited via the website "Mastery over Dementia", the monthly digital newsletter of the Alzheimer's Society, leaflets at Alzheimer Cafe meetings (meetings for people with dementia, their caregivers and other interested people) and information letters to memory clinics and other relevant care institutes.
Teles et al. (2020) / Portugal	Appropriateness	Most of the 13 features rated by the participants were overall considered important or very important. Most caregivers (94.3%; n = 83) reported using the Internet daily, confirming that a digitally literate sample was successfully recruited.
	Penetration	Multiple advertising channels were used to recruit participants: (a) the online channels of the national Alzheimer's association; (b) online channels of communitybased projects with informal dementia caregivers; and (c) Facebook pages on the topic of caring for someone with dementia
Xiao et al. (2020) / Australia	Acceptability<br>Appropriateness	Informal caregivers perceived iSupport as an opportunity to provide an online onestop shop to meet their education needs and their needs to manage care services.<br><br>Engaging the family as a unit in **iSupport may be particularly suitable for older female informal caregivers who also often have low health literacy and computer literacy**. This strategy may also enhance support from family members in dementia care, especially in the late stage of dementia.
van Knippenberg et al. (2017) / The Netherlands	Acceptability<br>Appropriateness	Feedback provided by the coach at the end of each session was seen as a useful addition and was considered easy to understand participants considered the intervention program 'Partner in Sight' acceptable and feasible. Participants were satisfied with the respect to the content and structure of the program. The ESM derived feedback was considered supportive and increased participants'awareness of their feelings and behavior. However, there was a large variance in the extent to which participants tried to apply the feedback into their daily lives."<br><br>**(Outcome measurement) Outcomes (acceptability of content) measured during feedback sessions and evaluated using a 7-point Likert scale.**
	Penetration	"Informal caregivers (N= 295) were recruited from memory clinics (Maastricht University Medical Center +, ZuyderlandMedical Center), ambulatory mental health care institutions(VirenzeRIAGG Maastricht, Lionarons GGZ), dementia daycare centers (Sevagram, NOVIzorg, Orbis Glana, Proteion, care farm Ransdalerveld), caregiver support services in the southern Netherlands, and via dementia case managers ('Hulp bijDementie') and the Dutch Alzheimer Association.<br><br>The Dutch Alzheimer Association promoted the intervention program via (1) Alzheimer Cafes for people with dementia and their caregivers, (2) a digital newsletter, and (3) their website. Almost all participating caregivers were recruited by health care professionals; only seven caregivers were informed about the intervention by the Dutch Alzheimer Association or in another way.

		Main recruitment barriers in our study included no need for additional support and considering the intervention as too timeconsuming or burdensome. "
Williams et al. (2020) / USA	Acceptability Appropriateness	The majority of FamTechCare caregivers reported the VMU was easy to set up and use (75.6%) and found it easy to transfer recordings to the expert team (75.6%). Having the VMU in their home was acceptable (85.4%) for the majority of caregivers, and the VMU did not intrude on the majority of caregivers 'privacy (63.4%). Because many caregivers used the manual iPad button to initiate recording, nearly half of the responses relating to use of the remote control were missing.<br><br>**(Outcome measurement) Satisfaction with video monitoring was measured at the end of the 3-month trial with a 13-item survey. A 5-point Likert scale (1 = strongly disagree to 5 = strongly agree) was used to assess ease of use and perceived satisfaction with the VMU and telehealth feedback.**
	Adoption	"However, another expert asserted that it cannot be streamlined into clinical practice because "providers don't have time and often don't care about dementia...it's unrealistic to think providers would be interested or have time for videos."
	Feasibility	One expert noted "Currently FamTechCare is doable. It's not too much work, but the question is who will pay for and manage it. "However, another expert asserted that it cannot be streamlined into clinical practice because "providers don't have time and often don't care about dementia...it's unrealistic to think providers would be interested or have time for videos. "This expert later described FamTechCare as more feasible outside the traditional healthcare system and more as a communitybased resource that does not operate within the healthcare system.<br><br>Due to these concerns about feasibility, some experts suggested that FamTechCare should be tested with a communitybased partnership. One expert noted, "It's not enough to say call[community resource], we need to help coordinate and take the work off the caregiver to organize and find resources."Experts disagreed about how the intervention should interface with the person living with dementia's care team.
Respite Care (n=5)		
Beisecker et al. (1996) / USA	Acceptability	Users ADC benefited both the PwD and the caregiver; nonuser found ADC benefited the caregiver only<br>PwD unhappiness, safety, physical health, functional levels and behavior were viewed as barriers to ADC use
	Penetration	Users and nonusers of ADC participated in the study; researchers maintained confidentiality by not obtaining list of clients from service providers and Alzheimer's disease networks.<br><br>Flyers announcing the study and the need for volunteer subjects were distributed in the Alzheimer's Association newsletter and the Kansas Department on Aging newsletter as well as AD support group meetings, medical clinics, adult day care centers and physicians' offices.
Brandao et al. (2016) / Portugal	Acceptability	61.4% of respondents expressed possibility of use. Most common reasons for becoming a user were the care receiver's progressive dependency level (38.5 percent) and his or her presence of health problems (23.1 percent). Most users (90.9 percent) considered the possibility of using these services again, even though their costs were perceived as high (66.7 percent). Overall, they were satisfied with the services
	Penetration	Caregivers were recruited through local health and social services agencies, community flyers, the project's newsletters, and through word of mouth from February 2010 through April 2013
Gaugler (2014) / USA	Acceptability Appropriateness	Philosophy and programming of ADS were positive compared to nursing homes (including staff, care routinization, scheduling)<br><br>Activities involved in program benefited clients through socialization, independence and stimulation
Gitlin et al. (2019) / USA	Fidelity	three areas to consider for enhancing, monitoring, and measuring fidelity: treatment delivery (e.g., program is delivered by interventionists as it is intended), treatment receipt (e.g., program isreceived by caregivers); and enactment (caregivers use treatment strategies)
	Implementation Cost	The economic analysis will consist of measuring whether ADS Plus(costs of ADS Plus = ADS plus program delivery costs + person with dementia's direct costs + primary caregivers direct costs + primary caregivers lost productivity + primary caregivers time spent providing informal care) results in a cost offset<br><br>Direct costs encompass healthcare service use (inpatient, outpatient, emergency visits, medications), community service use (e.g., meals on wheels, social worker visits, adult day services,formal homemaker/housecleaning services, formal caregiver/home aides), and longterm care use (e.g. admission or respite stays in nursing home, rehabilitation, assisted living, group homes). We will also measure caregiver healthcare service and indirect costs in the form of caregiver productivity losses.
Roberts and Struckmeyer (2017) / USA	Acceptability Appropriateness	While some study participants used respite programming frequently, others had only used respite once or twice. For some, just knowing that help was available should they need it, allowed some relief. This is due to the push and pull of emotions for caregivers, as often there is a sense of guilt in asking for help, and the fact that the programs are available can be seen as a validation of the larger need.
	Implementation Cost	While many receive vouchers for the day care, others are fighting for funding while they continue to selfpay. Mary discusses how her fight for funding adds to the burden that she is feeling with her husband's early onset Alzheimer disease: "We cannot use Adult Day Care much because of finances. The VA is helping some but I don't know how long that will last and I am still fighting them for money that is due." If full funding is not made available, Mary will likely lose her opportunity for the respite that she currently needs and uses.
	Sustainability	Most state voucher programs provide services in vouchers of $200 to $400 once every 3 months, based on available funding. Caregivers can hire a person of their choice as the respite provider, as long as the person is 18 years or older, has a social security card, and lives outside the caregiver and care recipient's home. Respite services can be provided in the caregiver's home or someone else<br><br>Study participants were often funding outside help on their own prior to finding out about vouchers, and while the opportunity to get financial help with the services was seen as a benefit, that

		quickly became complicated and timeconsuming for many. **Vouchers are released quarterly, and even if caregivers get their paperwork in on time, there is a chance that the program will already have run out of funds due to high demand and small quarterly state allocations. Despite these holdups, family caregivers appreciate the opportunities that vouchers provide.** 's home.
Psychoeducation (n=12)		
Chang et al. (2010) / Australia	Acceptability Appropriateness	The descriptive statistics indicated that most of the carers found the booklet to be helpful (97%) and that the booklet contained sections that were useful (96%)<br><br>**(Outcome measurement) Caregiver responses were measured using a booklet evaluation questionnaire (Likert scale)**<br><br>More than half of the caregivers reported not becoming anxious while reading the booklet, almost twothirds did not find it overwhelming to read (69%), and only 11% thought the booklet was too confronting (dying process, the time of death, and the physical symptoms associated with endstage dementia, together with the need for future decisions around healthcare needs).
	Penetration	A total of 672 booklet packs were sent to 15 Statefunded dementia advisory services (DASs) (n = 129 booklets) and 48 Residential agedcare facilities (RACFs) (n = 543 booklets) for distribution. Of these, a total of 233 (33%) family caregivers completed and returned the booklet questionnaire evaluation.
Foley et al. (2020) / UK	Acceptability Appropriateness	Written feedback revealed themes of appreciating having their difficulties recognised, enjoying being able to share concerns, improved confidence in their ability to care and feeling more prepared for the future<br><br>**(Outcome measurement) Caregiver outcomes measured using Adult Carer Quality of Life Questionnaire (AC-QoL) Authors measured depression and anxiety using the Hospital Anxiety and Depression Scale (HADS), which has previously been used to measure emotional distress in dementia carers.**
	Feasibility	When we evaluated agreement to the therapy, attendance at sessions and written feedback, our findings suggested that our modified version of START, adapted for LBD, was both feasible and acceptable to our LBD carers.
Sommerland et al. (2014) / UK	Acceptability Appropriateness	17 of the 75 participants told us that they valued theinteraction with the therapist for varied reasons. Somewere grateful for the opportunity to share their concernswith a professional; others appreciated the personalattributes of their therapist, while yet others noted theempathetic approach of the therapist and the validationof their own feelings<br><br>Advice on coping with behaviour and communication was cited by 11/75 participants as welcome and was noted by some to have reduced their own distress
	Sustainability	The relaxation CDs were most commonly cited as being useful duringthe period of therapy and beyond, and **22/75 participants told us that they continued to use these and thetaught relaxation techniques**<br><br>**50 of the 75 participants of those who responded to the questionnaire said that they had continued to use the intervention since the end of thesessions**<br><br>Reasons for discontinuation Feeling too busy or tired to continue to engage withthe therapy was a frequently cited reason for not continuing to use the intervention
Griffiths et al. (2015) / USA	Acceptability Appropriateness	The majority of caregivers (all but the one neutral) "agreed" or "strongly agreed" that the program provided useful strategies and the content was personally meaningful to them and their situation.<br><br>**(Outcome measurement) At program's end, caregivers were asked to respond to evaluative statements about the program, such as "I believe I am more knowledgeable as a caregiver after participation in this program." The response scale ranged from 1 (strongly disagree) to 5 (strongly agree). Similarly scaled items elicited caregivers' perceptions of the utility of pro- gram overall and for the major components of the program. Caregivers were also asked to assign a quality rating for the program overall and for each of the individual components (manual, videos, group classes, and self-care sessions) using a scale of 0 (low quality) to 10 (high quality).**
	Fidelity	**Recruit original developers of SCP intervention**<br>we recruited six experts well versed in SCP (including two of the original authors) to assess the fidelity of TeleSavvy to the original Savvy Caregiver program. We prepared a packet of materials detailing the TeleSavvy curriculum and delivery strategy, and we sent links to several of the daily video modules to allow participants to see what caregivers would be experiencing in the program. The fidelity evaluation asked the experts to compare TeleSavvy with the original across 10 dimensions.
	Penetration	Participants in the VA TeleSavvy implementation were referred from the Atlanta VA Medical Center (AVAMC) dementia committee, the Geriatrics Research Education and Clinical Center, and by social workers and other providers in Geriatrics and Extended Care from the AVAMC and surrounding Community Based Outpatient Clinics.
	Sustainability	iPads were on loan to participants. Within 2 weeks after program completion, a project coordinator returned to participants' homes to collect the iPads and conduct followup interviews. (MAY AFFECT LONGTERM SUSTAINMENT OF INTERVENTION)<br><br>The project was conducted in a clinical implementation/quality improvement rather than a research framework and implemented by staff in the research service line rather than clinical (social work, mental health, or geriatrics) which might have led to sustainment of the program.
Kovaleva et al. (2017) / USA	Acceptability Appropriateness	1) Sense of connectedness despite virtual setting: participants understood it as the sense of rapport and satisfactory communication where topics that are pertinent to their situations are discussed and caregivers are being listened to. When asked whether they felt connected to others, none mentioned that this was a virtual connection<br>convenience of distance participation

		2) Poor user interface/coordination Participants suggested that the manual be laid out more clearly (e.g., include a table of contents and a glossary) and be more precisely coordinated with the videos, videoconference "lectures," and "homework" assignments. For many who attempted to complete all assignments, the lack of clear coordination was frustrating"
	Penetration	Caregivers were recruited through a variety of means, including ClinicalTrials.gov, TrialMatch, online and hard copy advertisements, referrals from health care professionals, and word of mouth
	Sustainability	Caregivers could not access the videos after TeleSavvy conclusion [Participants also asked for extra manuals to share with their family members and hard copies of PowerPoint slides presented during videoconferences.]
Judge et al. (2010) / USA	Acceptability Appropriateness	CGs indicated that the sessions and overall program were well equipped to address the challenges they faced and their care partners. CGs believed that the information and strategies provided in the sessions and overall were very useful, and the session materials were rated as highly useful and understandable. Additionally, CGs rated the discussions that took place in each session as very helpful and indicated they would highly recommend the intervention program to other caregivers and individuals with memory loss<br><br>**(Outcome measurement) Measures (Likert scale) used to assess the acceptability and feasibility of the ANSWERS intervention included (a) the number of dyads who successfully completed the program, (b) CGs' and CRs' ratings of the session content and process, and (c) intervention specialists' ratings of the sessions.**
	Fidelity	To ensure fidelity to the protocols, intervention specialists received supervision and feedback when initially working with dyads. Ongoing monitoring of ANSWERS was accomplished through biweekly conference calls, which allowed for discussion of cases and review of protocols, including length and content of sessions, modifying techniques, and working with dyads.
Lykens et al. (2014) / US	Acceptability Appropriateness	REACH II intervention hasthe potential to improve the quality of life of Alzheimer's caregivers over an extended period beyond the prepost period we observed. The Caregiver Burden domain which also demonstrated significant improvement in this study measures caregiver feelings of stress and also their good feelings associated with providing assistance to the Alzheimer's patient.
	Penetration	Referral sources to the program included a 24/7 telephone helpline, support groups, case managers, the Aging & Disabilities Resource Center (ARDC), partnering agencies, and home health agencies were utilized to recruit program participants.
	Sustainability	<u>Implemented in real community context and funded through sustainable funding mechanisms however, scaling up of programs remains challenging with limited community resources.</u><br><br>**Through the United Way of Tarrant County support for a smallscale evaluation of multiple programs funded as part of their Healthy Aging and Independent Living initiativ**e, we were able to establish that the REACH II intervention could be successfully implemented in a community setting with outcomes comparable to the original REACH II clinically based study. These findings support a wider scale implementation of the REACH Intervention in community settings to help address the growing demands on family and community caregivers of Alzheimer's patients in the coming decades as the U.S. population continues to age and acquire this condition.
MartindaleAdam et al. (2017) / USA	Acceptability Appropriateness	four session REACH model caregivers experience significant decreases in burden, depression, anxiety, caregiving frustrations, and stress symptoms (feeling overwhelmed, feeling like crying, being frustrated as a result of caregiving, being lonely).For patient care, caregivers report 1.2 fewer troubling patient behaviors and a decrease in safety concerns, both of which are significant findings. Concomitant with these findings, caregivers report a significant decrease of 1.6 h per day on duty
	Adoption	the organization determines if the new evidencebased practice is needed, assesses the potential match between the organization and the practice, and determines whether or not the practice can be implemented.<br><br>A partnership of IHS, primarily the Division of Nursing Services, the Administration on Community Living (ACL) through the Administration on Aging's Native American Caregiver Support Services Program, and the University of Tennessee Health Science Center (UTHSC), was formed to implement REACH for caregivers of persons with dementia regardless of veteran status. UTHSC was involved as the VA Caregiver Center did not have capacity to train nonVA staff. Staff included public health nurses and community health representatives (Tribal and IHS) and Tribal aging network (senior center) staff funded through Title VI of the Older Americans Act (ACL/AoA).<br><br>For example, staff training was designed to be carried out using webinar technology. Although this model is still being used, states and Tribes have requested large, inperson trainings followed by onsite certifications. These facetoface trainings allow staff to interact with the Caregiver Center Coordinator in amore personal way and to interact and share with each other about strategies to deliver the program.<br><br>The initial process of implementation is slow and dependent on agency leadership support, publicity about the program, and word of mouth. Staff are taking increasing ownership, and several strategies have been implemented to increase participation by staff and by caregivers. For staff, the IHS Clinical Support Center made it possible for training to be accredited for nursing continuing education credits. The two federal agencies, IHS and ACL/AoA, have shared facetoface training venues and costs to increase opportunities for training.
	Implementation Cost	With funding for3 years from the private Rx Foundation, the process of tailoring the intervention for AI/AN caregivers begun in February 2015.
	Penetration	In addition, patient referral processes and networks must be developed, and initial data on the intervention process and outcomes are gathered. (...) The initial process of implementation is slow and dependent on agency leadership support, publicity about the program, and word of mouth. Staff are taking increasing ownership, and several strategies have been implemented to increase participation by staff and by caregivers. For staff, the IHS Clinical Support Center made it possible for training to be accredited for nursing continuing education credits. The two federal agencies, IHS and ACL/AoA, have shared facetoface training venues and costs to increase opportunities for training.<br><br>**Use of a marketing committee** The Marketing/Strategy Committee initially identified two areas of focus. The first is to assist REACH Coaches in recruitment using strategies and language that will resonate with AI/AN dementia caregivers. For example, a REACH Coach participated in a local radio program providing information on Alzheimer's and dementia and highlighted REACH as a community resource available for family caregivers. Coaches have requested a publicity brochure for distribution throughout the community. The Marketing/Strategy committee is working on developing a

		brochure template that can be customized to a community. This brochure could be used to encourage primary care providers to make referrals of caregivers who are in need of help, encourage IHS, Tribal, and AoA/ACLstaff to become Coaches, and encourage local agencies and services that support elders or family caregivers to include REACH in their programming. However, for other areas without wellfrequented community gathering places, other ways to publicize, such as radio and television PSAs and social media, may be a better strategy to reach caregivers.
	Sustainability	All implementation activities are carried outby local staff already working in the community. (...) Staff included public health nurses and community health representatives (Tribal and IHS) and Tribal aging network (senior center) staff funded through Title VI of the Older Americans Act (ACL/AoA).<br><br>The initial process of implementation is slow and dependent on agency leadership support, publicity about the program, and word of mouth. Staff are taking increasing ownership, and several strategies have been implemented to increase participation by staff and by caregivers. For staff, the IHS Clinical Support Center made it possible for training to be accredited for nursing continuing education credits. The two federal agencies, IHS and ACL/AoA, have shared facetoface training venues and costs to increase opportunities for training.
McCurry et al. (2015) / USA	Acceptability Appropriateness	**(Outcome measurement) Ninety two percent of caregivers who returned the survey felt the program had been very or somewhat helpful, and 80% said they would definitely or probably continue to use STARC skills.**
	Adoption	STARC consultants are critical to the success and effectiveness of the program. Oregon chose to implement STARC in part because existing case managers could be trained to offer it, but consultants must learn to "wear a different hat" during their STARC sessions, focusing less on direct advicegiving and more on training STARC concepts and empowering caregiver participants to brainstorm their own ideas
	Feasibility	The Oregon project intentionally did not allow consultants to provide the intervention to individuals in their own case management portfolios, but in many locations this separation of roles would be neither feasible nor desirable
	Fidelity	Because one of the aims of the OTS was to examine the feasibility of training AAA case managers to administer STARC, after certification two randomly selected session recordings from each case were reviewed by UW trainers for fidelity to the treatment protocol. This was in contrast to the original RCT, where clinical supervisors reviewed every audiorecorded STARC session. OTC consultants were encouraged to contact trainers with any questions or concerns that they had about STARC concepts or how best to present the material to clients with particularly challenging behaviors or situations. Once certified, consultant fidelity was maintained through (a) participation in periodic group conference calls with UW investigators, (b) ongoing supervision with their individual regional coordinators to address clinical issues or logistical difficulties, and (c) attendance at subsequent training workshops, both as a way to refresh their own knowledge about the program and to enhance the group training by sharing their experiences with new consultants.<br><br>Careful attention to treatment fidelity during the certification process has ensured that the cadre of Oregon STARC consultants is well trained according to standards developed by the program originators. However, the translation of any evidencebased program into the community is not without challenges. Some of the lessons learned as we engaged in this expansion will be helpful for future sites looking to implement such programs.
	Penetration	"Printed brochures were developed in the first year of the grant. Existing AAA casemanaged clients were reviewed to identify potential participants. AAA regional coordinators also distributed brochures at community presentations to medical providers, elder law attorneys, adult day and other dementia care programs, faithbased organizations, senior meal sites, and libraries. Lastly, information about STARC was provided to the Alzheimer's Association National Helpline, as well as to local newspapers that featured articles about the program.<br><br>intensive community outreach is critical for successful recruitment of program participants Initially, the Oregon agencies believed that capturing participants through existing caseloads would be sufficient. They also anticipated that large numbers of caregivers would have great interest in a free of charge, evidencebased program that was offered in their own homes. It was therefore initially a surprise to find recruitment for the program was an ongoing effort. In retrospect, both AAAs wished that they had started using paid advertisement more actively from the beginning of the grant to help develop familiarity and interest in the program. Outreach to community health care providers was also critical for generating referrals. The role of the Alzheimer's Association Chapter was (by their choice) limited to participation in a project advisory group and provision of general information about dementia care resources. Developing more active involvement between Alzheimer's Association Chapters and AAAs in future studies might help with outreach."
	Sustainability	Participating AAAs and coaches were enthusiastic about offering STARC to their clients, and have continued to make the program available since AoA grant funding ended in June 30, 2013.<br><br>The longterm sustainability of STARC (indeed any program) requires diligent attention and planning. Since grant funding ended, both AAAs have continued to offer the STARC program but overall costs and demands on staff time are a challenge given limited budgets. AAAs raised concerns early on about the sustainability of delivering eight weekly, inhome sessions, particularly for clients who lived in more remote locations where increased travel time and expense greatly increased intervention costs. As the OTS approached the end of AoA funding, discussions were held between the State Unit on Aging, AAA directors, and UW investigators to explore alternative delivery options, including possibilities such as offering STARC groups, switching to a telephonedelivery model, requiring fewer inhome visits, and/or scheduling sessions in consultants' offices rather than caregivers' homes. In response to the need for the AAAs to reduce the costs of service delivery, UW investigators reconfigured the original STARC from eight inperson sessions to four inperson sessions plus two telephone sessions (STARC2). This reconfigured STARC2 program—with inhome visits during Weeks 1, 2, 4, and 6, and telephone sessions in Weeks 3 and 5—includes the same content as the original STARC program. The AAAs have begun to test this program, and report that this reconfiguration has reduced concerns about staff time and program delivery costs, and has been helpful for recruitment of caregivers who had previously been reluctant to commit to so many inperson sessions requiring respite care. Although additional research is needed to evaluate the efficacy of this reconfigured version of STARC, caregiver and consultant feedback about the program has been very positive. Because the content of STARC was maintained in the reconfigured program, we believe the outcomes will be similar to the 8week program, but at this time, systematic outcome data are not available.
Milne et al. (2014) / UK	Acceptability Appropriateness	

		**(Outcome measurement) All sessions were rated at 3.9 or above; the highest score was 4.8. (out of 5). (...) A third (32%) of participants considered that they had a better understanding of dementia, a quarter (25%) had increased tolerance or patience, and a quarter (24%) said they had 'changed their approach' towards their relative. (...) A third (36%) of participants noted improved coping skills by the end of the Course and they had been helped by meeting others in the same situation**<br><br>**Each participant rated the value (usefulness) of each session – and if the session has more than one distinctive element each part – on a Likert scale of 1–5 (1 represented 'least useful' and 5 'most useful'). The second part invited answers to 8 open questions (Table 2). This methodological approach is commonly employed in small scale service evaluations (Bowling, 1997).**
	Sustainability	The first Courses for relatives with early stage dementia ran in two areas of Medway from 1995; three Courses were offered per annum. Although they stopped running in the second area in 2009,they continue to be offered in Medway to the present day.<br><br>That the Course continues to be funded to the present day suggests that it is considered to provide effectivecare and support to early dementia carers and value for money by commissioners. Whilst the findings of this evaluation are both local and small scale they have implications for national service development. The involvement of specialist psychologists in developing and delivering the Carers Course marks it out as superior to agencies providing 'information' alone or nonexpert advice. It is also the kind of programme that could be easily replicated and developed elsewhere in the country and, once established, is relatively economical to run.
Pleasant et al. (2016) / USA	Acceptability Appropriateness	Participants showed improvements in 'competency' (4/5 items), but 'keeping a positive attitude toward care recipients' declined. (...) Overall results indicated the CARES®Dementia Basics Program was successful in improving dementiaspecific knowledge and certain aspects of sense of competence in caring for persons with dementia.<br><br>**(Outcome measurement) The Sense of Competence in Dementia Care Staff Scale (SCIDS), developed and validated to assess competency in dementia care staff across multiple care settings. The Likert- type items within the subscale of Professionalism relate to motivation, attitude, the responsibility of caregiving and being part of a care team. Participants were asked if they could keep a positive attitude toward the care recipient and the family of the care recipient, be an active member of the care team, stay motivated, and provide personal care such as incontinence care.**
	Penetration	Oregon Care Partners was established to implement free training for all caregivers in the state, reflecting the Oregon Alzheimer's Disease State Plan recommendations<br><br>Participants were recruited through existing consumers of CARES®in Oregon and through networking with the Oregon Health Care Association.
Smith and Graves (2020) / USA	Acceptability Appropriateness	This project validated that the education intervention guided by"Taking Care of YOU: SelfCare for Family Caregivers Toolkit"(FCA, 2006) improved caregiver knowledge and selfcare. Based on the Stress Inventory, there was notable change in high risk/low risk categorization from pretest to posttest. Again, the educational intervention had a strong preventative effect. Even in the short period between pretest and posttest, there was an increase in caregivers' scheduled specific medical examinations for treatment and/or regular medical checkups.<br><br>**(Outcome measurement) The 16 items on the CSAQ questionnaire were summed to create a total stress score at pretest and posttest. Caregivers were also categorized into high-risk and low-risk groups according to their responses at pretest and posttest.**
	Penetration	quality improvement project used a convenience sample of 47 caregivers of patients with a diagnosis of dementia from a primary care location in Miami, Florida; investigator handed out flyers to announce the education sessions to patients/caregivers while in the waiting room
Exercise (n=3)		
Barrado Martin et al. (2019) / UK	Acceptability Appropriateness	"The intervention was widely accepted by participants who particularly adhered to the classbased component.<br><br>"Classbased exercises were useful but homebased exercises received some negative responses due to the booklet's poor fit/unsuitableThe homebased component was generally well perceived by participants who included Tai Chi practice in their routines. However, their acceptability was challenged due to their difficulties remembering the Tai Chi movements at home, which was not improved by the use of the homeexercise booklet.<br><br>age of participant must be considered this may not be appropriate for a user with late stage dementia "
	Penetration	Potential participants were initially identified and approached by three NHS Trusts in the South of England, as well as by the research team using the Join Dementia Research website where people living with dementia can express their interest in taking parting research. In addition, the study was advertised locally, allowing potential participants to contact the research team directly to express their interest in the study.<br><br>Recruitment materials included a leaflet, a key facts sheet, and a participant information sheet. These materials provided information regarding balance, falls prevention, Tai Chi, and the implications of getting involved in the study for each member of the dyad.
BarradoMartin et al. (2020) / UK	Acceptability Appropriateness	Most dyads did not report using the sheets provided to guide their practice; those who reported using it found it helpful.<br>Booklet Six participants found that the booklet worked well for them with no additional information required. Others reported that the pictures and description of the movement patterns were difficult to comprehend<br><br>(appropriateness) Unrealistic length of program content two carers pointed that the contents described would not fit the 20 minutes recommended practice

	Penetration	Recruitment sources included three National Health Service Trusts, 15 General Practitioner surgeries, the Join Dementia Research Website, the Alzheimer's Society and publicity (via flyers or facetoface events attended by Bournemouth University Team) across three different research sites in the South of England.
Prick et al. (2014) / The Netherlands	Acceptability Appropriateness	Most interviewed participants indicated that they experienced some benefits from the exercises: most people with dementia and almost all caregivers indicated increased pleasure and mood. Some people with dementia and a few caregivers indicated better selfesteem.<br><br>With regard to the exercise component, participants experienced pleasure, better mood, more selfesteem, increased awareness of the importance of exercise and improvement in the quality of the relationship. Furthermore, caregivers mentioned that doing exercises was a pleasant daytime activity in case of bad weather. With regard to the support component, named benefits were increased awareness of the importance of pleasant activities and decreased loneliness.
	Feasibility	Three quarters of the dyads completed six to eight home visits, which indicates that it was feasible to deliver the intervention at its current frequency and duration.
	Penetration	We started the recruitment with advertisements in national and local newspapers and on geriatric websites. These advertisements yielded almost no reaction from interested dyads. This was also true for personal letters sent to caregivers of people with dementia via caregiver organizations. Therefore, we changed our recruitment strategy into a personal approach to the dyads by giving presentations at local Alzheimer cafes(public meetings for people with dementia, their caregivers and others). We started with visiting Alzheimer cafes in the neighborhood of Amsterdam. Later, we expanded our visits to Alzheimer cafes throughout the Netherlands. Personally contacting potential participants or case managers was more successful. When contacting case managers, we asked them to look out for potential participants in their own caseload. Case managers contacted clients that seemed to be potential participants for this study and, if they were
	Sustainability	**Feasible but may require refinement:** Both the exercise and the support component may have different feasibility by moderate attrition to exercise and pleasant activities homework and some negative experiences of participants, which may be an indication of too intensive an intervention for this frail population in this specific region.
Care coordination and case management (n=6)		
Bass et al. (2003) / USA	Acceptability Appropriateness	Results of this demonstration suggest that care consultation delivered within a partnership between a managed care system and Alzheimer's Association may be a promising strategy for improving care for dementia patients and their caregivers. The beneficial impact of the intervention is evident in reduced use of selected managed care and community services, increased satisfaction with managed care services, and decreased caregiver depression and care-related strain.<br><br>**(Outcome measurement) The three measures include: a two-item index of satisfaction with the types of Kaiser services, an eight-item index of satisfaction with the quality of Kaiser services, and a five-item index of satisfaction with information received about the illness.**
	Feasibility	It is unknown whether this type of partnership can be established and yield similar benefits in other types of managed care environments or whether this type of partnership can benefit Medicare patients not enrolled in managed care.
	Penetration	The s**ample was drawn from Kaiser members whose medical records i**ndicated they had either a specific diagnosis of dementia or a symptom code indicating memory loss.
Bass et al. (2014) / USA	Acceptability	"PDC was associated with significantly less adverse outcomes, particularly for more impaired veterans. (...) Beneficial program effects after six months were evident in reduced relationship strain, depression and unmet need for more impaired veterans, and reduced embarrassment about memory problems for all veterans. "
	Implementation Cost	PDC is a lowcost service delivered by telephone, mail and email, with inperson contacts rarely needed. The two halftime care coordinators from the partnering organizations (one fulltime equivalent (FTE)) maintained caseloads of 75 to 125 families. All expenses to deliver PDC (that is, salaries, benefits, equipment, supplies, training, software, licensing, supervision, administrative overhead) can be recovered by charging a fee of $60 to $80 per month per family.
	Penetration	Participants were enrolled within the VA services
	Sustainability	The Benjamin Rose Institute on Aging holds the copyright to BRI Care Consultation and currently licenses and trains organizations to deliver the program. Since completion of PDC, more than two dozen diverse organizations have been licensed to deliver BRI Care Consultation, including healthcare organizations, Alzheimer's Association chapters, family counseling agencies and Area Agencies on Aging.
Bruce and Patterson (2000) / Australia	Acceptability Appropriateness	**not very acceptable carers felt staff did not validate or understand their experiences, and the referral system was inefficient use of ACT was often followed by institutionalization**<br><br>Referral by the GP to the ACAT was virtually the sole method by which these carers gained access to formal support. Our knowledge of the local system of care suggests to us that this referral process is likely to be the most common route of access for dementia carers in Western Australia. Referral for community supports occurred late, despite prolonged and often severe carer stress. Perhaps not surprisingly in these circumstances, referral to ACAT was often quickly followed by permanent institutional care.
	Penetration	Livein carers of dementia su�erers who had been seen by members of the Fremantle Aged Care Assessment Teams between January 1996 and June 1997 were invited to take part in this study
	Sustainability	**embedded into health system and previous evaluated; this study examines barriers**<br><br>In Australia, Aged Care Assessment Teams(ACAT) were set up by the Commonwealth Government of Australia initially for assessment for institutional care, but their role has developed to include assessment for a variety of community support programs ; GP refer patients to use ACAT
Connell and Kole (1999) / USA	Acceptability	The conference was well received as determined by the participants' evaluations. As a result of the session on community resources, physicians became aware of the services provided by the Detroit chapter of the Alzheimer's Association.
	Penetration	The publicity and promotion work group developed a program brochure and distributed 3,000 copies with a cover letter to dementia service providers, family caregivers, health care professionals, and community and voluntary agencies. Separate mailings were targeted to local newspapers with a suggested press release and to Thumb Area physicians encouraging their involvement in the

		program. Radio programs described the project and articles appeared in local newspapers, newsletters, and hospital bulletins. The community forum, which was open to the public, took place in November 1993. Fortytwo people attended. A 1day program on dementia followed, attended by health professionals, service providers, and family caregivers.<br><br>The COEP addressed this problem by actively recruiting additional advisory board members from agencies that served primarily minority clients. Their efforts were successful, particularly in the recruitment of a community organizer who was knowledgeable about issues related to dementia faced by clergy and congregation members in the African American church.
	Sustainability	**intervention developed in partnership and has been embedded in services** The COEP was established as a communitybased initiative, built on a partnership between the project team and the community. Such partnerships can facilitate the definition of locally relevant public health issues, the development of culturally appropriate interventions, a sense of project ownership, and the process of creating sustainable community change<br><br>[midMichigan] The advisory board continues to serve in an advocacy role to increase awareness of dementia, coordinate existing dementia services, and develop new services. Dementia training sessions for public safety officials were held in both counties several months after the completion of the program. These sessions represent an important step in the transfer of the project to the community because they were planned by the advisory board with minimal input from the COEP team.
Laparidou et al. (2018) / UK	Acceptability	Caregivers felt that HCPs require more training and education about the needs of caregivers/PwD as well as provide them with more useful information.
	Penetration	Informal caregivers of people with dementia were recruited by various methods, including through carer support groups (from different geographical areas of the rural county of Lincolnshire), the University of Lincoln, and a Patient and Public Involvement (PPI)Group. HCPs were recruited from two Lincolnshire general practices (in different geographical areas) and the main mental health provider of care for patients and caregivers of people with dementia within Lincolnshire. Although our focus was on **HCPs working in primary are, we included staff from the local Dementia and Specialist Older Adult Mental Health Service, as they received referrals from community health professionals regarding patients with dementia.**
Mavandadi et al. (2017) / USA	Acceptability<br>Appropriateness	In addition, the findings suggest that collaborative dementia care management is effective in realworld settings, as analysis of caregivers of older adults enrolled in the SUSTAIN program allowed us access to a unique sample of geographically diverse, communitydwelling, informal caregivers who were offered services through their care recipients' health program.<br><br>Offering caregivers a program that is telephonebased; individually tailored to take variability in care recipient/caregiver needs, preferences, and comorbidity into account; manualized (which enables immediate and convenient access to program material at the caregivers' convenience); and brief (i.e., 3 months) in duration may help overcome some of the logistical issues that serve as barriers to engagement in caregiverbased interventions and minimize caregiver strain. By scheduling calls based on the caregivers' availability and taking a tailored approach to the delivery of care, we were able to improve access to and engagement rates among caregivers who may otherwise not have participated.
	Penetration	440 older adults enrolled in the SUSTAIN program who received pharmaceutical assistance from the Pennsylvania Department of Aging's Pharmaceutical Assistance Contract for the Elderly/Pharmaceutical Assistance Contract for the Elderly Needs Enhancement Tier (PACE/PACENET) program.
	Sustainability	Since 2008, **SUSTAIN has been funded by PACE/PACENET as a clinical program**; thus, the research portion of the project specifically relates to the evaluation of clinical data collected from beneficiaries and caregivers enrolled in SUSTAIN's various services and followup data collected specifically for program evaluation purposes.
Occupational therapy (n=3)		
Burgess et al. (2020) / UK	Acceptability<br>Appropriateness	Pairs reported feeling that the occupational therapist had really listened to what they had said andunderstood what was important to them when agreeing their goals, referring to'working together'.They valued how the therapist had enabled them to go at their own pace as well as feel in control bybeing active partners within the process.<br><br>The appropriate support provided at the optimal time can reduce carer stressand subsequent care home admission. It is important for people with dementia and their families toplan for the future. COTiDUK contributes to this by providing the carer with skills and copingstrategies to use in the shortand longerterm, as well as adapting the environment to optimiseactivity engagement.
	Penetration	Participants were identified and selected from participants within the COTiDUK RCT (purposive sampling)
Field et al. (2019) / UK	Acceptability<br>Appropriateness	Participants appeared to be receptive to the intervention when they were struggling to adjust to the diagnosisor cope with symptoms such as memory difficulties, lack ofinitiative or reduced activity levels.<br><br>The timing of the intervention offeralso seemed to influence uptake, alongside people's experiences of adjustment, symptoms or coping. For most, it wasimportant to offer the intervention early after diagnosis.
	Penetration	Participants were recruited to participate in the occupational therapy intervention from two NHS sites in England as part of the VALID programme's development phase **(selected from existing program)**
Gitlin et al. (2010) / USA	Acceptability<br>Appropriateness	Of 20 caregivers with surveys, more than 80.0% reported a "great deal" of knowledge and skill enhancement in dementia understanding, home safety, and communication. Also, 68.4% reported enhanced ability to engage dementia patients in daily activities. Similarly, most caregivers reported "a great deal" of overall benefit, enhanced confidence managing behaviors and caring for patients, reduced upset, and selfcare<br><br>close to 90% believed that ESP did not require too much effort; 78% indicated just right number of sessions; 17% indicated that it was too much; and 5% too little.<br><br>**(Outcome measurement) (measuring care provider perspective) Surveys with 3-items were used to evaluate difficulty of implementation into practice.**

	Adoption	Of those surveyed, therapists indicated training components supported learning ESP (100%) and facetoface training strategies (roleplay, videos, case stories, and PowerPoint presentations) helpful (95%).<br><br>Most therapists expressed no difficulties introducing ESP (72%), integrating ESP into patient treatment sessions (60%), completing assessment forms (56%), identifying caregiver concerns (62%) and depression (62%), educating about stress (88%), and practicing stress reduction (60%). However, therapists indicated “some” to “a lot of difficulty” using treatment documentation (75%) and using problem solving (62%) with caregivers. Most (95.5%) therapists indicated intent to con¬tinue using ESP
	Feasibility	A second challenge involved fitting fidelity checks within agency and therapist practices. **Randomized trial methodologies (rating audiotaped sessions and direct observation) were not costefficient or part of agency culture**. In addition to strategies for strengthening and monitoring fidelity, we manualized aspects of ESP not previously developed (scripts for providing education materials).
	Fidelity	To enhance fidelity, we followed Lichstein, Riedel, and Griev (1994) recommendations and provided manual of procedures, guiding scripts, treatment documentation forms, and training through active learning with the original primary investigator and research interventionist.<br><br>Documentation was reviewed by the agency project coordinator and research interventionist following case completion. Select items from the caregiver posttreatment survey were used as indicators of receipt (treated with respect, adequate number of sessions received) and enactment (strategies used). Also, for each caregiveridentified problem, therapists asked caregivers upset with and confidence managing it to evaluate pretreatment and posttreatment benefits.
	Implementation Cost	Medicare, a major public resource for longterm care reimbursement, provides parttime skilled homecare including physical, speech, and occupational therapy to participants in outpatient clinics or homes. Whereas Medicare Part A provides reimbursement for traditional home health services and requires participants to be home bound, with Part B, a physician referral is required and patients with functional decline and/or safety concerns qualify for this service which can occur in outpatient settings or the home
	Penetration	Reaching OTs We identified 30 Fox OTs who met these criteria: employed at Fox for greater than or equal to 6 months, in good standing with Fox proce¬dures, policies and documentation requirements, and caseloads within the targeted geographic region. Of these, 23 (77%) agreed to participate and were trained.<br><br>Reaching participants/endusers Trained therapists subsequently identified caregivers from their respective active caseloads. Fox physical therapists in the targeted region also referred eligible patients and caregivers.<br><br>Over 2 years, therapists identified and approached 69 eligible caregivers for participation from active caseloads. Of those, 41 (59%) agreed to participate. Reasons for refusal included not identifying as caregivers, not perceiving need, or fear participation detracted from patient therapy, although this was explained as not true.
	Sustainability	**Private organisation involved in sustaining intervention:** Fox, an independently owned private practice, provides physical therapy, occupational therapy, and speech–language pathology services to older adults in homes, assisted living, or adult medical day centers in five midAtlantic states. Fox employs more than 350 clinicians of whom 120 are OTs. Therapists provide an average of 2,800 visits to approximately 930 patients weekly.<br><br>As ESP was designed to link caregiver training to the patient’s functional goals, through careful docu¬mentation, skilled therapists certified in ESP dem¬onstrated patient functional improvement and justified use of ESP as a Medicare Part B reimburs¬able intervention for the patient. All ESP sessions were reimbursed through the Medicare Part B carrier. This demonstrates the potential to sustain delivery through this funding mechanism when existing Medicare Part B requirements are met.
Multicomponent interventions (n=18)		
Burgio et al. (2001) / USA	Feasibility	The specific techniques selected can have profound implications for the **feasibility of implementing the intervention in the real world. For example, videotaping client behavior in the home, although an excellent enactment assessment strategy, may be too intrusive for some types of interventions and may even undermine intervention goals. In general, it is preferable in naturalistic settings to use strategies that are unobtrusive and maximally feasible.**
	Sustainability	REACH is a unique, 5year program sponsored by the National Institute on Aging and the National Institute for Nursing Research. REACH grew out of a National Institutes of Health initiative that acknowledged the welldocumented burdens associated with dementia caregiving, as well as the emergence in the literature of promising dementia caregiver interventions
Burgio et al. (2009) / USA	Acceptability Appropriateness	Greater than 93% of the participants indicated positive responses to this intervention for every question on the survey. Responses were slightly less enthusiastic about some components of the intervention than others.<br><br>Case managers were unanimous in their belief that the intervention was very helpful to the participants, especially people in rural areas who had no home computer, few social supports, and few community resources compared with people in urban settings. They noted that having a provider come to the home to focus on the caregiver was important, as most inhome services, such as home health care, typically focus only on the care recipient.<br><br>caregivers expressed very high degree of satisfaction and acceptability of the program, including evaluations of individual treatment components and number of treatment visits and phone calls.<br><br>**(Outcome measurement) The 21-item version used in this study included 16 items assessing caregiver burden and strain (e.g., How bothered are you about the care recipient’s memory problems?), 1 item assessing posi- tive aspects of caregiving (PAC), and 4 items relating to care recipient risk (care recipient smok- ing, wandering, driving, and lack of supervision)**
	Adoption	It was agreed that the intensive REACH II intervention used in the clinical trial was not feasible for use in the AAAs. The AAA case managers and supervisors were near saturation with the services already being provided. The intervention needed to be modifi ed signifi cantly, but in a way that caregivers and care recipients would still benefi t from participation.

	Feasibility	As reported previously, case managers reported delivering 95.2% of the treatment components to the sample at least once. Also, although we did not use a formal checklist during the workshops, all participants were required to demonstrate each skill component to the university trainer prior to moving on to the next training module.
	Fidelity	Treatment Fidelity Form.—This form allowed the case manager to record the number of home visits and therapeutic phone calls, and also listed all treatment components to be "checked off" if used during a visit.
	Penetration	Dyads were recruited by the AAAs. (…) Referrals to AAAs came from home health organizations, hospitals, physicians, caseworkers, selfreferral (e.g., families calling about respite services or fi nancial assistance), and outreach (e.g., advertising, health fairs). In recruiting the dyads for the REACH OUT Program, the case managers were provided much leeway in choosing dyads to invite to participate in the project. Case managers were already familiar with their current clients' basic needs.
	Sustainability	It is important to note that almost all aspects of the program were controlled by the AAAs with input from the ADSS. The University of Alabama collaborated in a genuine partnership with the AAAs and ADSS to modify the REACH II materials and intervention procedures so that they were feasible for use in the community.
Cho et al. (2019) / USA	Acceptability	Caregiver QoL (burden and depression) decreased following this intervention<br><br>**(Outcome measurement) Tool used to measure outcomes include Depression 10-item CES-D , Caregiver burden: Zarit caregiver burden interview, Social support: Social support composite (8 item), Self care – 11 item measure on self care (Belle), and Problem behavior (Revised memory and behavior problems checklist)**
	Feasibility	Outcomes of this study suggest that REACHTX is a feasible and sustainable evidencebased program to support family caregivers for people living with dementia (i.e., care recipients).
	Sustainability	in partnership with a nonprofit health care organization, an evaluation model that incorporated communitybased participatory research methods provided valuable evaluation data back to the funding agency (United Way of Tarrant County) who, based on outcome data, continued to fund the program.
Droes et al. (2019) / the Netherlands	Acceptability Appropriateness	"All people with dementia in the DemenTalent group and almost all people in the regular MCSP reported that they liked or very much liked the DemenTalent volunteer work(60% and 40%, respectively) and the regular MCSP (29%and 63%).<br><br>Also, all informal caregivers highly appreciated thevolunteer work of the person with dementia (50% beingsatisfied and 50% very satisfied)<br><br>As high as 79% of the caregivers who followed theSTAR eLearning course reported to have received a lot ofnew information and 21% some new information.<br><br>**(Outcome measurement) The informal caregivers of persons with dementia using DemenTalent and caregivers using Dementelcoach or STAR: (primary outcome measure) Short sense of competence scale34 and (secondary) emotional impact of neuropsychiatric symptoms (NPI burden subscale) quality of life, and happiness (by two TOPICS-MDS items). The Short Sense of Competence Questionnaire (SSCQ) consists of 7 self-report items, scored on a 5-point Likert scale from "strongly agree" to "strongly disagree", to assess the family caregiver's feeling of competence.**
	Implementation Cost	In addition, support was provided to all Meeting Centersin the experimental group for the acquisition of (startup and structural) funding from the municipalities (within theframework of the Social Support Act, WMO) for the implementation of the three new iMCSP interventions.
	Penetration	97% attended all four home visits. Of the 265 caregivers who completed all four home visits, 29 did not complete the posttreatment assessment, for a total discontinuation rate of 13%. The reasons for discontinuation were nursing home placement (12), care recipient death (7), and reason unknown (17). It is the AAA staff's opinion that many of the " unknowns " were related to migration related to Hurricane Katrina in 2005.
	Sustainability	To implement the new interventions, the staff of theMeeting Centers who were responsible for the implementation received a twoday training, followed by"coachingon the job"provided by the private company DirkseAnders Zorgen (DAZ), the Dementelcoach cooperativeassociation, and the Amsterdam Center on Aging of VUUniversity Medical Center (VUmc).<br><br>Information on the **content of the STAR eLearningcourse was provided to the Meeting Centers by VUm**c,which also arranged the course registration of prospectiveparticipants and provided a technical helpdesk for support.
Fortinsky et al. (2016) / USA	Fidelity	Our fidelity plan is based on a conceptualization of fidelity as composed of three components: delivery; receipt; and enactment; each of which can be enhanced, measured, and monitored, as used in the COPE efficacy trial and recommended by others [5,58,59]. COPE interventionists will complete fidelity checklists for each home visit for purposes of monitoring delivery, receipt and enactment of imparted strategies. Completed fidelity forms and COPE prescriptions will be reviewed for the first three dyads assigned to each OT interventionist. Interventionists also will participate in bimonthly phone calls with the investigative team to review case studies and discuss any challenges that might disrupt treatment fidelity. The study coordinator will conduct inhome fidelity visits for each interventionist at least once per year and complete a fidelity checklist and write up a report based on this fidelity visit.<br><br>As an important translational component of this study, COPE fidelity monitoring responsibilities will be transferred from Drs. Gitlin and Piersol to CCCI care managers after a minimum of two years of intervention implementation. Members of the investigative team will conduct COPE fidelity monitoring training for CCCI care managers. Fidelity monitoring forms from the original COPE trial will be modified with input from care managers and their director, to reflect charting methods routinely used by CCCI care managers to track all CHCPE services. As part of their service oversight responsibilities, care managers will then begin using revised fidelity monitoring forms to track home visits made by COPE interventionists. This approach seeks to assure continued maintenance of fidelity as the program becomes fully integrated into and normalized within routine practice. Evaluation of success of this fidelity transfer process will be an important component of the implementation process in this study."
	Penetration	During routine monthly clinical monitoring telephone calls, CCCIcare managers will explain key study features to provisionally eligibleclients' CGs and refer interested CGs and clients to the research studycoordinator. [Identify existing clients]

	Sustainability	As an important translational component of this study, COPE fidelity monitoring responsibilities will be transferred from Drs. Gitlin and Piersolto CCCI care managers after a minimum of two years of intervention implementation. Members of the investigative team will conduct COPE fidelity monitoring training for CCCI care managers. Fidelity monitoringforms from the original COPE trial will be modified with input from caremanagers and their director, to reflect charting methods routinely usedby CCCI care managers to track all CHCPE services.<br><br>As part of their serviceoversight responsibilities, care managers will then begin using revisedfidelity monitoring forms to track home visits made by COPE interventionists. This approach seeks to assure continued maintenance offidelity asthe program becomes fully integrated into and normalized within routinepractice. Evaluation of success of thisfidelity transfer process will be animportant component of the implementation process in this study.
Gaugler (2018) / USA	Acceptability<br>Appropriateness	Adult child caregivers indicated that the individual/family counseling (M = 4.55), the support group (M = 4.36), and ad hoc counseling (M = 4.00) components were all “helpful” to “very helpful” on the final item/overall rating of helpfulness.
	Feasibility	During the evaluation of the NYUCIAC, the research team found that adult children generally: (a) did not want to participate in as many as six sessions in 4 months; (b) viewed counseling sessions as a resource they could use per their needs; (c) wished to join support groups within the first 4 months of participation; and (d) were reluctant to include other family members or simply did not have any available to participate in the counseling sessions. For these reasons, the ultimate structure of the NYUCIAC was considerably different from the parent NYUCI.
Hendriks et al. (2018) / The Netherlands	Acceptability	Unforgettable program the trained people had an increased understanding of dementia and/or increased recognition of people affected by dementia as unique individuals with the same value as any other person.
	Fidelity	For the guides, the training started with a twoday introduction in which information about the occurence, symptoms, and consequences of dementia, and information about the Unforgettable method were provided. In the weeks following the twoday introduction, each trainee gave one pilot tour with people with dementia and caregivers participating. The hosts received a oneday introduction course about dementia and the Unforgettable method and joined a pilot tour.
	Sustainability	Continuous promotion appeared to be of great importance in all phases of the implementation. Letting possible users and referrers experience the program appeared to be essential. Some program coordinators lacked time to prioritize activities for the program, which often led to less promotion and consequently to lower turnout.<br><br>The Unforgettable museums met yearly in a meeting organized by the project coordinator of Unforgettable. In this meeting, they could share experiences and ideas, which was highly appreciated.
Menne et al. (2014) / USA	Appropriateness	Results showed that two caregiver outcomes, unmet needs and care efficacy, improved for RDAD enrollees, regardless of the number of sessions of exercise, behavior management, and dementia education. RDAD was associated with decreases in a broad range of unmet needs including in areas such as understanding memory and dementiarelated behavior problems. Improved care efficacy meant caregivers who used RDAD felt more capable and confident to manage their caregiving tasks and responsibilities after completing the 12 program sessions.
	Feasibility	RDAD was delivered by regular staff members of Alzheimer’s Association chapters across the State of Ohio. RDAD was integrated into chapters in a manner consistent with other information and support services routinely offered by these organizations.
	Penetration	Alzheimer’s Association chapters in Ohio promoted RDAD and recruited potential participants in a manner similar to how these chapters would promote their other service programs, such as through Help Line (information and referral) calls, flyers at community events, and presentations to local agencies and groups. A large proportion of IWDs and caregivers were recruited from those using other Alzheimer’s Association services (79%), with most other enrolled families recruited from Area Agencies on Aging or other members of the aging services network.
	Sustainability	For the Ohio replication, the interventionists delivering RDAD were clinical staff from the seven Alzheimer’s Association chapters that served the state, hereafter referred to as trainers. None of the Ohio trainers were physical therapists, although each had extensive background and training in dementia. Ohio trainers came from a variety of disciplines, including nursing, social work, counseling, and gerontology, and functioned as regular employees of Alzheimer’s Association chapters. **The goal was to implement RDAD as part of usual Alzheimer’s Association services.**
Meyer et al. (2018) / USA	Acceptability<br>Appropriateness	A suggestion that emerged from the data was tailoring the intervention to what the participants needed,such as asking people if they want to do Tai Chi or some other stress management technique. Some participants felt that Tai Chi might be too time intensive for caregivers whowere already stretched thin.<br><br>Stakeholders were generally supportive of the proposed intervention but had recommendations related to recruitment and engagement of the community and intervention content.
	Penetration	Participants for both the interviews and focus groups were recruited through the local Alzheimer’sAssociation, community partners (two Asian specific organizations in the Sacramento area), and word-of-mouth.<br><br>Stakeholders highlighted the multiple ways that faithbased institutions, including both churches and temples, were important to the intervention. They were helpful in terms of engaging and recruiting from the Vietnamese population since a large majority attended church or temple.
	Sustainability	Both caregivers and professionals believed that there should continueto be a space for intervention participants to meet after the sixweek intervention was over.<br><br>The role of the interventionist was to facilitate a group that would over time, be sustainable and selffunctioning. Thus, community empowerment and ownership of the interventionwould help the intervention’s effectiveness and sustainability. A professional mentioned thateven when there were Vietnamesespecific programs in an area with a large Vietnamesepopulation, people did not attend. Thus, it was not a matter of availability of culturallyand linguistic appropriate services (even though those were rare), she suggested there needed to be momentum and initiative from the community and a demand for services.<br><br>Participants expressed the importance of maintaining support for caregivers long after the sixweek intervention was complete.
Milders et al. (2016) / UK	Acceptability<br>Appropriateness	

		Twenty-seven caregiver manuals were returned at the end of the study. The number of activities recorded in the manuals varied widely. Seven caregivers recorded less than 10 activities and other caregivers indicated ‘‘does daily’’ for an activity. Therefore, it is difficult to estimate the total number of activities that dyads carried out during the study. What is clear is that most activities presented were enjoyed by the person with dementia;77% of the activities were rated as ‘‘enjoyed a lot’’ and 20% as ‘‘quite enjoyed’’. Activities that were enjoyed best were presented more frequently. Popular activities were household tasks (e.g. drying dishes), current affairs, musical reminiscence, ‘‘life story work’’ and walking and stair walking exercises.<br><br>**Study 2 All 20 indicated that the manual was clear and that they had understood the material. Seventeen (85%) found the training useful and that it had increased their confidence to train caregivers.**<br><br>**(Outcome measurements) When the caregiver training was completed, trainers were asked to evaluate the training that they had received in a 17-item questionnaire, to be returned anonymously. At the six-month assessment caregivers received a 17-item questionnaire to evaluate the manual and the training that they had received.**
	Adoption	Overall trainers were very positive about the manuals and the training that they had received, indicating that the instructions were clear (13/13) and that they had understood the material (13/13); most also indicated that the training was useful for their area of work and had increased their confidence to train caregivers (12/13). According to most trainers, the caregivers in their group had understood the instructions (12/13) and had benefitted from the training (11/13).
	Fidelity	In turn, these trainers were instructed by a health care professional. This cascade model of training was expected to improve access to the intervention without greatly increasing the workload of health care professionals and the associated health care costs. Training manuals limited the time required for training staff and caregivers and helped to promote treatment fidelity<br><br>**Treatment fidelity:** To assess whether the trainers trained the caregivers in accordance with instructions, the research coordinator observed trainers delivering the caregiver training and rated their performance against eight predetermined criteria as good, adequate, or less than adequate.
	Implementation Cost	Additional costs included were venue hire, travel costs for the purpose of training and printing costs for the manuals. Costs of the research coordinator were not included as these were incurred for research purposes only.<br>In both interventions the intervention costs reflected primarily staff costs.
	Penetration	The participants were recruited via the care team of the person with dementia, the database of the Scottish Dementia Clinical Research Network or locally through health and social careorganisations. Fourteen trainers were recruited from charities and support organisations in the Northeast of Scotland.<br><br>Study 2 Thirty-six trainers were recruited from six different organizations in the Northeastof Scotland. Four were dementia charities and voluntary organizations, one was a social careorganisation and one was a health care organization.
	Sustainability	Sixteen (84%) caregivers intended to continue with the activities after the study had ended. The care and support organizations involved in the studies reported here were intending to continue presenting the training. Since the completion of our studies, several of the participating organizations have indeedapproached the authors seeking further training opportunities.
Mittelman and Bartel (2014) / USA	Acceptability Appropriateness	participation was associated with a decrease in caregiver depression and in severity of caregiver reactions to all three domains of problem behaviors (memory, disruption, depression). In addition, caregivers reported an overall increase in satisfaction with social support and assistance.
	Penetration	Implementation sites were selected by the Minnesota Board on Aging in cooperation with Area Agencies on Aging.<br><br>Marketing materials were developed jointly by the Minnesota Board on Aging and the sites. The service was marketed locally by the sites through their network of referral sources, educational activities of consultants, radio programs, newspaper articles, and newsletters.
	Sustainability	An additional indicator of success is the sustainability of the program: The state has made a commitment to continue to provide the NYUCI.ACT on Alzheimer’s, the Minnesota Alzheimer’s Plan implementation team, has developed an economic model in an effort to build the case for sustainable funding of psychosocial interventions such as the NYUCI. Social workers with master’s degrees will be targeted to receive either in person training or newly developed web based training in providing the NYUCI. Methods to ensure that counselors receive the ongoing expert and peer clinical supervision that is essential to maintaining the core elements of the intervention are currently being discussed
Nichols et al. (2011) / USA	Acceptability Appropriateness	Caregivers felt that the program benefited them, helped them better understand the disease and their role, and increased their knowledge and ability to provide care. The caregivers felt that the telephone support groups were away to align with others who were in similar circumstances, while learning and teaching from experience. While both individual sessions and support groups were positively received, caregivers reported that they would like more in-home sessions.
	Penetration	Because HBPC staff members recruited caregivers of their own patients, the number who declined or were not appropriate is unknown.
	Sustainability	REACH VA is also appropriate for primary care, particularly geriatrics primary care, in which there is an ongoing relationship between patient, family, and provider. With rising interest in patient centered medicalhome concepts of ongoing care coordination, REACH VA could provide a tool for primary care providers to help caregivers manage their family members at home. The intervention is designed to be staff user friendly, with scripts, talking points, and all caregiver materials collected in 1 place, and is currently being implemented in community settings by hospitals, universities, area agencies on aging, and other organizations. The interventioni s also being developed for use in a facility or office setting, rather than in the home, and for delivering all sessions over the telephone.<br><br>Policy incentive The VA is in the forefront of this movement. In May 2010, Public Law 111163 Caregivers and Veterans Omnibus Health Services Act of 2010 was signed into law. It will allow the VA to provide unprecedented benefits to caregivers who support the veterans who have sacrificed for this nation. The VA is discussing the feasibilityof implementing national options, including REACH VA,for caregivers. On a local level, several VAMCs that havenot previously implemented REACH VA have requested training. This growing interest in assisting caregivers reflects the statements of a REACH VA interventionist who said, “Investment in the caregiver is a direct investment inpatient care.”

Nichols et al. (2016) / USA	Acceptability	Caregivers also had an increase in their ability to provide care. During the course of the intervention, caregivers experienced significant decreases in burden, depression, anxiety, caregiving frustrations, stress symptoms (feeling overwhelmed, feeling like crying, being frustrated as a result of caregiving, being lonely), and general stress rating
	Adoption	Administration on all levels must buy into the intervention. It is not enough for top level leadership, in our case, Congress or VHA, to be supportive of the program, although this certainly helps. Local facility and clinic leadership and local clinicians must all be onboard. Timing of implementation is critical. When the clinicians were ready, a facility administration might have other priorities and when leadership was ready, clinicians might not be ready. Securing coordination became part of site readiness preparation—to encourage discussion between clinicians and administration before training and certification.<br><br>adapting to other needs REACH VA is being expanded for caregivers of individuals with multiple sclerosis and post-traumatic stress disorder. Each will have its own Caregiver Notebook and Risk Priority Inventory that highlights the needs of caregivers of individuals with the targeted condition. The REACH VA model is standardized and structured, but it is shaped by parameters of the clinical condition and draws upon best practices in managing that condition.
	Feasibility	The REACH intervention has proven to be simple to use and robust in its results. Neither decreasing the number of sessions nor changing the method of delivery from primarily facetoface to primarily by telephone has negatively impacted outcomes. Staff from many different disciplines and levels of training have used it successfully.
	Fidelity	strategy to enhance fidelity (implementation) During the move to full operation of the four-session model, several implementation drivers were critical: training, consultation and coaching, administrative support, and system innovations. The Caregiver Center included more roleplaying, feedback, and examples to help staff better understand the intervention and their role. Certifying interventionists by videoconferencing has been extremely useful because it allows immediate feedback as interventionists roleplay the intervention. From REACH I through REACH VA Program Phase 2, interventionists range from a variety of disciplines including social workers, psychologists, nurses, and other social and behavioral scientists, and at a variety of levels from bachelor to graduate degrees.
	Penetration	From February 2011 to May 2012, REACH VA trained 30 sites and 124 staff and certified 85 staff in the 12 session model.<br><br>Staff at the individual VA Medical Centers identified caregivers and patients in their clinical panels who fit the REACH II criteria for enrollment.<br><br>scaling up In many ways, the VA's size, number of facilities, and large catchment areas add a complexity that smaller agencies would not have. Training, certification, and caregiver interactions would be onsite or more likely to be local, rather than national or regional.
	Sustainability	In 2010, a change in law and regulations allowed the VA to provide care for caregivers in addition to veterans. Because family caregivers provide most of the care to these patients, the VA has developed multiple initiatives to facilitate clinicians working with caregivers. REACH VA is one of the caregiver centered programs implemented during this change.<br><br>Since rollout of the four session model, the Memphis Caregiver Center has provided training, certification, and coaching to 151 VA sites in providing the intervention, a 500% increase, with 444 staff trained and 265 certified. Data from a Return on Investment evaluation of REACH VA training conducted by the VA's Employee Education System, showed about 68% of those trained are certified within three months with others taking six months or longer due to job constraints.<br><br>The Caregiver Center continues to train staff at new sites but also provides retraining for sites where certified staff have left. In addition to retraining staff at these sites, new marketing must take place to highlight the benefits of the intervention and assistance must be provided in placing the intervention in the appropriate context in the clinical setting. REACH VA has become a popular intervention for trainees to deliver. They are provided with specialized training and skills and consultation that their site may not be able to offer, and the site is able to provide the intervention to stressed caregivers.<br><br>**funding** The National Institute on Aging has recently provided funding to investigate patient and caregiver healthcare costs for REACH II and REACH VA Clinical Translation. If there are positive findings, organizations may be more likely to invest in behavioral interventions for caregivers.
OrsulicJeras et al. (2016) / USA	Acceptability Appropriateness	We found that 80% of CGs and 65% of PWDs reported that the number of sessions were either just right or that they would have liked more sessions. CGs andPWDs rated as very convenient the location of their SHARE sessions and the times of day sessions were scheduled; they also found the length of the sessions acceptable.<br><br>A strong emphasis is placed on giving PWDs a voice in planning their own care. Simultaneously, allowing CGs an opportunity to hear, acknowledge, and validate PWDs' voices gives them a starting point from which to frame discussions on decision making. With assistance from a SHARE Counselor, this approach creates a safe and comfortable environment that enables care dyads to work at their own level of readiness to discuss and plan for what lies ahead. Once this strong foundation is built, the focus moves to enhancing the care dyad's communication skills, increasing their knowledge about dementia and available services in the community, and ultimately developing a mutually agreed upon future plan of care.<br><br>**(Outcome measurement) Using a 5-point Likert scale (4=very satisfied, 3=somewhat satisfied, 2=neither satisfied nor dissatisfied, 1=somewhat dissatisfied, and 0=very dissatisfied), CGs rated highly their SHARE Counselor's level of skill, helpfulness, and friendliness, as well as the usefulness of session information and materials**.
	Feasibility	Results indicate that SHARE Counselors successfully built sufficient rapport with care partners, as evidenced by 100% of CGs and 97% of PWD's stating they felt understood by and were comfortable having discussions with their SHARE Counselor. Almost all CGs andPWDs (97%) felt there was enough time to discuss the topics they were interested in. Similarly, with respect to establishing buyin to

		the program, most CGs (90%) and PWDs(94%) agreed the topics discussed in SHARE were relevant to their care situation and as a result would highly recommend the program to others in a similar situation (100% and 94%,respectively).
	Fidelity	The SHARE Counselors also received training on SHARE protocols and procedures, and participated in weekly one hour facetoface supervision sessions with the Principal Investigator (PI) and the Project Director (PD) to maintain fidelity to study protocols and prevent drift. Supervision also helped SHARE Counselors to ensure all content was covered while accommodating each dyad's tolerance for discussing sensitive topics. In addition, counselor trainees met weekly with their licensed counseling supervisor to complete their clinical training requirements. Materials provided to SHARE Counselors included a SHARE Counselor Manual, Counselor's Guide, and set of magnetic boards used to facilitate the care values and preferences discussions.
	Penetration	Participants were recruited over a 20month period from various social service agencies in Northeast Ohio.
Paone (2014) / USA	Acceptability	Caregivers reported a high level of satisfaction and high degree of perceived value from this program/intervention. Out of 105 responding caregivers, 85 (81%) said that participating in the program was very helpful to them. Another 17 caregivers (16%) said that this was somewhat helpful, and only 2 caregivers were neutral as to the helpfulness of the program (...) Ninety-four percent of these family members said the program was very or somewhat helpful in improving their ability to participate in assisting their relative to care for the person with the disease<br><br>**(Outcome measurement) Caregiver Experience Survey: Out of 105 responding caregivers, 85 (81%) said that participating in the program was very helpful to them.**
	Adoption	Eighteen FMC Consultants were trained in the program protocol and in all of the tools and processes.<br><br>the availability of the research team to provide technical assistance to the FMC Consultants was invaluable. Consultants initially had questions about eligibility of caregivers who were potentially appropriate for the program. The availability of the research team helped maintain fidelity to the original research, since there was not an implementation manual or written guide at the time. Even with clears election criteria and extensive published information about the intervention (including books, articles, and presentation materials), the Minnesota consultants confronted many nuances related to the selection criteria, implementation issues, and ongoing strategies which required consultation with the research team.
	Fidelity	Eighteen FMC Consultants were trained in the program protocol and in all of the tools and processes.<br><br>The availability of the research team to provide technical assistance to the FMC Consultants was invaluable. Consultants initially had questions about eligibility of caregivers who were potentially appropriate for the program. The availability of the research team helped maintain fidelity to the original research, since there was not an implementation manual or written guide at the time.4Even with clears election criteria and extensive published information about the intervention (including books, articles, and presentation materials), the Minnesota consultants confronted many nuances related to the selection criteria, implementation issues, and ongoing strategies which required consultation with the research team.
	Implementation Cost	"In the initial grant application, costs of the FMC program were projected to be about $3,500 per caregiver. The actualaverage cost per caregiver was somewhat more than this estimate. Costsof the program were calculated based on quarterly financial reports fromprogram sites provided to the evaluator over 3 years. The program costs wereprimarily the salary expenses of the FMC Consultants. Program costs differedwidely from site to site. The more rural sites had higher travel expensesrelated to logging more miles to get to participants' homes, attend trainings,and conduct marketing efforts but tended to have lower salary costs.(...) Time spent in recruitment, intake, assessment, and counseling of caregivers who started but did not complete the program remained in thecost analysis as well. Therefore, the total program site costs were spread overa small number of people. The first program sites served more caregivers (per consultant) than the later program sites. This is why the average costs rose year to year. In addition to tracking costs, the consultants tracked how they spent their time. Time logs identified activities and hours by (a) direct service and (b) administration, reporting, and training. In analyzing the time reports of the consultants, it was clear that a hefty proportion of time was spent on grantrelated activities that would not be experienced in an operational "steady state." The evaluator determined that from 35% to 50% of the time that the consultants were spending related to startup efforts in learning the program protocol or to grant requirements (e.g., regional meetings, grant reports, training meetings, fidelity monitoring, and other activities).(...) The program cost was considered high in relation to the costs of other caregiver services provided by these organizations. However, when compared to the costs of a nursing home stay, the program costs are modest.
	Penetration	The types of organizations serving as program sites included: nonprofit community based organizations focused onservices for older adults and caregivers (N=7), hospital sponsored serviceprogram for seniors (N=2), clinic based programs (N=2), county based program (N=1), nursing facility/assisted living based program (N=1), andAlzheimer's Association chapter (N=1). Three of the 14 program sites were located in the Twin Cities metropolitan area. Five were near larger cities (Duluth, St. Cloud, Mankato) and the rest were located in rural areas of the state.<br><br>Although all of the program sites actively pursued a variety of marketing and recruitment strategies to make the FMC program known to communities and caregivers, the total enrollment of 137 caregivers was less than was expected by program sites and state agents. Recruitment efforts were primarily at the local program site level. The Memory Care Consultants worked to build referral relationships and program awareness in their respective market areas<br><br>Two of the program sites were particularly successful in recruitment—the FMC Consultants at these organizations accounted for more than half of all caregivers enrolled in the 5year period. These organizations had longstanding market presence around caregiver support services and memory care support. Consultants that were successful appeared to have several traits: understanding of the norms and cultural preferences of caregivers in the area, strong existing relationships with local providers, active and resourceful in identifying potential referral sources, and persistent in promoting the program communitywide.
	Sustainability	In Minnesota, the NYUCI program is called Family Memory Care(FMC).Implementation began in 2007 through a grant from the US Administration on Aging to the Minnesota Board on Aging.<br><br>Minnesota consultants described the substantial training and clinical consultant mentoring that was provided through the State as "extremely important" to them for program adoption and ongoing implementation and to ensure adherence to the NYUCI program protocol. This training and mentoring by the NYUCI researchers likewise helped consultants "develop skills and build confidence." Consultants reported that they brought these skills to their usual care practices with other caregivers and families with whom they came in contact—thus enhancing support offered by the consultant

		to all caregivers they served (whether participating in FMC or usual care).<br><br>As of June 1, 2012, 11 sites had discontinued FMC program as a service offered by their organizations. However, some of these organizations were changing how they offered usual care to caregivers to incorporate elements of enhanced assessment, engagement of families, and caregiver education. A few of the trained FMC Consultants left their organizations and reported that they hoped to offer the program again. During this time period, some organizations experienced significant financial difficulties and a few closed their doors entirely. Obviously, this affected program continuity. Despite the challenges, FMC Consultants reported that their skills, knowledge, and abilities related to working with other nonparticipating caregivers increased as a result of this program. That knowledge would stay with them.
Samia et al. (2014) / USA	Acceptability Appropriateness	"The caregiver's manual is understandable; The manual tied in well with the material presented in the sessions;andThetrainer(s) followed the program content.<br><br>Maine caregivers were more confident, better able to manage the situation and expectations, and grew from the experience. They were less depressed and had fewer negative reactions to the care receiver's behavior. Although there was no significant improvement in caregiver mastery, scores remained stable over the course of 12 months, despite the progressive nature of dementing illness and likelihood of greater demands."
	Adoption	Two adoption themes identified in the focus group were organizational commitment, resources, and readiness for MSCP marketing; and investment in developing paid associate trainers either within the existing organization or partnership sites. Marketing and recruitment were resource intensive and greater success was experienced by the agencies with established relationships and champions in the location in which the training was offered. Depth in training resources with provisions for backup trainers was essential for program stability. Of the four original lead partners, two ADRC/AAAs participated fully in adopting the MSCP. They supplied the necessary infrastructure for trainer development and support, intake, screening, marketing, and program delivery. These agencies embedded the MSCP in their existing Family Caregiver Program (FCP). Each agency had at least two program trainers, with one assuming responsibility for program coordination and fidelity monitoring. These were well-established agencies that had a decentralized infrastructure for program delivery within their service area. These agencies also had experienced previous success with adoption of other EBPs. They created unique delivery models based on existing community resources such as satellite offices and senior and community centers while also forging new partnerships to reach beyond the traditional aging services network where 74%(n=78) of the MSCP trainings were offered. The most northern and rural ADRC/AAA also had a decentralized infrastructure, several certified trainers, and previous EBP experience. It embedded the program within its FCP but was challenged by the rurality of its territory and marketing demands. This agency met 58% of its MSCP training commitment (n=14 trainings), and 57% of the 6week trainings (n=8) were held with five or fewer participants. The agency reported relying more word-of-mouth for recruitment to the trainings, which was evident from participant data that indicated 26.1% learned of the MSCP from family or friends. The Alzheimer's Association had significant staff turnover during the3year project, including five MSCP trainers, the executive director, and marketing staff. For most of the project, there was only one trainer serving seven counties, the majority very rural, with the commitment to deliver 16 MSCPtrainings per year. In the end, the Association had no trainer resources and was unable to subcontract for this resource. The Association was able to deliver 92% of their contracted MSCP trainings (44), but over the course of3 years 45% of these trainings (20) were cancelled or rescheduled due to low enrollment or lack of trainer resources. Two new agencies were successful in adopting the MSCP, Maine's Department of Health and Human Services, and a private nonprofitdementiacare agency. These agencies invested in training resources, collaborated with established ADRC/AAAs to provide trainings in their serviceterritories, and they reached out to caregiver employees and contacts within their established networks to populate MSCP workshops. Their ability to be more selective and restrictive with their service territory was essential to their success as compared to the experience of the Association that had MSCP demands beyond available resources.<br><br>program adoption through diverse partnerships beyond those commonly defined as the aging network, was the most challenging. For the MSCP, adoption was affected not only by program demands but also the 2008 economic downturn.<br><br>Organizational adoption required that the program be a fit with the agency's mission and its ability to meet the minimal resource commitment for program development, marketing, coordination, training, and fidelity monitoring. A minimum of two certified trainer sat each organization was necessary for program stability, or agency willingness to subcontract for trainer resources. A strong community presence and relationships were essential. Public/private relationships evolved with the rollout of the MSCP which enhanced adoption potential when the aging services network provided the certified trainer, program materials, and intake resources while tapping into space, marketing, and client base resources of the partner organization. Typically, partners had a shared mission to serve the local community and strengthen the presence and access to their respective organizations.
	Fidelity	Of the four original lead partners, two ADRC/AAAs participated fully in adopting the MSCP. They supplied the necessary infrastructure for trainer development and support, intake, screening, marketing, and program delivery. These agencies embedded the MSCP in their existing Family Caregiver Program (FCP). Each agency had at least two program trainers, with one assuming responsibility for program coordination and fidelity monitoring. These were well-established agencies that had a decentralized infrastructure for program delivery within their service area. These agencies also had experienced previous success with adoption of other EBPs. They created unique delivery models based on existing community resources such as satellite offices and senior and community centers while also forging new partnerships to reach beyond the traditional aging services network where 74% (n = 78) of the MSCP trainings were offered. Trainers' fidelity checklists indicated adherence to core program elements, including use of caregiver manual, slides, and trainer manual; provisions for practice and debriefing; and hours of program delivery. Deviations were observed in group size and trainer model. Six to 12 participants per MSCP training was the goal. Thirty-one percent (n = 32) of the trainings included five or fewer participants, which were more prevalent in rural communities. The trainer model was adapted and piloted in one ADRC/AAA following the death of its master trainer/program coordinator early in the project. In an effort to continue the program, two family caregiver specialists were trained and certified for the MSCP and then they cofacilitated 6week sessions, each becoming an expert in 3 weeks of content. They had regular planning and debriefing sessions to ensure seamless program delivery with fidelity. There were no significant differences in MSCP outcomes for this agency.
	Penetration	Caregivers were recruited by the partner agencies using flyers, public service advertisements, community outreach, email, Website postings, program cross fertilization, and professional presentations.<br><br>Participants reported learning about MSCP via a variety of media. Forty percent learned from an ADRC/AAAs or the Association. Almost 20% learned from news print, 10% learned from support groups, and close to 10%learned from another service provider (typically a social worker, nurse, or physician).

	Sustainability	A formal train-the-trainer model with a specific protocol for associate trainer certification was developed and implemented. Associate training and certification involved attendance at a one day workshop, observation of a6week MSCP workshop, and then cofacilitation of a 6week workshop with master trainer observation and feedback. The original intent was to utilize both employee and volunteer trainers, who had dementia knowledge and experience, and ideally group facilitation experience. In total, 37 trainers were trained (12 master and 25 associate). All master trainers and 18 associate trainers were certified.<br><br>The MSCP ended with a statewide organizational structure in place to serve all 16 of Maine's counties with the ADRC/AAAs assuming the lead role in partnership with multiple and varied community organizations. An MSCP Associate Trainer curriculum was in place with plans to offer annual statewide trainings. This infrastructure enabled two additional ADRC/AAAs to join the MSCP and advance the program in their regions. Agencies will offer fewer MSCP trainings, likely one per quarter, when grant funding ceases. This will not meet caregiver demand necessitating significant efforts to forge public/private partnerships to sustain the program. Results from a cost analysis will establish recommendations for a caregiver fee, with provisions for scholarships, post grant funding.
Stevens et al. (2012) / USA	Acceptability	Over 82 % of caregivers said that they agreed the types of services offered to them and their care recipients were helpful, and over 98 % of caregivers reported that if they had questions, they knew where to get answers. In regard to the satisfaction with the quality of services provided by the FCP, 93 % of caregivers stated that help for them and their care recipients were provided in a caring and knowledgeable way.<br><br>All caregivers reported that the information provided to them regarding the original REACH II intervention components was helpful, and they were satisfied with the phone contacts received from FCP staff.
	Adoption	Resulting adaptations facilitated translation of the clinical intervention into a customer friendly program delivered in a format customarily used in healthcare settings. This was necessary to achieve acceptance from the clinical delivery staff, who would be key referral sources, as well as individuals to be served by the intervention.<br><br>Program staff met with the nurse directors, nurse managers, nurse educators, and nurses on each hospital unit and with the nurse managers and nurses of all internal medicine clinic teams. Placement of information packets, nursing education concerning the FCP, and training about the prompts within the electronic medical record (EMR) system were discussed with each of the respective units and teams. This customization and collaborative discussion enhanced the adoption of the FCP. All units of the large hospital (n = 9) and care teams of the internal medicine clinic (n=6) have fully participated in the FCP, representing 100 % adoption.<br><br>Integration of the FCP into existing structures and systems within Scott & White was critical to the successful adoption of the program, as it allowed program staff to screen a large number of potentially eligible Alzheimer's or dementia patients served by Scott & White. Two approaches were used: integration into the EMR and integration into nursing staff development programming.<br><br>Created in collaboration with technology specialists from the Scott & White Siemens Information Technology team and the nursing staff, two key questions were embedded into the hospital admissions EMR infrastructure across the entire hospital. If a nurse identified a person as having Alzheimer's or dementia or being a caregiver of a person with Alzheimer's or dementia, an automatic display would prompt the nurse to give the caregiver a "Caregiver Packet," our primary recruitment tool. These prompts electronically created daily reports of admitted eligible patients for the FCP staff and were critical for self-evaluation of the program methodology. FCP staff was able to monitor daily the number of patients identified with Alzheimer's or dementia who met our target criteria and attempted to make personal contact with those families.<br><br>FCP staff worked with the Scott & White Nursing Clinical Development staff to develop ongoing training methods about the FCP which occurred through staff meetings, newsletters, and emails. Education of over 300 nurses in the main Scott & White hospital and over 65 nurses and 20 physicians occurred in the main clinic. The educational training had three objectives: (1) train nurses in techniques to identify family caregivers in ambulatory and inpatient care settings, (2) foster awareness of procedures for referring at-risk caregivers to the FCP, and (3) provide long-term maintenance strategies for incoming staff. In the FCP, structured implementation of new skills was facilitated by nurse educators who were assigned to each hospital unit. Program staff worked closely with nurse educators to facilitate on-the-job training and continuing education throughout their designated hospital units. Long-term maintenance of new skills was encouraged through the integration of project goals into nursing staff development programs, such as including information about the FCP into the general nursing orientation. This incorporation ensured that program education was given to all incoming nursing staff during the year. Outreach was also conducted with other staff members, including social workers and geriatricians, throughout the hospital and clinics.<br><br>Based upon suggestions from the nurse educators and managers, we systematically launched the program in three of the nine nursing units of the hospital which had the highest volume of geriatric patients. Throughout the rest of the year, the program was<br>strategically launched in all other units of the hospital. Similar to the hospital roll out, strategic dissemination of the FCP throughout the targeted primary care clinic occurred in phases with FCP staff working in collaboration with the nursing and physician staff.
	Fidelity	To ensure fidelity to the REACH II clinical trial, all FCP intervention materials including the treatment delivery schedule (described in the "Delivering the REACH II Intervention Components via the Caregiver Notebook and Family Profile" section) were based on the REACH II intervention materials. Training and oversight of FCP staff by a member of the REACH II research team promoted fidelity to the intervention as well as specific training in the techniques used with caregivers. Staff members were master 'strained counselors. Additionally, the primary outcome measure (described in the "Evaluation framework and data analysis" section below) was developed and tested by the REACH II investigators to be consistent with the REACH II baseline assessment
	Implementation Cost	Generous support from the Rosalynn Carter Institute Caregiver Program as well as supplemental support from Scott & White Healthcare allowed for the translation of the REACH II intervention materials into A Caregiver's Notebook, a format that was familiar to consumers of support services and one that could be widely disseminated.

	Penetration	Furthermore, referrals to formal community services, such as respite care, mental health counseling, and benefits and options counseling were made to the partnering agency, the Central Texas AAA.
	Sustainability	Generous support from the Rosalynn Carter Institute Caregiver Program as well as supplemental support from Scott & White Healthcare allowed for the translation of the REACH II intervention materials into A Caregiver's Notebook, a format that was familiar to consumers of support services and one that could be widely disseminated.<br><br>At the institutional level, maintenance and sustainability of the FCP throughout the Scott & White Healthcare system has occurred through various mechanisms. Institutional support of the FCP, monetary, organizational, and verbal, continues to establish the FCP throughout Scott & White Healthcare. Ongoing education to the healthcare providers is conducted in staff meetings, hospital wide presentations, and lunch seminars. At the nursing level, the FCP has been incorporated as a component of the general nursing orientation, thus ensuring that education about the program is given to all incoming staff.<br><br>In 2011, Scott & White Healthcare invested $150,000 in the Family Caregiver Program to promote continued growth of the program. As a result, the FCP has been expanded into two new service regions, allowing a more robust evaluation of our translation of REACH II.
Werner et al. (2020) / Israel	Appropriateness	There was a significant main effect for treatment, with the enhanced treatment group reporting fewer depressive symptoms compared to the control group after the intervention was conducted
	Adoption	**aligning mission of intervention with agency agenda** Perhaps of greatest relevance was the difference between the primary mandate of the research study, namely adhering closely to a rigorous randomized controlled design, and the mandate of the Israeli Alzheimer's Association to provide service.
	Penetration	recruiting trainers Potential NYUCI counselors were recruited for the two-day training via an advertisement posted on several professional websites; 24 social service providers (88% female) were selected for the training from among 100 applicants, based on level of relevant education (in social work, psychology or related disciplines) and competence as reflected by having previous experience working with persons with dementia and their family caregivers.<br><br>Participants were recruited from January 2012 till August 2014 via the Israeli Alzheimer's Association hot line, support groups and from the local health and social services where the counselors worked.
	Sustainability	The research team communicated on a regular basis with the project coordinator who maintained steady contact with the counselors and reminded them to complete the assessments as required. (...) First, the counselors were not part of the research team, and were not previously trained in research methodology. They were experienced clinicians employed by community service agencies and reimbursed by the Israeli Alzheimer's Association for participating in the study. They were not supervised by the research team but by the agencies for which they worked and by the coordinator of the project, who as stated above, was paid by the Israeli Alzheimer's Association.
Support interventions (n=5)		
Mazurek et al. (2019) / Poland	Acceptability Appropriateness	**increase in formal support** At baseline, people with dementia from both groups reported the same level of informal support (P=0.20), but there was a significant difference in perceived formal support between MCSP and UC groups<br><br>In should be noted that unmet needs decreased in the opinion of MCSP carers (2.41 vs 0.05), but increased in the UC group (2.25 vs 2.53).<br><br>Moreover, an increase in formal support both reported by the persons with dementia and their carers was found to a much greater extent in the MCSP, than in the UC groups
	Feasibility	The program is offered in accessible locations that facilitate social inclusiveness and community integration and promoting social participation. This makes them more attractive than institutional day care and makes it easier for people to use support from an early stage of the disease.
	Penetration	Elderly people with dementia, who were subsequently admitted to the Department of Psychiatry in Wroclaw, were included in the study.
Meiland et al. (2005) / The Netherlands	Adoption	**Factors influencing adoption in organizations** The increasing number of initiatives concerning support for people with dementia and their carers left little room for a meeting centre in their region. Another impeding factor was the failure to recognize the surplus value of a meeting centre, despite existing gaps in the offer. (...) The arrival of a meeting centre can help overcome waiting periods for other services. Finally, having examples of meeting centres available proved an advantage, because people could form a clear image of the model and they could benefit from available materials and expertise, which reduced the time needed to set up a meeting centre. In the words of one initiator: "the initiators found the video (on the meeting centres) very illuminating and said it had improved their idea of what a meeting centre was".
	Penetration	"In two of the regions, attendance was very low, despite the fact that care and welfare workers had assessed this need to be high. Perhaps the taboo on the subject of dementia caused this.<br><br>Finding a sufficient number of participants was facilitated by using multiple strategies: informing referrers/referring organizations and potential participants of the existence of the meeting centre and getting the information across in various ways (oral as well as written information and visual materials). The programme coordinators experienced the fact that potential referrers sometimes had little time to be informed, and that information is not adequately distributed among other workers within organizations as a barrier. Recruitment was also hampered if the potential participants were not reached, for example because there was no welfare organization in the region, a lack of PR activities in the neighbourhood, or the information was presented in an inappropriate tone (a childish flyer for instance). The target group of the meeting centres is a difficult group to reach in any case: these are people with mild to moderately severe dementia, who have generally not yet had any dealings with the care and welfare services."
	Sustainability	The advantages are that programme coordinators gain more insight into behaviour, as well as starting points for their supervision/guidance, that a lot of support and safety can be offered because both the person with dementia and the carer receive support, and that the nature of the meeting centre is visibly open because people can walk in without an appointment, and they can try it out a

		few times first. Integration with other activities within the location also proved to contribute to the fact that people like coming to the meeting centre and not feeling like they are in an institution. (...) Family members or friends of the participants can also come to the meeting group and, like the people with dementia, utilize other activities or services in the building (take courses, have their hair done, visit the restaurant). The participants appreciate the programme and the programme can also be adapted to the participants' wishes.<br><br>A barrier to the continuation of the meeting centre support programme was lack of capacity in other facilities. This makes it increasingly difficult to carry out the case management task adequately and organize additional help quickly.<br>A facilitator to the continuation of the meeting centres was structural financing. Especially key figures from welfare organizations indicated that the chances of structural financing increased if the project was successful and the (scientific) evaluation of the support programme was positive. Financial impediments were obviously if the project made a loss or if the initiating organization was itself in financial difficulty.<br><br>Another factor that could impede continuation was the existence of waiting lists for the meeting centres. This would make people tend to neglect publicity, which would in turn result in referrers "starting to forget about" the meeting centres. An additional problem was that participants had often deteriorated so they no longer met the criteria of the target group by the time there was room for them.<br><br>Other factors that hampered obtaining and/or keeping the right group of participants were: (a) referrers were not familiar with the difference between meeting centres, day care and psychogeriatric day treatment, (b) a lack of suitable alternative facilities for people who were not part of the target group, (c) stagnation in follow-up day care, and (d) participants who had trouble saying goodbye to the meeting centre group. Finally, a few facilitating factors were: a suitable location and good staff. The qualifications of these factors were already described in the previous phases, but they were also considered important during the continuation phase.<br><br>Another factor that could impede continuation was the existence of waiting lists for the meeting centres. This would make people tend to neglect publicity, which would in turn result in referrers "starting to forget about" the meeting centres. An additional problem was that participants had often deteriorated so they no longer met the criteria of the target group by the time there was room for them.
van Mierlo et al. (2017) / The Netherlands	Adoption	Adoption within one center in Italy was difficult since it already had specific activities for old people and there was high resistance from elders of preexisting centers.<br><br>Poland Aligning intervention with meeting center was difficult since "day care is usually for people with mental or physical disabilities. Day care participants are usually passive, not interested in many activities"
	Penetration	In Italy, stakeholders had mixed experiences: the communication with some organizations went well: "with the healthcare organization the continuity of information was good. They have always communicated effectively about problems and they have referred people with dementia to us when there were problematic situations," but it was difficult with others: "The collaboration with other dementia organizations is growing, but this collaboration is not achieved with general practitioners and with other referrers like the hospital and the Alzheimer's unit." In Poland, most people with dementia and informal caregivers were often self referred. There was "good communication between families and specialists," which had a positive impact on the implementation of the MCSP. In the UK stakeholders expressed a clear problem in communication with referrers: "We need one main person to make contact with referrers, but we have no time to do this." Another stakeholder from the UK: "We need to let people know it is there, we need more publicity." As a result of this problem, the Meeting Centers in the UK had difficulties recruiting sufficient people with dementia and informal caregivers.<br><br>continuous PR in the region facilitated the implementation of MCSP in Poland, in particular: "the MCSP website, open informative meetings, PR in local newspapers and radio. Also support of the MCSP leader and directors of social welfare and the Department of Social Issues in Wroclaw." In Italy, however, a stakeholder involved in oneof the MC's with no waiting list thought thatthere was a lack of good PR, which impeded the implementation.
	Sustainability	In Italy and Poland, the financial continuation of the Meeting Centers was expected to be supported by the Municipality. According to a MCSP program coordinator in Poland: "Social welfare, as part of the Municipality, is going to maintain the MCSP."In Italy, a stakeholder mentioned that: "The Municipality has adhered to implementation of the MCSP because they represent a perfect integrationbetween social and health aspects."
van Haeftenvan Dijk et al. (2015) / The Netherlands	Adoption	Impeding factors were staff having problems adopting a person-centered way of working and needing more coaching on the job (micro level), problems regarding involvement of managers and referrals to the day care centre (meso level), and insufficient time and money for cooperation (macro level). (...) As a result, in our study most staff were already present (for many years) and they found it difficult to adopt a new way of working. The staff experienced a need for more on-the-job training on how to integrate the adaptation coping model in their daily work. This was less of an issue in the Meeting Centres study where new staff was appointed
	Feasibility	This process evaluation shows that the transition of nursing homebased day care centres for people with dementia into community based day care with carer support according to the MCSPmodel is indeed feasible, also for people in the more advanced stages of dementia.
	Penetration	Another problem was that the target group, i.e. people with early stage dementia, is difficult to reach and to recruit. Many people with memory problems have their hands full facing the diagnosis of dementia. (…) An important success factor in two of the CO day cares was the financial support by municipalities for recruiting people with memory problems without a formal diagnosis of dementia. This made it easier to recruit people in an earlier stage of dementia.
	Sustainability	The main stimulating factors were differentiating groups on severity of dementia, staff getting used to work according to the new philosophy, adjusting the programme to the needs and wishes of the participants (micro level), and stable and productive cooperation with referrers which were seen in some day care centres (meso level). The main impeding factors related to communication and

		cooperation (meso level): it was not always clear to referrers who constituted the target group of the new day care centre (mild or moderate dementia/with or without wheelchair). (...) At the macro level the national care policy (regarding cutbacks in financial arrangements for day care) caused uncertainty about the possibilities of continuing the new day care centre in the future.
van Rijn et al. (2019) / The Netherlands	Adoption	However, professional project leaders also need enough time to invest in the implementation of DemenTalent. Most project leaders worked as program coordinators in the Meeting Center and therefore had to manage the implementation of DemenTalent on top of their regular duties. Extension of their contract and/or extra personnel to share their tasks facilitated implementation, whereas no extra hours or no extra personnel were mentioned as one of the biggest impeding factors in implementing DemenTalent.<br><br>Organizational conditions: Support from the own organization was experienced as an important facilitating factor. Sufficient human and !nancial resources appeared more likely if DemenTalent was compatible with the vision of the organization, and the management supported the project. Conversely, the organization not being supportive impeded the implementation.
	Feasibility	The results show that the implementation was feasible and matched the needs of a subgroup of people with dementia, but that successfulness of the implementation is largely dependent of human resources, the collaboration network in the region and the dementia friendliness of a region.
	Penetration	Finally, the location and network of the Meeting Centers appeared to be important here as well: being located in a region with an active and effective care network facilitated reaching the target group of potential participants for DemenTalent.
	Sustainability	Nevertheless, eventually all Meeting Centers received the funding they needed from the local government, which of course facilitated the implementation. (…) The downside of being funded by the local government is that funding is never certain for more than four years (when policies can change after elections).

Table 8. Common trends identified between implementation strategies and implementation outcomes

ERIC Implementation strategies & reported influence on implementation outcomes	Details surrounding influence between the strategy and outcome (Details from studies)	Studies that report the relationship
**ERIC 33 – Facilitation (26/67) was reported as an implementation strategy that influenced acceptability (implementation outcome) in 23 studies**<br><br>**Potential hypothesis:**<br>Facilitation, provided by a human regulator/provider, as an implementation strategy will enhance the user's experience of the intervention.	[1] Feedback provided by the coach at the end of each session was seen as a useful addition and was considered easy to understand → participants considered the intervention program 'Partner in Sight' acceptable and feasible.<br><br>[2] Typically, the IT support person was required for the first 10 minutes of each meeting, after which they left. The facilitator would contact IT support by SMS during the meeting if any participants required calling by phone to resolve any problems → Participants with no prior experience of using VC often reported feeling nervous about its use. Following the completion of the programme, most were very positive about VC and its potential use in the future.<br><br>[3] Having a competent, proactive human being to talk to and troubleshoot was crucial for adjusting the system to fit users' needs. → Only a little over half (57.1%) of participants reported that they were satisfied with RAM, and only 59.2% felt it met their care recipient's needs, emphasizing that some users do not find RAM satisfactory.	**[1] van Knippenberg et al. (2017)**<br><br>**[2] Banbury et al. (2019)**<br><br>**[3] Mitchell et al. (2017)**
**ERIC 71 – use train-the-trainer strategies (26/67) was employed in 11 of 14 studies that reported fidelity as an implementation outcome**<br><br>**Potential hypothesis:**<br>Employing train-the-trainer strategies will enhance the providers' implementation fidelity by providing clear instruction and reduce information fragmentation when scaling-up interventions.	**[1]** For this project, intervention specialists received extensive training in the following areas: memory and cognitive processes, symptoms of dementia, implementing cognitive rehabilitation techniques, and working with CGs and CRs as a dyad. Intervention specialists received a total of 22 hr of initial training consisting of lecture, modeling, role playing, case examples, feedback, and discussion. → To ensure fidelity to the protocols, intervention specialists received supervision and feedback when initially working with dyads. On- going monitoring of ANSWERS was accomplished through biweekly conference calls, which allowed for discussion of cases and review of protocols, including length and content of sessions, modifying techniques, and working with dyads.<br><br>**[2]** Because one of the aims of the OTS was to examine the feasibility of training AAA case managers to administer STAR- C, after certification two randomly selected session recordings from each case were reviewed by UW trainers for fidelity to the treatment protocol. (…) Once certified, consultant fidelity was maintained through (a) participation in periodic group conference calls with UW investigators, (b) ongoing supervision with their individual regional coordinators to address clinical issues or logistical difficulties, and (c) attendance at subsequent training workshops, both as a way to refresh their own knowledge about the program and to enhance the group training by sharing their experiences with new consultants.<br><br>[3] Following training, therapists participated in three individual telephone calls with the research interventionist and five monthly group conference calls to discuss challenging cases, provide coaching, and reinforce fidelity to core treatment principles.<br><br>[4] All SHARE Counselors received extensive training from project staff on counseling aging persons, dementia signs and symptoms, caregiving, care values and preferences, and working with a dyadic unit. The SHARE Counselors also received training on SHARE protocols and procedures, and participated in weekly one-hour face-to-face supervision sessions with the Principal Investigator (PI) and the Project Director (PD) to maintain fidelity to study protocols and prevent drift.	**[1] Judge et al. (2010)**<br>**[2] McCurry et al. (2015)**<br>**[3] Gitlin et al. (2010)**<br>**[4] Orsulic-Jeras et al. (2016)**
**ERIC 71 – use train-the-trainer strategies (26/67) was employed in 23 of 40 studies that reported sustainability as an implementation outcome**<br><br>**Potential hypothesis:**<br>The use of train-the-trainer as an implementation strategy will enhance the routinization of interventions.	[1] To implement the new interventions, the staff of the Meeting Centers who were responsible for the implementation received a two-day training, followed by "coaching on the job" provided by the private company Dirkse Anders Zorgen (DAZ), the Dementelcoach cooperative association, and the Amsterdam Center on Aging of VU University Medical Center (VUmc).<br><br>[2] Program staff were trained to deliver intervention components using the training material available in the REACH II intervention manual. This included a formal training program followed by routine supervision from the first author (a REACH II principal investigator).	**[1] Droes et al. (2019)**<br>**[2] Stevens et al. (2012)**
**ERIC 69 – Use mass media (12/67) was employed in all studies that reported penetration as an implementation outcome**<br><br>**Potential hypothesis:**<br>The use of mass media as an implementation strategy will improve penetration outcomes.	[1] The Dutch Alzheimer Association disseminated information about the program via the following: (1) monthly meeting spots for people with dementia and their caregivers, (2) newsletters, and(3) their website and social media platforms, including Facebook and Twitter. (69)<br><br>**[2]** The use of social media technology can provide support to caregivers in a variety of ways: improving knowledge and skills, reducing isolation, linking caregivers with each other, and with other resources needed for caregiving<br><br>[3] This brochure could be used to encourage primary care providers to make referrals of caregivers who are in need of help, encourage IHS, Tribal, and AoA/ACL staff to become Coaches, and encourage local agencies and services that support elders or family caregivers to include REACH in their programming. However, for other areas without well-frequented community gath-ering places, other ways to publicize, such as radio and television PSAs and social media, may be a better strategy to reach caregivers.<br><br>**[4]** two strategies to increase public awareness of dementia were suggested: (a) a media advocacy session targeted to members of the board of the local chapter of the Alzheimer's Association and (b) a speakers' bureau training for volunteers willing to disseminate information about dementia to interested groups.	**[1] Boots et al. (2017)**<br>**[2] Pagan-Ortiz et al. (2014)**<br>**[3] Martindale-Adam et al. (2017)**<br>**[4] Connell and Kole (1999)**
